# Supplementary material for: Photocrosslinking-induced CRAC channel-like Orai1 activation independent of STIM1
Source: Nat Commun. 2023 Mar 8;14:1286. doi: 10.1038/s41467-023-36458-4 (PMC9995687; doi:10.1038/s41467-023-36458-4)
Supplement: Supplementary file 1 — Supplementary Information [file 41467_2023_36458_MOESM1_ESM.pdf]

# Supplementary Material

## Photocrosslinking-induced CRAC channel-like Orai1 activation independent of STIM1

Lena Maltan*[1], Sarah Weiß*[1], Hadil Najjar*[1], Melanie Leopold[1], Sonja Lindinger[1], Carmen Höglinger[1], Lorenz Höbarth[1], Matthias Sallinger[1], Herwig Grabmayr[1], Sascha Berlansky[1], Denis Krivic[2], Valentina Hopl[1], Anna Blaimschein[1], Marc Fahrner[1], Irene Frischauf[1], Adela Tiffner[1], Isabella Derler#[1]

* These authors contributed equally
# corresponding author

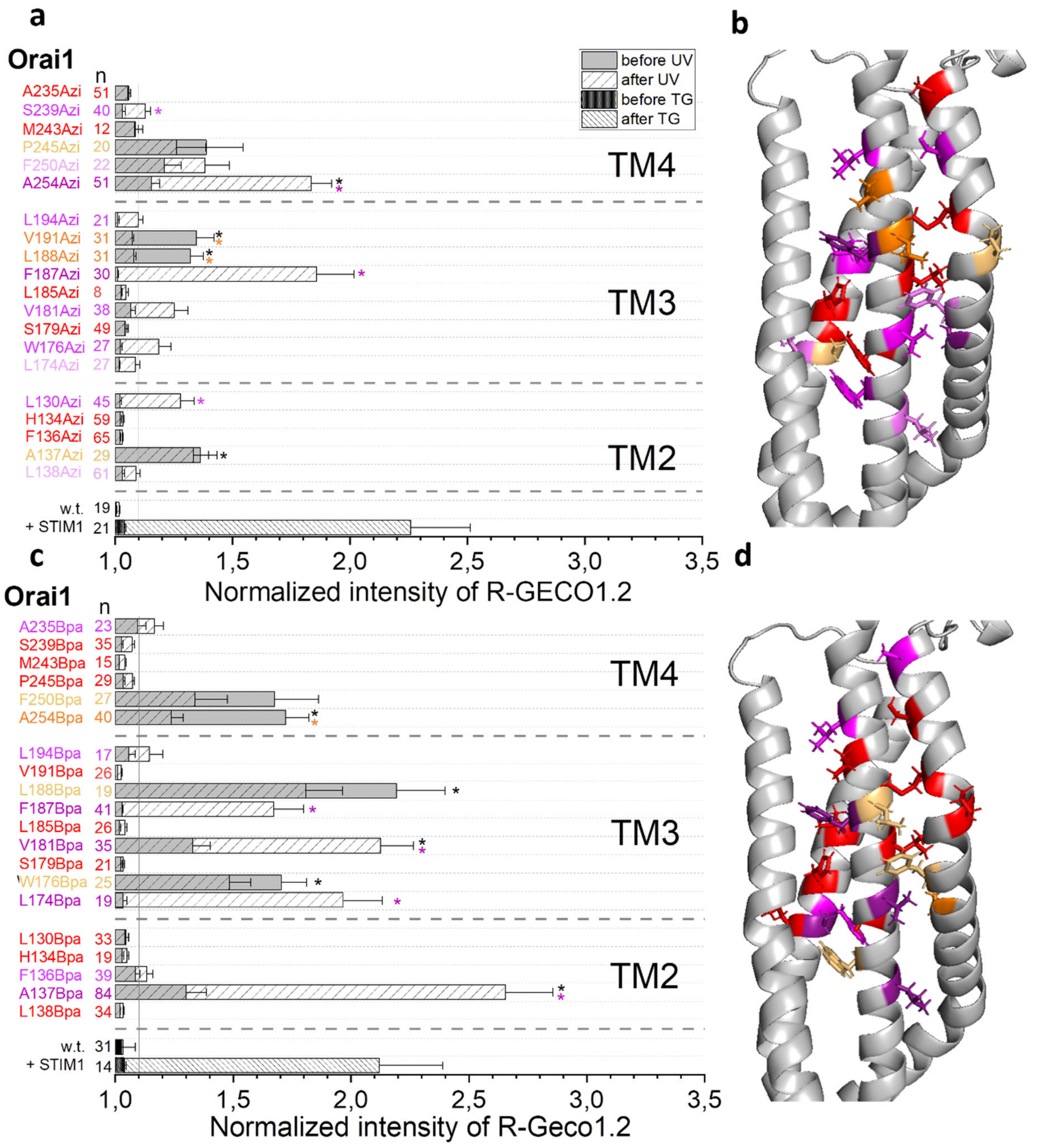


Supplementary Figure 1: **Incorporation of photocrosslinking UAAs at various positions in Orai1 is suitable to transfer light-sensitivity to the channel independent of STIM1. a) and c)** Bar diagram showing intracellular $Ca^{2+}$ levels, represented by normalized intensities of R-GECO1.2, before (filled bars) and after (hatched bars) application of 10s UV light for all screened Orai1 mutants incorporating Azi (a) or Bpa (c) in either TM2, TM3 or TM4. In comparison, the effects of wild-type Orai1 exposed to UV light as well as STIM1/Orai1 activation upon application of thapsigargin (TG) are shown (Welch-ANOVA for (a) and (c): comparing normalized intensity in 0mM versus 2mM $Ca^{2+}$-containing solution: $F(41;371,32)=10,02$, $p=0$ (a); $F(41;368,33)=7,79$, $p=0$ (c); comparing normalized intensity before versus after UV: $F(43;371,88)=11,55$, $p=0$ (a); $F(43;372,45)=15,29$, $p=0$ (c)). For normalized intensity of R-GECO1.2, a threshold (grey) line was set at 1.1 which means that changes below this line, even if significant, are considered inactive. Results correspond to the

Table in **Figure 1**. **b) and d)** Scheme highlights the effect of UV light at different positions exchanging the endogenous amino acid with Azi (b) or Bpa (d). Color code is comparable to the bar diagram and Figure 1. Data represent mean values ± SEM of indicated number (*n*) of experiments. *(black) indicate significant difference (p < 0,05) of values obtained in 0mM compared to 2mM $Ca^{2+}$-containing solution before application of UV light (filled bars), while *(colored) indicate significant difference (p < 0,05) before (filled bars) compared to after (hatched bars) UV light application. Detailed statistic values are shown in **Supplementary Table 3**. Source data are provided as a Source Data file**.**

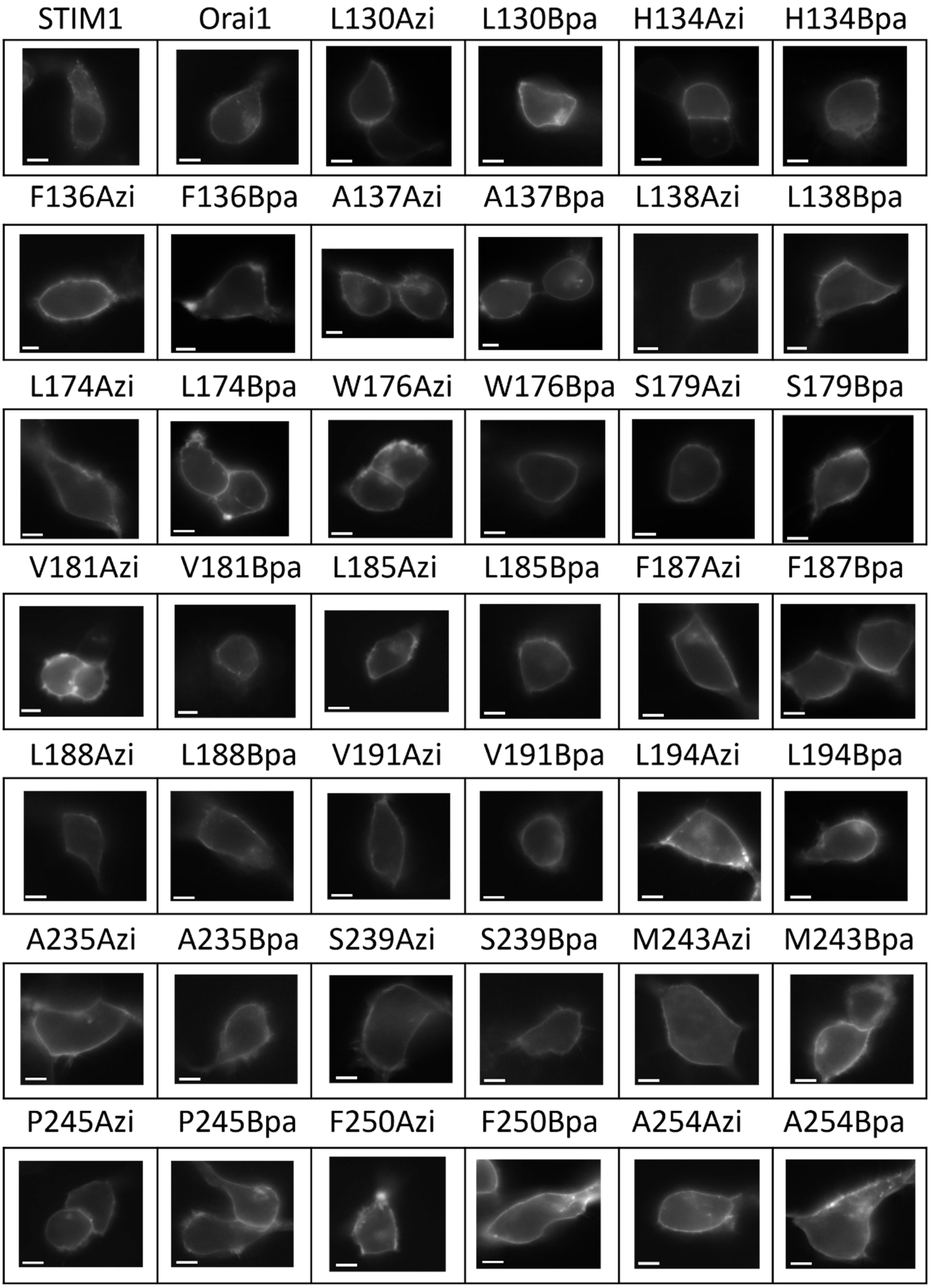


Supplementary Figure 2: **Insertion of UAAs retains plasma membrane localization of all tested UAA-containing Orai1 mutants.** Fluorescence images showing localization of overexpressed C-terminally labelled Orai1 UAA-containing mutants compared to wild-type Orai1 and STIM1. Positions used to mutate endogenous amino acids to UAAs (Azi and Bpa) are corresponding to Supplementary Figure 1. White bars indicate 5µm.

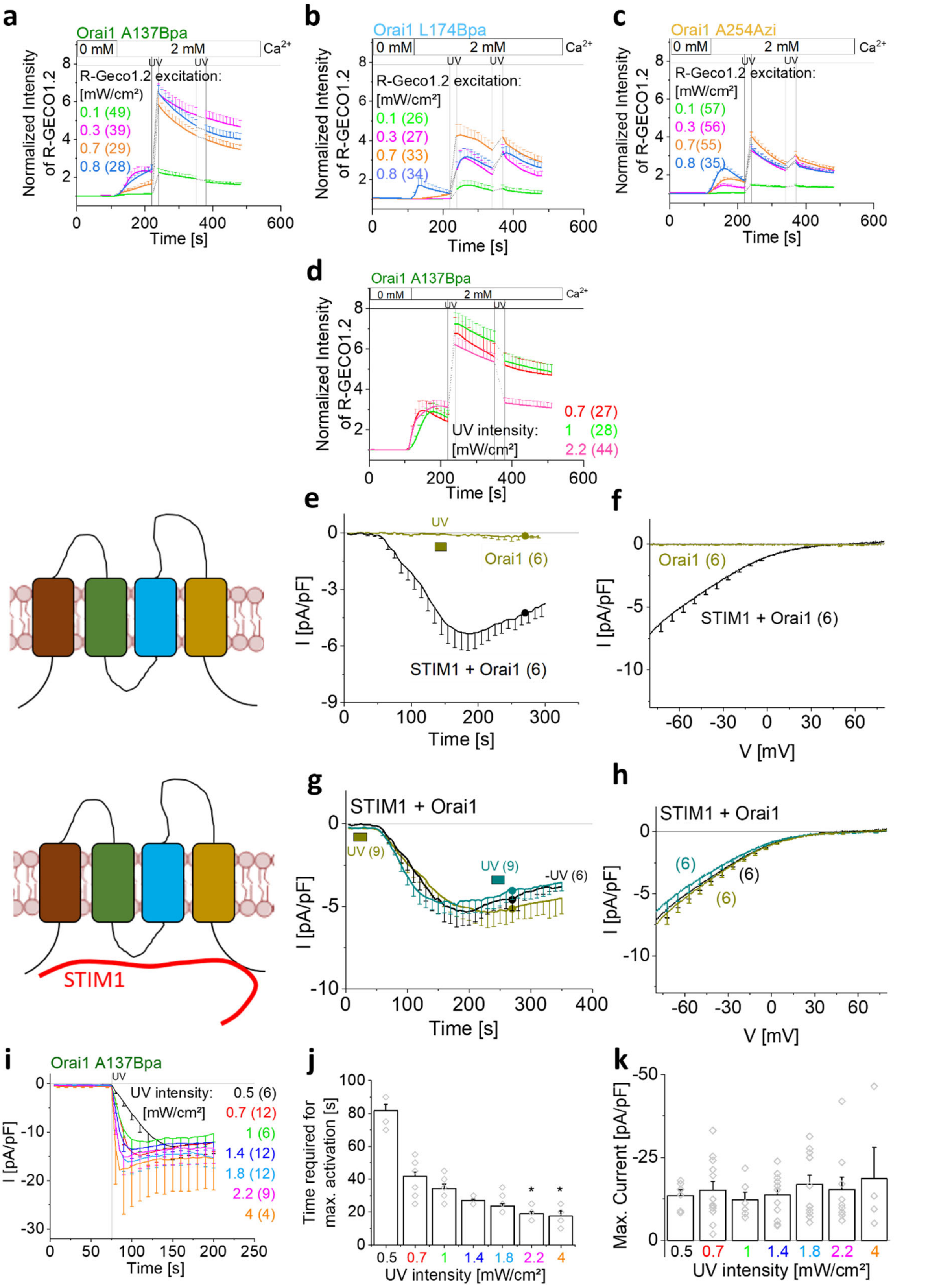


Supplementary Figure 3: **Intensities required to obtain maximum photocrosslinking-induced activation. a)-c)** $Ca^{2+}$ imaging measurements of Orai1 A137Bpa (a), Orai1 L174Bpa (b) and Orai1 A254Azi (c) detected via R-GECO1.2 fluorescence with different intensity levels (0.1/0.3/0.7/0.8 mW/cm²) of the *LedHUB®*. Intracellular $Ca^{2+}$ levels, represented by the

normalized intensity of R-GECO1.2 co-transfected with above mentioned UAA-containing Orai1 mutants in HEK293 cells, were monitored initially in 0mM $Ca^{2+}$ solution followed by a 2mM $Ca^{2+}$ solution. Under 2mM $Ca^{2+}$ solution conditions, UV light was applied for 10s and 30s. Due to filter exchange, R-GECO1.2 intensity could not be recorded simultaneously with exposure to UV light. **d)** $Ca^{2+}$ imaging measurements corresponding to a) detected via R-GECO1.2 fluorescence (0.3 mW/cm$^2$) while applying different UV light intensity levels (0.7/1/2.2 mW/cm$^2$). Schemes with one Orai1 subunit in the absence or presence of STIM1 as used under one of the two conditions shown in **e) and g)** Time courses of current densities after whole-cell break-in of Orai1 or STIM1+Orai1 expressing cells. UV light was applied for 15s in (d) at t = 140s or (f) either before (t = 15s) or after (t = 240s) passive store-depletion induced activation. **f) and h)** Corresponding I/V relationships were taken of current densities in (e) and (g) indicated by the respective circles. **i**) Time courses of current densities after whole-cell break-in of the light-sensitive Orai1 A137Bpa mutant varying the UV light intensity levels (0.5/0.7/1/1.4/1.8/2.2/4) of the *Lumencor Spectra III*. UV light was applied until maximum activation was achieved (t = 20 – 80s). **j) and k)** Corresponding bar diagram to (i) showing the time required for maximum activation at the different UV intensities ((j); Welch-ANOVA: F(6;16,91)=40,40, p=3,85*10$^{-9}$) or maximum currents at the different UV intensities ((k), Welch-ANOVA: F(6;18,35)=0,32, p=0,92). Data represent mean values ± SEM of indicated number (*n*) of experiments. *Significant differences ($p < 0.05$). Detailed statistic values are shown in **Supplementary Figure 3**. Source data are provided as a Source Data file.

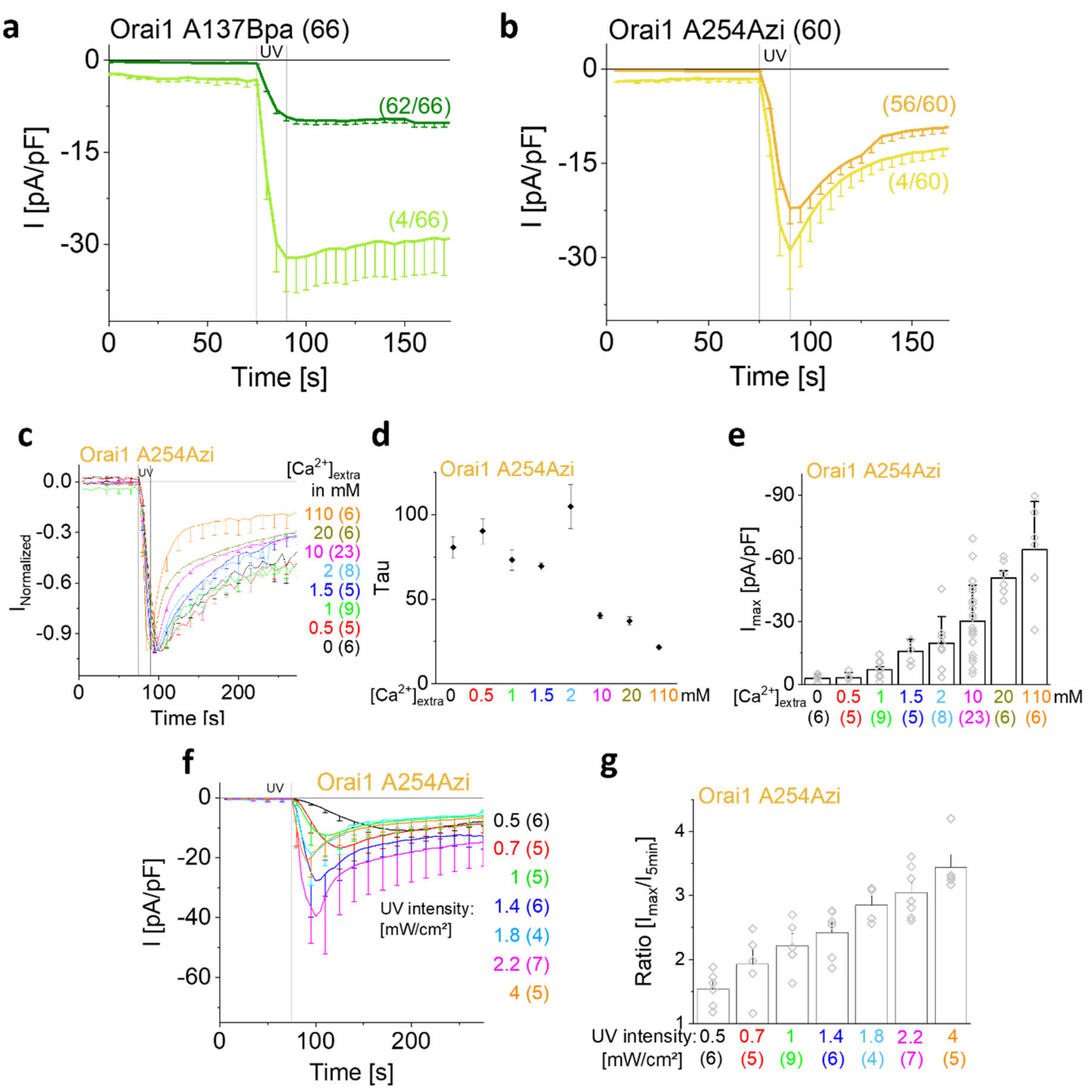


Supplementary Figure 4: **Constitutive activity of Orai1 A137Bpa and Orai1 A254Azi and slow inactivation of Orai1 A254Azi. a) and b)** Time courses of current densities after whole-cell break-in of the light-sensitive Orai1 A137Bpa (a) and Orai1 A254Azi (b). The numbers in parentheses indicate how many experiments (before the slash) from a pool of experiments (after the slash) are included in the mean. **(c)** Time courses of normalized current densities after whole-cell break-in of the light-sensitive Orai1 A254Azi mutant varying extracellular $Ca^{2+}$ concentrations (0/0.5/1/1.5/2/10/20/110mM $Ca^{2+}$). **d) and e)** Corresponding bar diagrams to (c) exhibiting $\tau$ for current decay after maximum activation (d) and maximum currents (e) at different extracellular $Ca^{2+}$ concentrations (Welch-ANOVA: $F(7, 26.77)=13{,}32$, $p=2{,}65*10^{-7}$). **(f)** Time courses of current densities after whole-cell break-in of the light-sensitive Orai1 A254Azi mutant varying the UV light intensity levels (0.5/0.7/1/1.4/1.8/2.2/4) of the Lumencor Spectra III. UV light was applied until maximum activation was achieved (t = 20 – 80s). **(g)** Corresponding bar diagram to (f) exhibiting the increasing ratio of $I_{max}/I_{5min}$ in dependence of enhancing UV light intensities (one-way ANOVA: $F(6, 37)=16{,}11$, $p<0{,}0001$). Data represent mean values ± SEM of indicated number (*n*) of experiments. Source data are provided as a Source Data file.

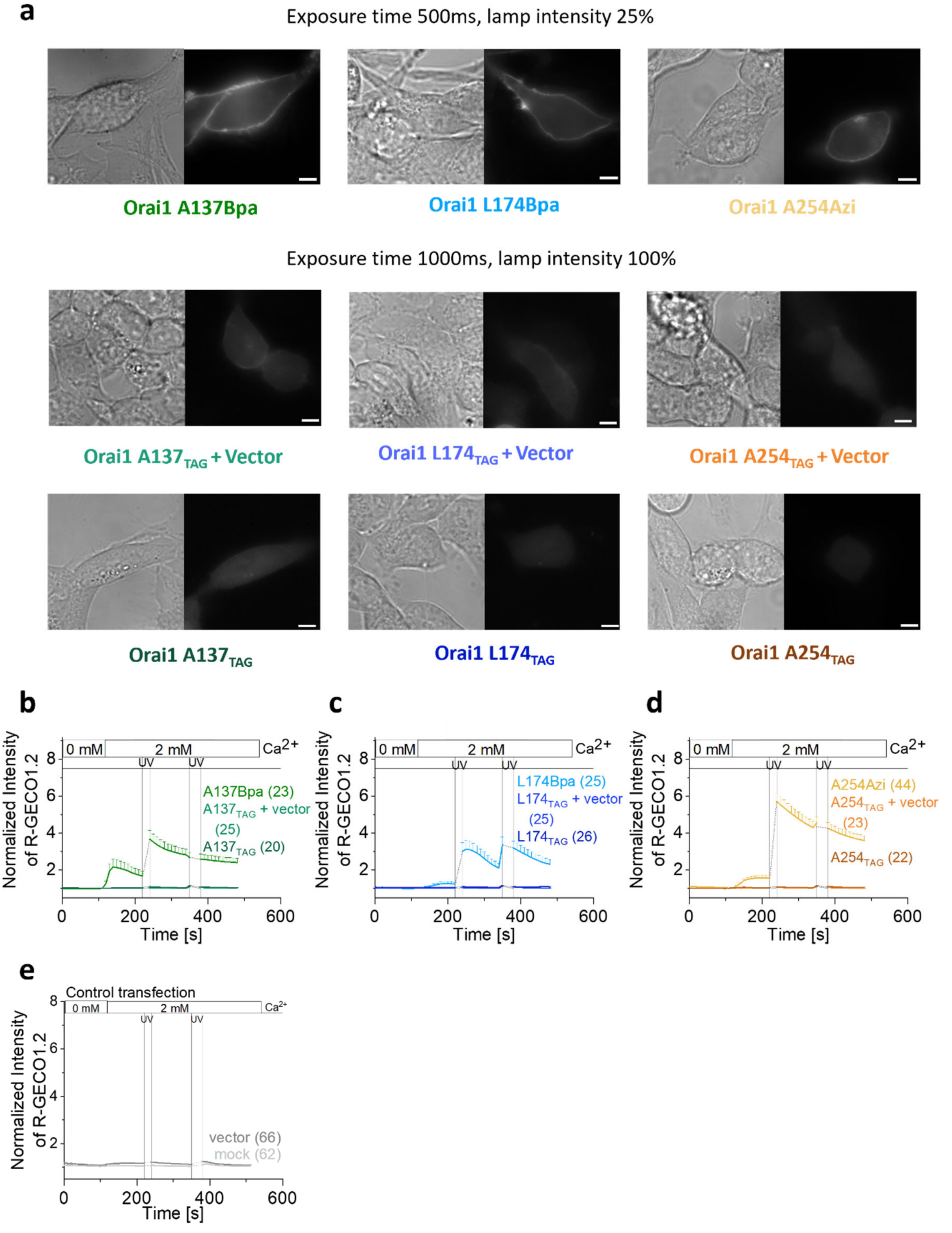


Supplementary Figure 5: **Intact plasma membrane expression and UV light-mediated activation is only obtained in the presence of the UAA and the respective tRNA/ aminoacyl synthetase pair. a)** Representative bright field and fluorescence images of Orai1 A137Bpa, Orai1 L174Bpa and Orai1 A254Azi while all components (UAA and tRNA/ aminoacyl synthetase pair) were available (upper panel; exposure time 500ms and 0.3 mW/cm² intensity of the *LedHUB*®), while the UAA was not supplemented (middle panel; exposure time 1000ms and 1.4 mW/cm² intensity of the *LedHUB*®) and while both, UAA and tRNA/ aminoacyl synthetase pair, were missing (lower panel, exposure time 1000 ms and 1.4 mW/cm² intensity

of the *LedHUB*®). White bars indicate 5µm. **b)-e)** $Ca^{2+}$ imaging measurements of Orai1 A137Bpa (b), Orai1 L174Bpa (c), Orai1 A254Azi (d) expressing cells and mock transfected cells (only Bpa-vector) (e) using R-GECO1.2 under conditions mentioned in (a). Intracellular $Ca^{2+}$ levels, represented by the normalized intensity of R-GECO1.2 co-transfected with above mentioned UAA-containing Orai1 mutants in HEK293 cells, were monitored initially in 0mM $Ca^{2+}$ solution followed by a 2mM $Ca^{2+}$ solution. Under 2mM $Ca^{2+}$ solution conditions, UV light was applied for 10s and 30s. Due to filter exchange, R-GECO1.2 intensity could not be recorded simultaneously with exposure to UV light. Data represent mean values ± SEM of indicated number (*n*) of experiments. Source data are provided as a Source Data file.

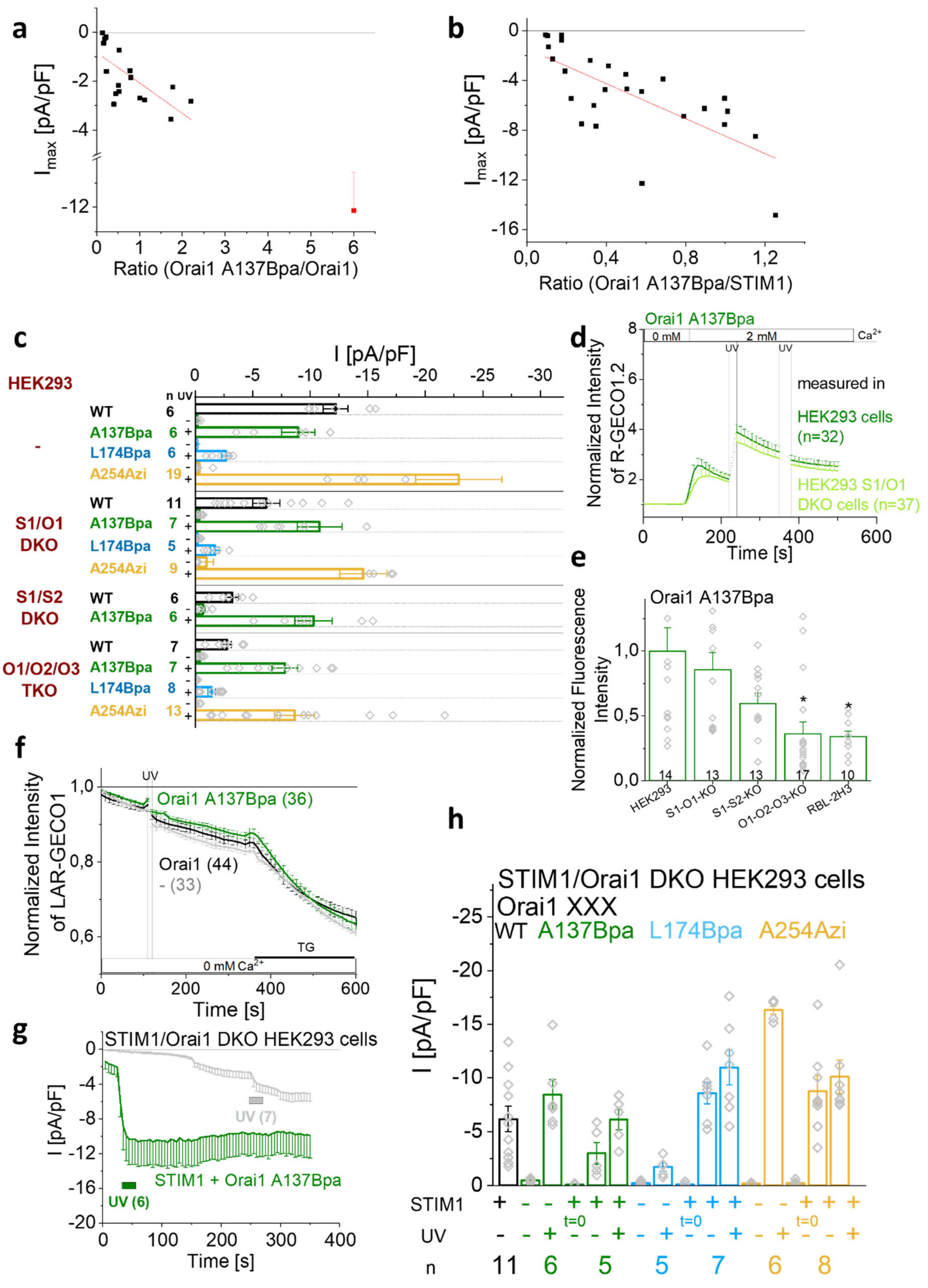


Supplementary Figure 6: **Maximum photocrosslinking-induced activation is reduced the higher the levels of co-expressed wild-type Orai1 or STIM1 a) b)** Scatterplot of maximum currents of cells co-expressing different intensity ratios of Orai1 A137Bpa and wild-type Orai1 after 15s UV pulse (ratio Orai1 A137Bpa/Orai1; Regression line (red; y = -0.82 ± 0.32 – (1.25

± 0.34)x)) (a) or of STIM1 and Orai1 A137Bpa after store-depletion and subsequent 15s UV pulse (Regression line (red; y = -1.42 ± 0.88 – (7.05 ± 1.42)x)) (b). In (a) for comparison, a mean value + SEM (red) is shown indicating UV-mediated currents of Orai1 A137Bpa (n = 11). **c)** Bar diagram showing maximum currents of Orai1 A137Bpa, Orai1 L174Bpa, Orai1 A254Azi and Orai1+STIM1 (WT) expressed in normal, STIM1/Orai1 DKO, STIM1/STIM2 DKO and Orai1/Orai2/Orai3 TKO HEK 293 cells, before and after 15s UV pulse (except Orai1+STIM1). **d)** Time course of intracellular $Ca^{2+}$ levels of Orai1 A137Bpa in normal compared to STIM1/Orai1 DKO HEK293 cells represented by the normalized intensity of R-GECO1.2 initially in 0mM followed by a 2mM $Ca^{2+}$ solution. Under 2mM $Ca^{2+}$ solution conditions, UV light was applied for 10s and 30s. **e)** Normalized fluorescence intensities of Orai1 A137Bpa-YFP in HEK293 cell lines mentioned in (c) and the RBL-2H3 cell line (Welch-ANOVA: F(4;29,94)=6,93, p=4,47*$10^{-4}$). **f)** Time course of ER-$Ca^{2+}$ levels in Orai1 A137Bpa- and Orai1-expressing cells monitored by the normalized intensity of LAR-GECO1 in 0mM $Ca^{2+}$ solution followed by a UV pulse (10s) and TG application. **g)** Time courses of current densities after whole-cell break-in of the light-sensitive Orai1 A137Bpa mutant co-expressed with STIM1 in STIM1/Orai1 DKO HEK293 cells. UV light was applied for 15s as indicated by the bars. **h)** Bar diagram showing maximum currents of Orai1 A137Bpa, Orai1 L174Bpa and Orai1 A254Azi expressed in the STIM1/Orai1 DKO HEK293 cells, without and with STIM1 before and after UV light (15s) (Welch-ANOVA: F(15;31,96)=111,47, p=0). Single data are indicated in grey. Data represent mean values ± SEM of indicated number (n) of experiments. *Significant differences ($p < 0.05$). Detailed statistic values are shown in **Supplementary Figure 3**. Source data are provided as a Source Data file.

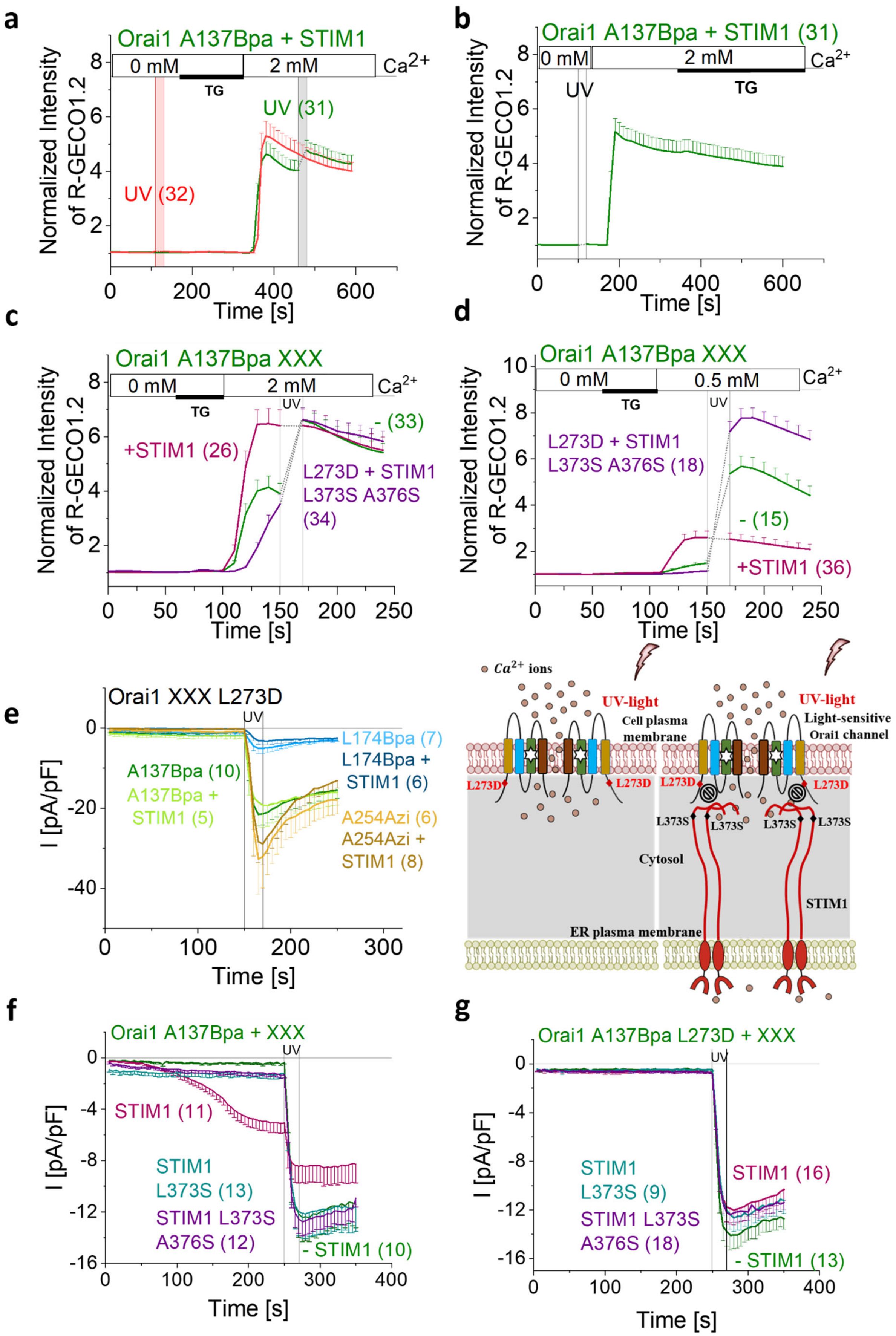
a
Orai1 A137Bpa + STIM1
0 mM
2 mM
Ca2+
TG
UV (31)
UV (32)
Normalized Intensity of R-GECO1.2
Time [s]
b
Orai1 A137Bpa + STIM1 (31)
0 mM
2 mM
Ca2+
UV
TG
Normalized Intensity of R-GECO1.2
Time [s]
c
Orai1 A137Bpa XXX
0 mM
2 mM
Ca2+
TG
UV
- (33)
+STIM1 (26)
L273D + STIM1 L373S A376S (34)
Normalized Intensity of R-GECO1.2
Time [s]
d
Orai1 A137Bpa XXX
0 mM
0.5 mM
Ca2+
TG
UV
L273D + STIM1 L373S A376S (18)
- (15)
+STIM1 (36)
Normalized Intensity of R-GECO1.2
Time [s]
e
Orai1 XXX L273D
UV
L174Bpa (7)
L174Bpa + STIM1 (6)
A137Bpa (10)
A137Bpa + STIM1 (5)
A254Azi (6)
A254Azi + STIM1 (8)
I [pA/pF]
Time [s]
Ca2+ ions
UV-light
Cell plasma membrane
UV-light
Light-sensitive Orai1 channel
L273D
L273D
L273D
L273D
L373S
L373S
L373S
L373S
Cytosol
STIM1
ER plasma membrane
f
Orai1 A137Bpa + XXX
UV
STIM1 (11)
STIM1 L373S (13)
STIM1 L373S A376S (12)
- STIM1 (10)
I [pA/pF]
Time [s]
g
Orai1 A137Bpa L273D + XXX
UV
STIM1 L373S (9)
STIM1 L373S A376S (18)
STIM1 (16)
- STIM1 (13)
I [pA/pF]
Time [s]

Supplementary Figure 7: **UV-mediated activation of Orai1 A137Bpa is sufficient to reach maximum activation of Orai1 A137Bpa**. a) $Ca^{2+}$ imaging measurements of Orai1 A137Bpa co-expressed with STIM1 using R-GECO1.2. Intracellular $Ca^{2+}$ levels, represented by the normalized intensity of R-GECO1.2 of the above mentioned Orai1/STIM1 mutants in HEK293 cells, were monitored initially in 0mM $Ca^{2+}$ solution followed by treatment with 1µM TG and perfusion of a 2mM $Ca^{2+}$ solution. UV light is applied for 10s under 0mM $Ca^{2+}$ conditions either before the application of TG or under 2mM $Ca^{2+}$ solution conditions after application of TG. Due to filter exchange, R-GECO1.2 intensity could not be recorded simultaneously with exposure to UV light. b) Corresponding measurement to (a) while exposure to UV light occurs under 0mM $Ca^{2+}$ solution conditions and application of TG is performed after UV-mediated increase in the $Ca^{2+}$ current under 2mM $Ca^{2+}$ solution condition (t = 375s). c) Corresponding measurements to (a) comparing Orai1 A137Bpa, Orai1 A137Bpa co-expressed with STIM1 and Orai1 A137Bpa L273D co-expressed with STIM1 L373S A376S. UV light is applied for 10s under 2mM Ca2+ solution conditions. Due to filter exchange, R-GECO1.2 intensity could not be recorded simultaneously with exposure to UV light. d) Corresponding measurements to (c) while using a 0.5mM $Ca^{2+}$ solution instead of the usual 2mM $Ca^{2+}$ solution. e) Time courses of current densities after whole-cell break-in of the light-sensitive Orai1 mutants (Orai1 A137Bpa, Orai1 L174Bpa and A254Azi) combined with the L273D mutation in the presence and absence of STIM1. UV light is applied for 15s. Illustration on the right-hand side highlights that the STIM1 and Orai1 mutations inhibiting their coupling, did not affect photocrosslinking-induced Orai1 activation. f) and g) Time courses of current densities after whole-cell break-in of the light-sensitive Orai1 A137Bpa (b) and Orai1 A137Bpa L273D (c) mutants co-expressed with STIM1, STIM1 L373S and STIM1 L373S A376S. UV light is applied for 15s. Data represent mean values ± SEM of indicated number (n) of experiments. Source data are provided as a Source Data file.

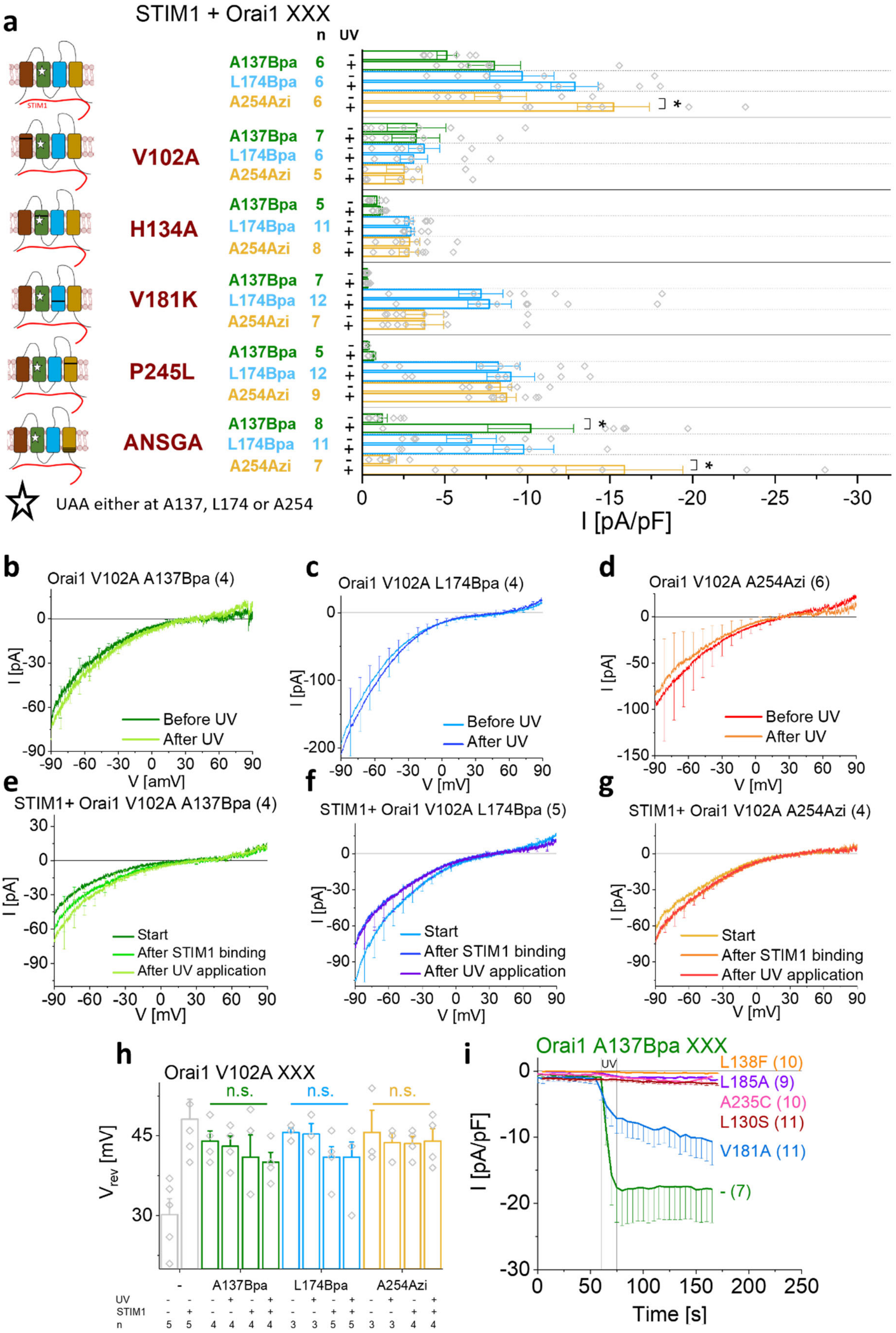
a
STIM1 + Orai1 XXX
n UV
A137Bpa 6
L174Bpa 6
A254Azi 6
V102A
A137Bpa 7
L174Bpa 6
A254Azi 5
H134A
A137Bpa 5
L174Bpa 11
A254Azi 8
V181K
A137Bpa 7
L174Bpa 12
A254Azi 7
P245L
A137Bpa 5
L174Bpa 12
A254Azi 9
ANSGA
A137Bpa 8
L174Bpa 11
A254Azi 7
UAA either at A137, L174 or A254
I [pA/pF]
b
Orai1 V102A A137Bpa (4)
Before UV
After UV
I [pA]
V [amV]
c
Orai1 V102A L174Bpa (4)
Before UV
After UV
I [pA]
V [mV]
d
Orai1 V102A A254Azi (6)
Before UV
After UV
I [pA]
V [mV]
e
STIM1+ Orai1 V102A A137Bpa (4)
Start
After STIM1 binding
After UV application
I [pA]
V [mV]
f
STIM1+ Orai1 V102A L174Bpa (5)
Start
After STIM1 binding
After UV application
I [pA]
V [mV]
g
STIM1+ Orai1 V102A A254Azi (4)
Start
After STIM1 binding
After UV application
I [pA]
V [mV]
h
Orai1 V102A XXX
n.s.
n.s.
n.s.
$V_{rev}$ [mV]
A137Bpa
L174Bpa
A254Azi
UV
STIM1
n
i
Orai1 A137Bpa XXX
UV
L138F (10)
L185A (9)
A235C (10)
L130S (11)
V181A (11)
- (7)
I [pA/pF]
Time [s]

Supplementary Figure 8: **GoF mutations in Orai1 TM domains, but not Orai1 nexus, largely interfere with STIM1-mediated activation of photocrosslinking UAA-containing Orai1 mutants.** Graphical illustration on the left-hand side represent photocrosslinking UAA-containing Orai1 mutants, exemplarily shown for Orai1 A137Bpa (star), combined with one of the following GoF mutations: V102A, H134A, V181K, P245L and $_{261}$ANSGA$_{265}$ (black line) in the presence of STIM1. **a)** Bar diagram summarizes currents measured before (t=235s) and after (t=265s) application of UV light for the above mentioned photocrosslinking UAA- and GoF mutation-containing Orai1 double mutants in the presence of STIM1 (Welch-ANOVA for Orai1 A137Bpa mutants: $F(11;20,08)=13,3$, $p=6,64*10^{-7}$; for Orai1 L174Bpa mutants $F(11;32,26)=9,82$, $p=1,88*10^{-7}$; for Orai1 A254Azi mutants $F(11;25,27)=15.90$, $p=1,18*10^{-8}$)). **b)-g**) I/V relationships of Orai1 V102A A137Bpa (b,e), Orai1 V102A L174Bpa (c,f), and Orai1 V102A A254Azi (d,g) with or without co-expression of STIM1. **h)** Corresponding reversal potential ($V_{rev}$) to b-g) and Orai1 V102A with and without co-expression of STIM1 (one way ANOVA $F(13, 42)=2,77$, $p=0,00619$). **i)** Time courses of current densities after whole-cell break-in of the light-sensitive Orai1 A137Bpa mutant combined with the various GoF mutations: L130S, L138F, V181A, L185A and A235C. UV light is applied for 15s. Data represent mean values ± SEM of indicated number (*n*) of experiments. * Significant differences ($p < 0.05$). Detailed statistic values are shown in **Supplementary Table 3.** Source data are provided as a Source Data file.

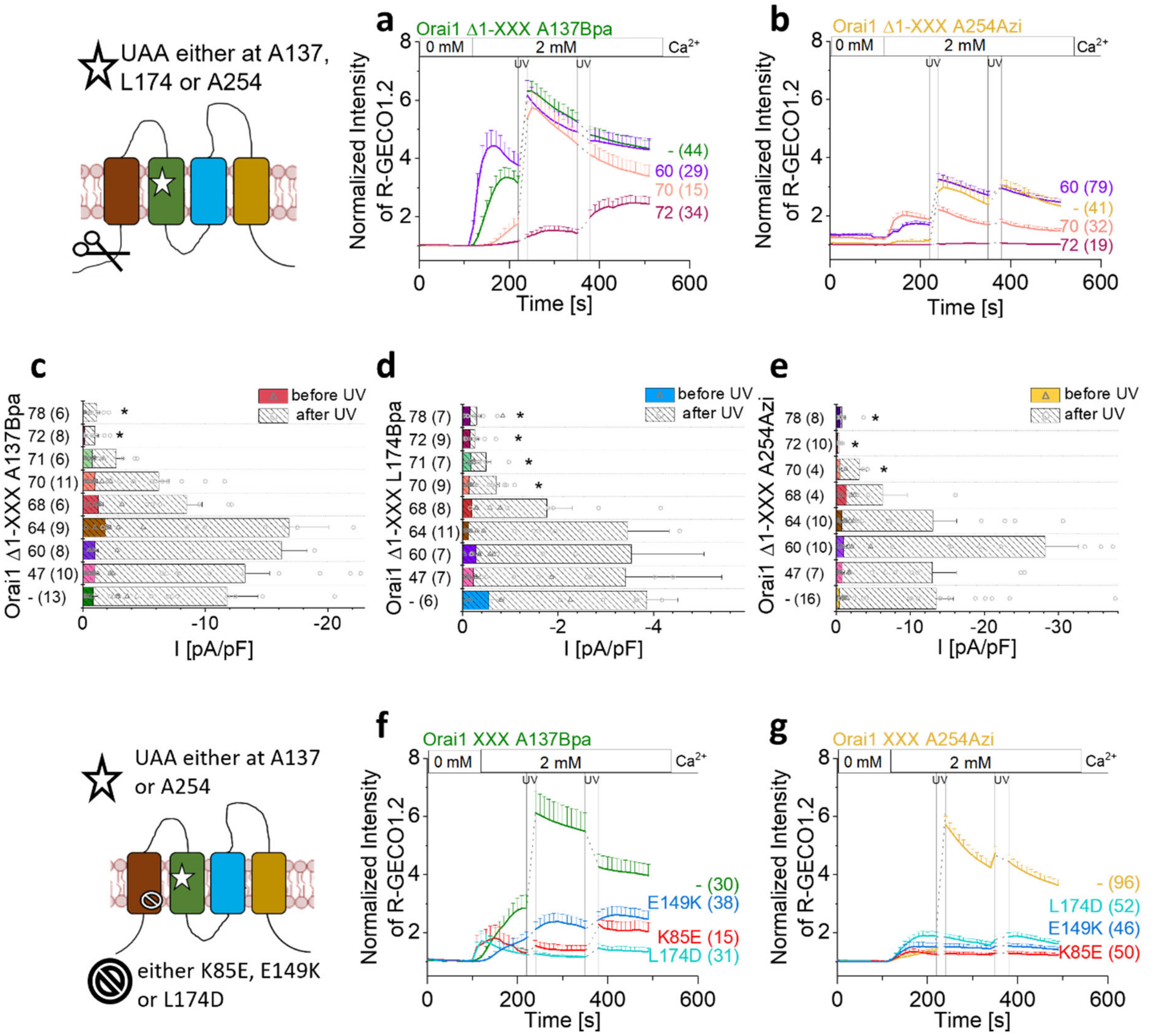


Supplementary Figure 9: **Light-sensitive Orai1 mutants require an intact channel geometry.** Graphical illustrations represent photocrosslinking UAA-containing Orai1 mutants, exemplarily shown for Orai1 A137Bpa, combined with various N-terminal truncations (scissor) or cytosolic extended TM region (CETR) – LoF mutations (crossed circle). **a) and b)** $Ca^{2+}$ imaging measurements of the light-sensitive Orai1 mutants (Orai1 A137Bpa (a), and Orai1 A254Azi (b)) paired with different N-terminal truncations (Δ1-60/70/72) using R-GECO1.2. Normalized intensity plots were initially monitored in 0mM $Ca^{2+}$ solution followed by 2mM $Ca^{2+}$ solution. UV light is applied for 10s and 30s. Due to filter exchange, R-GECO1.2 intensity could not be recorded simultaneously with exposure to UV light. **c)-e)** The bar diagram summarizes maximal currents of the light-sensitive Orai1 mutants (Orai1 A137Bpa (c), Orai1 L174Bpa (d) and Orai1 A254Azi (e)) paired with different N-terminal truncations (Δ1-47/60/64/68/70/71/72/78) before and after application of 15s UV light (Welch-ANOVA before UV: $F(8;25,15)=7,73$, $p=3,49*10^{-5}$ (c); $F(8;22,81)=1.44$, $p=0,23$ (d); $F(7;16,86)=2,55$, $p=0,05$ (e); after UV: $F(8;26,08)=19,43$, $p=3,92*10^{-9}$ (c); $F(8;24,84)=8,94$, $p=1,03*10^{-5}$ (d); $F(7;16,83)=12.88$, $p=1,09*10^{-5}$ (e)). **f) and g)** $Ca^{2+}$ imaging measurements of Orai1 A137Bpa (f) and Orai1 A254Azi (g) paired with CETR-LoF mutations (K85E/E149K/L174D) using R-GECO1.2. Normalized intensity plots were initially monitored in 0mM $Ca^{2+}$ solution followed by 2mM $Ca^{2+}$ solution. UV light is applied for 10s and 30s. Due to filter exchange, R-GECO1.2 intensity could not be recorded simultaneously with exposure to UV light. Data represent mean

values ± SEM of indicated number (*n*) of experiments. *Significant differences ($p < 0.05$). Detailed statistic values are shown in **Supplementary Table 3**. Source data are provided as a Source Data file.

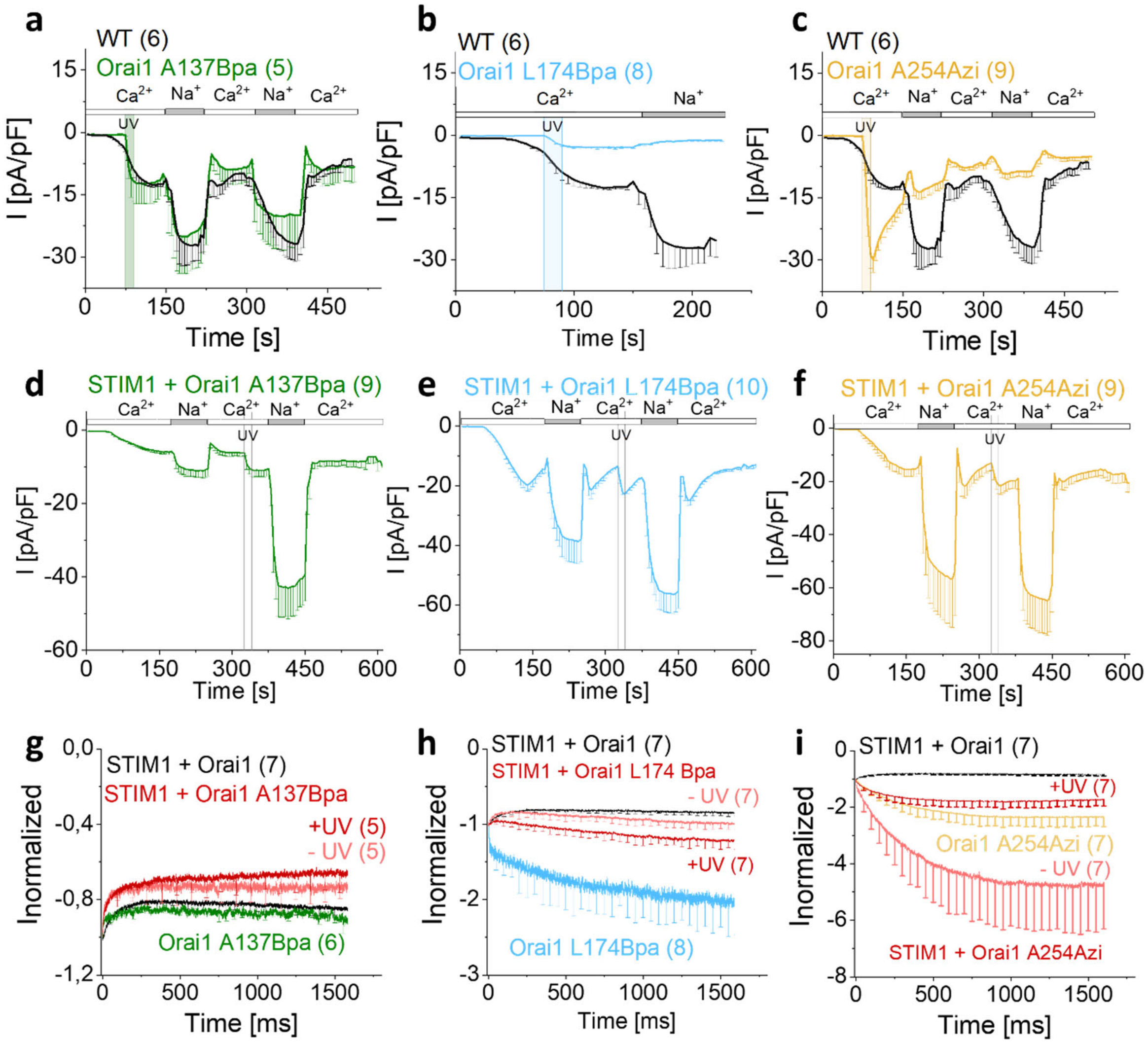


Supplementary Figure 10: **UV light-activated Orai1 A137Bpa currents match CRAC channel hallmarks best. a)-c)** Time course showing normalized currents of Orai1 A137Bpa (a), Orai1 L174Bpa (b) and Orai1 A254Azi (c) in the absence of STIM1 as well as with and without exposure to UV light compared to wild-type STIM1/Orai1 upon application of a voltage step to -70mV from a holding potential of 0mV. **d)-f)** Time course of current densities after whole-cell break-in comparing Orai1 A137Bpa (d), Orai1 L174Bpa (e) and Orai1 A254Azi (f) in the presence of STIM1. Currents were initially monitored in 10mM $Ca^{2+}$ solution which was then exchanged by a DVF $Na^{+}$-containing solution in a reversible manner (except (e)). 15s UV light illumination was only applicable for the light-sensitive Orai1 mutants. **g)-i)** Time course showing normalized currents of the three light-sensitive Orai1 mutants in the absence as well as the presence of STIM1 (+/- UV light) compared to wild-type CRAC channel (STIM1 + Orai1; WT) obtained upon application of a voltage step to -70mV from a holding potential of 0mV using 20mM EGTA in the internal pipette solution. Data represent mean values ± SEM of indicated number (*n*) of experiments. Source data are provided as a Source Data file.

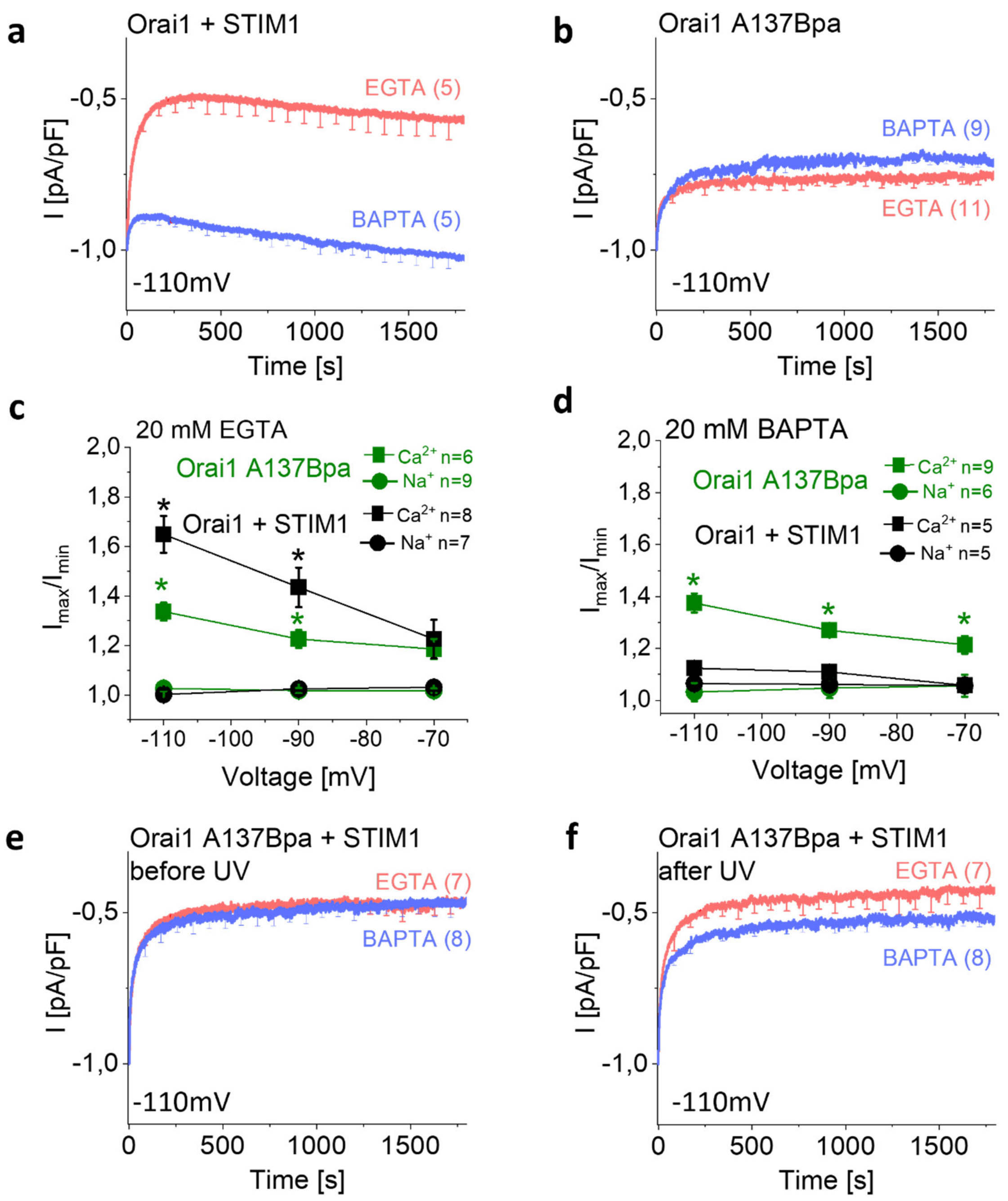


Supplementary Figure 11: **Orai1 A137Bpa displays $Ca^{2+}$ dependent inactivation, however, to distinct extents than a wild-type CRAC channel a) and b)** Time course showing normalized currents of a wild-type CRAC channel (STIM1 + Orai1) (a) or Orai1 A137Bpa (b) obtained upon application of a voltage step to -110mV from a holding potential of 0mV while using 20mM EGTA or 20mM BAPTA as $Ca^{2+}$-chelator within the internal pipette solution. **c) and d)** Ratio of maximum ($I_{max}$ at t=0s) versus minimum ($I_{min}$ at t=250s) currents obtained upon application of voltage steps to -110mV, -90mV or -70mV from a holding potential of 0mV using 20mM EGTA (c) or 20mM BAPTA (d) in the internal pipette solution. (One-way ANOVA/Welch-ANOVA for EGTA: $F(3, 26)=5{,}61$ , $p=0{,}0042$ (-70mV); $F(3, 11{,}38)=16{,}6$ , $p=02{,}14*10^{-4}$ (-90mV) and $F(3, 29)=50{,}6$ , $p<0{,}0001$ (-110mV); one-way ANOVA for BAPTA: : $F(3, 21)=6{,}3$ , $p=0{,}0032$ (-70mV); $F(3, 21)=13{,}95$ , $p<0{,}0001$ (-90mV) and $F(3, 21)=23{,}71$ , $p<0{,}0001$ (-110mV)) **e) and f)** Corresponding time course to (b) co-expressing Orai1 A137Bpa and STIM1

before versus after passive store-depletion, (e), before and after (f) store-depletion and subsequent application of UV light. Data represent mean values ± SEM of indicated number (*n*) of experiments. *Significant differences ($p < 0.05$). Detailed statistic values are shown in **Supplementary Table 3**. Source data are provided as a Source Data file.

Supplementary Table 1: **Fast and slow decay times of FCDI or reactivation of Orai channels.** Table indicates $\tau_{fast}$ and $\tau_{slow}$ for FCDI of STIM1/Orai1 and Orai1 A137Bpa currents and $\tau_{fast}$ for Orai1 A254Azi currents showing only reactivation. Decay times for FCDI of STIM1/Orai1 and Orai1 A137Bpa ± STIM1 currents were determined using EGTA and BAPTA. Decay times for FCDI and/or reactivation were determined for Orai1 L174Bpa ± STIM1 and Orai1 A254Azi ± STIM1 currents using EGTA.

| | | | | EGTA | | BAPTA | |
|---|---|---|---|---|---|---|---|
| STIM1 | UV | mV | | $\tau_{slow}$ [ms] | $\tau_{fast}$ [ms] | $\tau_{slow}$ [ms] | $\tau_{fast}$ [ms] |
| + | - | -70 | Orai | 16,82 ± 1,33 | 86,42 ± 1,03 | 1,91 ± 0,43 | 18,98 ± 0,70 |
| | | -90 | | 10,55 ± 0,29 | 62,00 ± 0,57 | 5,25 ± 0,39 | 32,92 ± 0,90 |
| | | -110 | | 11,04 ± 0,15 | 68,13 ± 0,31 | 2,01 ± 0,36 | 22,45 ± 1,20 |
| - | + | -70 | A137Bpa | 3,75 ± 0,30 | 242,92 ± 31,75 | 45,28 ±2,52 | 228,95 ± 8,49 |
| | | -90 | | 6,81 ± 0,17 | 144,88 ± 8,79 | 39,26 ± 2,18 | 292,77 ± 7,04 |
| | | -110 | | 8,62 ± 0,19 | 128,03 ± 1,78 | 42,49 ± 1,58 | 350,50 ± 9,98 |
| + | - | -70 | A137Bpa | 10,17 ± 0,43 | 91,46 ± 2,33 | 10,37 ± 0,29 | 171,31 ± 2,95 |
| | | -90 | | 17,07 ± 0,20 | 133,31 ± 1,13 | 16,69 ± 0,18 | 236,88 ± 1,26 |
| | | -110 | | 30,17 ± 0,19 | 343,52 ± 2,12 | 12,83 ± 0,15 | 131,84 ± 1,32 |
| + | + | -70 | A137Bpa | 22,90 ± 0,88 | 163,38 ± 6,21 | 14,50 ± 0,35 | 243,26 ± 3,63 |
| | | -90 | | 17,11 ± 0,38 | 166,30 ± 6,27 | 22,25 ± 0,26 | 386,72 ± 2,64 |
| | | -110 | | 41,73 ± 0,19 | 566,00 ± 4,62 | 27,16 ± 0,22 | 373,47 ± 2,21 |
| - | + | -70 | L174Bpa | 7,07 ± 0,29 | 702,20 ± 4,46 | n.d. | n.d. |
| + | - | -70 | L174Bpa | 32,25 ± 0,34 | 208,10 ± 2,55 | n.d. | n.d. |
| + | + | -70 | L174Bpa | 19,13 ± 0,95 | 1133,72 ± 6,64 | n.d. | n.d. |
| - | + | -70 | A254Azi | 294,11 ± 0,33 | | n.d. | n.d. |
| + | - | -70 | A254Azi | 339,40 ± 0,36 | | n.d. | n.d. |
| + | + | -70 | A254Azi | 186,93 ± 0,31 | | n.d. | n.d. |

Supplementary Table 2: **List of used primers** ( F indicates forward primer, R indicates reverse primer).

| | Orai1 mutants | Primer | Primer sequence (5' - 3') |
|---|---|---|---|
| cloning | Orai1 Δ 1 - 47 | F | tatata gaattc atg tcc gcc gtc acc tac ccg |
| | | R | tatata ccgcgg ggc ata gtg gct gcc ggg |
| | Orai1 Δ 1 - 60 | F | tatata gaattc atg tcc gag gtg atg agc ctc aac gag cac |
| | | R | tatata ccgcgg ggc ata gtg gct gcc ggg |
| | Orai1 Δ 1 - 64 | F | tatata gaattc atg agc ctc aac gag cac tcc atg |
| | | R | tatata ccgcgg ggc ata gtg gct gcc ggg |
| | Orai1 Δ 1 - 68 | F | tatata gaattc atg cac tcc atg cag gcg ctg |
| | | R | tatata ccgcgg ggc ata gtg gct gcc ggg |
| | Orai1 Δ 1 - 70 | F | tatata gaattc atg atg cag gcg ctg tcc tgg |
| | | R | tatata ccgcgg ggc ata gtg gct gcc ggg |
| | Orai1 Δ 1 - 71 | F | tatata gaattc atg cag gcg ctg tcc tgg |
| | | R | tatata ccgcgg ggc ata gtg gct gcc ggg |
| | Orai1 Δ 1 - 72 | F | tatata gaattc atg gcg ctg tcc tgg cgc aag |
| | | R | tatata ccgcgg ggc ata gtg gct gcc ggg |
| | Orai1 Δ 1 - 78 | F | tatata gaattc atg ctc tac ttg agc cgc gcc |
| | | R | tatata ccgcgg ggc ata gtg gct gcc ggg |
| TM1 | K85E | F | acttgagccgcgccgagcttaaagcctcc |
| | | R | ggaggctttaagctcggcgcggctcaagt |
| | V102A | F | ggcttcgccatggcggcaatggtggag |
| | | R | ctccaccattgccgccatggcgaagcc |
| | E106Q | F | ggtggcaatggtgcaggtgcagctgga |
| | | R | tccagctgcacctgcaccattgccacc |
| TM2 | L130S | F | cctgcaccacagtgtcggtggctgtgcacc |
| | | R | ggtgcacagccaccgacactgtggtgcagg |
| | L130* | F | cctgcaccacagtgtaggtggctgtgcacc |
| | | R | ggtgcacagccacctacactgtggtgcagg |
| | H134A | F | gtgctggtggctgtggccctgtttgcgctcat |
| | | R | atgagcgcaaacagggccacagccaccagcac |
| | H134A A137* | F | cagtgctggtggctgtggccctgttttagctcatgatcagcacct |
| | | R | aggtgctgatcatgagctaaaacagggccacagccaccagcactg |
| | H134* | F | gtgctggtggctgtgtagctgtttgcgctcatg |
| | | R | catgagcgcaaacagctacacagccaccagcac |
| | F136* | F | atcatgagcgcctacaggtgcacagccaccagca |
| | | R | tgctggtggctgtgcacctgtaggcgctcatgat |
| | A137* | F | gctggtggctgtgcacctgttttagctcatgatcag |
| | | R | ctgatcatgagctaaaacaggtgcacagccaccagc |
| | A137* L138F | F | gctggtggctgtgcacctgttttagttcatgatcagcacctg |
| | | R | caggtgctgatcatgaactaaaacaggtgcacagccaccagc |
| | L138F | F | tgtgcacctgtttgcgttcatgatcagcacctg |
| | | R | caggtgctgatcatgaacgcaaacaggtgcaca |
| | L138* | F | gctgtgcacctgtttgcgtagatgatcagcacctgcatc |
| | | R | gatgcaggtgctgatcatctacgcaaacaggtgcacagc |
| | E149K | F | atcctgcccaacatcaaggcggtgagcaac |
| | | R | gttgctcaccgccttgatgttgggcaggat |

| | | | |
|---|---|---|---|
| TM3 | L174D | F | caccgccacatcgaggatgcctgggccttctcc |
| | | R | ggagaaggcccaggcatcctcgatgtggcggtg |
| | L174* | F | ccgccacatcgagtaggcctgggccttc |
| | | R | gaaggcccaggcctactcgatgtggcgg |
| | W176* | F | cacatcgagctggcctaggccttctcca |
| | | R | tggagaaggcctaggccagctcgatgtg |
| | S179* | F | gctggcctgggccttctagaccgtcatcg |
| | | R | cgatgacggtctagaaggcccaggccagc |
| | V181A | F | gccttctccaccgccatcggcacgctg |
| | | R | cagcgtgccgatggcggtggagaaggc |
| | V181K | F | tgggccttctccaccaagatcggcacgctgctc |
| | | R | gagcagcgtgccgatcttggtggagaaggccca |
| | V181* | F | tgggccttctccacctagatcggcacgctgctc |
| | | R | gagcagcgtgccgatctaggtggagaaggccca |
| | L185A | F | ccgtcatcggcacggcgctcttcctagctg |
| | | R | cagctaggaagagcgccgtgccgatgacgg |
| | L185* | F | caccgtcatcggcacgtagctcttcctagctgag |
| | | R | ctcagctaggaagagctacgtgccgatgacggtg |
| | F187* | F | cggcacgctgctctagctagctgaggtggt |
| | | R | accacctcagctagctagagcagcgtgccg |
| | L188* | F | cggcacgctgctcttctaggctgaggtggtgctgc |
| | | R | gcagcaccacctcagcctagaagagcagcgtgccg |
| | V191* | F | gctcttcctagctgagtaggtgctgctctgctgg |
| | | R | ccagcagagcagcacctactcagctaggaagagc |
| | L194* | F | ctagctgaggtggtgctgtagtgctgggtcaagttcttg |
| | | R | caagaacttgacccagcactacagcaccacctcagctag |
| TM4 | A235C | F | accccgggccaggcatgtgccatcgcc |
| | | R | ggcgatggcacatgcctggcccggggt |
| | A235* | F | caccccgggccaggcataggccatcgcctcg |
| | | R | cgaggcgatggcctatgcctggcccggggtg |
| | S239* | F | agctgccatcgcctagaccaccatcatgg |
| | | R | ccatgatggtggtctaggcgatggcagct |
| | M243* | F | cctcgaccaccatctaggtgcccttcggcc |
| | | R | ggccgaagggcacctagatggtggtcgagg |
| | P245L | F | ccaccatcatggtgctcttcggcctgatctt |
| | | R | aagatcaggccgaagagcaccatgatggtgg |
| | P245* | F | ctcgaccaccatcatggtgtagttcggcctgatctttatcg |
| | | R | cgataaagatcaggccgaactacaccatgatggtggtcgag |
| | F250* | F | cccttcggcctgatctagatcgtcttcgccgtcc |
| | | R | ggacggcgaagacgatctagatcaggccgaaggg |
| | A254* | F | gcctgatctttatcgtcttctaggtccacttctaccgctcact |
| | | R | agtgagcggtagaagtggacctagaagacgataaagatcaggc |
| | ANSGA | F | tcgccgtccacttctaccgctcagcgaatagcggtgcgactgaccgacagttccaggagc |
| | | R | gctcctggaactgtcggtcagtcgcaccgctattcgctgagcggtagaagtggacggcga |
| C-ter | L273D | F | gacagttccaggaggacaacgagctggcgg |
| | | R | ccgccagctcgttgtcctcctggaactgtc |
| | STIM1 mutants | Primer | Primer sequence (5' - 3') |
| STIM1 | L373S | F | aaaatgctgagaagcagtcgctggtggccaaggagg |
| | | R | cctccttggccaccagcgactgcttctcagcatttt |

| | | | |
|---|---|---|---|
| | L373S A376S | F | aaatgctgagaagcagtcgctggtgagcaaggagggggctgag |
| | | R | ctcagccccctccttgctcaccagcgactgcttctcagcattt |

Supplementary Table 3: **Statistical analysis** of significant differences ($p<0.05$) of data sets in their respective graphs and statistical test (0 indicates not significant, 1 indicates significant).

| Figure | test | p value | F statistics (only for ANOVA) | Post-hoc (in case of ANOVA) | Probability | Significance p<0,05 |
|---|---|---|---|---|---|---|
| 2g-i | Mann-Whitney | 0,24026 | | WT - A137Bpa | | |
| | | 0,24026 | | WT - L174Bpa | | |
| | | 0,93723 | | WT - A254Azi | | |
| 3d | one-way ANOVA | 0,47227 | F(2, 15)=0,789 | WT - A137Bpa + S1 | 0,26458 | 0 |
| | | | | A137Bpa + S1 A137Bpa + S1 +UV | 0,33358 | 0 |
| | | | | A137Bpa + S1 +UV WT | 0,87512 | 0 |
| 3d | one-way ANOVA | 0,95636 | F(2, 15)=0,045 | WT L174Bpa + S1 | 0,78651 | 0 |
| | | | | A137Bpa + S1 L174Bpa + S1 +UV | 0,81483 | 0 |
| | | | | L174Bpa + S1 +UV WT | 0,97067 | 0 |
| 3d | one-way ANOVA | 0,84277 | F(2, 15)=0,173 | WT A254Azi + S1 | 0,61785 | 0 |
| | | | | A254Azi + S1 +UV WT | 1 | 0 |
| | | | | A137Bpa + S1 A254Azi + S1 +UV | 0,61785 | 0 |
| 3m/4d | Welch-ANOVA | 1,53E-13 | F(7, 20.37)=86,17 | STIM1+Orai1-UV STIM1+Orai1 +UV | 1 | 0 |
| | | | | STIM1+Orai1-A137bpa+UV STIM1+Orai1 +UV | 0,88233 | 0 |
| | | | | STIM1+Orai1-A137bpa+UV STIM1+Orai1-UV | 0,95125 | 0 |
| | | | | STIM1+Orai1-A137bpa-UV STIM1+Orai1 +UV | 0,99851 | 0 |
| | | | | STIM1+Orai1-A137bpa-UV STIM1+Orai1-UV | 0,99972 | 0 |
| | | | | STIM1+Orai1-A137bpa-UV STIM1+Orai1-A137bpa+UV | 0,9996 | 0 |
| | | | | STIM1+Orai1-A137bpa-L273D+UV STIM1+Orai1 +UV | 1,14E-04 | 1 |
| | | | | STIM1+Orai1-A137bpa-L273D+UV STIM1+Orai1-UV | 9,86E-07 | 1 |
| | | | | STIM1+Orai1-A137bpa-L273D+UV STIM1+Orai1-A137bpa+UV | 1,04E-06 | 1 |
| | | | | STIM1+Orai1-A137bpa-L273D+UV STIM1+Orai1-A137bpa-UV | 1,05E-04 | 1 |
| | | | | STIM1+Orai1-A137bpa-L273D-UV STIM1+Orai1 +UV | 1,99E-04 | 1 |
| | | | | STIM1+Orai1-A137bpa-L273D-UV STIM1+Orai1-UV | 1,65E-06 | 1 |
| | | | | STIM1+Orai1-A137bpa-L273D-UV STIM1+Orai1-A137bpa+UV | 3,50E-06 | 1 |
| | | | | STIM1+Orai1-A137bpa-L273D-UV STIM1+Orai1-A137bpa-UV | 1,38E-04 | 1 |
| | | | | STIM1+Orai1-A137bpa-L273D-UV STIM1+Orai1-A137bpa-L273D+UV | 0,99994 | 0 |
| | | | | STIM1-L373S-A376S+Orai1-A137bpa-L273D+UV STIM1+Orai1 +UV | 8,83E-05 | 1 |
| | | | | STIM1-L373S-A376S+Orai1-A137bpa-L273D+UV STIM1+Orai1-UV | 1,86E-05 | 1 |
| | | | | STIM1-L373S-A376S+Orai1-A137bpa-L273D+UV STIM1+Orai1-A137bpa+UV | 9,83E-05 | 1 |
| | | | | STIM1-L373S-A376S+Orai1-A137bpa-L273D+UV STIM1+Orai1-A137bpa-UV | 2,22E-04 | 1 |
| | | | | STIM1-L373S-A376S+Orai1-A137bpa-L273D+UV STIM1+Orai1-A137bpa-L273D+UV | 0,528 | 0 |
| | | | | STIM1-L373S-A376S+Orai1-A137bpa-L273D+UV STIM1+Orai1-A137bpa-L273D-UV | 0,45299 | 0 |
| | | | | STIM1-L373S-A376S+Orai1-A137bpa-L273D-UV STIM1+Orai1 +UV | 4,99E-05 | 1 |
| | | | | STIM1-L373S-A376S+Orai1-A137bpa-L273D-UV STIM1+Orai1-UV | 6,21E-07 | 1 |
| | | | | STIM1-L373S-A376S+Orai1-A137bpa-L273D-UV STIM1+Orai1-A137bpa+UV | 5,05E-07 | 1 |
| | | | | STIM1-L373S-A376S+Orai1-A137bpa-L273D-UV STIM1+Orai1-A137bpa-UV | 7,92E-05 | 1 |
| | | | | STIM1-L373S-A376S+Orai1-A137bpa-L273D-UV STIM1+Orai1-A137bpa-L273D+UV | 0,97644 | 0 |
| | | | | STIM1-L373S-A376S+Orai1-A137bpa-L273D-UV STIM1+Orai1-A137bpa-L273D-UV | 0,88764 | 0 |
| | | | | STIM1-L373S-A376S+Orai1-A137bpa-L273D-UV STIM1-L373S-A376S+Orai1-A137bpa-L273D+UV | 0,8196 | 0 |
| 4b | one-way ANOVA | 0,04571 | F(7, 99)=2,15 | A137Bpa L273D -STIM A137Bpa - STIM | 0,9929 | 0 |
| | | | | A137Bpa + STIM A137Bpa - STIM | 0,0019 | 1 |
| | | | | A137Bpa + STIM A137Bpa L273D -STIM | 7,15E-04 | 1 |
| | | | | A137Bpa L273D +STIM A137Bpa - STIM | 0,21698 | 0 |
| | | | | A137Bpa L273D +STIM A137Bpa L273D -STIM | 0,16561 | 0 |
| | | | | A137Bpa L273D +STIM A137Bpa + STIM | 0,02043 | 1 |
| | | | | A137Bpa + STIM1 L373S A137Bpa - STIM | 0,28175 | 0 |
| | | | | A137Bpa + STIM1 L373S A137Bpa L273D -STIM | 0,23232 | 0 |
| | | | | A137Bpa + STIM1 L373S A137Bpa + STIM | 0,02279 | 1 |
| | | | | A137Bpa + STIM1 L373S A137Bpa L273D +STIM | 0,91572 | 0 |
| | | | | A137Bpa L273D+ STIM1 L373S A137Bpa - STIM | 0,40158 | 0 |
| | | | | A137Bpa L273D+ STIM1 L373S A137Bpa L273D -STIM | 0,35737 | 0 |
| | | | | A137Bpa L273D+ STIM1 L373S A137Bpa + STIM | 0,02185 | 1 |
| | | | | A137Bpa L273D+ STIM1 L373S A137Bpa L273D +STIM | 0,77267 | 0 |
| | | | | A137Bpa L273D+ STIM1 L373S A137Bpa + STIM1 L373S | 0,85608 | 0 |
| | | | | A137Bpa + STIM1 L373S L376S A137Bpa - STIM | 0,35543 | 0 |
| | | | | A137Bpa + STIM1 L373S L376S A137Bpa L273D -STIM | 0,30261 | 0 |
| | | | | A137Bpa + STIM1 L373S L376S A137Bpa + STIM | 0,01293 | 1 |
| | | | | A137Bpa + STIM1 L373S L376S A137Bpa L273D +STIM | 0,75832 | 0 |
| | | | | A137Bpa + STIM1 L373S L376S A137Bpa + STIM1 L373S | 0,85237 | 0 |
| | | | | A137Bpa + STIM1 L373S L376S A137Bpa L273D+ STIM1 L373S | 0,98992 | 0 |
| | | | | A137Bpa L273D+ STIM1 L373S L376S A137Bpa - STIM | 0,33024 | 0 |
| | | | | A137Bpa L273D+ STIM1 L373S L376S A137Bpa L273D -STIM | 0,27054 | 0 |
| | | | | A137Bpa L273D+ STIM1 L373S L376S A137Bpa + STIM | 0,00799 | 1 |
| | | | | A137Bpa L273D+ STIM1 L373S L376S A137Bpa L273D +STIM | 0,73123 | 0 |
| | | | | A137Bpa L273D+ STIM1 L373S L376S A137Bpa + STIM1 L373S | 0,83498 | 0 |
| | | | | A137Bpa L273D+ STIM1 L373S L376S A137Bpa L273D+ STIM1 L373S | 0,99543 | 0 |
| | | | | A137Bpa L273D+ STIM1 L373S L376S A137Bpa + STIM1 L373S L376S | 0,99311 | 0 |
| 5b | Welch-ANOVA | 6,96E-12 | F(9, 160.73)=9.79 | Orai1 END Orai1 MAX | 2,70E-04 | 1 |
| | | | | A137Bpa MAX Orai1 MAX | 1 | 0 |
| | | | | A137Bpa MAX Orai1 END | 1,38E-05 | 1 |
| | | | | A137Bpa END Orai1 MAX | 0,58513 | 0 |
| | | | | A137Bpa END Orai1 END | 0,03568 | 1 |
| | | | | A137Bpa END A137Bpa MAX | 0,39371 | 0 |
| | | | | A137Bpa+STIM1-UV MAX Orai1 MAX | 1 | 0 |
| | | | | A137Bpa+STIM1-UV MAX Orai1 END | 4,36E-06 | 1 |
| | | | | A137Bpa+STIM1-UV MAX A137Bpa MAX | 1 | 0 |
| | | | | A137Bpa+STIM1-UV MAX A137Bpa END | 0,30519 | 0 |
| | | | | A137Bpa+STIM1-UV END Orai1 MAX | 0,00826 | 1 |
| | | | | A137Bpa+STIM1-UV END Orai1 END | 0,87398 | 0 |
| | | | | A137Bpa+STIM1-UV END A137Bpa MAX | 0,00107 | 1 |
| | | | | A137Bpa+STIM1-UV END A137Bpa END | 0,53433 | 0 |
| | | | | A137Bpa+STIM1-UV END A137Bpa+STIM1-UV MAX | 4,32E-04 | 1 |
| | | | | A137Bpa+STIM1+UV MAX Orai1 MAX | 0,99998 | 0 |
| | | | | A137Bpa+STIM1+UV MAX Orai1 END | 1,77E-04 | 1 |
| | | | | A137Bpa+STIM1+UV MAX A137Bpa MAX | 0,99982 | 0 |
| | | | | A137Bpa+STIM1+UV MAX A137Bpa END | 0,80217 | 0 |
| | | | | A137Bpa+STIM1+UV MAX A137Bpa+STIM1-UV MAX | 0,99938 | 0 |
| | | | | A137Bpa+STIM1+UV MAX A137Bpa+STIM1-UV END | 0,0102 | 1 |
| | | | | A137Bpa+STIM1+UV END Orai1 MAX | 0,81927 | 0 |
| | | | | A137Bpa+STIM1+UV END Orai1 END | 2,58E-04 | 1 |
| | | | | A137Bpa+STIM1+UV END A137Bpa MAX | 0,64015 | 0 |
| | | | | A137Bpa+STIM1+UV END A137Bpa END | 0,9995 | 0 |
| | | | | A137Bpa+STIM1+UV END A137Bpa+STIM1-UV MAX | 0,52853 | 0 |
| | | | | A137Bpa+STIM1+UV END A137Bpa+STIM1-UV END | 0,04124 | 1 |
| | | | | A137Bpa+STIM1+UV END A137Bpa+STIM1+UV MAX | 0,96293 | 0 |
| | | | | wo CCH MAX Orai1 MAX | 1 | 0 |
| | | | | wo CCH MAX Orai1 END | 1,77E-06 | 1 |
| | | | | wo CCH MAX A137Bpa MAX | 1 | 0 |
| | | | | wo CCH MAX A137Bpa END | 0,24842 | 0 |
| | | | | wo CCH MAX A137Bpa+STIM1-UV MAX | 1 | 0 |
| | | | | wo CCH MAX A137Bpa+STIM1-UV END | 2,01E-04 | 1 |
| | | | | wo CCH MAX A137Bpa+STIM1+UV MAX | 0,99893 | 0 |
| | | | | wo CCH MAX A137Bpa+STIM1+UV END | 0,44686 | 0 |

| | | | | | | |
|---|---|---|---|---|---|---|
| | | | | wo CCH END Orai1 MAX | 0,62575 | 0 |
| | | | | wo CCH END Orai1 END | 0,00457 | 1 |
| | | | | wo CCH END A137Bpa MAX | 0,41442 | 0 |
| | | | | wo CCH END A137Bpa END | 1 | 0 |
| | | | | wo CCH END A137Bpa+STIM1-UV MAX | 0,31481 | 0 |
| | | | | wo CCH END A137Bpa+STIM1-UV END | 0,22747 | 0 |
| | | | | wo CCH END A137Bpa+STIM1+UV MAX | 0,84174 | 0 |
| | | | | wo CCH END A137Bpa+STIM1+UV END | 0,99996 | 0 |
| | | | | wo CCH END wo CCH MAX | 0,24959 | 0 |
| 5d | Mann-Whitney test | 3,50E-01 | | WT START 137 START | | |
| | | 6,60E-01 | | WT MAX 137 MAX | | |
| | | 8,84E-01 | | WT END 137 END | | |
| 6a | Mann-Whitney test | 0,00507 | | A137Bpa after UV-light - A137Bpa before UV-light | | |
| | | 0,00507 | | L174Bpa after UV-light - L174Bpa before UV-light | | |
| | | 1,48E-07 | | A254Azi after UV-light - A254Azi before UV-light | | |
| | | 9,39E-04 | | A137Bpa P245L after UV-light - A137Bpa P245L before UV-light | | |
| | | 0,00148 | | L174Bpa P245L after UV-light - L174Bpa P245L before UV-light | | |
| | | 0,02145 | | A254Azi P245L after UV-light - A254Azi P245L before UV-light | | |
| | | 0,03038 | | A137Bpa ANSGA after UV-light - A137Bpa ANSGA before UV-light | | |
| | | 0,00507 | | L174Bpa ANSGA after UV-light - L174Bpa ANSGA before UV-light | | |
| | | 0,00507 | | A254Azi ANSGA after UV-light -A254Azi ANSGA before UV-light | | |
| 6a A137Bpa | Welch-ANOVA | 7,50E-05 | F(11, 21.41)=7 | + - | 0,02591 | 1 |
| | | | | V102A - - | 0,32475 | 0 |
| | | | | V102A - + | 0,05979 | 0 |
| | | | | V102A + - | 0,26925 | 0 |
| | | | | V102A + + | 0,09996 | 0 |
| | | | | V102A + V102A - | 0,9994 | 0 |
| | | | | H134A - - | 1 | 0 |
| | | | | H134A - + | 0,02656 | 1 |
| | | | | H134A - V102A - | 0,34887 | 0 |
| | | | | H134A - V102A + | 0,28448 | 0 |
| | | | | H134A + - | 0,99695 | 0 |
| | | | | H134A + + | 0,02747 | 1 |
| | | | | H134A + V102A - | 0,41034 | 0 |
| | | | | H134A + V102A + | 0,31995 | 0 |
| | | | | H134A + H134A - | 0,99993 | 0 |
| | | | | V181K - - | 1 | 0 |
| | | | | V181K - + | 0,02602 | 1 |
| | | | | V181K - V102A - | 0,33606 | 0 |
| | | | | V181K - V102A + | 0,27575 | 0 |
| | | | | V181K - H134A - | 1 | 0 |
| | | | | V181K - H134A + | 0,99962 | 0 |
| | | | | V181K + - | 0,99681 | 0 |
| | | | | V181K + + | 0,02754 | 1 |
| | | | | V181K + V102A - | 0,40371 | 0 |
| | | | | V181K + V102A + | 0,31683 | 0 |
| | | | | V181K + H134A - | 0,99994 | 0 |
| | | | | V181K + H134A + | 1 | 0 |
| | | | | V181K + V181K - | 0,99965 | 0 |
| | | | | P245L - - | 1 | 0 |
| | | | | P245L - + | 0,02565 | 1 |
| | | | | P245L - V102A - | 0,31297 | 0 |
| | | | | P245L - V102A + | 0,26195 | 0 |
| | | | | P245L - H134A - | 0,99982 | 0 |
| | | | | P245L - H134A + | 0,98679 | 0 |
| | | | | P245L - V181K - | 1 | 0 |
| | | | | P245L - V181K + | 0,98492 | 0 |
| | | | | P245L + - | 0,0096 | 1 |
| | | | | P245L + + | 0,05484 | 0 |
| | | | | P245L + V102A - | 0,99999 | 0 |
| | | | | P245L + V102A + | 0,96496 | 0 |
| | | | | P245L + H134A - | 0,01198 | 1 |
| | | | | P245L + H134A + | 0,02246 | 1 |
| | | | | P245L + V181K - | 0,00978 | 1 |
| | | | | P245L + V181K + | 0,01875 | 1 |
| | | | | P245L + P245L - | 0,00889 | 1 |
| | | | | ANSGA - - | 0,71943 | 0 |
| | | | | ANSGA - + | 0,03546 | 1 |
| | | | | ANSGA - V102A - | 0,9655 | 0 |
| | | | | ANSGA - V102A + | 0,76557 | 0 |
| | | | | ANSGA - H134A - | 0,76599 | 0 |
| | | | | ANSGA - H134A + | 0,87293 | 0 |
| | | | | ANSGA - V181K - | 0,74652 | 0 |
| | | | | ANSGA - V181K + | 0,86166 | 0 |
| | | | | ANSGA - P245L - | 0,69345 | 0 |
| | | | | ANSGA - P245L + | 0,95509 | 0 |
| | | | | ANSGA + - | 0,45652 | 0 |
| | | | | ANSGA + + | 0,99993 | 0 |
| | | | | ANSGA + V102A - | 0,60349 | 0 |
| | | | | ANSGA + V102A + | 0,67037 | 0 |
| | | | | ANSGA + H134A - | 0,46029 | 0 |
| | | | | ANSGA + H134A + | 0,46795 | 0 |
| | | | | ANSGA + V181K - | 0,45808 | 0 |
| | | | | ANSGA + V181K + | 0,46745 | 0 |
| | | | | ANSGA + P245L - | 0,45465 | 0 |
| | | | | ANSGA + P245L + | 0,57634 | 0 |
| | | | | ANSGA + ANSGA - | 0,52487 | 0 |
| 6a L174Bpa GOF | Welch-ANOVA | 1,96E-09 | F(11, 27.79)=16.48 | + - | 0,0274 | 1 |
| | | | | V102A - - | 0,06824 | 0 |
| | | | | V102A - + | 0,17798 | 0 |
| | | | | V102A + - | 0,05711 | 0 |
| | | | | V102A + + | 0,14047 | 0 |
| | | | | V102A + V102A - | 1 | 0 |
| | | | | H134A - - | 0,03748 | 1 |
| | | | | H134A - + | 0,78097 | 0 |
| | | | | H134A - V102A - | 0,11733 | 0 |
| | | | | H134A - V102A + | 0,09493 | 0 |
| | | | | H134A + - | 0,00794 | 1 |
| | | | | H134A + + | 0,98075 | 0 |
| | | | | H134A + V102A - | 0,13565 | 0 |
| | | | | H134A + V102A + | 0,109 | 0 |
| | | | | H134A + H134A - | 0,99969 | 0 |
| | | | | V181K - - | 0,08027 | 0 |
| | | | | V181K - + | 1 | 0 |
| | | | | V181K - V102A - | 0,16642 | 0 |
| | | | | V181K - V102A + | 0,12971 | 0 |
| | | | | V181K - H134A - | 0,97704 | 0 |
| | | | | V181K - H134A + | 0,99971 | 0 |
| | | | | V181K + - | 0,02411 | 1 |
| | | | | V181K + + | 0,99714 | 0 |
| | | | | V181K + V102A - | 0,25962 | 0 |
| | | | | V181K + V102A + | 0,19838 | 0 |
| | | | | V181K + H134A - | 0,52523 | 0 |

| | | | | | | |
|---|---|---|---|---|---|---|
| | | | | V181K + H134A + | 0,78254 | 0 |
| | | | | V181K + V181K - | 0,99643 | 0 |
| | | | | P245L - - | 0,07204 | 0 |
| | | | | P245L - + | 0,99185 | 0 |
| | | | | P245L - V102A - | 0,13335 | 0 |
| | | | | P245L - V102A + | 0,10642 | 0 |
| | | | | P245L - H134A - | 0,99994 | 0 |
| | | | | P245L - H134A + | 1 | 0 |
| | | | | P245L - V181K - | 0,99985 | 0 |
| | | | | P245L - V181K + | 0,83502 | 0 |
| | | | | P245L + - | 0,00859 | 1 |
| | | | | P245L + + | 0,17433 | 0 |
| | | | | P245L + V102A - | 0,87989 | 0 |
| | | | | P245L + V102A + | 0,75451 | 0 |
| | | | | P245L + H134A - | 0,04575 | 1 |
| | | | | P245L + H134A + | 0,07132 | 0 |
| | | | | P245L + V181K - | 0,17807 | 0 |
| | | | | P245L + V181K + | 0,53816 | 0 |
| | | | | P245L + P245L - | 0,07525 | 0 |
| | | | | ANSGA - - | 1 | 0 |
| | | | | ANSGA - + | 0,03595 | 1 |
| | | | | ANSGA - V102A - | 0,07017 | 0 |
| | | | | ANSGA - V102A + | 0,05878 | 0 |
| | | | | ANSGA - H134A - | 0,03528 | 1 |
| | | | | ANSGA - H134A + | 0,00882 | 1 |
| | | | | ANSGA - V181K - | 0,08349 | 0 |
| | | | | ANSGA - V181K + | 0,02626 | 1 |
| | | | | ANSGA - P245L - | 0,07585 | 0 |
| | | | | ANSGA - P245L + | 0,00945 | 1 |
| | | | | ANSGA + - | 0,20711 | 0 |
| | | | | ANSGA + + | 1 | 0 |
| | | | | ANSGA + V102A - | 0,16436 | 0 |
| | | | | ANSGA + V102A + | 0,12773 | 0 |
| | | | | ANSGA + H134A - | 0,98441 | 0 |
| | | | | ANSGA + H134A + | 0,99985 | 0 |
| | | | | ANSGA + V181K - | 1 | 0 |
| | | | | ANSGA + V181K + | 0,99524 | 0 |
| | | | | ANSGA + P245L - | 0,99993 | 0 |
| | | | | ANSGA + P245L + | 0,19104 | 0 |
| | | | | ANSGA + ANSGA - | 0,21572 | 0 |
| 6a A254Azi GOF | Welch-ANOVA | 6,72E-10 | F(11, 32,59)=15.15 | + - | 4,91E-04 | 1 |
| | | | | V102A - - | 0,04066 | 1 |
| | | | | V102A - + | 0,00142 | 1 |
| | | | | V102A + - | 0,01412 | 1 |
| | | | | V102A + + | 0,00167 | 1 |
| | | | | V102A + V102A - | 0,99999 | 0 |
| | | | | H134A - - | 0,55781 | 0 |
| | | | | H134A - + | 7,33E-04 | 1 |
| | | | | H134A - V102A - | 0,52735 | 0 |
| | | | | H134A - V102A + | 0,22626 | 0 |
| | | | | H134A + - | 0,46825 | 0 |
| | | | | H134A + + | 7,91E-04 | 1 |
| | | | | H134A + V102A - | 0,72971 | 0 |
| | | | | H134A + V102A + | 0,39 | 0 |
| | | | | H134A + H134A - | 1 | 0 |
| | | | | V181K - - | 0,16093 | 0 |
| | | | | V181K - + | 0,00111 | 1 |
| | | | | V181K - V102A - | 0,9997 | 0 |
| | | | | V181K - V102A + | 0,97696 | 0 |
| | | | | V181K - H134A - | 0,95662 | 0 |
| | | | | V181K - H134A + | 0,99279 | 0 |
| | | | | V181K + - | 0,07361 | 0 |
| | | | | V181K + + | 0,00152 | 1 |
| | | | | V181K + V102A - | 1 | 0 |
| | | | | V181K + V102A + | 1 | 0 |
| | | | | V181K + H134A - | 0,6125 | 0 |
| | | | | V181K + H134A + | 0,77598 | 0 |
| | | | | V181K + V181K - | 0,99926 | 0 |
| | | | | P245L - - | 0,0256 | 1 |
| | | | | P245L - + | 0,00326 | 1 |
| | | | | P245L - V102A - | 0,71102 | 0 |
| | | | | P245L - V102A + | 0,88014 | 0 |
| | | | | P245L - H134A - | 0,08421 | 0 |
| | | | | P245L - H134A + | 0,11743 | 0 |
| | | | | P245L - V181K - | 0,42383 | 0 |
| | | | | P245L - V181K + | 0,86191 | 0 |
| | | | | P245L + - | 0,01852 | 1 |
| | | | | P245L + + | 0,06035 | 0 |
| | | | | P245L + V102A - | 0,06316 | 0 |
| | | | | P245L + V102A + | 0,07886 | 0 |
| | | | | P245L + H134A - | 0,02703 | 1 |
| | | | | P245L + H134A + | 0,02936 | 1 |
| | | | | P245L + V181K - | 0,04464 | 1 |
| | | | | P245L + V181K + | 0,06886 | 0 |
| | | | | P245L + P245L - | 0,21193 | 0 |
| | | | | ANSGA - - | 0,97723 | 0 |
| | | | | ANSGA - + | 5,78E-04 | 1 |
| | | | | ANSGA - V102A - | 0,13881 | 0 |
| | | | | ANSGA - V102A + | 0,04465 | 1 |
| | | | | ANSGA - H134A - | 0,99315 | 0 |
| | | | | ANSGA - H134A + | 0,96704 | 0 |
| | | | | ANSGA - V181K - | 0,52531 | 0 |
| | | | | ANSGA - V181K + | 0,21769 | 0 |
| | | | | ANSGA - P245L - | 0,03689 | 1 |
| | | | | ANSGA - P245L + | 0,02086 | 1 |
| | | | | ANSGA + - | 0,05384 | 0 |
| | | | | ANSGA + + | 0,99201 | 0 |
| | | | | ANSGA + V102A - | 0,08551 | 0 |
| | | | | ANSGA + V102A + | 0,09222 | 0 |
| | | | | ANSGA + H134A - | 0,06366 | 0 |
| | | | | ANSGA + H134A + | 0,06576 | 0 |
| | | | | ANSGA + V181K - | 0,07614 | 0 |
| | | | | ANSGA + V181K + | 0,08747 | 0 |
| | | | | ANSGA + P245L - | 0,1242 | 0 |
| | | | | ANSGA + P245L + | 0,49877 | 0 |
| | | | | ANSGA + ANSGA - | 0,05749 | 0 |
| 6b | Welch-ANOVA | 0 | F(35, 326.09)=27,12 | A137Bpa+ UV A137Bpa - UV | 6,07E-05 | 1 |
| | | | | L174Bpa -UV A137Bpa - UV | 0,15761 | 0 |
| | | | | L174Bpa -UV A137Bpa+ UV | 3,02E-07 | 1 |
| | | | | L174Bpa +UV A137Bpa - UV | 0,99999 | 0 |
| | | | | L174Bpa +UV A137Bpa+ UV | 0,03217 | 1 |
| | | | | L174Bpa +UV L174Bpa -UV | 0,26637 | 0 |
| | | | | A254Azi-UV A137Bpa - UV | 0,69267 | 0 |
| | | | | A254Azi-UV A137Bpa+ UV | 1,12E-06 | 1 |

| | | |
|---|---|---|
| A254Azi-UV L174Bpa -UV | 0,99847 | 0 |
| A254Azi-UV L174Bpa +UV | 0,57244 | 0 |
| A254Azi+UV A137Bpa - UV | 0 | 1 |
| A254Azi+UV A137Bpa+ UV | 1 | 0 |
| A254Azi+UV L174Bpa -UV | 5,39E-07 | 1 |
| A254Azi+UV L174Bpa +UV | 6,12E-04 | 1 |
| A254Azi+UV A254Azi-UV | 0 | 1 |
| A137Bpa V102A -UV A137Bpa - UV | 0,22314 | 0 |
| A137Bpa V102A -UV A137Bpa+ UV | 4,32E-07 | 1 |
| A137Bpa V102A -UV L174Bpa -UV | 1 | 0 |
| A137Bpa V102A -UV L174Bpa +UV | 0,32298 | 0 |
| A137Bpa V102A -UV A254Azi-UV | 0,99997 | 0 |
| A137Bpa V102A -UV A254Azi+UV | 0 | 1 |
| A137Bpa V102A+UV A137Bpa - UV | 0,0414 | 1 |
| A137Bpa V102A+UV A137Bpa+ UV | 1,99E-07 | 1 |
| A137Bpa V102A+UV L174Bpa -UV | 1 | 0 |
| A137Bpa V102A+UV L174Bpa +UV | 0,16317 | 0 |
| A137Bpa V102A+UV A254Azi-UV | 0,28016 | 0 |
| A137Bpa V102A+UV A254Azi+UV | 1,59E-07 | 1 |
| A137Bpa V102A+UV A137Bpa V102A -UV | 0,99277 | 0 |
| L174Bpa V102A-UV A137Bpa - UV | 0,98794 | 0 |
| L174Bpa V102A-UV A137Bpa+ UV | 1,41E-06 | 1 |
| L174Bpa V102A-UV L174Bpa -UV | 1 | 0 |
| L174Bpa V102A-UV L174Bpa +UV | 0,79878 | 0 |
| L174Bpa V102A-UV A254Azi-UV | 1 | 0 |
| L174Bpa V102A-UV A254Azi+UV | 0 | 1 |
| L174Bpa V102A-UV A137Bpa V102A -UV | 1 | 0 |
| L174Bpa V102A-UV A137Bpa V102A+UV | 0,99718 | 0 |
| L174Bpa V102A+UV A137Bpa - UV | 0,98644 | 0 |
| L174Bpa V102A+UV A137Bpa+ UV | 1,37E-06 | 1 |
| L174Bpa V102A+UV L174Bpa -UV | 1 | 0 |
| L174Bpa V102A+UV L174Bpa +UV | 0,79289 | 0 |
| L174Bpa V102A+UV A254Azi-UV | 1 | 0 |
| L174Bpa V102A+UV A254Azi+UV | 0 | 1 |
| L174Bpa V102A+UV A137Bpa V102A -UV | 1 | 0 |
| L174Bpa V102A+UV A137Bpa V102A+UV | 0,99717 | 0 |
| L174Bpa V102A+UV L174Bpa V102A-UV | 1 | 0 |
| A254Azi V102A-UV A137Bpa - UV | 0,99998 | 0 |
| A254Azi V102A-UV A137Bpa+ UV | 6,33E-04 | 1 |
| A254Azi V102A-UV L174Bpa -UV | 3,46E-04 | 1 |
| A254Azi V102A-UV L174Bpa +UV | 1 | 0 |
| A254Azi V102A-UV A254Azi-UV | 0,00752 | 1 |
| A254Azi V102A-UV A254Azi+UV | 4,60E-08 | 1 |
| A254Azi V102A-UV A137Bpa V102A -UV | 6,29E-04 | 1 |
| A254Azi V102A-UV A137Bpa V102A+UV | 6,29E-05 | 1 |
| A254Azi V102A-UV L174Bpa V102A-UV | 0,22494 | 0 |
| A254Azi V102A-UV L174Bpa V102A+UV | 0,21357 | 0 |
| A254Azi V102A+UV A137Bpa - UV | 1 | 0 |
| A254Azi V102A+UV A137Bpa+ UV | 5,15E-04 | 1 |
| A254Azi V102A+UV L174Bpa -UV | 5,40E-04 | 1 |
| A254Azi V102A+UV L174Bpa +UV | 1 | 0 |
| A254Azi V102A+UV A254Azi-UV | 0,01145 | 1 |
| A254Azi V102A+UV A254Azi+UV | 1,17E-08 | 1 |
| A254Azi V102A+UV A137Bpa V102A -UV | 9,74E-04 | 1 |
| A254Azi V102A+UV A137Bpa V102A+UV | 9,46E-05 | 1 |
| A254Azi V102A+UV L174Bpa V102A-UV | 0,2811 | 0 |
| A254Azi V102A+UV L174Bpa V102A+UV | 0,26811 | 0 |
| A254Azi V102A+UV A254Azi V102A-UV | 1 | 0 |
| A137Bpa H134A-UV A137Bpa - UV | 0,01778 | 1 |
| A137Bpa H134A-UV A137Bpa+ UV | 1,09E-07 | 1 |
| A137Bpa H134A-UV L174Bpa -UV | 0,99578 | 0 |
| A137Bpa H134A-UV L174Bpa +UV | 0,11269 | 0 |
| A137Bpa H134A-UV A254Azi-UV | 0,02572 | 1 |
| A137Bpa H134A-UV A254Azi+UV | 1,71E-07 | 1 |
| A137Bpa H134A-UV A137Bpa V102A -UV | 0,59912 | 0 |
| A137Bpa H134A-UV A137Bpa V102A+UV | 0,54457 | 0 |
| A137Bpa H134A-UV L174Bpa V102A-UV | 0,96446 | 0 |
| A137Bpa H134A-UV L174Bpa V102A+UV | 0,96386 | 0 |
| A137Bpa H134A-UV A254Azi V102A-UV | 2,32E-05 | 1 |
| A137Bpa H134A-UV A254Azi V102A+UV | 3,45E-05 | 1 |
| A137Bpa H134A+ UV A137Bpa - UV | 0,01978 | 1 |
| A137Bpa H134A+ UV A137Bpa+ UV | 1,19E-07 | 1 |
| A137Bpa H134A+ UV L174Bpa -UV | 0,99837 | 0 |
| A137Bpa H134A+ UV L174Bpa +UV | 0,11819 | 0 |
| A137Bpa H134A+ UV A254Azi-UV | 0,03577 | 1 |
| A137Bpa H134A+ UV A254Azi+UV | 1,70E-07 | 1 |
| A137Bpa H134A+ UV A137Bpa V102A -UV | 0,67729 | 0 |
| A137Bpa H134A+ UV A137Bpa V102A+UV | 0,79482 | 0 |
| A137Bpa H134A+ UV L174Bpa V102A-UV | 0,97223 | 0 |
| A137Bpa H134A+ UV L174Bpa V102A+UV | 0,97179 | 0 |
| A137Bpa H134A+ UV A254Azi V102A-UV | 2,64E-05 | 1 |
| A137Bpa H134A+ UV A254Azi V102A+UV | 3,92E-05 | 1 |
| A137Bpa H134A+ UV A137Bpa H134A-UV | 1 | 0 |
| L174Bpa H134A-UV A137Bpa - UV | 0,99997 | 0 |
| L174Bpa H134A-UV A137Bpa+ UV | 4,67E-06 | 1 |
| L174Bpa H134A-UV L174Bpa -UV | 0,81547 | 0 |
| L174Bpa H134A-UV L174Bpa +UV | 0,94675 | 0 |
| L174Bpa H134A-UV A254Azi-UV | 0,99998 | 0 |
| L174Bpa H134A-UV A254Azi+UV | 0 | 1 |
| L174Bpa H134A-UV A137Bpa V102A -UV | 0,90893 | 0 |
| L174Bpa H134A-UV A137Bpa V102A+UV | 0,3171 | 0 |
| L174Bpa H134A-UV L174Bpa V102A-UV | 1 | 0 |
| L174Bpa H134A-UV L174Bpa V102A+UV | 1 | 0 |
| L174Bpa H134A-UV A254Azi V102A-UV | 0,47064 | 0 |
| L174Bpa H134A-UV A254Azi V102A+UV | 0,56013 | 0 |
| L174Bpa H134A-UV A137Bpa H134A-UV | 0,1405 | 0 |
| L174Bpa H134A-UV A137Bpa H134A+ UV | 0,15648 | 0 |
| L174Bpa H134A+UV A137Bpa - UV | 1 | 0 |
| L174Bpa H134A+UV A137Bpa+ UV | 1,05E-05 | 1 |
| L174Bpa H134A+UV L174Bpa -UV | 0,27252 | 0 |
| L174Bpa H134A+UV L174Bpa +UV | 0,98969 | 0 |
| L174Bpa H134A+UV A254Azi-UV | 0,95338 | 0 |
| L174Bpa H134A+UV A254Azi+UV | 0 | 1 |
| L174Bpa H134A+UV A137Bpa V102A -UV | 0,38115 | 0 |
| L174Bpa H134A+UV A137Bpa V102A+UV | 0,04254 | 1 |
| L174Bpa H134A+UV L174Bpa V102A-UV | 0,99999 | 0 |
| L174Bpa H134A+UV L174Bpa V102A+UV | 0,99999 | 0 |
| L174Bpa H134A+UV A254Azi V102A-UV | 0,78912 | 0 |
| L174Bpa H134A+UV A254Azi V102A+UV | 0,85926 | 0 |
| L174Bpa H134A+UV A137Bpa H134A-UV | 0,01427 | 1 |
| L174Bpa H134A+UV A137Bpa H134A+ UV | 0,01637 | 1 |
| L174Bpa H134A+UV L174Bpa H134A-UV | 1 | 0 |
| A254Azi H134A-UV A137Bpa - UV | 0,10016 | 0 |
| A254Azi H134A-UV A137Bpa+ UV | 3,32E-07 | 1 |
| A254Azi H134A-UV L174Bpa -UV | 1 | 0 |
| A254Azi H134A-UV L174Bpa +UV | 0,23641 | 0 |
| A254Azi H134A-UV A254Azi-UV | 0,9319 | 0 |

| | | | | | |
|---|---|---|---|---|---|
| | | | A254Azi H134A-UV A254Azi+UV | 5,68E-07 | 1 |
| | | | A254Azi H134A-UV A137Bpa V102A -UV | 1 | 0 |
| | | | A254Azi H134A-UV A137Bpa V102A+UV | 0,99975 | 0 |
| | | | A254Azi H134A-UV L174Bpa V102A-UV | 0,99998 | 0 |
| | | | A254Azi H134A-UV L174Bpa V102A+UV | 0,99998 | 0 |
| | | | A254Azi H134A-UV A254Azi V102A-UV | 1,89E-04 | 1 |
| | | | A254Azi H134A-UV A254Azi V102A+UV | 2,89E-04 | 1 |
| | | | A254Azi H134A-UV A137Bpa H134A-UV | 0,29749 | 0 |
| | | | A254Azi H134A-UV A137Bpa H134A+ UV | 0,42385 | 0 |
| | | | A254Azi H134A-UV L174Bpa H134A-UV | 0,63113 | 0 |
| | | | A254Azi H134A-UV L174Bpa H134A+UV | 0,13581 | 0 |
| | | | A254Azi H134A+UV A137Bpa - UV | 0,05986 | 0 |
| | | | A254Azi H134A+UV A137Bpa+ UV | 2,49E-07 | 1 |
| | | | A254Azi H134A+UV L174Bpa -UV | 1 | 0 |
| | | | A254Azi H134A+UV L174Bpa +UV | 0,19078 | 0 |
| | | | A254Azi H134A+UV A254Azi-UV | 0,57706 | 0 |
| | | | A254Azi H134A+UV A254Azi+UV | 7,58E-07 | 1 |
| | | | A254Azi H134A+UV A137Bpa V102A -UV | 0,99996 | 0 |
| | | | A254Azi H134A+UV A137Bpa V102A+UV | 1 | 0 |
| | | | A254Azi H134A+UV L174Bpa V102A-UV | 0,99946 | 0 |
| | | | A254Azi H134A+UV L174Bpa V102A+UV | 0,99946 | 0 |
| | | | A254Azi H134A+UV A254Azi V102A-UV | 9,80E-05 | 1 |
| | | | A254Azi H134A+UV A254Azi V102A+UV | 1,48E-04 | 1 |
| | | | A254Azi H134A+UV A137Bpa H134A-UV | 0,39786 | 0 |
| | | | A254Azi H134A+UV A137Bpa H134A+ UV | 0,59857 | 0 |
| | | | A254Azi H134A+UV L174Bpa H134A-UV | 0,43471 | 0 |
| | | | A254Azi H134A+UV L174Bpa H134A+UV | 0,06905 | 0 |
| | | | A254Azi H134A+UV A254Azi H134A-UV | 1 | 0 |
| | | | A137Bpa V181K-UV A137Bpa - UV | 0,02129 | 1 |
| | | | A137Bpa V181K-UV A137Bpa+ UV | 1,25E-07 | 1 |
| | | | A137Bpa V181K-UV L174Bpa -UV | 0,99925 | 0 |
| | | | A137Bpa V181K-UV L174Bpa +UV | 0,12208 | 0 |
| | | | A137Bpa V181K-UV A254Azi-UV | 0,04535 | 1 |
| | | | A137Bpa V181K-UV A254Azi+UV | 1,69E-07 | 1 |
| | | | A137Bpa V181K-UV A137Bpa V102A -UV | 0,73319 | 0 |
| | | | A137Bpa V181K-UV A137Bpa V102A+UV | 0,93627 | 0 |
| | | | A137Bpa V181K-UV L174Bpa V102A-UV | 0,97682 | 0 |
| | | | A137Bpa V181K-UV L174Bpa V102A+UV | 0,97648 | 0 |
| | | | A137Bpa V181K-UV A254Azi V102A-UV | 2,87E-05 | 1 |
| | | | A137Bpa V181K-UV A254Azi V102A+UV | 4,27E-05 | 1 |
| | | | A137Bpa V181K-UV A137Bpa H134A-UV | 1 | 0 |
| | | | A137Bpa V181K-UV A137Bpa H134A+ UV | 1 | 0 |
| | | | A137Bpa V181K-UV L174Bpa H134A-UV | 0,16856 | 0 |
| | | | A137Bpa V181K-UV L174Bpa H134A+UV | 0,01799 | 1 |
| | | | A137Bpa V181K-UV A254Azi H134A-UV | 0,53707 | 0 |
| | | | A137Bpa V181K-UV A254Azi H134A+UV | 0,75986 | 0 |
| | | | A137Bpa V181K+UV A137Bpa - UV | 0,03347 | 1 |
| | | | A137Bpa V181K+UV A137Bpa+ UV | 1,76E-07 | 1 |
| | | | A137Bpa V181K+UV L174Bpa -UV | 1 | 0 |
| | | | A137Bpa V181K+UV L174Bpa +UV | 0,14932 | 0 |
| | | | A137Bpa V181K+UV A254Azi-UV | 0,15144 | 0 |
| | | | A137Bpa V181K+UV A254Azi+UV | 1,62E-07 | 1 |
| | | | A137Bpa V181K+UV A137Bpa V102A -UV | 0,95296 | 0 |
| | | | A137Bpa V181K+UV A137Bpa V102A+UV | 1 | 0 |
| | | | A137Bpa V181K+UV L174Bpa V102A-UV | 0,99385 | 0 |
| | | | A137Bpa V181K+UV L174Bpa V102A+UV | 0,99381 | 0 |
| | | | A137Bpa V181K+UV A254Azi V102A-UV | 5,02E-05 | 1 |
| | | | A137Bpa V181K+UV A254Azi V102A+UV | 7,50E-05 | 1 |
| | | | A137Bpa V181K+UV A137Bpa H134A-UV | 0,03203 | 1 |
| | | | A137Bpa V181K+UV A137Bpa H134A+ UV | 0,2525 | 0 |
| | | | A137Bpa V181K+UV L174Bpa H134A-UV | 0,25911 | 0 |
| | | | A137Bpa V181K+UV L174Bpa H134A+UV | 0,0321 | 1 |
| | | | A137Bpa V181K+UV A254Azi H134A-UV | 0,9702 | 0 |
| | | | A137Bpa V181K+UV A254Azi H134A+UV | 0,9999 | 0 |
| | | | A137Bpa V181K+UV A137Bpa V181K-UV | 0,74029 | 0 |
| | | | L174Bpa V181K-UV A137Bpa - UV | 1 | 0 |
| | | | L174Bpa V181K-UV A137Bpa+ UV | 6,06E-05 | 1 |
| | | | L174Bpa V181K-UV L174Bpa -UV | 0,13211 | 0 |
| | | | L174Bpa V181K-UV L174Bpa +UV | 0,99999 | 0 |
| | | | L174Bpa V181K-UV A254Azi-UV | 0,64171 | 0 |
| | | | L174Bpa V181K-UV A254Azi+UV | 0 | 1 |
| | | | L174Bpa V181K-UV A137Bpa V102A -UV | 0,18945 | 0 |
| | | | L174Bpa V181K-UV A137Bpa V102A+UV | 0,03377 | 1 |
| | | | L174Bpa V181K-UV L174Bpa V102A-UV | 0,98403 | 0 |
| | | | L174Bpa V181K-UV L174Bpa V102A+UV | 0,98212 | 0 |
| | | | L174Bpa V181K-UV A254Azi V102A-UV | 0,99997 | 0 |
| | | | L174Bpa V181K-UV A254Azi V102A+UV | 1 | 0 |
| | | | L174Bpa V181K-UV A137Bpa H134A-UV | 0,01451 | 1 |
| | | | L174Bpa V181K-UV A137Bpa H134A+ UV | 0,01614 | 1 |
| | | | L174Bpa V181K-UV L174Bpa H134A-UV | 0,99994 | 0 |
| | | | L174Bpa V181K-UV L174Bpa H134A+UV | 1 | 0 |
| | | | L174Bpa V181K-UV A254Azi H134A-UV | 0,0826 | 0 |
| | | | L174Bpa V181K-UV A254Azi H134A+UV | 0,04896 | 1 |
| | | | L174Bpa V181K-UV A137Bpa V181K-UV | 0,01736 | 1 |
| | | | L174Bpa V181K-UV A137Bpa V181K+UV | 0,02731 | 1 |
| | | | L174Bpa V181K+UV A137Bpa - UV | 0,98059 | 0 |
| | | | L174Bpa V181K+UV A137Bpa+ UV | 0,00207 | 1 |
| | | | L174Bpa V181K+UV L174Bpa -UV | 4,56E-05 | 1 |
| | | | L174Bpa V181K+UV L174Bpa +UV | 1 | 0 |
| | | | L174Bpa V181K+UV A254Azi-UV | 8,58E-04 | 1 |
| | | | L174Bpa V181K+UV A254Azi+UV | 6,03E-07 | 1 |
| | | | L174Bpa V181K+UV A137Bpa V102A -UV | 8,87E-05 | 1 |
| | | | L174Bpa V181K+UV A137Bpa V102A+UV | 1,50E-05 | 1 |
| | | | L174Bpa V181K+UV L174Bpa V102A-UV | 0,04915 | 1 |
| | | | L174Bpa V181K+UV L174Bpa V102A+UV | 0,04564 | 1 |
| | | | L174Bpa V181K+UV A254Azi V102A-UV | 1 | 0 |
| | | | L174Bpa V181K+UV A254Azi V102A+UV | 1 | 0 |
| | | | L174Bpa V181K+UV A137Bpa H134A-UV | 6,62E-06 | 1 |
| | | | L174Bpa V181K+UV A137Bpa H134A+ UV | 7,38E-06 | 1 |
| | | | L174Bpa V181K+UV L174Bpa H134A-UV | 0,1168 | 0 |
| | | | L174Bpa V181K+UV L174Bpa H134A+UV | 0,29604 | 0 |
| | | | L174Bpa V181K+UV A254Azi H134A-UV | 3,58E-05 | 1 |
| | | | L174Bpa V181K+UV A254Azi H134A+UV | 2,15E-05 | 1 |
| | | | L174Bpa V181K+UV A137Bpa V181K-UV | 7,89E-06 | 1 |
| | | | L174Bpa V181K+UV A137Bpa V181K+UV | 1,28E-05 | 1 |
| | | | L174Bpa V181K+UV L174Bpa V181K-UV | 0,97825 | 0 |
| | | | A254Azi V181K-UV A137Bpa - UV | 0,97653 | 0 |
| | | | A254Azi V181K-UV A137Bpa+ UV | 0,00304 | 1 |
| | | | A254Azi V181K-UV L174Bpa -UV | 1,76E-05 | 1 |
| | | | A254Azi V181K-UV L174Bpa +UV | 1 | 0 |
| | | | A254Azi V181K-UV A254Azi-UV | 5,62E-04 | 1 |
| | | | A254Azi V181K-UV A254Azi+UV | 1,25E-06 | 1 |
| | | | A254Azi V181K-UV A137Bpa V102A -UV | 3,27E-05 | 1 |
| | | | A254Azi V181K-UV A137Bpa V102A+UV | 1,80E-06 | 1 |
| | | | A254Azi V181K-UV L174Bpa V102A-UV | 0,04742 | 1 |
| | | | A254Azi V181K-UV L174Bpa V102A+UV | 0,04403 | 1 |
| | | | A254Azi V181K-UV A254Azi V102A-UV | 1 | 0 |

| | | |
|---|---|---|
| A254Azi V181K-UV A254Azi V102A+UV | 1 | 0 |
| A254Azi V181K-UV A137Bpa H134A-UV | 4,81E-07 | 1 |
| A254Azi V181K-UV A137Bpa H134A+ UV | 5,72E-07 | 1 |
| A254Azi V181K-UV L174Bpa H134A-UV | 0,11775 | 0 |
| A254Azi V181K-UV L174Bpa H134A+UV | 0,30181 | 0 |
| A254Azi V181K-UV A254Azi H134A-UV | 7,28E-06 | 1 |
| A254Azi V181K-UV A254Azi H134A+UV | 3,18E-06 | 1 |
| A254Azi V181K-UV A137Bpa V181K-UV | 6,42E-07 | 1 |
| A254Azi V181K-UV A137Bpa V181K+UV | 1,31E-06 | 1 |
| A254Azi V181K-UV L174Bpa V181K-UV | 0,97417 | 0 |
| A254Azi V181K-UV L174Bpa V181K+UV | 1 | 0 |
| A254Azi V181K+UV A137Bpa - UV | 0,7788 | 0 |
| A254Azi V181K+UV A137Bpa+ UV | 0,01058 | 1 |
| A254Azi V181K+UV L174Bpa -UV | 4,39E-06 | 1 |
| A254Azi V181K+UV L174Bpa +UV | 1 | 0 |
| A254Azi V181K+UV A254Azi-UV | 1,19E-04 | 1 |
| A254Azi V181K+UV A254Azi+UV | 1,18E-05 | 1 |
| A254Azi V181K+UV A137Bpa V102A -UV | 8,13E-06 | 1 |
| A254Azi V181K+UV A137Bpa V102A+UV | 5,52E-07 | 1 |
| A254Azi V181K+UV L174Bpa V102A-UV | 0,01159 | 1 |
| A254Azi V181K+UV L174Bpa V102A+UV | 0,01064 | 1 |
| A254Azi V181K+UV A254Azi V102A-UV | 0,99998 | 0 |
| A254Azi V181K+UV A254Azi V102A+UV | 0,9999 | 0 |
| A254Azi V181K+UV A137Bpa H134A-UV | 1,31E-07 | 1 |
| A254Azi V181K+UV A137Bpa H134A+ UV | 1,62E-07 | 1 |
| A254Azi V181K+UV L174Bpa H134A-UV | 0,02944 | 1 |
| A254Azi V181K+UV L174Bpa H134A+UV | 0,09017 | 0 |
| A254Azi V181K+UV A254Azi H134A-UV | 2,08E-06 | 1 |
| A254Azi V181K+UV A254Azi H134A+UV | 9,57E-07 | 1 |
| A254Azi V181K+UV A137Bpa V181K-UV | 1,86E-07 | 1 |
| A254Azi V181K+UV A137Bpa V181K+UV | 4,02E-07 | 1 |
| A254Azi V181K+UV L174Bpa V181K-UV | 0,76666 | 0 |
| A254Azi V181K+UV L174Bpa V181K+UV | 1 | 0 |
| A254Azi V181K+UV A254Azi V181K-UV | 1 | 0 |
| A137Bpa P245L-UV A137Bpa - UV | 0,09163 | 0 |
| A137Bpa P245L-UV A137Bpa+ UV | 2,22E-07 | 1 |
| A137Bpa P245L-UV L174Bpa -UV | 1 | 0 |
| A137Bpa P245L-UV L174Bpa +UV | 0,21685 | 0 |
| A137Bpa P245L-UV A254Azi-UV | 0,94513 | 0 |
| A137Bpa P245L-UV A254Azi+UV | 7,24E-07 | 1 |
| A137Bpa P245L-UV A137Bpa V102A -UV | 1 | 0 |
| A137Bpa P245L-UV A137Bpa V102A+UV | 1 | 0 |
| A137Bpa P245L-UV L174Bpa V102A-UV | 0,99992 | 0 |
| A137Bpa P245L-UV L174Bpa V102A+UV | 0,99993 | 0 |
| A137Bpa P245L-UV A254Azi V102A-UV | 1,49E-04 | 1 |
| A137Bpa P245L-UV A254Azi V102A+UV | 2,32E-04 | 1 |
| A137Bpa P245L-UV A137Bpa H134A-UV | 0,99874 | 0 |
| A137Bpa P245L-UV A137Bpa H134A+ UV | 0,99976 | 0 |
| A137Bpa P245L-UV L174Bpa H134A-UV | 0,61631 | 0 |
| A137Bpa P245L-UV L174Bpa H134A+UV | 0,13406 | 0 |
| A137Bpa P245L-UV A254Azi H134A-UV | 1 | 0 |
| A137Bpa P245L-UV A254Azi H134A+UV | 1 | 0 |
| A137Bpa P245L-UV A137Bpa V181K-UV | 0,99991 | 0 |
| A137Bpa P245L-UV A137Bpa V181K+UV | 1 | 0 |
| A137Bpa P245L-UV L174Bpa V181K-UV | 0,07532 | 0 |
| A137Bpa P245L-UV L174Bpa V181K+UV | 2,36E-05 | 1 |
| A137Bpa P245L-UV A254Azi V181K-UV | 6,30E-06 | 1 |
| A137Bpa P245L-UV A254Azi V181K+UV | 1,68E-06 | 1 |
| A137Bpa P245L+UV A137Bpa - UV | 0,94237 | 0 |
| A137Bpa P245L+UV A137Bpa+ UV | 1,36E-06 | 1 |
| A137Bpa P245L+UV L174Bpa -UV | 0,9985 | 0 |
| A137Bpa P245L+UV L174Bpa +UV | 0,72513 | 0 |
| A137Bpa P245L+UV A254Azi-UV | 1 | 0 |
| A137Bpa P245L+UV A254Azi+UV | 0 | 1 |
| A137Bpa P245L+UV A137Bpa V102A -UV | 0,99993 | 0 |
| A137Bpa P245L+UV A137Bpa V102A+UV | 0,76163 | 0 |
| A137Bpa P245L+UV L174Bpa V102A-UV | 1 | 0 |
| A137Bpa P245L+UV L174Bpa V102A+UV | 1 | 0 |
| A137Bpa P245L+UV A254Azi V102A-UV | 0,0545 | 0 |
| A137Bpa P245L+UV A254Azi V102A+UV | 0,07707 | 0 |
| A137Bpa P245L+UV A137Bpa H134A-UV | 0,39865 | 0 |
| A137Bpa P245L+UV A137Bpa H134A+ UV | 0,44071 | 0 |
| A137Bpa P245L+UV L174Bpa H134A-UV | 1 | 0 |
| A137Bpa P245L+UV L174Bpa H134A+UV | 0,9997 | 0 |
| A137Bpa P245L+UV A254Azi H134A-UV | 0,98186 | 0 |
| A137Bpa P245L+UV A254Azi H134A+UV | 0,89148 | 0 |
| A137Bpa P245L+UV A137Bpa V181K-UV | 0,47165 | 0 |
| A137Bpa P245L+UV A137Bpa V181K+UV | 0,66501 | 0 |
| A137Bpa P245L+UV L174Bpa V181K-UV | 0,92506 | 0 |
| A137Bpa P245L+UV L174Bpa V181K+UV | 0,00675 | 1 |
| A137Bpa P245L+UV A254Azi V181K-UV | 0,0063 | 1 |
| A137Bpa P245L+UV A254Azi V181K+UV | 0,00123 | 1 |
| A137Bpa P245L+UV A137Bpa P245L-UV | 0,97741 | 0 |
| L174Bpa P245L-UV A137Bpa - UV | 0,81995 | 0 |
| L174Bpa P245L-UV A137Bpa+ UV | 9,37E-07 | 1 |
| L174Bpa P245L-UV L174Bpa -UV | 0,99998 | 0 |
| L174Bpa P245L-UV L174Bpa +UV | 0,61674 | 0 |
| L174Bpa P245L-UV A254Azi-UV | 1 | 0 |
| L174Bpa P245L-UV A254Azi+UV | 0 | 1 |
| L174Bpa P245L-UV A137Bpa V102A -UV | 1 | 0 |
| L174Bpa P245L-UV A137Bpa V102A+UV | 0,92324 | 0 |
| L174Bpa P245L-UV L174Bpa V102A-UV | 1 | 0 |
| L174Bpa P245L-UV L174Bpa V102A+UV | 1 | 0 |
| L174Bpa P245L-UV A254Azi V102A-UV | 0,02058 | 1 |
| L174Bpa P245L-UV A254Azi V102A+UV | 0,03024 | 1 |
| L174Bpa P245L-UV A137Bpa H134A-UV | 0,60066 | 0 |
| L174Bpa P245L-UV A137Bpa H134A+ UV | 0,64935 | 0 |
| L174Bpa P245L-UV L174Bpa H134A-UV | 1 | 0 |
| L174Bpa P245L-UV L174Bpa H134A+UV | 0,99083 | 0 |
| L174Bpa P245L-UV A254Azi H134A-UV | 0,99918 | 0 |
| L174Bpa P245L-UV A254Azi H134A+UV | 0,9809 | 0 |
| L174Bpa P245L-UV A137Bpa V181K-UV | 0,68337 | 0 |
| L174Bpa P245L-UV A137Bpa V181K+UV | 0,85949 | 0 |
| L174Bpa P245L-UV L174Bpa V181K-UV | 0,78371 | 0 |
| L174Bpa P245L-UV L174Bpa V181K+UV | 0,0023 | 1 |
| L174Bpa P245L-UV A254Azi V181K-UV | 0,00198 | 1 |
| L174Bpa P245L-UV A254Azi V181K+UV | 3,86E-04 | 1 |
| L174Bpa P245L-UV A137Bpa P245L-UV | 0,99854 | 0 |
| L174Bpa P245L-UV A137Bpa P245L+UV | 1 | 0 |
| L174Bpa P245L+UV A137Bpa - UV | 1 | 0 |
| L174Bpa P245L+UV A137Bpa+ UV | 4,58E-05 | 1 |
| L174Bpa P245L+UV L174Bpa -UV | 0,01365 | 1 |
| L174Bpa P245L+UV L174Bpa +UV | 0,99998 | 0 |
| L174Bpa P245L+UV A254Azi-UV | 0,25558 | 0 |
| L174Bpa P245L+UV A254Azi+UV | 0 | 1 |
| L174Bpa P245L+UV A137Bpa V102A -UV | 0,02303 | 1 |
| L174Bpa P245L+UV A137Bpa V102A+UV | 0,00115 | 1 |

| | | |
|---|---|---|
| L174Bpa P245L+UV L174Bpa V102A-UV | 0,94567 | 0 |
| L174Bpa P245L+UV L174Bpa V102A+UV | 0,9399 | 0 |
| L174Bpa P245L+UV A254Azi V102A-UV | 0,99969 | 0 |
| L174Bpa P245L+UV A254Azi V102A+UV | 0,99994 | 0 |
| L174Bpa P245L+UV A137Bpa H134A-UV | 3,24E-04 | 1 |
| L174Bpa P245L+UV A137Bpa H134A+ UV | 3,80E-04 | 1 |
| L174Bpa P245L+UV L174Bpa H134A-UV | 0,9994 | 0 |
| L174Bpa P245L+UV L174Bpa H134A+UV | 1 | 0 |
| L174Bpa P245L+UV A254Azi H134A-UV | 0,00486 | 1 |
| L174Bpa P245L+UV A254Azi H134A+UV | 0,00206 | 1 |
| L174Bpa P245L+UV A137Bpa V181K-UV | 4,23E-04 | 1 |
| L174Bpa P245L+UV A137Bpa V181K+UV | 8,39E-04 | 1 |
| L174Bpa P245L+UV L174Bpa V181K-UV | 1 | 0 |
| L174Bpa P245L+UV L174Bpa V181K+UV | 0,9172 | 0 |
| L174Bpa P245L+UV A254Azi V181K-UV | 0,91502 | 0 |
| L174Bpa P245L+UV A254Azi V181K+UV | 0,57118 | 0 |
| L174Bpa P245L+UV A137Bpa P245L-UV | 0,0047 | 1 |
| L174Bpa P245L+UV A137Bpa P245L+UV | 0,74823 | 0 |
| L174Bpa P245L+UV L174Bpa P245L-UV | 0,48289 | 0 |
| A254Azi P245L-UV A137Bpa - UV | 0,05603 | 0 |
| A254Azi P245L-UV A137Bpa+ UV | 2,43E-07 | 1 |
| A254Azi P245L-UV L174Bpa -UV | 1 | 0 |
| A254Azi P245L-UV L174Bpa +UV | 0,18596 | 0 |
| A254Azi P245L-UV A254Azi-UV | 0,50415 | 0 |
| A254Azi P245L-UV A254Azi+UV | 7,62E-07 | 1 |
| A254Azi P245L-UV A137Bpa V102A -UV | 0,99983 | 0 |
| A254Azi P245L-UV A137Bpa V102A+UV | 1 | 0 |
| A254Azi P245L-UV L174Bpa V102A-UV | 0,99925 | 0 |
| A254Azi P245L-UV L174Bpa V102A+UV | 0,99926 | 0 |
| A254Azi P245L-UV A254Azi V102A-UV | 9,17E-05 | 1 |
| A254Azi P245L-UV A254Azi V102A+UV | 1,38E-04 | 1 |
| A254Azi P245L-UV A137Bpa H134A-UV | 0,22837 | 0 |
| A254Azi P245L-UV A137Bpa H134A+ UV | 0,41726 | 0 |
| A254Azi P245L-UV L174Bpa H134A-UV | 0,41032 | 0 |
| A254Azi P245L-UV L174Bpa H134A+UV | 0,063 | 0 |
| A254Azi P245L-UV A254Azi H134A-UV | 1 | 0 |
| A254Azi P245L-UV A254Azi H134A+UV | 1 | 0 |
| A254Azi P245L-UV A137Bpa V181K-UV | 0,61383 | 0 |
| A254Azi P245L-UV A137Bpa V181K+UV | 0,99979 | 0 |
| A254Azi P245L-UV L174Bpa V181K-UV | 0,04581 | 1 |
| A254Azi P245L-UV L174Bpa V181K+UV | 2,07E-05 | 1 |
| A254Azi P245L-UV A254Azi V181K-UV | 2,89E-06 | 1 |
| A254Azi P245L-UV A254Azi V181K+UV | 8,74E-07 | 1 |
| A254Azi P245L-UV A137Bpa P245L-UV | 1 | 0 |
| A254Azi P245L-UV A137Bpa P245L+UV | 0,87004 | 0 |
| A254Azi P245L-UV L174Bpa P245L-UV | 0,97381 | 0 |
| A254Azi P245L-UV L174Bpa P245L+UV | 0,00186 | 1 |
| A254Azi P245L+UV A137Bpa - UV | 0,99574 | 0 |
| A254Azi P245L+UV A137Bpa+ UV | 0,00153 | 1 |
| A254Azi P245L+UV L174Bpa -UV | 9,17E-05 | 1 |
| A254Azi P245L+UV L174Bpa +UV | 1 | 0 |
| A254Azi P245L+UV A254Azi-UV | 0,00187 | 1 |
| A254Azi P245L+UV A254Azi+UV | 3,58E-07 | 1 |
| A254Azi P245L+UV A137Bpa V102A -UV | 1,72E-04 | 1 |
| A254Azi P245L+UV A137Bpa V102A+UV | 2,22E-05 | 1 |
| A254Azi P245L+UV L174Bpa V102A-UV | 0,08576 | 0 |
| A254Azi P245L+UV L174Bpa V102A+UV | 0,08025 | 0 |
| A254Azi P245L+UV A254Azi V102A-UV | 1 | 0 |
| A254Azi P245L+UV A254Azi V102A+UV | 1 | 0 |
| A254Azi P245L+UV A137Bpa H134A-UV | 8,85E-06 | 1 |
| A254Azi P245L+UV A137Bpa H134A+ UV | 9,96E-06 | 1 |
| A254Azi P245L+UV L174Bpa H134A-UV | 0,20128 | 0 |
| A254Azi P245L+UV L174Bpa H134A+UV | 0,45185 | 0 |
| A254Azi P245L+UV A254Azi H134A-UV | 5,96E-05 | 1 |
| A254Azi P245L+UV A254Azi H134A+UV | 3,30E-05 | 1 |
| A254Azi P245L+UV A137Bpa V181K-UV | 1,08E-05 | 1 |
| A254Azi P245L+UV A137Bpa V181K+UV | 1,82E-05 | 1 |
| A254Azi P245L+UV L174Bpa V181K-UV | 0,99516 | 0 |
| A254Azi P245L+UV L174Bpa V181K+UV | 1 | 0 |
| A254Azi P245L+UV A254Azi V181K-UV | 1 | 0 |
| A254Azi P245L+UV A254Azi V181K+UV | 1 | 0 |
| A254Azi P245L+UV A137Bpa P245L-UV | 4,33E-05 | 1 |
| A254Azi P245L+UV A137Bpa P245L+UV | 0,01457 | 1 |
| A254Azi P245L+UV L174Bpa P245L-UV | 0,00512 | 1 |
| A254Azi P245L+UV L174Bpa P245L+UV | 0,97575 | 0 |
| A254Azi P245L+UV A254Azi P245L-UV | 3,13E-05 | 1 |
| A137Bpa ANSGA-UV A137Bpa - UV | 0,14656 | 0 |
| A137Bpa ANSGA-UV A137Bpa+ UV | 3,43E-07 | 1 |
| A137Bpa ANSGA-UV L174Bpa -UV | 1 | 0 |
| A137Bpa ANSGA-UV L174Bpa +UV | 0,27205 | 0 |
| A137Bpa ANSGA-UV A254Azi-UV | 0,99654 | 0 |
| A137Bpa ANSGA-UV A254Azi+UV | 5,47E-07 | 1 |
| A137Bpa ANSGA-UV A137Bpa V102A -UV | 1 | 0 |
| A137Bpa ANSGA-UV A137Bpa V102A+UV | 0,99973 | 0 |
| A137Bpa ANSGA-UV L174Bpa V102A-UV | 1 | 0 |
| A137Bpa ANSGA-UV L174Bpa V102A+UV | 1 | 0 |
| A137Bpa ANSGA-UV A254Azi V102A-UV | 3,12E-04 | 1 |
| A137Bpa ANSGA-UV A254Azi V102A+UV | 4,82E-04 | 1 |
| A137Bpa ANSGA-UV A137Bpa H134A-UV | 0,67244 | 0 |
| A137Bpa ANSGA-UV A137Bpa H134A+ UV | 0,76573 | 0 |
| A137Bpa ANSGA-UV L174Bpa H134A-UV | 0,78308 | 0 |
| A137Bpa ANSGA-UV L174Bpa H134A+UV | 0,22892 | 0 |
| A137Bpa ANSGA-UV A254Azi H134A-UV | 1 | 0 |
| A137Bpa ANSGA-UV A254Azi H134A+UV | 1 | 0 |
| A137Bpa ANSGA-UV A137Bpa V181K-UV | 0,82762 | 0 |
| A137Bpa ANSGA-UV A137Bpa V181K+UV | 0,99129 | 0 |
| A137Bpa ANSGA-UV L174Bpa V181K-UV | 0,12217 | 0 |
| A137Bpa ANSGA-UV L174Bpa V181K+UV | 4,95E-05 | 1 |
| A137Bpa ANSGA-UV A254Azi V181K-UV | 1,41E-05 | 1 |
| A137Bpa ANSGA-UV A254Azi V181K+UV | 3,74E-06 | 1 |
| A137Bpa ANSGA-UV A137Bpa P245L-UV | 1 | 0 |
| A137Bpa ANSGA-UV A137Bpa P245L+UV | 0,99787 | 0 |
| A137Bpa ANSGA-UV L174Bpa P245L-UV | 0,99998 | 0 |
| A137Bpa ANSGA-UV L174Bpa P245L+UV | 0,01001 | 1 |
| A137Bpa ANSGA-UV A254Azi P245L-UV | 1 | 0 |
| A137Bpa ANSGA-UV A254Azi P245L+UV | 9,05E-05 | 1 |
| A137Bpa ANSGA +UV A137Bpa - UV | 0,25219 | 0 |
| A137Bpa ANSGA +UV A137Bpa+ UV | 0,01248 | 1 |
| A137Bpa ANSGA +UV L174Bpa -UV | 0 | 1 |
| A137Bpa ANSGA +UV L174Bpa +UV | 1 | 0 |
| A137Bpa ANSGA +UV A254Azi-UV | 1,77E-07 | 1 |
| A137Bpa ANSGA +UV A254Azi+UV | 9,73E-06 | 1 |
| A137Bpa ANSGA +UV A137Bpa V102A -UV | 0 | 1 |
| A137Bpa ANSGA +UV A137Bpa V102A+UV | 0 | 1 |
| A137Bpa ANSGA +UV L174Bpa V102A-UV | 4,91E-04 | 1 |
| A137Bpa ANSGA +UV L174Bpa V102A+UV | 4,29E-04 | 1 |
| A137Bpa ANSGA +UV A254Azi V102A-UV | 0,97858 | 0 |

| | | |
|---|---|---|
| A137Bpa ANSGA +UV A254Azi V102A+UV | 0,95529 | 0 |
| A137Bpa ANSGA +UV A137Bpa H134A-UV | 0 | 1 |
| A137Bpa ANSGA +UV A137Bpa H134A+ UV | 0 | 1 |
| A137Bpa ANSGA +UV L174Bpa H134A-UV | 6,69E-04 | 1 |
| A137Bpa ANSGA +UV L174Bpa H134A+UV | 0,00259 | 1 |
| A137Bpa ANSGA +UV A254Azi H134A-UV | 0 | 1 |
| A137Bpa ANSGA +UV A254Azi H134A+UV | 0 | 1 |
| A137Bpa ANSGA +UV A137Bpa V181K-UV | 0 | 1 |
| A137Bpa ANSGA +UV A137Bpa V181K+UV | 0 | 1 |
| A137Bpa ANSGA +UV L174Bpa V181K-UV | 0,23507 | 0 |
| A137Bpa ANSGA +UV L174Bpa V181K+UV | 0,99999 | 0 |
| A137Bpa ANSGA +UV A254Azi V181K-UV | 1 | 0 |
| A137Bpa ANSGA +UV A254Azi V181K+UV | 1 | 0 |
| A137Bpa ANSGA +UV A137Bpa P245L-UV | 0 | 1 |
| A137Bpa ANSGA +UV A137Bpa P245L+UV | 5,32E-06 | 1 |
| A137Bpa ANSGA +UV L174Bpa P245L-UV | 1,02E-06 | 1 |
| A137Bpa ANSGA +UV L174Bpa P245L+UV | 0,06892 | 0 |
| A137Bpa ANSGA +UV A254Azi P245L-UV | 0 | 1 |
| A137Bpa ANSGA +UV A254Azi P245L+UV | 0,99987 | 0 |
| A137Bpa ANSGA +UV A137Bpa ANSGA-UV | 0 | 1 |
| L174Bpa ANSGA-UV A137Bpa - UV | 0,07101 | 0 |
| L174Bpa ANSGA-UV A137Bpa+ UV | 2,69E-07 | 1 |
| L174Bpa ANSGA-UV L174Bpa -UV | 1 | 0 |
| L174Bpa ANSGA-UV L174Bpa +UV | 0,20403 | 0 |
| L174Bpa ANSGA-UV A254Azi-UV | 0,7422 | 0 |
| L174Bpa ANSGA-UV A254Azi+UV | 7,46E-07 | 1 |
| L174Bpa ANSGA-UV A137Bpa V102A -UV | 1 | 0 |
| L174Bpa ANSGA-UV A137Bpa V102A+UV | 1 | 0 |
| L174Bpa ANSGA-UV L174Bpa V102A-UV | 0,99978 | 0 |
| L174Bpa ANSGA-UV L174Bpa V102A+UV | 0,99978 | 0 |
| L174Bpa ANSGA-UV A254Azi V102A-UV | 1,19E-04 | 1 |
| L174Bpa ANSGA-UV A254Azi V102A+UV | 1,81E-04 | 1 |
| L174Bpa ANSGA-UV A137Bpa H134A-UV | 0,59667 | 0 |
| L174Bpa ANSGA-UV A137Bpa H134A+ UV | 0,75015 | 0 |
| L174Bpa ANSGA-UV L174Bpa H134A-UV | 0,49938 | 0 |
| L174Bpa ANSGA-UV L174Bpa H134A+UV | 0,08729 | 0 |
| L174Bpa ANSGA-UV A254Azi H134A-UV | 1 | 0 |
| L174Bpa ANSGA-UV A254Azi H134A+UV | 1 | 0 |
| L174Bpa ANSGA-UV A137Bpa V181K-UV | 0,85173 | 0 |
| L174Bpa ANSGA-UV A137Bpa V181K+UV | 0,99978 | 0 |
| L174Bpa ANSGA-UV L174Bpa V181K-UV | 0,05817 | 0 |
| L174Bpa ANSGA-UV L174Bpa V181K+UV | 2,43E-05 | 1 |
| L174Bpa ANSGA-UV A254Azi V181K-UV | 4,14E-06 | 1 |
| L174Bpa ANSGA-UV A254Azi V181K+UV | 1,22E-06 | 1 |
| L174Bpa ANSGA-UV A137Bpa P245L-UV | 1 | 0 |
| L174Bpa ANSGA-UV A137Bpa P245L+UV | 0,93496 | 0 |
| L174Bpa ANSGA-UV L174Bpa P245L-UV | 0,99217 | 0 |
| L174Bpa ANSGA-UV L174Bpa P245L+UV | 0,00273 | 1 |
| L174Bpa ANSGA-UV A254Azi P245L-UV | 1 | 0 |
| L174Bpa ANSGA-UV A254Azi P245L+UV | 3,88E-05 | 1 |
| L174Bpa ANSGA-UV A137Bpa ANSGA-UV | 1 | 0 |
| L174Bpa ANSGA-UV A137Bpa ANSGA +UV | 0 | 1 |
| L174Bpa ANSGA+UV A137Bpa - UV | 1 | 0 |
| L174Bpa ANSGA+UV A137Bpa+ UV | 8,07E-06 | 1 |
| L174Bpa ANSGA+UV L174Bpa -UV | 0,20455 | 0 |
| L174Bpa ANSGA+UV L174Bpa +UV | 0,97732 | 0 |
| L174Bpa ANSGA+UV A254Azi-UV | 0,95828 | 0 |
| L174Bpa ANSGA+UV A254Azi+UV | 0 | 1 |
| L174Bpa ANSGA+UV A137Bpa V102A -UV | 0,30062 | 0 |
| L174Bpa ANSGA+UV A137Bpa V102A+UV | 0,01536 | 1 |
| L174Bpa ANSGA+UV L174Bpa V102A-UV | 1 | 0 |
| L174Bpa ANSGA+UV L174Bpa V102A+UV | 1 | 0 |
| L174Bpa ANSGA+UV A254Azi V102A-UV | 0,58934 | 0 |
| L174Bpa ANSGA+UV A254Azi V102A+UV | 0,68535 | 0 |
| L174Bpa ANSGA+UV A137Bpa H134A-UV | 0,00379 | 1 |
| L174Bpa ANSGA+UV A137Bpa H134A+ UV | 0,00451 | 1 |
| L174Bpa ANSGA+UV L174Bpa H134A-UV | 1 | 0 |
| L174Bpa ANSGA+UV L174Bpa H134A+UV | 1 | 0 |
| L174Bpa ANSGA+UV A254Azi H134A-UV | 0,07172 | 0 |
| L174Bpa ANSGA+UV A254Azi H134A+UV | 0,02899 | 1 |
| L174Bpa ANSGA+UV A137Bpa V181K-UV | 0,00508 | 1 |
| L174Bpa ANSGA+UV A137Bpa V181K+UV | 0,01063 | 1 |
| L174Bpa ANSGA+UV L174Bpa V181K-UV | 1 | 0 |
| L174Bpa ANSGA+UV L174Bpa V181K+UV | 0,15251 | 0 |
| L174Bpa ANSGA+UV A254Azi V181K-UV | 0,15794 | 0 |
| L174Bpa ANSGA+UV A254Azi V181K+UV | 0,03933 | 1 |
| L174Bpa ANSGA+UV A137Bpa P245L-UV | 0,07762 | 0 |
| L174Bpa ANSGA+UV A137Bpa P245L+UV | 0,9999 | 0 |
| L174Bpa ANSGA+UV L174Bpa P245L-UV | 0,99421 | 0 |
| L174Bpa ANSGA+UV L174Bpa P245L+UV | 0,99999 | 0 |
| L174Bpa ANSGA+UV A254Azi P245L-UV | 0,02559 | 1 |
| L174Bpa ANSGA+UV A254Azi P245L+UV | 0,26435 | 0 |
| L174Bpa ANSGA+UV A137Bpa ANSGA-UV | 0,15007 | 0 |
| L174Bpa ANSGA+UV A137Bpa ANSGA +UV | 4,90E-04 | 1 |
| L174Bpa ANSGA+UV L174Bpa ANSGA-UV | 0,03993 | 1 |
| A254Azi ANSGA-UV A137Bpa - UV | 1 | 0 |
| A254Azi ANSGA-UV A137Bpa+ UV | 0,03859 | 1 |
| A254Azi ANSGA-UV L174Bpa -UV | 0,68615 | 0 |
| A254Azi ANSGA-UV L174Bpa +UV | 1 | 0 |
| A254Azi ANSGA-UV A254Azi-UV | 0,91146 | 0 |
| A254Azi ANSGA-UV A254Azi+UV | 0,00167 | 1 |
| A254Azi ANSGA-UV A137Bpa V102A -UV | 0,74663 | 0 |
| A254Azi ANSGA-UV A137Bpa V102A+UV | 0,54756 | 0 |
| A254Azi ANSGA-UV L174Bpa V102A-UV | 0,97559 | 0 |
| A254Azi ANSGA-UV L174Bpa V102A+UV | 0,97437 | 0 |
| A254Azi ANSGA-UV A254Azi V102A-UV | 1 | 0 |
| A254Azi ANSGA-UV A254Azi V102A+UV | 1 | 0 |
| A254Azi ANSGA-UV A137Bpa H134A-UV | 0,4516 | 0 |
| A254Azi ANSGA-UV A137Bpa H134A+ UV | 0,46329 | 0 |
| A254Azi ANSGA-UV L174Bpa H134A-UV | 0,99797 | 0 |
| A254Azi ANSGA-UV L174Bpa H134A+UV | 0,99988 | 0 |
| A254Azi ANSGA-UV A254Azi H134A-UV | 0,65381 | 0 |
| A254Azi ANSGA-UV A254Azi H134A+UV | 0,59132 | 0 |
| A254Azi ANSGA-UV A137Bpa V181K-UV | 0,47133 | 0 |
| A254Azi ANSGA-UV A137Bpa V181K+UV | 0,52366 | 0 |
| A254Azi ANSGA-UV L174Bpa V181K-UV | 1 | 0 |
| A254Azi ANSGA-UV L174Bpa V181K+UV | 1 | 0 |
| A254Azi ANSGA-UV A254Azi V181K-UV | 1 | 0 |
| A254Azi ANSGA-UV A254Azi V181K+UV | 1 | 0 |
| A254Azi ANSGA-UV A137Bpa P245L-UV | 0,62616 | 0 |
| A254Azi ANSGA-UV A137Bpa P245L+UV | 0,96183 | 0 |
| A254Azi ANSGA-UV L174Bpa P245L-UV | 0,92649 | 0 |
| A254Azi ANSGA-UV L174Bpa P245L+UV | 1 | 0 |
| A254Azi ANSGA-UV A254Azi P245L-UV | 0,58415 | 0 |
| A254Azi ANSGA-UV A254Azi P245L+UV | 1 | 0 |
| A254Azi ANSGA-UV A137Bpa ANSGA-UV | 0,69502 | 0 |
| A254Azi ANSGA-UV A137Bpa ANSGA +UV | 1 | 0 |

| | | | | | | |
|---|---|---|---|---|---|---|
| | | | | A254Azi ANSGA-UV L174Bpa ANSGA-UV | 0,61043 | 0 |
| | | | | A254Azi ANSGA-UV L174Bpa ANSGA+UV | 0,99959 | 0 |
| | | | | A254Azi ANSGA+UV A137Bpa - UV | 0,00502 | 1 |
| | | | | A254Azi ANSGA+UV A137Bpa+ UV | 1 | 0 |
| | | | | A254Azi ANSGA+UV L174Bpa -UV | 1,92E-04 | 1 |
| | | | | A254Azi ANSGA+UV L174Bpa +UV | 0,13377 | 0 |
| | | | | A254Azi ANSGA+UV A254Azi-UV | 4,26E-04 | 1 |
| | | | | A254Azi ANSGA+UV A254Azi+UV | 1 | 0 |
| | | | | A254Azi ANSGA+UV A137Bpa V102A -UV | 2,40E-04 | 1 |
| | | | | A254Azi ANSGA+UV A137Bpa V102A+UV | 1,52E-04 | 1 |
| | | | | A254Azi ANSGA+UV L174Bpa V102A-UV | 4,96E-04 | 1 |
| | | | | A254Azi ANSGA+UV L174Bpa V102A+UV | 4,90E-04 | 1 |
| | | | | A254Azi ANSGA+UV A254Azi V102A-UV | 0,02179 | 1 |
| | | | | A254Azi ANSGA+UV A254Azi V102A+UV | 0,01926 | 1 |
| | | | | A254Azi ANSGA+UV A137Bpa H134A-UV | 1,18E-04 | 1 |
| | | | | A254Azi ANSGA+UV A137Bpa H134A+ UV | 1,22E-04 | 1 |
| | | | | A254Azi ANSGA+UV L174Bpa H134A-UV | 0,00108 | 1 |
| | | | | A254Azi ANSGA+UV L174Bpa H134A+UV | 0,00179 | 1 |
| | | | | A254Azi ANSGA+UV A254Azi H134A-UV | 1,97E-04 | 1 |
| | | | | A254Azi ANSGA+UV A254Azi H134A+UV | 1,69E-04 | 1 |
| | | | | A254Azi ANSGA+UV A137Bpa V181K-UV | 1,25E-04 | 1 |
| | | | | A254Azi ANSGA+UV A137Bpa V181K+UV | 1,44E-04 | 1 |
| | | | | A254Azi ANSGA+UV L174Bpa V181K-UV | 0,00503 | 1 |
| | | | | A254Azi ANSGA+UV L174Bpa V181K+UV | 0,04377 | 1 |
| | | | | A254Azi ANSGA+UV A254Azi V181K-UV | 0,05432 | 0 |
| | | | | A254Azi ANSGA+UV A254Azi V181K+UV | 0,10886 | 0 |
| | | | | A254Azi ANSGA+UV A137Bpa P245L-UV | 1,71E-04 | 1 |
| | | | | A254Azi ANSGA+UV A137Bpa P245L+UV | 5,18E-04 | 1 |
| | | | | A254Azi ANSGA+UV L174Bpa P245L-UV | 4,07E-04 | 1 |
| | | | | A254Azi ANSGA+UV L174Bpa P245L+UV | 0,00453 | 1 |
| | | | | A254Azi ANSGA+UV A254Azi P245L-UV | 1,67E-04 | 1 |
| | | | | A254Azi ANSGA+UV A254Azi P245L+UV | 0,0365 | 1 |
| | | | | A254Azi ANSGA+UV A137Bpa ANSGA-UV | 2,12E-04 | 1 |
| | | | | A254Azi ANSGA+UV A137Bpa ANSGA +UV | 0,12753 | 0 |
| | | | | A254Azi ANSGA+UV L174Bpa ANSGA-UV | 1,76E-04 | 1 |
| | | | | A254Azi ANSGA+UV L174Bpa ANSGA+UV | 0,00153 | 1 |
| | | | | A254Azi ANSGA+UV A254Azi ANSGA-UV | 0,12261 | 0 |
| 8b | Welch-ANOVA | 0 | F(9, 38.52)=62,85 | A137Bpa WT | 0,8885 | 0 |
| | | | | A137Bpa+S1-UV WT | 0,20317 | 0 |
| | | | | A137Bpa+S1-UV A137Bpa | 0,14833 | 0 |
| | | | | A137Bpa+S1+UV WT | 0,04162 | 1 |
| | | | | A137Bpa+S1+UV A137Bpa | 0,00172 | 1 |
| | | | | A137Bpa+S1+UV A137Bpa+S1-UV | 1,33E-04 | 1 |
| | | | | L174Bpa WT | 8,82E-05 | 1 |
| | | | | L174Bpa A137Bpa | 1,33E-08 | 1 |
| | | | | L174Bpa A137Bpa+S1-UV | 2,05E-06 | 1 |
| | | | | L174Bpa A137Bpa+S1+UV | 2,59E-06 | 1 |
| | | | | L174Bpa+S1-UV WT | 0,65268 | 0 |
| | | | | L174Bpa+S1-UV A137Bpa | 0,96232 | 0 |
| | | | | L174Bpa+S1-UV A137Bpa+S1-UV | 0,99888 | 0 |
| | | | | L174Bpa+S1-UV A137Bpa+S1+UV | 5,50E-04 | 1 |
| | | | | L174Bpa+S1-UV L174Bpa | 8,46E-04 | 1 |
| | | | | L174Bpa+S1+UV WT | 1 | 0 |
| | | | | L174Bpa+S1+UV A137Bpa | 0,77059 | 0 |
| | | | | L174Bpa+S1+UV A137Bpa+S1-UV | 0,15352 | 0 |
| | | | | L174Bpa+S1+UV A137Bpa+S1+UV | 0,06845 | 0 |
| | | | | L174Bpa+S1+UV L174Bpa | 2,26E-04 | 1 |
| | | | | L174Bpa+S1+UV L174Bpa+S1-UV | 0,5293 | 0 |
| | | | | A254Azi WT | 0,00617 | 1 |
| | | | | A254Azi A137Bpa | 0,00868 | 1 |
| | | | | A254Azi A137Bpa+S1-UV | 0,23418 | 0 |
| | | | | A254Azi A137Bpa+S1+UV | 3,18E-06 | 1 |
| | | | | A254Azi L174Bpa | 0,40665 | 0 |
| | | | | A254Azi L174Bpa+S1-UV | 0,17156 | 0 |
| | | | | A254Azi L174Bpa+S1+UV | 0,00517 | 1 |
| | | | | A254Azi+S1-UV WT | 0,75988 | 0 |
| | | | | A254Azi+S1-UV A137Bpa | 0,29727 | 0 |
| | | | | A254Azi+S1-UV A137Bpa+S1-UV | 0,10194 | 0 |
| | | | | A254Azi+S1-UV A137Bpa+S1+UV | 0,99942 | 0 |
| | | | | A254Azi+S1-UV L174Bpa | 0,00335 | 1 |
| | | | | A254Azi+S1-UV L174Bpa+S1-UV | 0,18572 | 0 |
| | | | | A254Azi+S1-UV L174Bpa+S1+UV | 0,82625 | 0 |
| | | | | A254Azi+S1-UV A254Azi | 0,01341 | 1 |
| | | | | A254Azi+S1+UV WT | 0,80536 | 0 |
| | | | | A254Azi+S1+UV A137Bpa | 0,04462 | 1 |
| | | | | A254Azi+S1+UV A137Bpa+S1-UV | 0,0029 | 1 |
| | | | | A254Azi+S1+UV A137Bpa+S1+UV | 0,51662 | 0 |
| | | | | A254Azi+S1+UV L174Bpa | 6,17E-06 | 1 |
| | | | | A254Azi+S1+UV L174Bpa+S1-UV | 0,0278 | 1 |
| | | | | A254Azi+S1+UV L174Bpa+S1+UV | 0,89913 | 0 |
| | | | | A254Azi+S1+UV A254Azi | 8,34E-05 | 1 |
| | | | | A254Azi+S1+UV A254Azi+S1-UV | 0,99867 | 0 |
| 8d | Welch-ANOVA | 3,54E-07 | F(9, 23.63)=12.79 | A137Bpa WT | 1 | 0 |
| | | | | A137Bpa+S1-UV WT | 0,37152 | 0 |
| | | | | A137Bpa+S1-UV A137Bpa | 0,35916 | 0 |
| | | | | A137Bpa+S1+UV WT | 0,08956 | 0 |
| | | | | A137Bpa+S1+UV A137Bpa | 0,07984 | 0 |
| | | | | A137Bpa+S1+UV A137Bpa+S1-UV | 0,99501 | 0 |
| | | | | L174Bpa WT | 0,10286 | 0 |
| | | | | L174Bpa A137Bpa | 0,10398 | 0 |
| | | | | L174Bpa A137Bpa+S1-UV | 0,0336 | 1 |
| | | | | L174Bpa A137Bpa+S1+UV | 0,04955 | 1 |
| | | | | L174Bpa+S1-UV WT | 0,99391 | 0 |
| | | | | L174Bpa+S1-UV A137Bpa | 0,98861 | 0 |
| | | | | L174Bpa+S1-UV A137Bpa+S1-UV | 0,61204 | 0 |
| | | | | L174Bpa+S1-UV A137Bpa+S1+UV | 0,56872 | 0 |
| | | | | L174Bpa+S1-UV L174Bpa | 0,08557 | 0 |
| | | | | L174Bpa+S1+UV WT | 0,41774 | 0 |
| | | | | L174Bpa+S1+UV A137Bpa | 0,40688 | 0 |
| | | | | L174Bpa+S1+UV A137Bpa+S1-UV | 0,15141 | 0 |
| | | | | L174Bpa+S1+UV A137Bpa+S1+UV | 0,00877 | 1 |
| | | | | L174Bpa+S1+UV L174Bpa | 0,15339 | 0 |
| | | | | L174Bpa+S1+UV L174Bpa+S1-UV | 0,37336 | 0 |
| | | | | A254Azi WT | 0,00284 | 1 |
| | | | | A254Azi A137Bpa | 0,00291 | 1 |
| | | | | A254Azi A137Bpa+S1-UV | 4,88E-04 | 1 |
| | | | | A254Azi A137Bpa+S1+UV | 0,00127 | 1 |
| | | | | A254Azi L174Bpa | 0,82509 | 0 |
| | | | | A254Azi L174Bpa+S1-UV | 0,00205 | 1 |
| | | | | A254Azi L174Bpa+S1+UV | 0,00397 | 1 |
| | | | | A254Azi+S1-UV WT | 0,05734 | 0 |
| | | | | A254Azi+S1-UV A137Bpa | 0,05762 | 0 |
| | | | | A254Azi+S1-UV A137Bpa+S1-UV | 0,03628 | 1 |

| | | | | | | |
|---|---|---|---|---|---|---|
| | | | | A254Azi+S1-UV A137Bpa+S1+UV | 0,04349 | 1 |
| | | | | A254Azi+S1-UV L174Bpa | 0,38514 | 0 |
| | | | | A254Azi+S1-UV L174Bpa+S1-UV | 0,05308 | 0 |
| | | | | A254Azi+S1-UV L174Bpa+S1+UV | 0,06642 | 0 |
| | | | | A254Azi+S1-UV A254Azi | 0,80961 | 0 |
| | | | | A254Azi+S1+UV WT | 0,06518 | 0 |
| | | | | A254Azi+S1+UV A137Bpa | 0,06595 | 0 |
| | | | | A254Azi+S1+UV A137Bpa+S1-UV | 0,02106 | 1 |
| | | | | A254Azi+S1+UV A137Bpa+S1+UV | 0,03299 | 1 |
| | | | | A254Azi+S1+UV L174Bpa | 1 | 0 |
| | | | | A254Azi+S1+UV L174Bpa+S1-UV | 0,05401 | 0 |
| | | | | A254Azi+S1+UV L174Bpa+S1+UV | 0,09412 | 0 |
| | | | | A254Azi+S1+UV A254Azi | 0,91204 | 0 |
| | | | | A254Azi+S1+UV A254Azi+S1-UV | 0,445 | 0 |
| 8f | Welch-ANOVA | 1,27E-05 | F(7, 15.82)=13.41 | WTB WT E | 0,9519 | 0 |
| | | | | 137 E WT E | 0,15884 | 0 |
| | | | | 137 E WTB | 0,69871 | 0 |
| | | | | 137 B WT E | 0,99748 | 0 |
| | | | | 137 B WTB | 0,99884 | 0 |
| | | | | 137 B 137 E | 0,30689 | 0 |
| | | | | WT Na E WT E | 0,01099 | 1 |
| | | | | WT Na E WTB | 0,18692 | 0 |
| | | | | WT Na E 137 E | 0,93575 | 0 |
| | | | | WT Na E 137 B | 0,03036 | 1 |
| | | | | WT Na B WT E | 0,00225 | 1 |
| | | | | WT Na B WTB | 0,05663 | 0 |
| | | | | WT Na B 137 E | 0,31372 | 0 |
| | | | | WT Na B 137 B | 0,008 | 1 |
| | | | | WT Na B WT Na E | 0,27488 | 0 |
| | | | | 137 Na E WT E | 0,12882 | 0 |
| | | | | 137 Na E WTB | 0,52746 | 0 |
| | | | | 137 Na E 137 E | 0,99963 | 0 |
| | | | | 137 Na E 137 B | 0,23408 | 0 |
| | | | | 137 Na E WT Na E | 0,99965 | 0 |
| | | | | 137 Na E WT Na B | 0,69888 | 0 |
| | | | | 137 Na B WT E | 0,00117 | 1 |
| | | | | 137 Na B WTB | 0,03385 | 1 |
| | | | | 137 Na B 137 E | 0,15178 | 0 |
| | | | | 137 Na B 137 B | 0,00456 | 1 |
| | | | | 137 Na B WT Na E | 0,0596 | 0 |
| | | | | 137 Na B WT Na B | 0,88042 | 0 |
| | | | | 137 Na B 137 Na E | 0,43898 | 0 |
| 8h | Welch-ANOVA | 2,20E-07 | F(7, 12.19)=39.98 | WT+CM4620 WT | 0,00174 | 1 |
| | | | | A137Bpa WT | 0,88806 | 0 |
| | | | | A137Bpa WT+CM4620 | 0,01933 | 1 |
| | | | | A137Bpa+CM4620 WT | 3,35E-04 | 1 |
| | | | | A137Bpa+CM4620 WT+CM4620 | 0,28743 | 0 |
| | | | | A137Bpa+CM4620 A137Bpa | 0,03808 | 1 |
| | | | | L174Bpa WT | 0,00237 | 1 |
| | | | | L174Bpa WT+CM4620 | 0,02662 | 1 |
| | | | | L174Bpa A137Bpa | 0,08042 | 0 |
| | | | | L174Bpa A137Bpa+CM4620 | 0,68854 | 0 |
| | | | | L174Bpa+CM4620 WT | 0,00283 | 1 |
| | | | | L174Bpa+CM4620 WT+CM4620 | 0,9493 | 0 |
| | | | | L174Bpa+CM4620 A137Bpa | 0,01796 | 1 |
| | | | | L174Bpa+CM4620 A137Bpa+CM4620 | 0,1934 | 0 |
| | | | | L174Bpa+CM4620 L174Bpa | 0,02258 | 1 |
| | | | | A254Azi WT | 0,07848 | 0 |
| | | | | A254Azi WT+CM4620 | 0,02604 | 1 |
| | | | | A254Azi A137Bpa | 0,11453 | 0 |
| | | | | A254Azi A137Bpa+CM4620 | 0,02809 | 1 |
| | | | | A254Azi L174Bpa | 0,03427 | 1 |
| | | | | A254Azi L174Bpa+CM4620 | 0,02569 | 1 |
| | | | | A254Azi+CM4620 WT | 5,02E-04 | 1 |
| | | | | A254Azi+CM4620 WT+CM4620 | 0,99587 | 0 |
| | | | | A254Azi+CM4620 A137Bpa | 0,02071 | 1 |
| | | | | A254Azi+CM4620 A137Bpa+CM4620 | 0,51937 | 0 |
| | | | | A254Azi+CM4620 L174Bpa | 0,04124 | 1 |
| | | | | A254Azi+CM4620 L174Bpa+CM4620 | 0,87879 | 0 |
| | | | | A254Azi+CM4620 A254Azi | 0,02589 | 1 |
| 9b | Mann-Whitney test | 5,32662E-05 | | A137Bpa after UV-light - A137Bpa from before UV-light | | 1 |
| | | 0,00288 | | A254Azi after UV-light - A254Azi from before UV-light | | 1 |
| | | 0,20804 | | A137Bpa after UV-light A254Azi after UV-light | | 0 |
| 9c | Mann-Whitney test | 0,77172 | | A137Bpa A254Azi | | 0 |
| 9e | Mann-Whitney test | 4,11353E-05 | | A137Bpa after UV-light - A137Bpa from before UV-light | | 1 |
| | | 0,0005828 | | A254Azi after UV-light - A254Azi from before UV-light | | 1 |
| | | 0,0711500 | | A137Bpa after UV-light A254Azi after UV-light | | 0 |
| 9f | Mann-Whitney test | 0,79221 | | A137Bpa A254Azi | | 0 |
| 9h | Mann-Whitney test | 0,18535 | | O1+UV start O1+UV end | | 0 |
| | | 0,80478 | | O1-UV start O1-UV end | | 0 |
| | | 2,72E-06 | | A137Bpa with UV Start-A137Bpa with UV End | | 1 |
| | | 1,46E-01 | | A137Bpa without UV Start-A137Bpa without UV End | | 0 |
| | | 2,33E-08 | | L174Bpa with UV Start-L174Bpa with UV End | | 1 |
| | | 0,01461 | | L174Bpa without UV Start-L174Bpa without UV End | | 1 |
| | | 8,57E-10 | | A254Azi with UV Start-A254Azi with UV End | | 1 |
| | | 0,03788 | | A254Azi without UV Start-A254Azi without UV End | | 1 |
| | | 0,65761 | | O1 + UV end O1-UV end | | 0 |
| | | 0,00588 | | A137Bpa +UV end A137Bpa -UV end | | 1 |
| | | 3,70E-04 | | L174Bpa+UV end L174Bpa-UV end | | 1 |
| | | 0,0025 | | A254Azi+UV end A254Azi-UV end | | 1 |
| 9k | Mann-Whitney test | 0,26567 | | endo start endo end | | 0 |
| | | 0,00245 | | O1 start O1 end | | 1 |
| | | 7,65E-04 | | 137 start 137 end | | 1 |
| | | 6,93E-06 | | 254 start 254 end | | 1 |
| S1a - 0mM and 2mM Ca2+ solution | Welch-ANOVA | 0 | F(41, 371.32)=10.02 | A235Azi 0 A235Azi 2 | 0,99997 | 0 |
| | | | | S239Azi 2 A235Azi 2 | 0,99987 | 0 |
| | | | | S239Azi 2 A235Azi 0 | 1 | 0 |
| | | | | S239Azi 0 A235Azi 2 | 0,84303 | 0 |
| | | | | S239Azi 0 A235Azi 0 | 1 | 0 |
| | | | | S239Azi 0 S239Azi 2 | 1 | 0 |
| | | | | M243Azi 2 A235Azi 2 | 1 | 0 |

| | | | | | | |
|---|---|---|---|---|---|---|
| | | | | M243Azi 2 A235Azi 0 | 0,99911 | 0 |
| | | | | M243Azi 2 S239Azi 2 | 0,99794 | 0 |
| | | | | M243Azi 2 S239Azi 0 | 0,98521 | 0 |
| | | | | M243Azi 0 A235Azi 2 | 0,99919 | 0 |
| | | | | M243Azi 0 A235Azi 0 | 1 | 0 |
| | | | | M243Azi 0 S239Azi 2 | 1 | 0 |
| | | | | M243Azi 0 S239Azi 0 | 1 | 0 |
| | | | | M243Azi 0 M243Azi 2 | 0,99837 | 0 |
| | | | | P245Azi 2 A235Azi 2 | 0,96604 | 0 |
| | | | | P245Azi 2 A235Azi 0 | 0,93933 | 0 |
| | | | | P245Azi 2 S239Azi 2 | 0,92978 | 0 |
| | | | | P245Azi 2 S239Azi 0 | 0,92306 | 0 |
| | | | | P245Azi 2 M243Azi 2 | 0,98964 | 0 |
| | | | | P245Azi 2 M243Azi 0 | 0,93713 | 0 |
| | | | | P245Azi 0 A235Azi 2 | 0,00694 | 1 |
| | | | | P245Azi 0 A235Azi 0 | 0,619 | 0 |
| | | | | P245Azi 0 S239Azi 2 | 0,99995 | 0 |
| | | | | P245Azi 0 S239Azi 0 | 0,77789 | 0 |
| | | | | P245Azi 0 M243Azi 2 | 0,81545 | 0 |
| | | | | P245Azi 0 M243Azi 0 | 0,53537 | 0 |
| | | | | P245Azi 0 P245Azi 2 | 0,88403 | 0 |
| | | | | F250Azi 2 A235Azi 2 | 0,96936 | 0 |
| | | | | F250Azi 2 A235Azi 0 | 0,89803 | 0 |
| | | | | F250Azi 2 S239Azi 2 | 0,8736 | 0 |
| | | | | F250Azi 2 S239Azi 0 | 0,84262 | 0 |
| | | | | F250Azi 2 M243Azi 2 | 0,99942 | 0 |
| | | | | F250Azi 2 M243Azi 0 | 0,89031 | 0 |
| | | | | F250Azi 2 P245Azi 2 | 1 | 0 |
| | | | | F250Azi 2 P245Azi 0 | 0,70668 | 0 |
| | | | | F250Azi 0 A235Azi 2 | 0,92601 | 0 |
| | | | | F250Azi 0 A235Azi 0 | 1 | 0 |
| | | | | F250Azi 0 S239Azi 2 | 1 | 0 |
| | | | | F250Azi 0 S239Azi 0 | 1 | 0 |
| | | | | F250Azi 0 M243Azi 2 | 0,97473 | 0 |
| | | | | F250Azi 0 M243Azi 0 | 1 | 0 |
| | | | | F250Azi 0 P245Azi 2 | 0,9089 | 0 |
| | | | | F250Azi 0 P245Azi 0 | 1 | 0 |
| | | | | F250Azi 0 F250Azi 2 | 0,80371 | 0 |
| | | | | A254Azi 2 A235Azi 2 | 0,76325 | 0 |
| | | | | A254Azi 2 A235Azi 0 | 0,36506 | 0 |
| | | | | A254Azi 2 S239Azi 2 | 0,32535 | 0 |
| | | | | A254Azi 2 S239Azi 0 | 0,20204 | 0 |
| | | | | A254Azi 2 M243Azi 2 | 0,99986 | 0 |
| | | | | A254Azi 2 M243Azi 0 | 0,33263 | 0 |
| | | | | A254Azi 2 P245Azi 2 | 0,99987 | 0 |
| | | | | A254Azi 2 P245Azi 0 | 0,05321 | 0 |
| | | | | A254Azi 2 F250Azi 2 | 1 | 0 |
| | | | | A254Azi 2 F250Azi 0 | 0,17089 | 0 |
| | | | | A254Azi 0 A235Azi 2 | 0,00498 | 1 |
| | | | | A254Azi 0 A235Azi 0 | 0,54733 | 0 |
| | | | | A254Azi 0 S239Azi 2 | 0,99993 | 0 |
| | | | | A254Azi 0 S239Azi 0 | 0,65876 | 0 |
| | | | | A254Azi 0 M243Azi 2 | 0,81068 | 0 |
| | | | | A254Azi 0 M243Azi 0 | 0,47776 | 0 |
| | | | | A254Azi 0 P245Azi 2 | 0,88375 | 0 |
| | | | | A254Azi 0 P245Azi 0 | 1 | 0 |
| | | | | A254Azi 0 F250Azi 2 | 0,7053 | 0 |
| | | | | A254Azi 0 F250Azi 0 | 1 | 0 |
| | | | | A254Azi 0 A254Azi 2 | 0,052 | 0 |
| | | | | L194Azi 2 A235Azi 2 | 0,02266 | 1 |
| | | | | L194Azi 2 A235Azi 0 | 0,83963 | 0 |
| | | | | L194Azi 2 S239Azi 2 | 1 | 0 |
| | | | | L194Azi 2 S239Azi 0 | 0,9719 | 0 |
| | | | | L194Azi 2 M243Azi 2 | 0,84382 | 0 |
| | | | | L194Azi 2 M243Azi 0 | 0,75193 | 0 |
| | | | | L194Azi 2 P245Azi 2 | 0,88759 | 0 |
| | | | | L194Azi 2 P245Azi 0 | 1 | 0 |
| | | | | L194Azi 2 F250Azi 2 | 0,71985 | 0 |
| | | | | L194Azi 2 F250Azi 0 | 1 | 0 |
| | | | | L194Azi 2 A254Azi 2 | 0,06172 | 0 |
| | | | | L194Azi 2 A254Azi 0 | 1 | 0 |
| | | | | L194Azi 0 A235Azi 2 | 0,99207 | 0 |
| | | | | L194Azi 0 A235Azi 0 | 1 | 0 |
| | | | | L194Azi 0 S239Azi 2 | 1 | 0 |
| | | | | L194Azi 0 S239Azi 0 | 1 | 0 |
| | | | | L194Azi 0 M243Azi 2 | 0,99509 | 0 |
| | | | | L194Azi 0 M243Azi 0 | 1 | 0 |
| | | | | L194Azi 0 P245Azi 2 | 0,92959 | 0 |
| | | | | L194Azi 0 P245Azi 0 | 0,91934 | 0 |
| | | | | L194Azi 0 F250Azi 2 | 0,86686 | 0 |
| | | | | L194Azi 0 F250Azi 0 | 1 | 0 |
| | | | | L194Azi 0 A254Azi 2 | 0,26941 | 0 |
| | | | | L194Azi 0 A254Azi 0 | 0,88641 | 0 |
| | | | | L194Azi 0 L194Azi 2 | 0,98431 | 0 |
| | | | | V191Azi 2 A235Azi 2 | 0,14513 | 0 |
| | | | | V191Azi 2 A235Azi 0 | 0,08285 | 0 |
| | | | | V191Azi 2 S239Azi 2 | 0,07222 | 0 |
| | | | | V191Azi 2 S239Azi 0 | 0,06218 | 0 |
| | | | | V191Azi 2 M243Azi 2 | 0,38135 | 0 |
| | | | | V191Azi 2 M243Azi 0 | 0,07932 | 0 |
| | | | | V191Azi 2 P245Azi 2 | 1 | 0 |
| | | | | V191Azi 2 P245Azi 0 | 0,03526 | 1 |
| | | | | V191Azi 2 F250Azi 2 | 1 | 0 |
| | | | | V191Azi 2 F250Azi 0 | 0,05186 | 0 |
| | | | | V191Azi 2 A254Azi 2 | 0,93752 | 0 |
| | | | | V191Azi 2 A254Azi 0 | 0,03508 | 1 |
| | | | | V191Azi 2 L194Azi 2 | 0,03707 | 1 |
| | | | | V191Azi 2 L194Azi 0 | 0,06994 | 0 |
| | | | | V191Azi 0 A235Azi 2 | 1 | 0 |
| | | | | V191Azi 0 A235Azi 0 | 1 | 0 |
| | | | | V191Azi 0 S239Azi 2 | 1 | 0 |
| | | | | V191Azi 0 S239Azi 0 | 1 | 0 |
| | | | | V191Azi 0 M243Azi 2 | 0,99999 | 0 |
| | | | | V191Azi 0 M243Azi 0 | 1 | 0 |
| | | | | V191Azi 0 P245Azi 2 | 0,94857 | 0 |
| | | | | V191Azi 0 P245Azi 0 | 0,96246 | 0 |
| | | | | V191Azi 0 F250Azi 2 | 0,93074 | 0 |
| | | | | V191Azi 0 F250Azi 0 | 1 | 0 |
| | | | | V191Azi 0 A254Azi 2 | 0,56224 | 0 |
| | | | | V191Azi 0 A254Azi 0 | 0,95492 | 0 |
| | | | | V191Azi 0 L194Azi 2 | 0,98572 | 0 |
| | | | | V191Azi 0 L194Azi 0 | 1 | 0 |
| | | | | V191Azi 0 V191Azi 2 | 0,10232 | 0 |
| | | | | L188Azi 2 A235Azi 2 | 0,01842 | 1 |
| | | | | L188Azi 2 A235Azi 0 | 0,00734 | 1 |
| | | | | L188Azi 2 S239Azi 2 | 0,00596 | 1 |
| | | | | L188Azi 2 S239Azi 0 | 0,00465 | 1 |

| | | |
|---|---|---|
| L188Azi 2 M243Azi 2 | 0,1317 | 0 |
| L188Azi 2 M243Azi 0 | 0,00685 | 1 |
| L188Azi 2 P245Azi 2 | 1 | 0 |
| L188Azi 2 P245Azi 0 | 0,00195 | 1 |
| L188Azi 2 F250Azi 2 | 1 | 0 |
| L188Azi 2 F250Azi 0 | 0,00353 | 1 |
| L188Azi 2 A254Azi 2 | 0,85042 | 0 |
| L188Azi 2 A254Azi 0 | 0,00194 | 1 |
| L188Azi 2 L194Azi 2 | 0,0021 | 1 |
| L188Azi 2 L194Azi 0 | 0,00561 | 1 |
| L188Azi 2 V191Azi 2 | 1 | 0 |
| L188Azi 2 V191Azi 0 | 0,01054 | 1 |
| L188Azi 0 A235Azi 2 | 0,08723 | 0 |
| L188Azi 0 A235Azi 0 | 0,98982 | 0 |
| L188Azi 0 S239Azi 2 | 1 | 0 |
| L188Azi 0 S239Azi 0 | 0,99998 | 0 |
| L188Azi 0 M243Azi 2 | 0,91115 | 0 |
| L188Azi 0 M243Azi 0 | 0,96096 | 0 |
| L188Azi 0 P245Azi 2 | 0,90096 | 0 |
| L188Azi 0 P245Azi 0 | 0,99993 | 0 |
| L188Azi 0 F250Azi 2 | 0,76509 | 0 |
| L188Azi 0 F250Azi 0 | 1 | 0 |
| L188Azi 0 A254Azi 2 | 0,0934 | 0 |
| L188Azi 0 A254Azi 0 | 0,99875 | 0 |
| L188Azi 0 L194Azi 2 | 1 | 0 |
| L188Azi 0 L194Azi 0 | 0,99992 | 0 |
| L188Azi 0 V191Azi 2 | 0,04442 | 1 |
| L188Azi 0 V191Azi 0 | 0,9994 | 0 |
| L188Azi 0 L188Azi 2 | 0,00277 | 1 |
| F187Azi 2 A235Azi 2 | 0,01935 | 1 |
| F187Azi 2 A235Azi 0 | 0,80998 | 0 |
| F187Azi 2 S239Azi 2 | 0,99999 | 0 |
| F187Azi 2 S239Azi 0 | 0,961 | 0 |
| F187Azi 2 M243Azi 2 | 0,83411 | 0 |
| F187Azi 2 M243Azi 0 | 0,72292 | 0 |
| F187Azi 2 P245Azi 2 | 0,88574 | 0 |
| F187Azi 2 P245Azi 0 | 1 | 0 |
| F187Azi 2 F250Azi 2 | 0,71374 | 0 |
| F187Azi 2 F250Azi 0 | 1 | 0 |
| F187Azi 2 A254Azi 2 | 0,05849 | 0 |
| F187Azi 2 A254Azi 0 | 1 | 0 |
| F187Azi 2 L194Azi 2 | 1 | 0 |
| F187Azi 2 L194Azi 0 | 0,97817 | 0 |
| F187Azi 2 V191Azi 2 | 0,03619 | 1 |
| F187Azi 2 V191Azi 0 | 0,98131 | 0 |
| F187Azi 2 L188Azi 2 | 0,00203 | 1 |
| F187Azi 2 L188Azi 0 | 1 | 0 |
| F187Azi 0 A235Azi 2 | 0,01438 | 1 |
| F187Azi 0 A235Azi 0 | 0,79023 | 0 |
| F187Azi 0 S239Azi 2 | 1 | 0 |
| F187Azi 0 S239Azi 0 | 0,9355 | 0 |
| F187Azi 0 M243Azi 2 | 0,84932 | 0 |
| F187Azi 0 M243Azi 0 | 0,68149 | 0 |
| F187Azi 0 P245Azi 2 | 0,88983 | 0 |
| F187Azi 0 P245Azi 0 | 1 | 0 |
| F187Azi 0 F250Azi 2 | 0,72627 | 0 |
| F187Azi 0 F250Azi 0 | 1 | 0 |
| F187Azi 0 A254Azi 2 | 0,06389 | 0 |
| F187Azi 0 A254Azi 0 | 1 | 0 |
| F187Azi 0 L194Azi 2 | 1 | 0 |
| F187Azi 0 L194Azi 0 | 0,97571 | 0 |
| F187Azi 0 V191Azi 2 | 0,03806 | 1 |
| F187Azi 0 V191Azi 0 | 0,98544 | 0 |
| F187Azi 0 L188Azi 2 | 0,00219 | 1 |
| F187Azi 0 L188Azi 0 | 1 | 0 |
| F187Azi 0 F187Azi 2 | 1 | 0 |
| L185Azi 2 A235Azi 2 | 0,54093 | 0 |
| L185Azi 2 A235Azi 0 | 1 | 0 |
| L185Azi 2 S239Azi 2 | 1 | 0 |
| L185Azi 2 S239Azi 0 | 1 | 0 |
| L185Azi 2 M243Azi 2 | 0,97015 | 0 |
| L185Azi 2 M243Azi 0 | 1 | 0 |
| L185Azi 2 P245Azi 2 | 0,91649 | 0 |
| L185Azi 2 P245Azi 0 | 0,80735 | 0 |
| L185Azi 2 F250Azi 2 | 0,81946 | 0 |
| L185Azi 2 F250Azi 0 | 1 | 0 |
| L185Azi 2 A254Azi 2 | 0,1592 | 0 |
| L185Azi 2 A254Azi 0 | 0,69387 | 0 |
| L185Azi 2 L194Azi 2 | 0,98375 | 0 |
| L185Azi 2 L194Azi 0 | 1 | 0 |
| L185Azi 2 V191Azi 2 | 0,05595 | 0 |
| L185Azi 2 V191Azi 0 | 1 | 0 |
| L185Azi 2 L188Azi 2 | 0,00395 | 1 |
| L185Azi 2 L188Azi 0 | 1 | 0 |
| L185Azi 2 F187Azi 2 | 0,97748 | 0 |
| L185Azi 2 F187Azi 0 | 0,93914 | 0 |
| L185Azi 0 A235Azi 2 | 0,8033 | 0 |
| L185Azi 0 A235Azi 0 | 1 | 0 |
| L185Azi 0 S239Azi 2 | 1 | 0 |
| L185Azi 0 S239Azi 0 | 1 | 0 |
| L185Azi 0 M243Azi 2 | 0,97116 | 0 |
| L185Azi 0 M243Azi 0 | 1 | 0 |
| L185Azi 0 P245Azi 2 | 0,91201 | 0 |
| L185Azi 0 P245Azi 0 | 0,99995 | 0 |
| L185Azi 0 F250Azi 2 | 0,80928 | 0 |
| L185Azi 0 F250Azi 0 | 1 | 0 |
| L185Azi 0 A254Azi 2 | 0,163 | 0 |
| L185Azi 0 A254Azi 0 | 0,99989 | 0 |
| L185Azi 0 L194Azi 2 | 1 | 0 |
| L185Azi 0 L194Azi 0 | 1 | 0 |
| L185Azi 0 V191Azi 2 | 0,05333 | 0 |
| L185Azi 0 V191Azi 0 | 1 | 0 |
| L185Azi 0 L188Azi 2 | 0,00369 | 1 |
| L185Azi 0 L188Azi 0 | 1 | 0 |
| L185Azi 0 F187Azi 2 | 1 | 0 |
| L185Azi 0 F187Azi 0 | 1 | 0 |
| L185Azi 0 L185Azi 2 | 1 | 0 |
| V181Azi 2 A235Azi 2 | 1 | 0 |
| V181Azi 2 A235Azi 0 | 1 | 0 |
| V181Azi 2 S239Azi 2 | 0,99996 | 0 |
| V181Azi 2 S239Azi 0 | 0,99629 | 0 |
| V181Azi 2 M243Azi 2 | 1 | 0 |
| V181Azi 2 M243Azi 0 | 0,99998 | 0 |
| V181Azi 2 P245Azi 2 | 0,97771 | 0 |
| V181Azi 2 P245Azi 0 | 0,66442 | 0 |
| V181Azi 2 F250Azi 2 | 0,9915 | 0 |
| V181Azi 2 F250Azi 0 | 0,99149 | 0 |

| | | | | | |
|---|---|---|---|---|---|
| | | | V181Azi 2 A254Azi 2 | 0,9754 | 0 |
| | | | V181Azi 2 A254Azi 0 | 0,64951 | 0 |
| | | | V181Azi 2 L194Azi 2 | 0,73861 | 0 |
| | | | V181Azi 2 L194Azi 0 | 0,99969 | 0 |
| | | | V181Azi 2 V191Azi 2 | 0,21791 | 0 |
| | | | V181Azi 2 V191Azi 0 | 1 | 0 |
| | | | V181Azi 2 L188Azi 2 | 0,0399 | 1 |
| | | | V181Azi 2 L188Azi 0 | 0,88535 | 0 |
| | | | V181Azi 2 F187Azi 2 | 0,71764 | 0 |
| | | | V181Azi 2 F187Azi 0 | 0,74257 | 0 |
| | | | V181Azi 2 L185Azi 2 | 0,98394 | 0 |
| | | | V181Azi 2 L185Azi 0 | 0,98672 | 0 |
| | | | V181Azi 0 A235Azi 2 | 0,01889 | 1 |
| | | | V181Azi 0 A235Azi 0 | 0,84932 | 0 |
| | | | V181Azi 0 S239Azi 2 | 1 | 0 |
| | | | V181Azi 0 S239Azi 0 | 0,96534 | 0 |
| | | | V181Azi 0 M243Azi 2 | 0,86666 | 0 |
| | | | V181Azi 0 M243Azi 0 | 0,73529 | 0 |
| | | | V181Azi 0 P245Azi 2 | 0,89326 | 0 |
| | | | V181Azi 0 P245Azi 0 | 1 | 0 |
| | | | V181Azi 0 F250Azi 2 | 0,73765 | 0 |
| | | | V181Azi 0 F250Azi 0 | 1 | 0 |
| | | | V181Azi 0 A254Azi 2 | 0,07063 | 0 |
| | | | V181Azi 0 A254Azi 0 | 0,9999 | 0 |
| | | | V181Azi 0 L194Azi 2 | 1 | 0 |
| | | | V181Azi 0 L194Azi 0 | 0,98749 | 0 |
| | | | V181Azi 0 V191Azi 2 | 0,03982 | 1 |
| | | | V181Azi 0 V191Azi 0 | 0,99176 | 0 |
| | | | V181Azi 0 L188Azi 2 | 0,00235 | 1 |
| | | | V181Azi 0 L188Azi 0 | 1 | 0 |
| | | | V181Azi 0 F187Azi 2 | 1 | 0 |
| | | | V181Azi 0 F187Azi 0 | 1 | 0 |
| | | | V181Azi 0 L185Azi 2 | 0,96038 | 0 |
| | | | V181Azi 0 L185Azi 0 | 1 | 0 |
| | | | V181Azi 0 V181Azi 2 | 0,78069 | 0 |
| | | | S179Azi 2 A235Azi 2 | 1 | 0 |
| | | | S179Azi 2 A235Azi 0 | 1 | 0 |
| | | | S179Azi 2 S239Azi 2 | 1 | 0 |
| | | | S179Azi 2 S239Azi 0 | 0,99472 | 0 |
| | | | S179Azi 2 M243Azi 2 | 0,9999 | 0 |
| | | | S179Azi 2 M243Azi 0 | 1 | 0 |
| | | | S179Azi 2 P245Azi 2 | 0,94976 | 0 |
| | | | S179Azi 2 P245Azi 0 | 4,77E-04 | 1 |
| | | | S179Azi 2 F250Azi 2 | 0,92692 | 0 |
| | | | S179Azi 2 F250Azi 0 | 0,99756 | 0 |
| | | | S179Azi 2 A254Azi 2 | 0,46575 | 0 |
| | | | S179Azi 2 A254Azi 0 | 1,43E-04 | 1 |
| | | | S179Azi 2 L194Azi 2 | 0,01134 | 1 |
| | | | S179Azi 2 L194Azi 0 | 1 | 0 |
| | | | S179Azi 2 V191Azi 2 | 0,09962 | 0 |
| | | | S179Azi 2 V191Azi 0 | 1 | 0 |
| | | | S179Azi 2 L188Azi 2 | 0,00985 | 1 |
| | | | S179Azi 2 L188Azi 0 | 0,06226 | 0 |
| | | | S179Azi 2 F187Azi 2 | 0,00896 | 1 |
| | | | S179Azi 2 F187Azi 0 | 0,0015 | 1 |
| | | | S179Azi 2 L185Azi 2 | 0,80121 | 0 |
| | | | S179Azi 2 L185Azi 0 | 0,9677 | 0 |
| | | | S179Azi 2 V181Azi 2 | 1 | 0 |
| | | | S179Azi 2 V181Azi 0 | 0,00179 | 1 |
| | | | S179Azi 0 A235Azi 2 | 0,99531 | 0 |
| | | | S179Azi 0 A235Azi 0 | 1 | 0 |
| | | | S179Azi 0 S239Azi 2 | 1 | 0 |
| | | | S179Azi 0 S239Azi 0 | 1 | 0 |
| | | | S179Azi 0 M243Azi 2 | 0,99734 | 0 |
| | | | S179Azi 0 M243Azi 0 | 1 | 0 |
| | | | S179Azi 0 P245Azi 2 | 0,93599 | 0 |
| | | | S179Azi 0 P245Azi 0 | 0,06917 | 0 |
| | | | S179Azi 0 F250Azi 2 | 0,88499 | 0 |
| | | | S179Azi 0 F250Azi 0 | 1 | 0 |
| | | | S179Azi 0 A254Azi 2 | 0,30356 | 0 |
| | | | S179Azi 0 A254Azi 0 | 0,03308 | 1 |
| | | | S179Azi 0 L194Azi 2 | 0,34349 | 0 |
| | | | S179Azi 0 L194Azi 0 | 1 | 0 |
| | | | S179Azi 0 V191Azi 2 | 0,07707 | 0 |
| | | | S179Azi 0 V191Azi 0 | 1 | 0 |
| | | | S179Azi 0 L188Azi 2 | 0,00653 | 1 |
| | | | S179Azi 0 L188Azi 0 | 0,81458 | 0 |
| | | | S179Azi 0 F187Azi 2 | 0,30838 | 0 |
| | | | S179Azi 0 F187Azi 0 | 0,16343 | 0 |
| | | | S179Azi 0 L185Azi 2 | 0,99997 | 0 |
| | | | S179Azi 0 L185Azi 0 | 0,99999 | 0 |
| | | | S179Azi 0 V181Azi 2 | 0,99992 | 0 |
| | | | S179Azi 0 V181Azi 0 | 0,19761 | 0 |
| | | | S179Azi 0 S179Azi 2 | 1 | 0 |
| | | | W176Azi 2 A235Azi 2 | 0,4909 | 0 |
| | | | W176Azi 2 A235Azi 0 | 1 | 0 |
| | | | W176Azi 2 S239Azi 2 | 1 | 0 |
| | | | W176Azi 2 S239Azi 0 | 1 | 0 |
| | | | W176Azi 2 M243Azi 2 | 0,95687 | 0 |
| | | | W176Azi 2 M243Azi 0 | 0,99997 | 0 |
| | | | W176Azi 2 P245Azi 2 | 0,91032 | 0 |
| | | | W176Azi 2 P245Azi 0 | 0,9998 | 0 |
| | | | W176Azi 2 F250Azi 2 | 0,79971 | 0 |
| | | | W176Azi 2 F250Azi 0 | 1 | 0 |
| | | | W176Azi 2 A254Azi 2 | 0,13611 | 0 |
| | | | W176Azi 2 A254Azi 0 | 0,99905 | 0 |
| | | | W176Azi 2 L194Azi 2 | 1 | 0 |
| | | | W176Azi 2 L194Azi 0 | 1 | 0 |
| | | | W176Azi 2 V191Azi 2 | 0,05125 | 0 |
| | | | W176Azi 2 V191Azi 0 | 1 | 0 |
| | | | W176Azi 2 L188Azi 2 | 0,00345 | 1 |
| | | | W176Azi 2 L188Azi 0 | 1 | 0 |
| | | | W176Azi 2 F187Azi 2 | 1 | 0 |
| | | | W176Azi 2 F187Azi 0 | 1 | 0 |
| | | | W176Azi 2 L185Azi 2 | 1 | 0 |
| | | | W176Azi 2 L185Azi 0 | 1 | 0 |
| | | | W176Azi 2 V181Azi 2 | 0,9703 | 0 |
| | | | W176Azi 2 V181Azi 0 | 1 | 0 |
| | | | W176Azi 2 S179Azi 2 | 0,80504 | 0 |
| | | | W176Azi 2 S179Azi 0 | 0,99989 | 0 |
| | | | W176Azi 0 A235Azi 2 | 0,9976 | 0 |
| | | | W176Azi 0 A235Azi 0 | 1 | 0 |
| | | | W176Azi 0 S239Azi 2 | 1 | 0 |
| | | | W176Azi 0 S239Azi 0 | 1 | 0 |
| | | | W176Azi 0 M243Azi 2 | 0,99579 | 0 |
| | | | W176Azi 0 M243Azi 0 | 1 | 0 |
| | | | W176Azi 0 P245Azi 2 | 0,9282 | 0 |

| | | |
|---|---|---|
| W176Azi 0 P245Azi 0 | 0,99744 | 0 |
| W176Azi 0 F250Azi 2 | 0,86498 | 0 |
| W176Azi 0 F250Azi 0 | 1 | 0 |
| W176Azi 0 A254Azi 2 | 0,28012 | 0 |
| W176Azi 0 A254Azi 0 | 0,99591 | 0 |
| W176Azi 0 L194Azi 2 | 0,99981 | 0 |
| W176Azi 0 L194Azi 0 | 1 | 0 |
| W176Azi 0 V191Azi 2 | 0,06918 | 0 |
| W176Azi 0 V191Azi 0 | 1 | 0 |
| W176Azi 0 L188Azi 2 | 0,00553 | 1 |
| W176Azi 0 L188Azi 0 | 1 | 0 |
| W176Azi 0 F187Azi 2 | 0,99954 | 0 |
| W176Azi 0 F187Azi 0 | 0,99971 | 0 |
| W176Azi 0 L185Azi 2 | 1 | 0 |
| W176Azi 0 L185Azi 0 | 1 | 0 |
| W176Azi 0 V181Azi 2 | 0,99979 | 0 |
| W176Azi 0 V181Azi 0 | 0,99993 | 0 |
| W176Azi 0 S179Azi 2 | 1 | 0 |
| W176Azi 0 S179Azi 0 | 1 | 0 |
| W176Azi 0 W176Azi 2 | 1 | 0 |
| L174Azi 2 A235Azi 2 | 0,06582 | 0 |
| L174Azi 2 A235Azi 0 | 0,97803 | 0 |
| L174Azi 2 S239Azi 2 | 1 | 0 |
| L174Azi 2 S239Azi 0 | 0,99981 | 0 |
| L174Azi 2 M243Azi 2 | 0,89973 | 0 |
| L174Azi 2 M243Azi 0 | 0,93349 | 0 |
| L174Azi 2 P245Azi 2 | 0,89854 | 0 |
| L174Azi 2 P245Azi 0 | 1 | 0 |
| L174Azi 2 F250Azi 2 | 0,75679 | 0 |
| L174Azi 2 F250Azi 0 | 1 | 0 |
| L174Azi 2 A254Azi 2 | 0,08641 | 0 |
| L174Azi 2 A254Azi 0 | 0,99994 | 0 |
| L174Azi 2 L194Azi 2 | 1 | 0 |
| L174Azi 2 L194Azi 0 | 0,99965 | 0 |
| L174Azi 2 V191Azi 2 | 0,04295 | 1 |
| L174Azi 2 V191Azi 0 | 0,99871 | 0 |
| L174Azi 2 L188Azi 2 | 0,00263 | 1 |
| L174Azi 2 L188Azi 0 | 1 | 0 |
| L174Azi 2 F187Azi 2 | 1 | 0 |
| L174Azi 2 F187Azi 0 | 1 | 0 |
| L174Azi 2 L185Azi 2 | 0,99995 | 0 |
| L174Azi 2 L185Azi 0 | 1 | 0 |
| L174Azi 2 V181Azi 2 | 0,86133 | 0 |
| L174Azi 2 V181Azi 0 | 1 | 0 |
| L174Azi 2 S179Azi 2 | 0,04021 | 1 |
| L174Azi 2 S179Azi 0 | 0,71211 | 0 |
| L174Azi 2 W176Azi 2 | 1 | 0 |
| L174Azi 2 W176Azi 0 | 1 | 0 |
| L174Azi 0 A235Azi 2 | 0,65073 | 0 |
| L174Azi 0 A235Azi 0 | 1 | 0 |
| L174Azi 0 S239Azi 2 | 1 | 0 |
| L174Azi 0 S239Azi 0 | 1 | 0 |
| L174Azi 0 M243Azi 2 | 0,96794 | 0 |
| L174Azi 0 M243Azi 0 | 1 | 0 |
| L174Azi 0 P245Azi 2 | 0,91373 | 0 |
| L174Azi 0 P245Azi 0 | 0,99898 | 0 |
| L174Azi 0 F250Azi 2 | 0,81189 | 0 |
| L174Azi 0 F250Azi 0 | 1 | 0 |
| L174Azi 0 A254Azi 2 | 0,15425 | 0 |
| L174Azi 0 A254Azi 0 | 0,9971 | 0 |
| L174Azi 0 L194Azi 2 | 0,99999 | 0 |
| L174Azi 0 L194Azi 0 | 1 | 0 |
| L174Azi 0 V191Azi 2 | 0,05402 | 0 |
| L174Azi 0 V191Azi 0 | 1 | 0 |
| L174Azi 0 L188Azi 2 | 0,00374 | 1 |
| L174Azi 0 L188Azi 0 | 1 | 0 |
| L174Azi 0 F187Azi 2 | 0,99998 | 0 |
| L174Azi 0 F187Azi 0 | 0,99998 | 0 |
| L174Azi 0 L185Azi 2 | 1 | 0 |
| L174Azi 0 L185Azi 0 | 1 | 0 |
| L174Azi 0 V181Azi 2 | 0,98337 | 0 |
| L174Azi 0 V181Azi 0 | 1 | 0 |
| L174Azi 0 S179Azi 2 | 0,93321 | 0 |
| L174Azi 0 S179Azi 0 | 1 | 0 |
| L174Azi 0 W176Azi 2 | 1 | 0 |
| L174Azi 0 W176Azi 0 | 1 | 0 |
| L174Azi 0 L174Azi 2 | 1 | 0 |
| L130Azi 2 A235Azi 2 | 0,46427 | 0 |
| L130Azi 2 A235Azi 0 | 0,99999 | 0 |
| L130Azi 2 S239Azi 2 | 1 | 0 |
| L130Azi 2 S239Azi 0 | 1 | 0 |
| L130Azi 2 M243Azi 2 | 0,9545 | 0 |
| L130Azi 2 M243Azi 0 | 0,99996 | 0 |
| L130Azi 2 P245Azi 2 | 0,90956 | 0 |
| L130Azi 2 P245Azi 0 | 0,99994 | 0 |
| L130Azi 2 F250Azi 2 | 0,7971 | 0 |
| L130Azi 2 F250Azi 0 | 1 | 0 |
| L130Azi 2 A254Azi 2 | 0,13276 | 0 |
| L130Azi 2 A254Azi 0 | 0,99976 | 0 |
| L130Azi 2 L194Azi 2 | 1 | 0 |
| L130Azi 2 L194Azi 0 | 1 | 0 |
| L130Azi 2 V191Azi 2 | 0,05068 | 0 |
| L130Azi 2 V191Azi 0 | 1 | 0 |
| L130Azi 2 L188Azi 2 | 0,00339 | 1 |
| L130Azi 2 L188Azi 0 | 1 | 0 |
| L130Azi 2 F187Azi 2 | 1 | 0 |
| L130Azi 2 F187Azi 0 | 1 | 0 |
| L130Azi 2 L185Azi 2 | 1 | 0 |
| L130Azi 2 L185Azi 0 | 1 | 0 |
| L130Azi 2 V181Azi 2 | 0,96731 | 0 |
| L130Azi 2 V181Azi 0 | 1 | 0 |
| L130Azi 2 S179Azi 2 | 0,7848 | 0 |
| L130Azi 2 S179Azi 0 | 0,99988 | 0 |
| L130Azi 2 W176Azi 2 | 1 | 0 |
| L130Azi 2 W176Azi 0 | 1 | 0 |
| L130Azi 2 L174Azi 2 | 1 | 0 |
| L130Azi 2 L174Azi 0 | 1 | 0 |
| L130Azi 0 A235Azi 2 | 0,00202 | 1 |
| L130Azi 0 A235Azi 0 | 0,34548 | 0 |
| L130Azi 0 S239Azi 2 | 0,99914 | 0 |
| L130Azi 0 S239Azi 0 | 0,35938 | 0 |
| L130Azi 0 M243Azi 2 | 0,7734 | 0 |
| L130Azi 0 M243Azi 0 | 0,33596 | 0 |
| L130Azi 0 P245Azi 2 | 0,878 | 0 |
| L130Azi 0 P245Azi 0 | 1 | 0 |
| L130Azi 0 F250Azi 2 | 0,68586 | 0 |
| L130Azi 0 F250Azi 0 | 1 | 0 |

| | | | | | |
|---|---|---|---|---|---|
| | | | L130Azi 0 A254Azi 2 | 0,04309 | 1 |
| | | | L130Azi 0 A254Azi 0 | 1 | 0 |
| | | | L130Azi 0 L194Azi 2 | 1 | 0 |
| | | | L130Azi 0 L194Azi 0 | 0,74373 | 0 |
| | | | L130Azi 0 V191Azi 2 | 0,03256 | 1 |
| | | | L130Azi 0 V191Azi 0 | 0,90426 | 0 |
| | | | L130Azi 0 L188Azi 2 | 0,00173 | 1 |
| | | | L130Azi 0 L188Azi 0 | 0,89061 | 0 |
| | | | L130Azi 0 F187Azi 2 | 1 | 0 |
| | | | L130Azi 0 F187Azi 0 | 0,9993 | 0 |
| | | | L130Azi 0 L185Azi 2 | 0,46138 | 0 |
| | | | L130Azi 0 L185Azi 0 | 0,99799 | 0 |
| | | | L130Azi 0 V181Azi 2 | 0,56397 | 0 |
| | | | L130Azi 0 V181Azi 0 | 0,56693 | 0 |
| | | | L130Azi 0 S179Azi 2 | 2,47E-05 | 1 |
| | | | L130Azi 0 S179Azi 0 | 0,00772 | 1 |
| | | | L130Azi 0 W176Azi 2 | 0,97908 | 0 |
| | | | L130Azi 0 W176Azi 0 | 0,9793 | 0 |
| | | | L130Azi 0 L174Azi 2 | 0,96717 | 0 |
| | | | L130Azi 0 L174Azi 0 | 0,96519 | 0 |
| | | | L130Azi 0 L130Azi 2 | 0,99016 | 0 |
| | | | H134Azi 2 A235Azi 2 | 0,93991 | 0 |
| | | | H134Azi 2 A235Azi 0 | 1 | 0 |
| | | | H134Azi 2 S239Azi 2 | 1 | 0 |
| | | | H134Azi 2 S239Azi 0 | 1 | 0 |
| | | | H134Azi 2 M243Azi 2 | 0,99414 | 0 |
| | | | H134Azi 2 M243Azi 0 | 1 | 0 |
| | | | H134Azi 2 P245Azi 2 | 0,932 | 0 |
| | | | H134Azi 2 P245Azi 0 | 0,00431 | 1 |
| | | | H134Azi 2 F250Azi 2 | 0,87097 | 0 |
| | | | H134Azi 2 F250Azi 0 | 1 | 0 |
| | | | H134Azi 2 A254Azi 2 | 0,25793 | 0 |
| | | | H134Azi 2 A254Azi 0 | 3,08E-04 | 1 |
| | | | H134Azi 2 L194Azi 2 | 0,17002 | 0 |
| | | | H134Azi 2 L194Azi 0 | 1 | 0 |
| | | | H134Azi 2 V191Azi 2 | 0,07159 | 0 |
| | | | H134Azi 2 V191Azi 0 | 1 | 0 |
| | | | H134Azi 2 L188Azi 2 | 0,00581 | 1 |
| | | | H134Azi 2 L188Azi 0 | 0,61504 | 0 |
| | | | H134Azi 2 F187Azi 2 | 0,14613 | 0 |
| | | | H134Azi 2 F187Azi 0 | 0,01515 | 1 |
| | | | H134Azi 2 L185Azi 2 | 0,99995 | 0 |
| | | | H134Azi 2 L185Azi 0 | 1 | 0 |
| | | | H134Azi 2 V181Azi 2 | 0,99944 | 0 |
| | | | H134Azi 2 V181Azi 0 | 0,01241 | 1 |
| | | | H134Azi 2 S179Azi 2 | 0,99984 | 0 |
| | | | H134Azi 2 S179Azi 0 | 1 | 0 |
| | | | H134Azi 2 W176Azi 2 | 0,99993 | 0 |
| | | | H134Azi 2 W176Azi 0 | 1 | 0 |
| | | | H134Azi 2 L174Azi 2 | 0,46814 | 0 |
| | | | H134Azi 2 L174Azi 0 | 1 | 0 |
| | | | H134Azi 2 L130Azi 2 | 0,99993 | 0 |
| | | | H134Azi 2 L130Azi 0 | 1,40E-05 | 1 |
| | | | H134Azi 0 A235Azi 2 | 0,03693 | 1 |
| | | | H134Azi 0 A235Azi 0 | 0,93962 | 0 |
| | | | H134Azi 0 S239Azi 2 | 1 | 0 |
| | | | H134Azi 0 S239Azi 0 | 0,99703 | 0 |
| | | | H134Azi 0 M243Azi 2 | 0,88521 | 0 |
| | | | H134Azi 0 M243Azi 0 | 0,85825 | 0 |
| | | | H134Azi 0 P245Azi 2 | 0,89619 | 0 |
| | | | H134Azi 0 P245Azi 0 | 1 | 0 |
| | | | H134Azi 0 F250Azi 2 | 0,7482 | 0 |
| | | | H134Azi 0 F250Azi 0 | 1 | 0 |
| | | | H134Azi 0 A254Azi 2 | 0,07885 | 0 |
| | | | H134Azi 0 A254Azi 0 | 0,99997 | 0 |
| | | | H134Azi 0 L194Azi 2 | 1 | 0 |
| | | | H134Azi 0 L194Azi 0 | 0,99777 | 0 |
| | | | H134Azi 0 V191Azi 2 | 0,04151 | 1 |
| | | | H134Azi 0 V191Azi 0 | 0,99678 | 0 |
| | | | H134Azi 0 L188Azi 2 | 0,0025 | 1 |
| | | | H134Azi 0 L188Azi 0 | 1 | 0 |
| | | | H134Azi 0 F187Azi 2 | 1 | 0 |
| | | | H134Azi 0 F187Azi 0 | 1 | 0 |
| | | | H134Azi 0 L185Azi 2 | 0,99779 | 0 |
| | | | H134Azi 0 L185Azi 0 | 1 | 0 |
| | | | H134Azi 0 V181Azi 2 | 0,82658 | 0 |
| | | | H134Azi 0 V181Azi 0 | 1 | 0 |
| | | | H134Azi 0 S179Azi 2 | 0,00945 | 1 |
| | | | H134Azi 0 S179Azi 0 | 0,45416 | 0 |
| | | | H134Azi 0 W176Azi 2 | 1 | 0 |
| | | | H134Azi 0 W176Azi 0 | 1 | 0 |
| | | | H134Azi 0 L174Azi 2 | 1 | 0 |
| | | | H134Azi 0 L174Azi 0 | 1 | 0 |
| | | | H134Azi 0 L130Azi 2 | 1 | 0 |
| | | | H134Azi 0 L130Azi 0 | 0,9285 | 0 |
| | | | H134Azi 0 H134Azi 2 | 0,13616 | 0 |
| | | | F136Azi 2 A235Azi 2 | 0,14446 | 0 |
| | | | F136Azi 2 A235Azi 0 | 0,99869 | 0 |
| | | | F136Azi 2 S239Azi 2 | 1 | 0 |
| | | | F136Azi 2 S239Azi 0 | 1 | 0 |
| | | | F136Azi 2 M243Azi 2 | 0,93404 | 0 |
| | | | F136Azi 2 M243Azi 0 | 0,99002 | 0 |
| | | | F136Azi 2 P245Azi 2 | 0,90656 | 0 |
| | | | F136Azi 2 P245Azi 0 | 0,95772 | 0 |
| | | | F136Azi 2 F250Azi 2 | 0,78414 | 0 |
| | | | F136Azi 2 F250Azi 0 | 1 | 0 |
| | | | F136Azi 2 A254Azi 2 | 0,11107 | 0 |
| | | | F136Azi 2 A254Azi 0 | 0,79345 | 0 |
| | | | F136Azi 2 L194Azi 2 | 0,99993 | 0 |
| | | | F136Azi 2 L194Azi 0 | 1 | 0 |
| | | | F136Azi 2 V191Azi 2 | 0,04807 | 1 |
| | | | F136Azi 2 V191Azi 0 | 0,99992 | 0 |
| | | | F136Azi 2 L188Azi 2 | 0,00313 | 1 |
| | | | F136Azi 2 L188Azi 0 | 1 | 0 |
| | | | F136Azi 2 F187Azi 2 | 0,99984 | 0 |
| | | | F136Azi 2 F187Azi 0 | 0,99942 | 0 |
| | | | F136Azi 2 L185Azi 2 | 1 | 0 |
| | | | F136Azi 2 L185Azi 0 | 1 | 0 |
| | | | F136Azi 2 V181Azi 2 | 0,9289 | 0 |
| | | | F136Azi 2 V181Azi 0 | 0,99995 | 0 |
| | | | F136Azi 2 S179Azi 2 | 0,11787 | 0 |
| | | | F136Azi 2 S179Azi 0 | 0,94387 | 0 |
| | | | F136Azi 2 W176Azi 2 | 1 | 0 |
| | | | F136Azi 2 W176Azi 0 | 1 | 0 |
| | | | F136Azi 2 L174Azi 2 | 1 | 0 |
| | | | F136Azi 2 L174Azi 0 | 1 | 0 |
| | | | F136Azi 2 L130Azi 2 | 1 | 0 |

| | | | | | |
|---|---|---|---|---|---|
| | | | F136Azi 2 L130Azi 0 | 0,21351 | 0 |
| | | | F136Azi 2 H134Azi 2 | 0,84183 | 0 |
| | | | F136Azi 2 H134Azi 0 | 1 | 0 |
| | | | F136Azi 0 A235Azi 2 | 0,17168 | 0 |
| | | | F136Azi 0 A235Azi 0 | 0,99934 | 0 |
| | | | F136Azi 0 S239Azi 2 | 1 | 0 |
| | | | F136Azi 0 S239Azi 0 | 1 | 0 |
| | | | F136Azi 0 M243Azi 2 | 0,93799 | 0 |
| | | | F136Azi 0 M243Azi 0 | 0,99437 | 0 |
| | | | F136Azi 0 P245Azi 2 | 0,90738 | 0 |
| | | | F136Azi 0 P245Azi 0 | 0,96767 | 0 |
| | | | F136Azi 0 F250Azi 2 | 0,78714 | 0 |
| | | | F136Azi 0 F250Azi 0 | 1 | 0 |
| | | | F136Azi 0 A254Azi 2 | 0,11471 | 0 |
| | | | F136Azi 0 A254Azi 0 | 0,85124 | 0 |
| | | | F136Azi 0 L194Azi 2 | 0,99993 | 0 |
| | | | F136Azi 0 L194Azi 0 | 1 | 0 |
| | | | F136Azi 0 V191Azi 2 | 0,04868 | 1 |
| | | | F136Azi 0 V191Azi 0 | 0,99995 | 0 |
| | | | F136Azi 0 L188Azi 2 | 0,00319 | 1 |
| | | | F136Azi 0 L188Azi 0 | 1 | 0 |
| | | | F136Azi 0 F187Azi 2 | 0,99983 | 0 |
| | | | F136Azi 0 F187Azi 0 | 0,99954 | 0 |
| | | | F136Azi 0 L185Azi 2 | 1 | 0 |
| | | | F136Azi 0 L185Azi 0 | 1 | 0 |
| | | | F136Azi 0 V181Azi 2 | 0,93656 | 0 |
| | | | F136Azi 0 V181Azi 0 | 0,99996 | 0 |
| | | | F136Azi 0 S179Azi 2 | 0,17016 | 0 |
| | | | F136Azi 0 S179Azi 0 | 0,97025 | 0 |
| | | | F136Azi 0 W176Azi 2 | 1 | 0 |
| | | | F136Azi 0 W176Azi 0 | 1 | 0 |
| | | | F136Azi 0 L174Azi 2 | 1 | 0 |
| | | | F136Azi 0 L174Azi 0 | 1 | 0 |
| | | | F136Azi 0 L130Azi 2 | 1 | 0 |
| | | | F136Azi 0 L130Azi 0 | 0,31726 | 0 |
| | | | F136Azi 0 H134Azi 2 | 0,92289 | 0 |
| | | | F136Azi 0 H134Azi 0 | 1 | 0 |
| | | | F136Azi 0 F136Azi 2 | 1 | 0 |
| | | | A137Azi 2 A235Azi 2 | 0,04554 | 1 |
| | | | A137Azi 2 A235Azi 0 | 0,02336 | 1 |
| | | | A137Azi 2 S239Azi 2 | 0,01982 | 1 |
| | | | A137Azi 2 S239Azi 0 | 0,01678 | 1 |
| | | | A137Azi 2 M243Azi 2 | 0,16444 | 0 |
| | | | A137Azi 2 M243Azi 0 | 0,02222 | 1 |
| | | | A137Azi 2 P245Azi 2 | 1 | 0 |
| | | | A137Azi 2 P245Azi 0 | 0,00884 | 1 |
| | | | A137Azi 2 F250Azi 2 | 0,9999 | 0 |
| | | | A137Azi 2 F250Azi 0 | 0,01353 | 1 |
| | | | A137Azi 2 A254Azi 2 | 0,76089 | 0 |
| | | | A137Azi 2 A254Azi 0 | 0,00879 | 1 |
| | | | A137Azi 2 L194Azi 2 | 0,00934 | 1 |
| | | | A137Azi 2 L194Azi 0 | 0,01918 | 1 |
| | | | A137Azi 2 V191Azi 2 | 1 | 0 |
| | | | A137Azi 2 V191Azi 0 | 0,02986 | 1 |
| | | | A137Azi 2 L188Azi 2 | 1 | 0 |
| | | | A137Azi 2 L188Azi 0 | 0,01146 | 1 |
| | | | A137Azi 2 F187Azi 2 | 0,00909 | 1 |
| | | | A137Azi 2 F187Azi 0 | 0,00963 | 1 |
| | | | A137Azi 2 L185Azi 2 | 0,01488 | 1 |
| | | | A137Azi 2 L185Azi 0 | 0,01403 | 1 |
| | | | A137Azi 2 V181Azi 2 | 0,07571 | 0 |
| | | | A137Azi 2 V181Azi 0 | 0,01014 | 1 |
| | | | A137Azi 2 S179Azi 2 | 0,02906 | 1 |
| | | | A137Azi 2 S179Azi 0 | 0,02151 | 1 |
| | | | A137Azi 2 W176Azi 2 | 0,01344 | 1 |
| | | | A137Azi 2 W176Azi 0 | 0,0189 | 1 |
| | | | A137Azi 2 L174Azi 2 | 0,01103 | 1 |
| | | | A137Azi 2 L174Azi 0 | 0,01427 | 1 |
| | | | A137Azi 2 L130Azi 2 | 0,01327 | 1 |
| | | | A137Azi 2 L130Azi 0 | 0,00809 | 1 |
| | | | A137Azi 2 H134Azi 2 | 0,01977 | 1 |
| | | | A137Azi 2 H134Azi 0 | 0,01062 | 1 |
| | | | A137Azi 2 F136Azi 2 | 0,01253 | 1 |
| | | | A137Azi 2 F136Azi 0 | 0,01271 | 1 |
| | | | A137Azi 0 A235Azi 2 | 0,00122 | 1 |
| | | | A137Azi 0 A235Azi 0 | 0,25499 | 0 |
| | | | A137Azi 0 S239Azi 2 | 0,99742 | 0 |
| | | | A137Azi 0 S239Azi 0 | 0,23989 | 0 |
| | | | A137Azi 0 M243Azi 2 | 0,749 | 0 |
| | | | A137Azi 0 M243Azi 0 | 0,2719 | 0 |
| | | | A137Azi 0 P245Azi 2 | 0,87415 | 0 |
| | | | A137Azi 0 P245Azi 0 | 1 | 0 |
| | | | A137Azi 0 F250Azi 2 | 0,67314 | 0 |
| | | | A137Azi 0 F250Azi 0 | 1 | 0 |
| | | | A137Azi 0 A254Azi 2 | 0,03822 | 1 |
| | | | A137Azi 0 A254Azi 0 | 0,99938 | 0 |
| | | | A137Azi 0 L194Azi 2 | 1 | 0 |
| | | | A137Azi 0 L194Azi 0 | 0,64817 | 0 |
| | | | A137Azi 0 V191Azi 2 | 0,03101 | 1 |
| | | | A137Azi 0 V191Azi 0 | 0,86207 | 0 |
| | | | A137Azi 0 L188Azi 2 | 0,00161 | 1 |
| | | | A137Azi 0 L188Azi 0 | 0,71586 | 0 |
| | | | A137Azi 0 F187Azi 2 | 1 | 0 |
| | | | A137Azi 0 F187Azi 0 | 0,9749 | 0 |
| | | | A137Azi 0 L185Azi 2 | 0,36358 | 0 |
| | | | A137Azi 0 L185Azi 0 | 0,99354 | 0 |
| | | | A137Azi 0 V181Azi 2 | 0,51211 | 0 |
| | | | A137Azi 0 V181Azi 0 | 0,25235 | 0 |
| | | | A137Azi 0 S179Azi 2 | 1,02E-05 | 1 |
| | | | A137Azi 0 S179Azi 0 | 0,00367 | 1 |
| | | | A137Azi 0 W176Azi 2 | 0,93863 | 0 |
| | | | A137Azi 0 W176Azi 0 | 0,95735 | 0 |
| | | | A137Azi 0 L174Azi 2 | 0,86678 | 0 |
| | | | A137Azi 0 L174Azi 0 | 0,91486 | 0 |
| | | | A137Azi 0 L130Azi 2 | 0,96285 | 0 |
| | | | A137Azi 0 L130Azi 0 | 1 | 0 |
| | | | A137Azi 0 H134Azi 2 | 4,19E-06 | 1 |
| | | | A137Azi 0 H134Azi 0 | 0,72072 | 0 |
| | | | A137Azi 0 F136Azi 2 | 0,09114 | 0 |
| | | | A137Azi 0 F136Azi 0 | 0,15478 | 0 |
| | | | A137Azi 0 A137Azi 2 | 0,00766 | 1 |
| | | | L138Azi 2 A235Azi 2 | 0,9912 | 0 |
| | | | L138Azi 2 A235Azi 0 | 1 | 0 |
| | | | L138Azi 2 S239Azi 2 | 1 | 0 |
| | | | L138Azi 2 S239Azi 0 | 1 | 0 |
| | | | L138Azi 2 M243Azi 2 | 0,99453 | 0 |
| | | | L138Azi 2 M243Azi 0 | 1 | 0 |

| | | | | | |
|---|---|---|---|---|---|
| | | | L138Azi 2 P245Azi 2 | 0,9291 | 0 |
| | | | L138Azi 2 P245Azi 0 | 0,93054 | 0 |
| | | | L138Azi 2 F250Azi 2 | 0,86491 | 0 |
| | | | L138Azi 2 F250Azi 0 | 1 | 0 |
| | | | L138Azi 2 A254Azi 2 | 0,26228 | 0 |
| | | | L138Azi 2 A254Azi 0 | 0,89568 | 0 |
| | | | L138Azi 2 L194Azi 2 | 0,98968 | 0 |
| | | | L138Azi 2 L194Azi 0 | 1 | 0 |
| | | | L138Azi 2 V191Azi 2 | 0,06925 | 0 |
| | | | L138Azi 2 V191Azi 0 | 1 | 0 |
| | | | L138Azi 2 L188Azi 2 | 0,00552 | 1 |
| | | | L138Azi 2 L188Azi 0 | 0,99998 | 0 |
| | | | L138Azi 2 F187Azi 2 | 0,98461 | 0 |
| | | | L138Azi 2 F187Azi 0 | 0,98441 | 0 |
| | | | L138Azi 2 L185Azi 2 | 1 | 0 |
| | | | L138Azi 2 L185Azi 0 | 1 | 0 |
| | | | L138Azi 2 V181Azi 2 | 0,99962 | 0 |
| | | | L138Azi 2 V181Azi 0 | 0,99346 | 0 |
| | | | L138Azi 2 S179Azi 2 | 1 | 0 |
| | | | L138Azi 2 S179Azi 0 | 1 | 0 |
| | | | L138Azi 2 W176Azi 2 | 1 | 0 |
| | | | L138Azi 2 W176Azi 0 | 1 | 0 |
| | | | L138Azi 2 L174Azi 2 | 0,99991 | 0 |
| | | | L138Azi 2 L174Azi 0 | 1 | 0 |
| | | | L138Azi 2 L130Azi 2 | 1 | 0 |
| | | | L138Azi 2 L130Azi 0 | 0,72132 | 0 |
| | | | L138Azi 2 H134Azi 2 | 1 | 0 |
| | | | L138Azi 2 H134Azi 0 | 0,99919 | 0 |
| | | | L138Azi 2 F136Azi 2 | 1 | 0 |
| | | | L138Azi 2 F136Azi 0 | 1 | 0 |
| | | | L138Azi 2 A137Azi 2 | 0,01896 | 1 |
| | | | L138Azi 2 A137Azi 0 | 0,5989 | 0 |
| | | | L138Azi 0 A235Azi 2 | 0,1578 | 0 |
| | | | L138Azi 0 A235Azi 0 | 0,99845 | 0 |
| | | | L138Azi 0 S239Azi 2 | 1 | 0 |
| | | | L138Azi 0 S239Azi 0 | 1 | 0 |
| | | | L138Azi 0 M243Azi 2 | 0,92744 | 0 |
| | | | L138Azi 0 M243Azi 0 | 0,99141 | 0 |
| | | | L138Azi 0 P245Azi 2 | 0,9041 | 0 |
| | | | L138Azi 0 P245Azi 0 | 0,99989 | 0 |
| | | | L138Azi 0 F250Azi 2 | 0,77647 | 0 |
| | | | L138Azi 0 F250Azi 0 | 1 | 0 |
| | | | L138Azi 0 A254Azi 2 | 0,10514 | 0 |
| | | | L138Azi 0 A254Azi 0 | 0,99905 | 0 |
| | | | L138Azi 0 L194Azi 2 | 1 | 0 |
| | | | L138Azi 0 L194Azi 0 | 1 | 0 |
| | | | L138Azi 0 V191Azi 2 | 0,04653 | 1 |
| | | | L138Azi 0 V191Azi 0 | 0,9999 | 0 |
| | | | L138Azi 0 L188Azi 2 | 0,00297 | 1 |
| | | | L138Azi 0 L188Azi 0 | 1 | 0 |
| | | | L138Azi 0 F187Azi 2 | 1 | 0 |
| | | | L138Azi 0 F187Azi 0 | 1 | 0 |
| | | | L138Azi 0 L185Azi 2 | 1 | 0 |
| | | | L138Azi 0 L185Azi 0 | 1 | 0 |
| | | | L138Azi 0 V181Azi 2 | 0,91951 | 0 |
| | | | L138Azi 0 V181Azi 0 | 1 | 0 |
| | | | L138Azi 0 S179Azi 2 | 0,18975 | 0 |
| | | | L138Azi 0 S179Azi 0 | 0,96113 | 0 |
| | | | L138Azi 0 W176Azi 2 | 1 | 0 |
| | | | L138Azi 0 W176Azi 0 | 1 | 0 |
| | | | L138Azi 0 L174Azi 2 | 1 | 0 |
| | | | L138Azi 0 L174Azi 0 | 1 | 0 |
| | | | L138Azi 0 L130Azi 2 | 1 | 0 |
| | | | L138Azi 0 L130Azi 0 | 0,93545 | 0 |
| | | | L138Azi 0 H134Azi 2 | 0,9248 | 0 |
| | | | L138Azi 0 H134Azi 0 | 1 | 0 |
| | | | L138Azi 0 F136Azi 2 | 1 | 0 |
| | | | L138Azi 0 F136Azi 0 | 1 | 0 |
| | | | L138Azi 0 A137Azi 2 | 0,01207 | 1 |
| | | | L138Azi 0 A137Azi 0 | 0,80521 | 0 |
| | | | L138Azi 0 L138Azi 2 | 1 | 0 |
| | | | O1 2 A235Azi 2 | 0,00102 | 1 |
| | | | O1 2 A235Azi 0 | 0,22676 | 0 |
| | | | O1 2 S239Azi 2 | 0,9966 | 0 |
| | | | O1 2 S239Azi 0 | 0,19795 | 0 |
| | | | O1 2 M243Azi 2 | 0,74289 | 0 |
| | | | O1 2 M243Azi 0 | 0,2529 | 0 |
| | | | O1 2 P245Azi 2 | 0,87332 | 0 |
| | | | O1 2 P245Azi 0 | 0,99992 | 0 |
| | | | O1 2 F250Azi 2 | 0,67032 | 0 |
| | | | O1 2 F250Azi 0 | 1 | 0 |
| | | | O1 2 A254Azi 2 | 0,03712 | 1 |
| | | | O1 2 A254Azi 0 | 0,97884 | 0 |
| | | | O1 2 L194Azi 2 | 1 | 0 |
| | | | O1 2 L194Azi 0 | 0,61358 | 0 |
| | | | O1 2 V191Azi 2 | 0,03068 | 1 |
| | | | O1 2 V191Azi 0 | 0,84834 | 0 |
| | | | O1 2 L188Azi 2 | 0,00158 | 1 |
| | | | O1 2 L188Azi 0 | 0,59555 | 0 |
| | | | O1 2 F187Azi 2 | 1 | 0 |
| | | | O1 2 F187Azi 0 | 0,90316 | 0 |
| | | | O1 2 L185Azi 2 | 0,32917 | 0 |
| | | | O1 2 L185Azi 0 | 0,99109 | 0 |
| | | | O1 2 V181Azi 2 | 0,49843 | 0 |
| | | | O1 2 V181Azi 0 | 0,06498 | 0 |
| | | | O1 2 S179Azi 2 | 7,13E-06 | 1 |
| | | | O1 2 S179Azi 0 | 0,00258 | 1 |
| | | | O1 2 W176Azi 2 | 0,91424 | 0 |
| | | | O1 2 W176Azi 0 | 0,94806 | 0 |
| | | | O1 2 L174Azi 2 | 0,777 | 0 |
| | | | O1 2 L174Azi 0 | 0,88798 | 0 |
| | | | O1 2 L130Azi 2 | 0,94447 | 0 |
| | | | O1 2 L130Azi 0 | 1 | 0 |
| | | | O1 2 H134Azi 2 | 1,77E-06 | 1 |
| | | | O1 2 H134Azi 0 | 0,53016 | 0 |
| | | | O1 2 F136Azi 2 | 0,04461 | 1 |
| | | | O1 2 F136Azi 0 | 0,08944 | 0 |
| | | | O1 2 A137Azi 2 | 0,00757 | 1 |
| | | | O1 2 A137Azi 0 | 1 | 0 |
| | | | O1 2 L138Azi 2 | 0,55373 | 0 |
| | | | O1 2 L138Azi 0 | 0,71598 | 0 |
| | | | O1 0 A235Azi 2 | 6,94E-04 | 1 |
| | | | O1 0 A235Azi 0 | 0,17398 | 0 |
| | | | O1 0 S239Azi 2 | 0,99346 | 0 |
| | | | O1 0 S239Azi 0 | 0,13399 | 0 |
| | | | O1 0 M243Azi 2 | 0,72555 | 0 |
| | | | O1 0 M243Azi 0 | 0,21428 | 0 |

| | | | | | | |
|---|---|---|---|---|---|---|
| | | | | O1 0 P245Azi 2 | 0,87071 | 0 |
| | | | | O1 0 P245Azi 0 | 0,99451 | 0 |
| | | | | O1 0 F250Azi 2 | 0,6617 | 0 |
| | | | | O1 0 F250Azi 0 | 1 | 0 |
| | | | | O1 0 A254Azi 2 | 0,03415 | 1 |
| | | | | O1 0 A254Azi 0 | 0,67489 | 0 |
| | | | | O1 0 L194Azi 2 | 0,99985 | 0 |
| | | | | O1 0 L194Azi 0 | 0,53829 | 0 |
| | | | | O1 0 V191Azi 2 | 0,02969 | 1 |
| | | | | O1 0 V191Azi 0 | 0,81097 | 0 |
| | | | | O1 0 L188Azi 2 | 0,00151 | 1 |
| | | | | O1 0 L188Azi 0 | 0,39996 | 0 |
| | | | | O1 0 F187Azi 2 | 1 | 0 |
| | | | | O1 0 F187Azi 0 | 0,66161 | 0 |
| | | | | O1 0 L185Azi 2 | 0,26984 | 0 |
| | | | | O1 0 L185Azi 0 | 0,9828 | 0 |
| | | | | O1 0 V181Azi 2 | 0,46283 | 0 |
| | | | | O1 0 V181Azi 0 | 0,00897 | 1 |
| | | | | O1 0 S179Azi 2 | 3,53E-06 | 1 |
| | | | | O1 0 S179Azi 0 | 0,00135 | 1 |
| | | | | O1 0 W176Azi 2 | 0,84748 | 0 |
| | | | | O1 0 W176Azi 0 | 0,92064 | 0 |
| | | | | O1 0 L174Azi 2 | 0,59097 | 0 |
| | | | | O1 0 L174Azi 0 | 0,8179 | 0 |
| | | | | O1 0 L130Azi 2 | 0,88746 | 0 |
| | | | | O1 0 L130Azi 0 | 0,96694 | 0 |
| | | | | O1 0 H134Azi 2 | 5,05E-07 | 1 |
| | | | | O1 0 H134Azi 0 | 0,26644 | 0 |
| | | | | O1 0 F136Azi 2 | 0,01457 | 1 |
| | | | | O1 0 F136Azi 0 | 0,03561 | 1 |
| | | | | O1 0 A137Azi 2 | 0,0073 | 1 |
| | | | | O1 0 A137Azi 0 | 1 | 0 |
| | | | | O1 0 L138Azi 2 | 0,45815 | 0 |
| | | | | O1 0 L138Azi 0 | 0,53389 | 0 |
| | | | | O1 0 O1 2 | 1 | 0 |
| | | | | | | |
| | | | | | | |
| S1a - before and after UV | Welch-ANOVA | 0 | F(43, 371.88)=11.55 | O1 after UV O1 before UV | 1 | 0 |
| | | | | O1+STIM1 before TG O1 before UV | 1 | 0 |
| | | | | O1+STIM1 before TG O1 after UV | 1 | 0 |
| | | | | O1+STIM1 after TG O1 before UV | 0,03927 | 1 |
| | | | | O1+STIM1 after TG O1 after UV | 0,0423 | 1 |
| | | | | O1+STIM1 after TG O1+STIM1 before TG | 0,04093 | 1 |
| | | | | L130Azi before UV O1 before UV | 1 | 0 |
| | | | | L130Azi before UV O1 after UV | 1 | 0 |
| | | | | L130Azi before UV O1+STIM1 before TG | 0,98969 | 0 |
| | | | | L130Azi before UV O1+STIM1 after TG | 0,03622 | 1 |
| | | | | L130Azi after UV O1 before UV | 0,06265 | 0 |
| | | | | L130Azi after UV O1 after UV | 0,09572 | 0 |
| | | | | L130Azi after UV O1+STIM1 before TG | 0,06301 | 0 |
| | | | | L130Azi after UV O1+STIM1 after TG | 0,20789 | 0 |
| | | | | L130Azi after UV L130Azi before UV | 0,02771 | 1 |
| | | | | H134Azi before UV O1 before UV | 1 | 0 |
| | | | | H134Azi before UV O1 after UV | 1 | 0 |
| | | | | H134Azi before UV O1+STIM1 before TG | 1 | 0 |
| | | | | H134Azi before UV O1+STIM1 after TG | 0,03926 | 1 |
| | | | | H134Azi before UV L130Azi before UV | 0,99996 | 0 |
| | | | | H134Azi before UV L130Azi after UV | 0,04677 | 1 |
| | | | | H134Azi after UV O1 before UV | 1 | 0 |
| | | | | H134Azi after UV O1 after UV | 1 | 0 |
| | | | | H134Azi after UV O1+STIM1 before TG | 0,9954 | 0 |
| | | | | H134Azi after UV O1+STIM1 after TG | 0,03748 | 1 |
| | | | | H134Azi after UV L130Azi before UV | 1 | 0 |
| | | | | H134Azi after UV L130Azi after UV | 0,034 | 1 |
| | | | | H134Azi after UV H134Azi before UV | 1 | 0 |
| | | | | F136Azi before UV O1 before UV | 1 | 0 |
| | | | | F136Azi before UV O1 after UV | 0,99976 | 0 |
| | | | | F136Azi before UV O1+STIM1 before TG | 0,66796 | 0 |
| | | | | F136Azi before UV O1+STIM1 after TG | 0,03593 | 1 |
| | | | | F136Azi before UV L130Azi before UV | 1 | 0 |
| | | | | F136Azi before UV L130Azi after UV | 0,02518 | 1 |
| | | | | F136Azi before UV H134Azi before UV | 0,85717 | 0 |
| | | | | F136Azi before UV H134Azi after UV | 1 | 0 |
| | | | | F136Azi after UV O1 before UV | 1 | 0 |
| | | | | F136Azi after UV O1 after UV | 1 | 0 |
| | | | | F136Azi after UV O1+STIM1 before TG | 1 | 0 |
| | | | | F136Azi after UV O1+STIM1 after TG | 0,03865 | 1 |
| | | | | F136Azi after UV L130Azi before UV | 1 | 0 |
| | | | | F136Azi after UV L130Azi after UV | 0,04201 | 1 |
| | | | | F136Azi after UV H134Azi before UV | 1 | 0 |
| | | | | F136Azi after UV H134Azi after UV | 1 | 0 |
| | | | | F136Azi after UV F136Azi before UV | 0,9815 | 0 |
| | | | | A137Azi before UV O1 before UV | 0,02429 | 1 |
| | | | | A137Azi before UV O1 after UV | 0,03445 | 1 |
| | | | | A137Azi before UV O1+STIM1 before TG | 0,02633 | 1 |
| | | | | A137Azi before UV O1+STIM1 after TG | 0,34934 | 0 |
| | | | | A137Azi before UV L130Azi before UV | 0,01422 | 1 |
| | | | | A137Azi before UV L130Azi after UV | 1 | 0 |
| | | | | A137Azi before UV H134Azi before UV | 0,02115 | 1 |
| | | | | A137Azi before UV H134Azi after UV | 0,0167 | 1 |
| | | | | A137Azi before UV F136Azi before UV | 0,01343 | 1 |
| | | | | A137Azi before UV F136Azi after UV | 0,01953 | 1 |
| | | | | A137Azi after UV O1 before UV | 0,04644 | 1 |
| | | | | A137Azi after UV O1 after UV | 0,06548 | 0 |
| | | | | A137Azi after UV O1+STIM1 before TG | 0,04952 | 1 |
| | | | | A137Azi after UV O1+STIM1 after TG | 0,29227 | 0 |
| | | | | A137Azi after UV L130Azi before UV | 0,02671 | 1 |
| | | | | A137Azi after UV L130Azi after UV | 1 | 0 |
| | | | | A137Azi after UV H134Azi before UV | 0,03972 | 1 |
| | | | | A137Azi after UV H134Azi after UV | 0,03134 | 1 |
| | | | | A137Azi after UV F136Azi before UV | 0,02514 | 1 |
| | | | | A137Azi after UV F136Azi after UV | 0,03667 | 1 |
| | | | | A137Azi after UV A137Azi before UV | 1 | 0 |
| | | | | L138Azi before UV O1 before UV | 1 | 0 |
| | | | | L138Azi before UV O1 after UV | 1 | 0 |
| | | | | L138Azi before UV O1+STIM1 before TG | 1 | 0 |
| | | | | L138Azi before UV O1+STIM1 after TG | 0,03867 | 1 |
| | | | | L138Azi before UV L130Azi before UV | 1 | 0 |
| | | | | L138Azi before UV L130Azi after UV | 0,04474 | 1 |
| | | | | L138Azi before UV H134Azi before UV | 1 | 0 |
| | | | | L138Azi before UV H134Azi after UV | 1 | 0 |
| | | | | L138Azi before UV F136Azi before UV | 1 | 0 |
| | | | | L138Azi before UV F136Azi after UV | 1 | 0 |
| | | | | L138Azi before UV A137Azi before UV | 0,02009 | 1 |
| | | | | L138Azi before UV A137Azi after UV | 0,03788 | 1 |
| | | | | L138Azi after UV O1 before UV | 0,96767 | 0 |
| | | | | L138Azi after UV O1 after UV | 0,99834 | 0 |

| | | |
|---|---|---|
| L138Azi after UV O1+STIM1 before TG | 0,88137 | 0 |
| L138Azi after UV O1+STIM1  after TG | 0,05715 | 0 |
| L138Azi after UV L130Azi before UV | 0,27819 | 0 |
| L138Azi after UV L130Azi after UV | 0,47322 | 0 |
| L138Azi after UV H134Azi before UV | 0,63144 | 0 |
| L138Azi after UV H134Azi after UV | 0,36288 | 0 |
| L138Azi after UV F136Azi before UV | 0,17844 | 0 |
| L138Azi after UV F136Azi after UV | 0,53488 | 0 |
| L138Azi after UV A137Azi before UV | 0,14753 | 0 |
| L138Azi after UV A137Azi after UV | 0,26325 | 0 |
| L138Azi after UV L138Azi before UV | 0,69032 | 0 |
| L194Azi before UV O1 before UV | 1 | 0 |
| L194Azi before UV O1 after UV | 0,9823 | 0 |
| L194Azi before UV O1+STIM1 before TG | 0,17263 | 0 |
| L194Azi before UV O1+STIM1  after TG | 0,03392 | 1 |
| L194Azi before UV L130Azi before UV | 1 | 0 |
| L194Azi before UV L130Azi after UV | 0,01682 | 1 |
| L194Azi before UV H134Azi before UV | 0,18 | 0 |
| L194Azi before UV H134Azi after UV | 0,8645 | 0 |
| L194Azi before UV F136Azi before UV | 0,99996 | 0 |
| L194Azi before UV F136Azi after UV | 0,34412 | 0 |
| L194Azi before UV A137Azi before UV | 0,01002 | 1 |
| L194Azi before UV A137Azi after UV | 0,01869 | 1 |
| L194Azi before UV L138Azi before UV | 0,9919 | 0 |
| L194Azi before UV L138Azi after UV | 0,06307 | 0 |
| L194Azi after UV O1 before UV | 0,7635 | 0 |
| L194Azi after UV O1 after UV | 0,93159 | 0 |
| L194Azi after UV O1+STIM1 before TG | 0,4998 | 0 |
| L194Azi after UV O1+STIM1  after TG | 0,06183 | 0 |
| L194Azi after UV L130Azi before UV | 0,11762 | 0 |
| L194Azi after UV L130Azi after UV | 0,61625 | 0 |
| L194Azi after UV H134Azi before UV | 0,28761 | 0 |
| L194Azi after UV H134Azi after UV | 0,15686 | 0 |
| L194Azi after UV F136Azi before UV | 0,08415 | 0 |
| L194Azi after UV F136Azi after UV | 0,23532 | 0 |
| L194Azi after UV A137Azi before UV | 0,20474 | 0 |
| L194Azi after UV A137Azi after UV | 0,35234 | 0 |
| L194Azi after UV L138Azi before UV | 0,32528 | 0 |
| L194Azi after UV L138Azi after UV | 1 | 0 |
| L194Azi after UV L194Azi before UV | 0,03673 | 1 |
| V191Azi before UV O1 before UV | 0,0862 | 0 |
| V191Azi before UV O1 after UV | 0,11558 | 0 |
| V191Azi before UV O1+STIM1 before TG | 0,09183 | 0 |
| V191Azi before UV O1+STIM1  after TG | 0,32503 | 0 |
| V191Azi before UV L130Azi before UV | 0,05398 | 0 |
| V191Azi before UV L130Azi after UV | 1 | 0 |
| V191Azi before UV H134Azi before UV | 0,07605 | 0 |
| V191Azi before UV H134Azi after UV | 0,06199 | 0 |
| V191Azi before UV F136Azi before UV | 0,05121 | 0 |
| V191Azi before UV F136Azi after UV | 0,071 | 0 |
| V191Azi before UV A137Azi before UV | 1 | 0 |
| V191Azi before UV A137Azi after UV | 1 | 0 |
| V191Azi before UV L138Azi before UV | 0,07295 | 0 |
| V191Azi before UV L138Azi after UV | 0,36277 | 0 |
| V191Azi before UV L194Azi before UV | 0,03956 | 1 |
| V191Azi before UV L194Azi after UV | 0,45726 | 0 |
| V191Azi after UV O1 before UV | 0,9839 | 0 |
| V191Azi after UV O1 after UV | 0,99984 | 0 |
| V191Azi after UV O1+STIM1 before TG | 0,59813 | 0 |
| V191Azi after UV O1+STIM1  after TG | 0,05075 | 0 |
| V191Azi after UV L130Azi before UV | 0,01896 | 1 |
| V191Azi after UV L130Azi after UV | 0,23381 | 0 |
| V191Azi after UV H134Azi before UV | 0,1183 | 0 |
| V191Azi after UV H134Azi after UV | 0,01906 | 1 |
| V191Azi after UV F136Azi before UV | 0,00271 | 1 |
| V191Azi after UV F136Azi after UV | 0,06394 | 0 |
| V191Azi after UV A137Azi before UV | 0,07623 | 0 |
| V191Azi after UV A137Azi after UV | 0,14066 | 0 |
| V191Azi after UV L138Azi before UV | 0,34384 | 0 |
| V191Azi after UV L138Azi after UV | 1 | 0 |
| V191Azi after UV L194Azi before UV | 3,31E-04 | 1 |
| V191Azi after UV L194Azi after UV | 0,99999 | 0 |
| V191Azi after UV V191Azi before UV | 0,22043 | 0 |
| L188Azi before UV O1 before UV | 0,00796 | 1 |
| L188Azi before UV O1 after UV | 0,01273 | 1 |
| L188Azi before UV O1+STIM1 before TG | 0,00845 | 1 |
| L188Azi before UV O1+STIM1  after TG | 0,26287 | 0 |
| L188Azi before UV L130Azi before UV | 0,00365 | 1 |
| L188Azi before UV L130Azi after UV | 1 | 0 |
| L188Azi before UV H134Azi before UV | 0,00625 | 1 |
| L188Azi before UV H134Azi after UV | 0,00453 | 1 |
| L188Azi before UV F136Azi before UV | 0,00337 | 1 |
| L188Azi before UV F136Azi after UV | 0,0056 | 1 |
| L188Azi before UV A137Azi before UV | 1 | 0 |
| L188Azi before UV A137Azi after UV | 1 | 0 |
| L188Azi before UV L138Azi before UV | 0,00586 | 1 |
| L188Azi before UV L138Azi after UV | 0,09736 | 0 |
| L188Azi before UV L194Azi before UV | 0,00227 | 1 |
| L188Azi before UV L194Azi after UV | 0,15338 | 0 |
| L188Azi before UV V191Azi before UV | 1 | 0 |
| L188Azi before UV V191Azi after UV | 0,03686 | 1 |
| L188Azi after UV O1 before UV | 0,86762 | 0 |
| L188Azi after UV O1 after UV | 0,98576 | 0 |
| L188Azi after UV O1+STIM1 before TG | 0,14957 | 0 |
| L188Azi after UV O1+STIM1  after TG | 0,05373 | 0 |
| L188Azi after UV L130Azi before UV | 0,00155 | 1 |
| L188Azi after UV L130Azi after UV | 0,30981 | 0 |
| L188Azi after UV H134Azi before UV | 0,01256 | 1 |
| L188Azi after UV H134Azi after UV | 0,00152 | 1 |
| L188Azi after UV F136Azi before UV | 1,92E-04 | 1 |
| L188Azi after UV F136Azi after UV | 0,00599 | 1 |
| L188Azi after UV A137Azi before UV | 0,09897 | 0 |
| L188Azi after UV A137Azi after UV | 0,18022 | 0 |
| L188Azi after UV L138Azi before UV | 0,06443 | 0 |
| L188Azi after UV L138Azi after UV | 1 | 0 |
| L188Azi after UV L194Azi before UV | 2,17E-05 | 1 |
| L188Azi after UV L194Azi after UV | 1 | 0 |
| L188Azi after UV V191Azi before UV | 0,27016 | 0 |
| L188Azi after UV V191Azi after UV | 1 | 0 |
| L188Azi after UV L188Azi before UV | 0,05295 | 0 |
| F187Azi before UV O1 before UV | 1 | 0 |
| F187Azi before UV O1 after UV | 0,97771 | 0 |
| F187Azi before UV O1+STIM1 before TG | 0,15212 | 0 |
| F187Azi before UV O1+STIM1  after TG | 0,03373 | 1 |
| F187Azi before UV L130Azi before UV | 1 | 0 |
| F187Azi before UV L130Azi after UV | 0,01621 | 1 |
| F187Azi before UV H134Azi before UV | 0,15528 | 0 |

| | | |
|---|---|---|
| F187Azi before UV H134Azi after UV | 0,83938 | 0 |
| F187Azi before UV F136Azi before UV | 0,99989 | 0 |
| F187Azi before UV F136Azi after UV | 0,3091 | 0 |
| F187Azi before UV A137Azi before UV | 0,00975 | 1 |
| F187Azi before UV A137Azi after UV | 0,01818 | 1 |
| F187Azi before UV L138Azi before UV | 0,98768 | 0 |
| F187Azi before UV L138Azi after UV | 0,05747 | 0 |
| F187Azi before UV L194Azi before UV | 1 | 0 |
| F187Azi before UV L194Azi after UV | 0,03406 | 1 |
| F187Azi before UV V191Azi before UV | 0,03862 | 1 |
| F187Azi before UV V191Azi after UV | 2,64E-04 | 1 |
| F187Azi before UV L188Azi before UV | 0,00219 | 1 |
| F187Azi before UV L188Azi after UV | 1,65E-05 | 1 |
| F187Azi after UV O1 before UV | 0,00833 | 1 |
| F187Azi after UV O1 after UV | 0,00985 | 1 |
| F187Azi after UV O1+STIM1 before TG | 0,00902 | 1 |
| F187Azi after UV O1+STIM1  after TG | 1 | 0 |
| F187Azi after UV L130Azi before UV | 0,00682 | 1 |
| F187Azi after UV L130Azi after UV | 0,32533 | 0 |
| F187Azi after UV H134Azi before UV | 0,00819 | 1 |
| F187Azi after UV H134Azi after UV | 0,00736 | 1 |
| F187Azi after UV F136Azi before UV | 0,00668 | 1 |
| F187Azi after UV F136Azi after UV | 0,0079 | 1 |
| F187Azi after UV A137Azi before UV | 0,69717 | 0 |
| F187Azi after UV A137Azi after UV | 0,56098 | 0 |
| F187Azi after UV L138Azi before UV | 0,00794 | 1 |
| F187Azi after UV L138Azi after UV | 0,01964 | 1 |
| F187Azi after UV L194Azi before UV | 0,00585 | 1 |
| F187Azi after UV L194Azi after UV | 0,02346 | 1 |
| F187Azi after UV V191Azi before UV | 0,65307 | 0 |
| F187Azi after UV V191Azi after UV | 0,01478 | 1 |
| F187Azi after UV L188Azi before UV | 0,46685 | 0 |
| F187Azi after UV L188Azi after UV | 0,01682 | 1 |
| F187Azi after UV F187Azi before UV | 0,00578 | 1 |
| L185Azi  before UV O1 before UV | 1 | 0 |
| L185Azi  before UV O1 after UV | 1 | 0 |
| L185Azi  before UV O1+STIM1 before TG | 0,99291 | 0 |
| L185Azi  before UV O1+STIM1  after TG | 0,0371 | 1 |
| L185Azi  before UV L130Azi before UV | 1 | 0 |
| L185Azi  before UV L130Azi after UV | 0,03209 | 1 |
| L185Azi  before UV H134Azi before UV | 0,99997 | 0 |
| L185Azi  before UV H134Azi after UV | 1 | 0 |
| L185Azi  before UV F136Azi before UV | 1 | 0 |
| L185Azi  before UV F136Azi after UV | 1 | 0 |
| L185Azi  before UV A137Azi before UV | 0,01594 | 1 |
| L185Azi  before UV A137Azi after UV | 0,02992 | 1 |
| L185Azi  before UV L138Azi before UV | 1 | 0 |
| L185Azi  before UV L138Azi after UV | 0,34442 | 0 |
| L185Azi  before UV L194Azi before UV | 0,98603 | 0 |
| L185Azi  before UV L194Azi after UV | 0,14777 | 0 |
| L185Azi  before UV V191Azi before UV | 0,05955 | 0 |
| L185Azi  before UV V191Azi after UV | 0,02881 | 1 |
| L185Azi  before UV L188Azi before UV | 0,00426 | 1 |
| L185Azi  before UV L188Azi after UV | 0,00305 | 1 |
| L185Azi  before UV F187Azi before UV | 0,98051 | 0 |
| L185Azi  before UV F187Azi after UV | 0,0072 | 1 |
| L185Azi after UV O1 before UV | 1 | 0 |
| L185Azi after UV O1 after UV | 1 | 0 |
| L185Azi after UV O1+STIM1 before TG | 1 | 0 |
| L185Azi after UV O1+STIM1  after TG | 0,04263 | 1 |
| L185Azi after UV L130Azi before UV | 0,98865 | 0 |
| L185Azi after UV L130Azi after UV | 0,08994 | 0 |
| L185Azi after UV H134Azi before UV | 1 | 0 |
| L185Azi after UV H134Azi after UV | 0,99618 | 0 |
| L185Azi after UV F136Azi before UV | 0,9266 | 0 |
| L185Azi after UV F136Azi after UV | 0,99994 | 0 |
| L185Azi after UV A137Azi before UV | 0,0338 | 1 |
| L185Azi after UV A137Azi after UV | 0,06383 | 0 |
| L185Azi after UV L138Azi before UV | 1 | 0 |
| L185Azi after UV L138Azi after UV | 0,99302 | 0 |
| L185Azi after UV L194Azi before UV | 0,68845 | 0 |
| L185Azi after UV L194Azi after UV | 0,84554 | 0 |
| L185Azi after UV V191Azi before UV | 0,11356 | 0 |
| L185Azi after UV V191Azi after UV | 0,99392 | 0 |
| L185Azi after UV L188Azi before UV | 0,01214 | 1 |
| L185Azi after UV L188Azi after UV | 0,88051 | 0 |
| L185Azi after UV F187Azi before UV | 0,66642 | 0 |
| L185Azi after UV F187Azi after UV | 0,00996 | 1 |
| L185Azi after UV L185Azi  before UV | 0,99517 | 0 |
| V181Azi  before UV O1 before UV | 1 | 0 |
| V181Azi  before UV O1 after UV | 1 | 0 |
| V181Azi  before UV O1+STIM1 before TG | 1 | 0 |
| V181Azi  before UV O1+STIM1  after TG | 0,04958 | 1 |
| V181Azi  before UV L130Azi before UV | 0,97203 | 0 |
| V181Azi  before UV L130Azi after UV | 0,26481 | 0 |
| V181Azi  before UV H134Azi before UV | 0,99959 | 0 |
| V181Azi  before UV H134Azi after UV | 0,99009 | 0 |
| V181Azi  before UV F136Azi before UV | 0,93697 | 0 |
| V181Azi  before UV F136Azi after UV | 0,99854 | 0 |
| V181Azi  before UV A137Azi before UV | 0,08046 | 0 |
| V181Azi  before UV A137Azi after UV | 0,15011 | 0 |
| V181Azi  before UV L138Azi before UV | 0,99967 | 0 |
| V181Azi  before UV L138Azi after UV | 1 | 0 |
| V181Azi  before UV L194Azi before UV | 0,75548 | 0 |
| V181Azi  before UV L194Azi after UV | 1 | 0 |
| V181Azi  before UV V191Azi before UV | 0,22919 | 0 |
| V181Azi  before UV V191Azi after UV | 1 | 0 |
| V181Azi  before UV L188Azi before UV | 0,04267 | 1 |
| V181Azi  before UV L188Azi after UV | 1 | 0 |
| V181Azi  before UV F187Azi before UV | 0,735 | 0 |
| V181Azi  before UV F187Azi after UV | 0,01429 | 1 |
| V181Azi  before UV L185Azi  before UV | 0,98661 | 0 |
| V181Azi  before UV L185Azi after UV | 1 | 0 |
| V181Azi after UV O1 before UV | 0,19361 | 0 |
| V181Azi after UV O1 after UV | 0,26963 | 0 |
| V181Azi after UV O1+STIM1 before TG | 0,19647 | 0 |
| V181Azi after UV O1+STIM1  after TG | 0,17684 | 0 |
| V181Azi after UV L130Azi before UV | 0,10114 | 0 |
| V181Azi after UV L130Azi after UV | 1 | 0 |
| V181Azi after UV H134Azi before UV | 0,15505 | 0 |
| V181Azi after UV H134Azi after UV | 0,11974 | 0 |
| V181Azi after UV F136Azi before UV | 0,09351 | 0 |
| V181Azi after UV F136Azi after UV | 0,14221 | 0 |
| V181Azi after UV A137Azi before UV | 1 | 0 |
| V181Azi after UV A137Azi after UV | 1 | 0 |
| V181Azi after UV L138Azi before UV | 0,14942 | 0 |
| V181Azi after UV L138Azi after UV | 0,78079 | 0 |

| | | |
|---|---|---|
| V181Azi after UV L194Azi before UV | 0,06672 | 0 |
| V181Azi after UV L194Azi after UV | 0,88301 | 0 |
| V181Azi after UV V191Azi before UV | 1 | 0 |
| V181Azi after UV V191Azi after UV | 0,51507 | 0 |
| V181Azi after UV L188Azi before UV | 1 | 0 |
| V181Azi after UV L188Azi after UV | 0,61698 | 0 |
| V181Azi after UV F187Azi before UV | 0,06467 | 0 |
| V181Azi after UV F187Azi after UV | 0,24563 | 0 |
| V181Azi after UV L185Azi  before UV | 0,11415 | 0 |
| V181Azi after UV L185Azi after UV | 0,25802 | 0 |
| V181Azi after UV V181Azi  before UV | 0,55383 | 0 |
| S179Azi  before UV O1 before UV | 1 | 0 |
| S179Azi  before UV O1 after UV | 1 | 0 |
| S179Azi  before UV O1+STIM1 before TG | 1 | 0 |
| S179Azi  before UV O1+STIM1  after TG | 0,04232 | 1 |
| S179Azi  before UV L130Azi before UV | 0,80218 | 0 |
| S179Azi  before UV L130Azi after UV | 0,07784 | 0 |
| S179Azi  before UV H134Azi before UV | 0,99989 | 0 |
| S179Azi  before UV H134Azi after UV | 0,78565 | 0 |
| S179Azi  before UV F136Azi before UV | 0,1257 | 0 |
| S179Azi  before UV F136Azi after UV | 0,99402 | 0 |
| S179Azi  before UV A137Azi before UV | 0,03103 | 1 |
| S179Azi  before UV A137Azi after UV | 0,05831 | 0 |
| S179Azi  before UV L138Azi before UV | 1 | 0 |
| S179Azi  before UV L138Azi after UV | 0,96236 | 0 |
| S179Azi  before UV L194Azi before UV | 0,01226 | 1 |
| S179Azi  before UV L194Azi after UV | 0,65013 | 0 |
| S179Azi  before UV V191Azi before UV | 0,10555 | 0 |
| S179Azi  before UV V191Azi after UV | 0,86559 | 0 |
| S179Azi  before UV L188Azi before UV | 0,01057 | 1 |
| S179Azi  before UV L188Azi after UV | 0,33218 | 0 |
| S179Azi  before UV F187Azi before UV | 0,00971 | 1 |
| S179Azi  before UV F187Azi after UV | 0,00974 | 1 |
| S179Azi  before UV L185Azi  before UV | 0,81512 | 0 |
| S179Azi  before UV L185Azi after UV | 1 | 0 |
| S179Azi  before UV V181Azi  before UV | 1 | 0 |
| S179Azi  before UV V181Azi after UV | 0,2318 | 0 |
| S179Azi after UV O1 before UV | 1 | 0 |
| S179Azi after UV O1 after UV | 1 | 0 |
| S179Azi after UV O1+STIM1 before TG | 1 | 0 |
| S179Azi after UV O1+STIM1  after TG | 0,04159 | 1 |
| S179Azi after UV L130Azi before UV | 1 | 0 |
| S179Azi after UV L130Azi after UV | 0,08562 | 0 |
| S179Azi after UV H134Azi before UV | 1 | 0 |
| S179Azi after UV H134Azi after UV | 1 | 0 |
| S179Azi after UV F136Azi before UV | 1 | 0 |
| S179Azi after UV F136Azi after UV | 1 | 0 |
| S179Azi after UV A137Azi before UV | 0,03154 | 1 |
| S179Azi after UV A137Azi after UV | 0,06003 | 0 |
| S179Azi after UV L138Azi before UV | 1 | 0 |
| S179Azi after UV L138Azi after UV | 0,99666 | 0 |
| S179Azi after UV L194Azi before UV | 0,99771 | 0 |
| S179Azi after UV L194Azi after UV | 0,89896 | 0 |
| S179Azi after UV V191Azi before UV | 0,1075 | 0 |
| S179Azi after UV V191Azi after UV | 0,99973 | 0 |
| S179Azi after UV L188Azi before UV | 0,0112 | 1 |
| S179Azi after UV L188Azi after UV | 0,97817 | 0 |
| S179Azi after UV F187Azi before UV | 0,99667 | 0 |
| S179Azi after UV F187Azi after UV | 0,00947 | 1 |
| S179Azi after UV L185Azi  before UV | 1 | 0 |
| S179Azi after UV L185Azi after UV | 1 | 0 |
| S179Azi after UV V181Azi  before UV | 1 | 0 |
| S179Azi after UV V181Azi after UV | 0,24798 | 0 |
| S179Azi after UV S179Azi  before UV | 1 | 0 |
| W176Azi  before UV O1 before UV | 1 | 0 |
| W176Azi  before UV O1 after UV | 1 | 0 |
| W176Azi  before UV O1+STIM1 before TG | 0,99096 | 0 |
| W176Azi  before UV O1+STIM1  after TG | 0,03631 | 1 |
| W176Azi  before UV L130Azi before UV | 1 | 0 |
| W176Azi  before UV L130Azi after UV | 0,02821 | 1 |
| W176Azi  before UV H134Azi before UV | 0,99996 | 0 |
| W176Azi  before UV H134Azi after UV | 1 | 0 |
| W176Azi  before UV F136Azi before UV | 1 | 0 |
| W176Azi  before UV F136Azi after UV | 1 | 0 |
| W176Azi  before UV A137Azi before UV | 0,01441 | 1 |
| W176Azi  before UV A137Azi after UV | 0,02706 | 1 |
| W176Azi  before UV L138Azi before UV | 1 | 0 |
| W176Azi  before UV L138Azi after UV | 0,28967 | 0 |
| W176Azi  before UV L194Azi before UV | 1 | 0 |
| W176Azi  before UV L194Azi after UV | 0,12239 | 0 |
| W176Azi  before UV V191Azi before UV | 0,05458 | 0 |
| W176Azi  before UV V191Azi after UV | 0,02311 | 1 |
| W176Azi  before UV L188Azi before UV | 0,00371 | 1 |
| W176Azi  before UV L188Azi after UV | 0,0021 | 1 |
| W176Azi  before UV F187Azi before UV | 1 | 0 |
| W176Azi  before UV F187Azi after UV | 0,00686 | 1 |
| W176Azi  before UV L185Azi  before UV | 1 | 0 |
| W176Azi  before UV L185Azi after UV | 0,99039 | 0 |
| W176Azi  before UV V181Azi  before UV | 0,97469 | 0 |
| W176Azi  before UV V181Azi after UV | 0,10262 | 0 |
| W176Azi  before UV S179Azi  before UV | 0,82085 | 0 |
| W176Azi  before UV S179Azi after UV | 1 | 0 |
| W176Azi after UV O1 before UV | 0,67947 | 0 |
| W176Azi after UV O1 after UV | 0,79216 | 0 |
| W176Azi after UV O1+STIM1 before TG | 0,68095 | 0 |
| W176Azi after UV O1+STIM1  after TG | 0,11488 | 0 |
| W176Azi after UV L130Azi before UV | 0,46098 | 0 |
| W176Azi after UV L130Azi after UV | 1 | 0 |
| W176Azi after UV H134Azi before UV | 0,59819 | 0 |
| W176Azi after UV H134Azi after UV | 0,51242 | 0 |
| W176Azi after UV F136Azi before UV | 0,43672 | 0 |
| W176Azi after UV F136Azi after UV | 0,56883 | 0 |
| W176Azi after UV A137Azi before UV | 0,98869 | 0 |
| W176Azi after UV A137Azi after UV | 0,99937 | 0 |
| W176Azi after UV L138Azi before UV | 0,58717 | 0 |
| W176Azi after UV L138Azi after UV | 0,99818 | 0 |
| W176Azi after UV L194Azi before UV | 0,34623 | 0 |
| W176Azi after UV L194Azi after UV | 0,99983 | 0 |
| W176Azi after UV V191Azi before UV | 0,99923 | 0 |
| W176Azi after UV V191Azi after UV | 0,96112 | 0 |
| W176Azi after UV L188Azi before UV | 0,99905 | 0 |
| W176Azi after UV L188Azi after UV | 0,98477 | 0 |
| W176Azi after UV F187Azi before UV | 0,33866 | 0 |
| W176Azi after UV F187Azi after UV | 0,10334 | 0 |
| W176Azi after UV L185Azi  before UV | 0,49759 | 0 |
| W176Azi after UV L185Azi after UV | 0,77619 | 0 |
| W176Azi after UV V181Azi  before UV | 0,97164 | 0 |

| | | |
|---|---|---|
| W176Azi after UV V181Azi after UV | 1 | 0 |
| W176Azi after UV S179Azi  before UV | 0,73853 | 0 |
| W176Azi after UV S179Azi after UV | 0,7649 | 0 |
| W176Azi after UV W176Azi  before UV | 0,46533 | 0 |
| L174Azi  before UV O1 before UV | 1 | 0 |
| L174Azi  before UV O1 after UV | 0,99766 | 0 |
| L174Azi  before UV O1+STIM1 before TG | 0,40011 | 0 |
| L174Azi  before UV O1+STIM1  after TG | 0,03504 | 1 |
| L174Azi  before UV L130Azi before UV | 1 | 0 |
| L174Azi  before UV L130Azi after UV | 0,02114 | 1 |
| L174Azi  before UV H134Azi before UV | 0,48773 | 0 |
| L174Azi  before UV H134Azi after UV | 0,99808 | 0 |
| L174Azi  before UV F136Azi before UV | 1 | 0 |
| L174Azi  before UV F136Azi after UV | 0,75411 | 0 |
| L174Azi  before UV A137Azi before UV | 0,01182 | 1 |
| L174Azi  before UV A137Azi after UV | 0,0221 | 1 |
| L174Azi  before UV L138Azi before UV | 0,99995 | 0 |
| L174Azi  before UV L138Azi after UV | 0,11545 | 0 |
| L174Azi  before UV L194Azi before UV | 1 | 0 |
| L174Azi  before UV L194Azi after UV | 0,05897 | 0 |
| L174Azi  before UV V191Azi before UV | 0,04579 | 1 |
| L174Azi  before UV V191Azi after UV | 0,00106 | 1 |
| L174Azi  before UV L188Azi before UV | 0,00284 | 1 |
| L174Azi  before UV L188Azi after UV | 7,19E-05 | 1 |
| L174Azi  before UV F187Azi before UV | 1 | 0 |
| L174Azi  before UV F187Azi after UV | 0,00631 | 1 |
| L174Azi  before UV L185Azi  before UV | 0,99996 | 0 |
| L174Azi  before UV L185Azi after UV | 0,83836 | 0 |
| L174Azi  before UV V181Azi  before UV | 0,87368 | 0 |
| L174Azi  before UV V181Azi after UV | 0,08084 | 0 |
| L174Azi  before UV S179Azi  before UV | 0,04323 | 1 |
| L174Azi  before UV S179Azi after UV | 0,99991 | 0 |
| L174Azi  before UV W176Azi  before UV | 1 | 0 |
| L174Azi  before UV W176Azi after UV | 0,39584 | 0 |
| L174Azi after UV O1 before UV | 0,95028 | 0 |
| L174Azi after UV O1 after UV | 0,99639 | 0 |
| L174Azi after UV O1+STIM1 before TG | 0,79763 | 0 |
| L174Azi after UV O1+STIM1  after TG | 0,05673 | 0 |
| L174Azi after UV L130Azi before UV | 0,22789 | 0 |
| L174Azi after UV L130Azi after UV | 0,44852 | 0 |
| L174Azi after UV H134Azi before UV | 0,52397 | 0 |
| L174Azi after UV H134Azi after UV | 0,29449 | 0 |
| L174Azi after UV F136Azi before UV | 0,15108 | 0 |
| L174Azi after UV F136Azi after UV | 0,43708 | 0 |
| L174Azi after UV A137Azi before UV | 0,13985 | 0 |
| L174Azi after UV A137Azi after UV | 0,25031 | 0 |
| L174Azi after UV L138Azi before UV | 0,5936 | 0 |
| L174Azi after UV L138Azi after UV | 1 | 0 |
| L174Azi after UV L194Azi before UV | 0,0599 | 0 |
| L174Azi after UV L194Azi after UV | 1 | 0 |
| L174Azi after UV V191Azi before UV | 0,34904 | 0 |
| L174Azi after UV V191Azi after UV | 1 | 0 |
| L174Azi after UV L188Azi before UV | 0,08969 | 0 |
| L174Azi after UV L188Azi after UV | 1 | 0 |
| L174Azi after UV F187Azi before UV | 0,05512 | 0 |
| L174Azi after UV F187Azi after UV | 0,01926 | 1 |
| L174Azi after UV L185Azi  before UV | 0,28075 | 0 |
| L174Azi after UV L185Azi after UV | 0,98355 | 0 |
| L174Azi after UV V181Azi  before UV | 1 | 0 |
| L174Azi after UV V181Azi after UV | 0,75967 | 0 |
| L174Azi after UV S179Azi  before UV | 0,91489 | 0 |
| L174Azi after UV S179Azi after UV | 0,99304 | 0 |
| L174Azi after UV W176Azi  before UV | 0,23721 | 0 |
| L174Azi after UV W176Azi after UV | 0,99742 | 0 |
| L174Azi after UV L174Azi  before UV | 0,10209 | 0 |
| A235Azi before UV O1 before UV | 1 | 0 |
| A235Azi before UV O1 after UV | 1 | 0 |
| A235Azi before UV O1+STIM1 before TG | 0,99994 | 0 |
| A235Azi before UV O1+STIM1  after TG | 0,04609 | 1 |
| A235Azi before UV L130Azi before UV | 0,48422 | 0 |
| A235Azi before UV L130Azi after UV | 0,13863 | 0 |
| A235Azi before UV H134Azi before UV | 0,94771 | 0 |
| A235Azi before UV H134Azi after UV | 0,54537 | 0 |
| A235Azi before UV F136Azi before UV | 0,15348 | 0 |
| A235Azi before UV F136Azi after UV | 0,85128 | 0 |
| A235Azi before UV A137Azi before UV | 0,04851 | 1 |
| A235Azi before UV A137Azi after UV | 0,09087 | 0 |
| A235Azi before UV L138Azi before UV | 0,99081 | 0 |
| A235Azi before UV L138Azi after UV | 0,99999 | 0 |
| A235Azi before UV L194Azi before UV | 0,02443 | 1 |
| A235Azi before UV L194Azi after UV | 0,98114 | 0 |
| A235Azi before UV V191Azi before UV | 0,15324 | 0 |
| A235Azi before UV V191Azi after UV | 1 | 0 |
| A235Azi before UV L188Azi before UV | 0,01974 | 1 |
| A235Azi before UV L188Azi after UV | 0,99948 | 0 |
| A235Azi before UV F187Azi before UV | 0,02088 | 1 |
| A235Azi before UV F187Azi after UV | 0,01188 | 1 |
| A235Azi before UV L185Azi  before UV | 0,56033 | 0 |
| A235Azi before UV L185Azi after UV | 1 | 0 |
| A235Azi before UV V181Azi  before UV | 1 | 0 |
| A235Azi before UV V181Azi after UV | 0,35744 | 0 |
| A235Azi before UV S179Azi  before UV | 1 | 0 |
| A235Azi before UV S179Azi after UV | 1 | 0 |
| A235Azi before UV W176Azi  before UV | 0,5109 | 0 |
| A235Azi before UV W176Azi after UV | 0,87819 | 0 |
| A235Azi before UV L174Azi  before UV | 0,07046 | 0 |
| A235Azi before UV L174Azi after UV | 0,99993 | 0 |
| A235Azi after UV O1 before UV | 1 | 0 |
| A235Azi after UV O1 after UV | 1 | 0 |
| A235Azi after UV O1+STIM1 before TG | 1 | 0 |
| A235Azi after UV O1+STIM1  after TG | 0,04557 | 1 |
| A235Azi after UV L130Azi before UV | 0,63485 | 0 |
| A235Azi after UV L130Azi after UV | 0,13022 | 0 |
| A235Azi after UV H134Azi before UV | 0,98491 | 0 |
| A235Azi after UV H134Azi after UV | 0,71248 | 0 |
| A235Azi after UV F136Azi before UV | 0,2632 | 0 |
| A235Azi after UV F136Azi after UV | 0,93931 | 0 |
| A235Azi after UV A137Azi before UV | 0,04601 | 1 |
| A235Azi after UV A137Azi after UV | 0,08632 | 0 |
| A235Azi after UV L138Azi before UV | 0,99773 | 0 |
| A235Azi after UV L138Azi after UV | 0,99996 | 0 |
| A235Azi after UV L194Azi before UV | 0,0504 | 0 |
| A235Azi after UV L194Azi after UV | 0,97164 | 0 |
| A235Azi after UV V191Azi before UV | 0,14675 | 0 |
| A235Azi after UV V191Azi after UV | 1 | 0 |
| A235Azi after UV L188Azi before UV | 0,01836 | 1 |
| A235Azi after UV L188Azi after UV | 0,99836 | 0 |

| | | |
|---|---|---|
| A235Azi after UV F187Azi before UV | 0,0437 | 1 |
| A235Azi after UV F187Azi after UV | 0,01158 | 1 |
| A235Azi after UV L185Azi  before UV | 0,71163 | 0 |
| A235Azi after UV L185Azi after UV | 1 | 0 |
| A235Azi after UV V181Azi  before UV | 1 | 0 |
| A235Azi after UV V181Azi after UV | 0,34142 | 0 |
| A235Azi after UV S179Azi  before UV | 1 | 0 |
| A235Azi after UV S179Azi after UV | 1 | 0 |
| A235Azi after UV W176Azi  before UV | 0,6587 | 0 |
| A235Azi after UV W176Azi after UV | 0,86509 | 0 |
| A235Azi after UV L174Azi  before UV | 0,13298 | 0 |
| A235Azi after UV L174Azi after UV | 0,99976 | 0 |
| A235Azi after UV A235Azi before UV | 1 | 0 |
| S239Azi before UV O1 before UV | 1 | 0 |
| S239Azi before UV O1 after UV | 1 | 0 |
| S239Azi before UV O1+STIM1 before TG | 1 | 0 |
| S239Azi before UV O1+STIM1  after TG | 0,03867 | 1 |
| S239Azi before UV L130Azi before UV | 1 | 0 |
| S239Azi before UV L130Azi after UV | 0,05044 | 0 |
| S239Azi before UV H134Azi before UV | 1 | 0 |
| S239Azi before UV H134Azi after UV | 1 | 0 |
| S239Azi before UV F136Azi before UV | 1 | 0 |
| S239Azi before UV F136Azi after UV | 1 | 0 |
| S239Azi before UV A137Azi before UV | 0,02122 | 1 |
| S239Azi before UV A137Azi after UV | 0,04032 | 1 |
| S239Azi before UV L138Azi before UV | 1 | 0 |
| S239Azi before UV L138Azi after UV | 0,88075 | 0 |
| S239Azi before UV L194Azi before UV | 1 | 0 |
| S239Azi before UV L194Azi after UV | 0,53642 | 0 |
| S239Azi before UV V191Azi before UV | 0,07677 | 0 |
| S239Azi before UV V191Azi after UV | 0,87352 | 0 |
| S239Azi before UV L188Azi before UV | 0,00642 | 1 |
| S239Azi before UV L188Azi after UV | 0,51734 | 0 |
| S239Azi before UV F187Azi before UV | 1 | 0 |
| S239Azi before UV F187Azi after UV | 0,00799 | 1 |
| S239Azi before UV L185Azi  before UV | 1 | 0 |
| S239Azi before UV L185Azi after UV | 1 | 0 |
| S239Azi before UV V181Azi  before UV | 0,99997 | 0 |
| S239Azi before UV V181Azi after UV | 0,16396 | 0 |
| S239Azi before UV S179Azi  before UV | 1 | 0 |
| S239Azi before UV S179Azi after UV | 1 | 0 |
| S239Azi before UV W176Azi  before UV | 1 | 0 |
| S239Azi before UV W176Azi after UV | 0,62139 | 0 |
| S239Azi before UV L174Azi  before UV | 1 | 0 |
| S239Azi before UV L174Azi after UV | 0,8272 | 0 |
| S239Azi before UV A235Azi before UV | 0,99991 | 0 |
| S239Azi before UV A235Azi after UV | 0,99998 | 0 |
| S239Azi after UV O1 before UV | 0,25159 | 0 |
| S239Azi after UV O1 after UV | 0,4442 | 0 |
| S239Azi after UV O1+STIM1 before TG | 0,10235 | 0 |
| S239Azi after UV O1+STIM1  after TG | 0,07487 | 0 |
| S239Azi after UV L130Azi before UV | 0,01311 | 1 |
| S239Azi after UV L130Azi after UV | 0,91792 | 0 |
| S239Azi after UV H134Azi before UV | 0,04449 | 1 |
| S239Azi after UV H134Azi after UV | 0,01931 | 1 |
| S239Azi after UV F136Azi before UV | 0,00855 | 1 |
| S239Azi after UV F136Azi after UV | 0,03355 | 1 |
| S239Azi after UV A137Azi before UV | 0,42659 | 0 |
| S239Azi after UV A137Azi after UV | 0,64261 | 0 |
| S239Azi after UV L138Azi before UV | 0,05152 | 0 |
| S239Azi after UV L138Azi after UV | 1 | 0 |
| S239Azi after UV L194Azi before UV | 0,003 | 1 |
| S239Azi after UV L194Azi after UV | 1 | 0 |
| S239Azi after UV V191Azi before UV | 0,72401 | 0 |
| S239Azi after UV V191Azi after UV | 0,91867 | 0 |
| S239Azi after UV L188Azi before UV | 0,41839 | 0 |
| S239Azi after UV L188Azi after UV | 0,98944 | 0 |
| S239Azi after UV F187Azi before UV | 0,00273 | 1 |
| S239Azi after UV F187Azi after UV | 0,0362 | 1 |
| S239Azi after UV L185Azi  before UV | 0,01784 | 1 |
| S239Azi after UV L185Azi after UV | 0,31491 | 0 |
| S239Azi after UV V181Azi  before UV | 0,98908 | 0 |
| S239Azi after UV V181Azi after UV | 0,99318 | 0 |
| S239Azi after UV S179Azi  before UV | 0,16367 | 0 |
| S239Azi after UV S179Azi after UV | 0,36825 | 0 |
| S239Azi after UV W176Azi  before UV | 0,01382 | 1 |
| S239Azi after UV W176Azi after UV | 1 | 0 |
| S239Azi after UV L174Azi  before UV | 0,00543 | 1 |
| S239Azi after UV L174Azi after UV | 0,99999 | 0 |
| S239Azi after UV A235Azi before UV | 0,56862 | 0 |
| S239Azi after UV A235Azi after UV | 0,52431 | 0 |
| S239Azi after UV S239Azi before UV | 0,11325 | 0 |
| M243Azi before UV O1 before UV | 0,99971 | 0 |
| M243Azi before UV O1 after UV | 1 | 0 |
| M243Azi before UV O1+STIM1 before TG | 0,99942 | 0 |
| M243Azi before UV O1+STIM1  after TG | 0,0558 | 0 |
| M243Azi before UV L130Azi before UV | 0,95896 | 0 |
| M243Azi before UV L130Azi after UV | 0,55439 | 0 |
| M243Azi before UV H134Azi before UV | 0,99502 | 0 |
| M243Azi before UV H134Azi after UV | 0,97768 | 0 |
| M243Azi before UV F136Azi before UV | 0,93969 | 0 |
| M243Azi before UV F136Azi after UV | 0,99116 | 0 |
| M243Azi before UV A137Azi before UV | 0,17365 | 0 |
| M243Azi before UV A137Azi after UV | 0,30561 | 0 |
| M243Azi before UV L138Azi before UV | 0,99496 | 0 |
| M243Azi before UV L138Azi after UV | 1 | 0 |
| M243Azi before UV L194Azi before UV | 0,85348 | 0 |
| M243Azi before UV L194Azi after UV | 1 | 0 |
| M243Azi before UV V191Azi before UV | 0,39764 | 0 |
| M243Azi before UV V191Azi after UV | 1 | 0 |
| M243Azi before UV L188Azi before UV | 0,13957 | 0 |
| M243Azi before UV L188Azi after UV | 1 | 0 |
| M243Azi before UV F187Azi before UV | 0,8441 | 0 |
| M243Azi before UV F187Azi after UV | 0,01927 | 1 |
| M243Azi before UV L185Azi  before UV | 0,97338 | 0 |
| M243Azi before UV L185Azi after UV | 1 | 0 |
| M243Azi before UV V181Azi  before UV | 1 | 0 |
| M243Azi before UV V181Azi after UV | 0,83115 | 0 |
| M243Azi before UV S179Azi  before UV | 0,99992 | 0 |
| M243Azi before UV S179Azi after UV | 1 | 0 |
| M243Azi before UV W176Azi  before UV | 0,96117 | 0 |
| M243Azi before UV W176Azi after UV | 0,99898 | 0 |
| M243Azi before UV L174Azi  before UV | 0,90717 | 0 |
| M243Azi before UV L174Azi after UV | 1 | 0 |
| M243Azi before UV A235Azi before UV | 1 | 0 |
| M243Azi before UV A235Azi after UV | 1 | 0 |
| M243Azi before UV S239Azi before UV | 0,99834 | 0 |

| | | |
|---|---|---|
| M243Azi before UV S239Azi after UV | 1 | 0 |
| M243Azi after UV O1 before UV | 0,91142 | 0 |
| M243Azi after UV O1 after UV | 0,99255 | 0 |
| M243Azi after UV O1+STIM1 before TG | 0,43738 | 0 |
| M243Azi after UV O1+STIM1  after TG | 0,05391 | 0 |
| M243Azi after UV L130Azi before UV | 0,04959 | 1 |
| M243Azi after UV L130Azi after UV | 0,32389 | 0 |
| M243Azi after UV H134Azi before UV | 0,17354 | 0 |
| M243Azi after UV H134Azi after UV | 0,07266 | 0 |
| M243Azi after UV F136Azi before UV | 0,03193 | 1 |
| M243Azi after UV F136Azi after UV | 0,12893 | 0 |
| M243Azi after UV A137Azi before UV | 0,10255 | 0 |
| M243Azi after UV A137Azi after UV | 0,18665 | 0 |
| M243Azi after UV L138Azi before UV | 0,25664 | 0 |
| M243Azi after UV L138Azi after UV | 1 | 0 |
| M243Azi after UV L194Azi before UV | 0,0106 | 1 |
| M243Azi after UV L194Azi after UV | 1 | 0 |
| M243Azi after UV V191Azi before UV | 0,27743 | 0 |
| M243Azi after UV V191Azi after UV | 1 | 0 |
| M243Azi after UV L188Azi before UV | 0,05619 | 0 |
| M243Azi after UV L188Azi after UV | 1 | 0 |
| M243Azi after UV F187Azi before UV | 0,00939 | 1 |
| M243Azi after UV F187Azi after UV | 0,01698 | 1 |
| M243Azi after UV L185Azi  before UV | 0,07066 | 0 |
| M243Azi after UV L185Azi after UV | 0,93828 | 0 |
| M243Azi after UV V181Azi  before UV | 1 | 0 |
| M243Azi after UV V181Azi after UV | 0,6332 | 0 |
| M243Azi after UV S179Azi  before UV | 0,63993 | 0 |
| M243Azi after UV S179Azi after UV | 0,98722 | 0 |
| M243Azi after UV W176Azi  before UV | 0,05371 | 0 |
| M243Azi after UV W176Azi after UV | 0,98709 | 0 |
| M243Azi after UV L174Azi  before UV | 0,01992 | 1 |
| M243Azi after UV L174Azi after UV | 1 | 0 |
| M243Azi after UV A235Azi before UV | 0,99967 | 0 |
| M243Azi after UV A235Azi after UV | 0,99904 | 0 |
| M243Azi after UV S239Azi before UV | 0,66105 | 0 |
| M243Azi after UV S239Azi after UV | 0,99434 | 0 |
| M243Azi after UV M243Azi before UV | 1 | 0 |
| P245Azi before UV O1 before UV | 0,94131 | 0 |
| P245Azi before UV O1 after UV | 0,95662 | 0 |
| P245Azi before UV O1+STIM1 before TG | 0,94835 | 0 |
| P245Azi before UV O1+STIM1  after TG | 0,62066 | 0 |
| P245Azi before UV L130Azi before UV | 0,91763 | 0 |
| P245Azi before UV L130Azi after UV | 1 | 0 |
| P245Azi before UV H134Azi before UV | 0,93867 | 0 |
| P245Azi before UV H134Azi after UV | 0,92682 | 0 |
| P245Azi before UV F136Azi before UV | 0,9148 | 0 |
| P245Azi before UV F136Azi after UV | 0,93482 | 0 |
| P245Azi before UV A137Azi before UV | 1 | 0 |
| P245Azi before UV A137Azi after UV | 1 | 0 |
| P245Azi before UV L138Azi before UV | 0,93551 | 0 |
| P245Azi before UV L138Azi after UV | 0,99172 | 0 |
| P245Azi before UV L194Azi before UV | 0,89684 | 0 |
| P245Azi before UV L194Azi after UV | 0,99525 | 0 |
| P245Azi before UV V191Azi before UV | 1 | 0 |
| P245Azi before UV V191Azi after UV | 0,98192 | 0 |
| P245Azi before UV L188Azi before UV | 1 | 0 |
| P245Azi before UV L188Azi after UV | 0,98705 | 0 |
| P245Azi before UV F187Azi before UV | 0,89508 | 0 |
| P245Azi before UV F187Azi after UV | 0,98597 | 0 |
| P245Azi before UV L185Azi  before UV | 0,92415 | 0 |
| P245Azi before UV L185Azi after UV | 0,95726 | 0 |
| P245Azi before UV V181Azi  before UV | 0,98067 | 0 |
| P245Azi before UV V181Azi after UV | 1 | 0 |
| P245Azi before UV S179Azi  before UV | 0,95516 | 0 |
| P245Azi before UV S179Azi after UV | 0,95333 | 0 |
| P245Azi before UV W176Azi  before UV | 0,91835 | 0 |
| P245Azi before UV W176Azi after UV | 1 | 0 |
| P245Azi before UV L174Azi  before UV | 0,90722 | 0 |
| P245Azi before UV L174Azi after UV | 0,99122 | 0 |
| P245Azi before UV A235Azi before UV | 0,9701 | 0 |
| P245Azi before UV A235Azi after UV | 0,96842 | 0 |
| P245Azi before UV S239Azi before UV | 0,93662 | 0 |
| P245Azi before UV S239Azi after UV | 0,99911 | 0 |
| P245Azi before UV M243Azi before UV | 0,99127 | 0 |
| P245Azi before UV M243Azi after UV | 0,98741 | 0 |
| P245Azi after UV O1 before UV | 0,99577 | 0 |
| P245Azi after UV O1 after UV | 0,99794 | 0 |
| P245Azi after UV O1+STIM1 before TG | 0,99682 | 0 |
| P245Azi after UV O1+STIM1  after TG | 0,277 | 0 |
| P245Azi after UV L130Azi before UV | 0,99055 | 0 |
| P245Azi after UV L130Azi after UV | 1 | 0 |
| P245Azi after UV H134Azi before UV | 0,99521 | 0 |
| P245Azi after UV H134Azi after UV | 0,99278 | 0 |
| P245Azi after UV F136Azi before UV | 0,98977 | 0 |
| P245Azi after UV F136Azi after UV | 0,99448 | 0 |
| P245Azi after UV A137Azi before UV | 1 | 0 |
| P245Azi after UV A137Azi after UV | 1 | 0 |
| P245Azi after UV L138Azi before UV | 0,99464 | 0 |
| P245Azi after UV L138Azi after UV | 0,99997 | 0 |
| P245Azi after UV L194Azi before UV | 0,98428 | 0 |
| P245Azi after UV L194Azi after UV | 1 | 0 |
| P245Azi after UV V191Azi before UV | 1 | 0 |
| P245Azi after UV V191Azi after UV | 0,99977 | 0 |
| P245Azi after UV L188Azi before UV | 1 | 0 |
| P245Azi after UV L188Azi after UV | 0,9999 | 0 |
| P245Azi after UV F187Azi before UV | 0,98368 | 0 |
| P245Azi after UV F187Azi after UV | 0,63024 | 0 |
| P245Azi after UV L185Azi  before UV | 0,99217 | 0 |
| P245Azi after UV L185Azi after UV | 0,99799 | 0 |
| P245Azi after UV V181Azi  before UV | 0,99973 | 0 |
| P245Azi after UV V181Azi after UV | 1 | 0 |
| P245Azi after UV S179Azi  before UV | 0,99773 | 0 |
| P245Azi after UV S179Azi after UV | 0,99754 | 0 |
| P245Azi after UV W176Azi  before UV | 0,99074 | 0 |
| P245Azi after UV W176Azi after UV | 1 | 0 |
| P245Azi after UV L174Azi  before UV | 0,9876 | 0 |
| P245Azi after UV L174Azi after UV | 0,99997 | 0 |
| P245Azi after UV A235Azi before UV | 0,99917 | 0 |
| P245Azi after UV A235Azi after UV | 0,99904 | 0 |
| P245Azi after UV S239Azi before UV | 0,9949 | 0 |
| P245Azi after UV S239Azi after UV | 1 | 0 |
| P245Azi after UV M243Azi before UV | 0,99997 | 0 |
| P245Azi after UV M243Azi after UV | 0,99991 | 0 |
| P245Azi after UV P245Azi before UV | 1 | 0 |
| F250Azi before UV O1 before UV | 0,90385 | 0 |
| F250Azi before UV O1 after UV | 0,94553 | 0 |

| | | |
|---|---|---|
| F250Azi before UV O1+STIM1 before TG | 0,91379 | 0 |
| F250Azi before UV O1+STIM1  after TG | 0,14322 | 0 |
| F250Azi before UV L130Azi before UV | 0,81026 | 0 |
| F250Azi before UV L130Azi after UV | 1 | 0 |
| F250Azi before UV H134Azi before UV | 0,88128 | 0 |
| F250Azi before UV H134Azi after UV | 0,8404 | 0 |
| F250Azi before UV F136Azi before UV | 0,79763 | 0 |
| F250Azi before UV F136Azi after UV | 0,86815 | 0 |
| F250Azi before UV A137Azi before UV | 0,99993 | 0 |
| F250Azi before UV A137Azi after UV | 1 | 0 |
| F250Azi before UV L138Azi before UV | 0,87386 | 0 |
| F250Azi before UV L138Azi after UV | 0,99943 | 0 |
| F250Azi before UV L194Azi before UV | 0,7348 | 0 |
| F250Azi before UV L194Azi after UV | 0,99992 | 0 |
| F250Azi before UV V191Azi before UV | 1 | 0 |
| F250Azi before UV V191Azi after UV | 0,99225 | 0 |
| F250Azi before UV L188Azi before UV | 1 | 0 |
| F250Azi before UV L188Azi after UV | 0,99694 | 0 |
| F250Azi before UV F187Azi before UV | 0,7288 | 0 |
| F250Azi before UV F187Azi after UV | 0,18305 | 0 |
| F250Azi before UV L185Azi  before UV | 0,83187 | 0 |
| F250Azi before UV L185Azi after UV | 0,94346 | 0 |
| F250Azi before UV V181Azi  before UV | 0,99294 | 0 |
| F250Azi before UV V181Azi after UV | 1 | 0 |
| F250Azi before UV S179Azi  before UV | 0,93413 | 0 |
| F250Azi before UV S179Azi after UV | 0,9367 | 0 |
| F250Azi before UV W176Azi  before UV | 0,81278 | 0 |
| F250Azi before UV W176Azi after UV | 1 | 0 |
| F250Azi before UV L174Azi  before UV | 0,77098 | 0 |
| F250Azi before UV L174Azi after UV | 0,99927 | 0 |
| F250Azi before UV A235Azi before UV | 0,97323 | 0 |
| F250Azi before UV A235Azi after UV | 0,9698 | 0 |
| F250Azi before UV S239Azi before UV | 0,88394 | 0 |
| F250Azi before UV S239Azi after UV | 1 | 0 |
| F250Azi before UV M243Azi before UV | 0,99956 | 0 |
| F250Azi before UV M243Azi after UV | 0,9973 | 0 |
| F250Azi before UV P245Azi before UV | 1 | 0 |
| F250Azi before UV P245Azi after UV | 1 | 0 |
| F250Azi after UV O1 before UV | 0,37443 | 0 |
| F250Azi after UV O1 after UV | 0,42902 | 0 |
| F250Azi after UV O1+STIM1 before TG | 0,39187 | 0 |
| F250Azi after UV O1+STIM1  after TG | 0,45101 | 0 |
| F250Azi after UV L130Azi before UV | 0,30611 | 0 |
| F250Azi after UV L130Azi after UV | 1 | 0 |
| F250Azi after UV H134Azi before UV | 0,3599 | 0 |
| F250Azi after UV H134Azi after UV | 0,32731 | 0 |
| F250Azi after UV F136Azi before UV | 0,29921 | 0 |
| F250Azi after UV F136Azi after UV | 0,34866 | 0 |
| F250Azi after UV A137Azi before UV | 1 | 0 |
| F250Azi after UV A137Azi after UV | 1 | 0 |
| F250Azi after UV L138Azi before UV | 0,35187 | 0 |
| F250Azi after UV L138Azi after UV | 0,69332 | 0 |
| F250Azi after UV L194Azi before UV | 0,26435 | 0 |
| F250Azi after UV L194Azi after UV | 0,7586 | 0 |
| F250Azi after UV V191Azi before UV | 1 | 0 |
| F250Azi after UV V191Azi after UV | 0,5733 | 0 |
| F250Azi after UV L188Azi before UV | 1 | 0 |
| F250Azi after UV L188Azi after UV | 0,62359 | 0 |
| F250Azi after UV F187Azi before UV | 0,2613 | 0 |
| F250Azi after UV F187Azi after UV | 0,89186 | 0 |
| F250Azi after UV L185Azi  before UV | 0,32095 | 0 |
| F250Azi after UV L185Azi after UV | 0,42867 | 0 |
| F250Azi after UV V181Azi  before UV | 0,57273 | 0 |
| F250Azi after UV V181Azi after UV | 1 | 0 |
| F250Azi after UV S179Azi  before UV | 0,41745 | 0 |
| F250Azi after UV S179Azi after UV | 0,41556 | 0 |
| F250Azi after UV W176Azi  before UV | 0,30772 | 0 |
| F250Azi after UV W176Azi after UV | 0,99902 | 0 |
| F250Azi after UV L174Azi  before UV | 0,28363 | 0 |
| F250Azi after UV L174Azi after UV | 0,68417 | 0 |
| F250Azi after UV A235Azi before UV | 0,49065 | 0 |
| F250Azi after UV A235Azi after UV | 0,48135 | 0 |
| F250Azi after UV S239Azi before UV | 0,35767 | 0 |
| F250Azi after UV S239Azi after UV | 0,89429 | 0 |
| F250Azi after UV M243Azi before UV | 0,70229 | 0 |
| F250Azi after UV M243Azi after UV | 0,629 | 0 |
| F250Azi after UV P245Azi before UV | 1 | 0 |
| F250Azi after UV P245Azi after UV | 1 | 0 |
| F250Azi after UV F250Azi before UV | 1 | 0 |
| A254Azi before UV O1 before UV | 0,45405 | 0 |
| A254Azi before UV O1 after UV | 0,63836 | 0 |
| A254Azi before UV O1+STIM1 before TG | 0,39052 | 0 |
| A254Azi before UV O1+STIM1  after TG | 0,0901 | 0 |
| A254Azi before UV L130Azi before UV | 0,14103 | 0 |
| A254Azi before UV L130Azi after UV | 0,99875 | 0 |
| A254Azi before UV H134Azi before UV | 0,27151 | 0 |
| A254Azi before UV H134Azi after UV | 0,17985 | 0 |
| A254Azi before UV F136Azi before UV | 0,11816 | 0 |
| A254Azi before UV F136Azi after UV | 0,23709 | 0 |
| A254Azi before UV A137Azi before UV | 0,77729 | 0 |
| A254Azi before UV A137Azi after UV | 0,93039 | 0 |
| A254Azi before UV L138Azi before UV | 0,27079 | 0 |
| A254Azi before UV L138Azi after UV | 0,99983 | 0 |
| A254Azi before UV L194Azi before UV | 0,066 | 0 |
| A254Azi before UV L194Azi after UV | 1 | 0 |
| A254Azi before UV V191Azi before UV | 0,94503 | 0 |
| A254Azi before UV V191Azi after UV | 0,95244 | 0 |
| A254Azi before UV L188Azi before UV | 0,86398 | 0 |
| A254Azi before UV L188Azi after UV | 0,98896 | 0 |
| A254Azi before UV F187Azi before UV | 0,06257 | 0 |
| A254Azi before UV F187Azi after UV | 0,05674 | 0 |
| A254Azi before UV L185Azi  before UV | 0,16873 | 0 |
| A254Azi before UV L185Azi after UV | 0,58098 | 0 |
| A254Azi before UV V181Azi  before UV | 0,97949 | 0 |
| A254Azi before UV V181Azi after UV | 0,99999 | 0 |
| A254Azi before UV S179Azi  before UV | 0,48451 | 0 |
| A254Azi before UV S179Azi after UV | 0,58638 | 0 |
| A254Azi before UV W176Azi  before UV | 0,14455 | 0 |
| A254Azi before UV W176Azi after UV | 1 | 0 |
| A254Azi before UV L174Azi  before UV | 0,09213 | 0 |
| A254Azi before UV L174Azi after UV | 0,99964 | 0 |
| A254Azi before UV A235Azi before UV | 0,7804 | 0 |
| A254Azi before UV A235Azi after UV | 0,75302 | 0 |
| A254Azi before UV S239Azi before UV | 0,34158 | 0 |
| A254Azi before UV S239Azi after UV | 1 | 0 |
| A254Azi before UV M243Azi before UV | 0,99991 | 0 |
| A254Azi before UV M243Azi after UV | 0,99202 | 0 |

| | | | | | | |
|---|---|---|---|---|---|---|
| | | | | A254Azi before UV P245Azi before UV | 0,99991 | 0 |
| | | | | A254Azi before UV P245Azi after UV | 1 | 0 |
| | | | | A254Azi before UV F250Azi before UV | 1 | 0 |
| | | | | A254Azi before UV F250Azi after UV | 0,9754 | 0 |
| | | | | A254Azi after UV O1 before UV | 0 | 1 |
| | | | | A254Azi after UV O1 after UV | 0 | 1 |
| | | | | A254Azi after UV O1+STIM1 before TG | 0 | 1 |
| | | | | A254Azi after UV O1+STIM1 after TG | 0,99939 | 0 |
| | | | | A254Azi after UV L130Azi before UV | 0 | 1 |
| | | | | A254Azi after UV L130Azi after UV | 4,16E-04 | 1 |
| | | | | A254Azi after UV H134Azi before UV | 0 | 1 |
| | | | | A254Azi after UV H134Azi after UV | 2,54E-06 | 1 |
| | | | | A254Azi after UV F136Azi before UV | 0 | 1 |
| | | | | A254Azi after UV F136Azi after UV | 0 | 1 |
| | | | | A254Azi after UV A137Azi before UV | 0,0267 | 1 |
| | | | | A254Azi after UV A137Azi after UV | 0,00809 | 1 |
| | | | | A254Azi after UV L138Azi before UV | 0 | 1 |
| | | | | A254Azi after UV L138Azi after UV | 0 | 1 |
| | | | | A254Azi after UV L194Azi before UV | 0 | 1 |
| | | | | A254Azi after UV L194Azi after UV | 0 | 1 |
| | | | | A254Azi after UV V191Azi before UV | 0,02449 | 1 |
| | | | | A254Azi after UV V191Azi after UV | 0 | 1 |
| | | | | A254Azi after UV L188Azi before UV | 0,00179 | 1 |
| | | | | A254Azi after UV L188Azi after UV | 0 | 1 |
| | | | | A254Azi after UV F187Azi before UV | 0 | 1 |
| | | | | A254Azi after UV F187Azi after UV | 1 | 0 |
| | | | | A254Azi after UV L185Azi before UV | 0 | 1 |
| | | | | A254Azi after UV L185Azi after UV | 0 | 1 |
| | | | | A254Azi after UV V181Azi before UV | 0 | 1 |
| | | | | A254Azi after UV V181Azi after UV | 1,54E-04 | 1 |
| | | | | A254Azi after UV S179Azi before UV | 0 | 1 |
| | | | | A254Azi after UV S179Azi after UV | 0 | 1 |
| | | | | A254Azi after UV W176Azi before UV | 0 | 1 |
| | | | | A254Azi after UV W176Azi after UV | 5,96E-06 | 1 |
| | | | | A254Azi after UV L174Azi before UV | 0 | 1 |
| | | | | A254Azi after UV L174Azi after UV | 0 | 1 |
| | | | | A254Azi after UV A235Azi before UV | 3,89E-06 | 1 |
| | | | | A254Azi after UV A235Azi after UV | 0 | 1 |
| | | | | A254Azi after UV S239Azi before UV | 0 | 1 |
| | | | | A254Azi after UV S239Azi after UV | 0 | 1 |
| | | | | A254Azi after UV M243Azi before UV | 0 | 1 |
| | | | | A254Azi after UV M243Azi after UV | 0 | 1 |
| | | | | A254Azi after UV P245Azi before UV | 0,87089 | 0 |
| | | | | A254Azi after UV P245Azi after UV | 0,15343 | 0 |
| | | | | A254Azi after UV F250Azi before UV | 2,92E-04 | 1 |
| | | | | A254Azi after UV F250Azi after UV | 0,31857 | 0 |
| | | | | A254Azi after UV A254Azi before UV | 2,22E-07 | 1 |
| S1c - 0mM and 2mM Ca2+ solution | Welch-ANOVA | 0 | F(41, 368.33)=7.79 | A235Bpa 0 A235Bpa 2 | 0,91192 | 0 |
| | | | | S239Bpa 2 A235Bpa 2 | 0,99497 | 0 |
| | | | | S239Bpa 2 A235Bpa 0 | 0,35462 | 0 |
| | | | | S239Bpa 0 A235Bpa 2 | 0,99975 | 0 |
| | | | | S239Bpa 0 A235Bpa 0 | 0,21333 | 0 |
| | | | | S239Bpa 0 S239Bpa 2 | 1 | 0 |
| | | | | M243Bpa 2 A235Bpa 2 | 0,96423 | 0 |
| | | | | M243Bpa 2 A235Bpa 0 | 0,94336 | 0 |
| | | | | M243Bpa 2 S239Bpa 2 | 0,99614 | 0 |
| | | | | M243Bpa 2 S239Bpa 0 | 0,81377 | 0 |
| | | | | M243Bpa 0 A235Bpa 2 | 0,91766 | 0 |
| | | | | M243Bpa 0 A235Bpa 0 | 1 | 0 |
| | | | | M243Bpa 0 S239Bpa 2 | 0,45036 | 0 |
| | | | | M243Bpa 0 S239Bpa 0 | 0,25689 | 0 |
| | | | | M243Bpa 0 M243Bpa 2 | 0,98422 | 0 |
| | | | | P245Bpa 2 A235Bpa 2 | 0,99929 | 0 |
| | | | | P245Bpa 2 A235Bpa 0 | 0,0461 | 1 |
| | | | | P245Bpa 2 S239Bpa 2 | 1 | 0 |
| | | | | P245Bpa 2 S239Bpa 0 | 1 | 0 |
| | | | | P245Bpa 2 M243Bpa 2 | 0,57077 | 0 |
| | | | | P245Bpa 2 M243Bpa 0 | 0,06452 | 0 |
| | | | | P245Bpa 0 A235Bpa 2 | 0,97435 | 0 |
| | | | | P245Bpa 0 A235Bpa 0 | 0,99992 | 0 |
| | | | | P245Bpa 0 S239Bpa 2 | 1 | 0 |
| | | | | P245Bpa 0 S239Bpa 0 | 0,9871 | 0 |
| | | | | P245Bpa 0 M243Bpa 2 | 1 | 0 |
| | | | | P245Bpa 0 M243Bpa 0 | 0,99999 | 0 |
| | | | | P245Bpa 0 P245Bpa 2 | 0,97775 | 0 |
| | | | | F250Bpa 2 A235Bpa 2 | 0,52396 | 0 |
| | | | | F250Bpa 2 A235Bpa 0 | 0,24714 | 0 |
| | | | | F250Bpa 2 S239Bpa 2 | 0,2909 | 0 |
| | | | | F250Bpa 2 S239Bpa 0 | 0,3181 | 0 |
| | | | | F250Bpa 2 M243Bpa 2 | 0,26444 | 0 |
| | | | | F250Bpa 2 M243Bpa 0 | 0,24855 | 0 |
| | | | | F250Bpa 2 P245Bpa 2 | 0,31085 | 0 |
| | | | | F250Bpa 2 P245Bpa 0 | 0,26828 | 0 |
| | | | | F250Bpa 0 A235Bpa 2 | 0,99948 | 0 |
| | | | | F250Bpa 0 A235Bpa 0 | 0,83849 | 0 |
| | | | | F250Bpa 0 S239Bpa 2 | 1 | 0 |
| | | | | F250Bpa 0 S239Bpa 0 | 1 | 0 |
| | | | | F250Bpa 0 M243Bpa 2 | 0,99783 | 0 |
| | | | | F250Bpa 0 M243Bpa 0 | 0,87173 | 0 |
| | | | | F250Bpa 0 P245Bpa 2 | 1 | 0 |
| | | | | F250Bpa 0 P245Bpa 0 | 0,99998 | 0 |
| | | | | F250Bpa 0 F250Bpa 2 | 0,30999 | 0 |
| | | | | A254Bpa 2 A235Bpa 2 | 1,70E-04 | 1 |
| | | | | A254Bpa 2 A235Bpa 0 | 6,70E-06 | 1 |
| | | | | A254Bpa 2 S239Bpa 2 | 1,19E-05 | 1 |
| | | | | A254Bpa 2 S239Bpa 0 | 1,66E-05 | 1 |
| | | | | A254Bpa 2 M243Bpa 2 | 8,50E-06 | 1 |
| | | | | A254Bpa 2 M243Bpa 0 | 6,83E-06 | 1 |
| | | | | A254Bpa 2 P245Bpa 2 | 1,52E-05 | 1 |
| | | | | A254Bpa 2 P245Bpa 0 | 8,88E-06 | 1 |
| | | | | A254Bpa 2 F250Bpa 2 | 1 | 0 |
| | | | | A254Bpa 2 F250Bpa 0 | 1,50E-05 | 1 |
| | | | | A254Bpa 0 A235Bpa 2 | 0,99652 | 0 |
| | | | | A254Bpa 0 A235Bpa 0 | 0,99586 | 0 |
| | | | | A254Bpa 0 S239Bpa 2 | 1 | 0 |
| | | | | A254Bpa 0 S239Bpa 0 | 1 | 0 |
| | | | | A254Bpa 0 M243Bpa 2 | 1 | 0 |
| | | | | A254Bpa 0 M243Bpa 0 | 0,99785 | 0 |
| | | | | A254Bpa 0 P245Bpa 2 | 1 | 0 |
| | | | | A254Bpa 0 P245Bpa 0 | 1 | 0 |
| | | | | A254Bpa 0 F250Bpa 2 | 0,29138 | 0 |
| | | | | A254Bpa 0 F250Bpa 0 | 1 | 0 |
| | | | | A254Bpa 0 A254Bpa 2 | 1,19E-05 | 1 |
| | | | | L194Bpa 2 A235Bpa 2 | 1 | 0 |
| | | | | L194Bpa 2 A235Bpa 0 | 0,98836 | 0 |

| | | |
|---|---|---|
| L194Bpa 2 S239Bpa 2 | 1 | 0 |
| L194Bpa 2 S239Bpa 0 | 1 | 0 |
| L194Bpa 2 M243Bpa 2 | 0,99889 | 0 |
| L194Bpa 2 M243Bpa 0 | 0,99015 | 0 |
| L194Bpa 2 P245Bpa 2 | 1 | 0 |
| L194Bpa 2 P245Bpa 0 | 0,9996 | 0 |
| L194Bpa 2 F250Bpa 2 | 0,38933 | 0 |
| L194Bpa 2 F250Bpa 0 | 1 | 0 |
| L194Bpa 2 A254Bpa 2 | 3,80E-05 | 1 |
| L194Bpa 2 A254Bpa 0 | 1 | 0 |
| L194Bpa 0 A235Bpa 2 | 1 | 0 |
| L194Bpa 0 A235Bpa 0 | 0,94636 | 0 |
| L194Bpa 0 S239Bpa 2 | 1 | 0 |
| L194Bpa 0 S239Bpa 0 | 1 | 0 |
| L194Bpa 0 M243Bpa 2 | 0,99332 | 0 |
| L194Bpa 0 M243Bpa 0 | 0,95348 | 0 |
| L194Bpa 0 P245Bpa 2 | 1 | 0 |
| L194Bpa 0 P245Bpa 0 | 0,99782 | 0 |
| L194Bpa 0 F250Bpa 2 | 0,37118 | 0 |
| L194Bpa 0 F250Bpa 0 | 1 | 0 |
| L194Bpa 0 A254Bpa 2 | 3,03E-05 | 1 |
| L194Bpa 0 A254Bpa 0 | 1 | 0 |
| L194Bpa 0 L194Bpa 2 | 1 | 0 |
| V191Bpa 2 A235Bpa 2 | 0,92567 | 0 |
| V191Bpa 2 A235Bpa 0 | 1 | 0 |
| V191Bpa 2 S239Bpa 2 | 0,46738 | 0 |
| V191Bpa 2 S239Bpa 0 | 0,2834 | 0 |
| V191Bpa 2 M243Bpa 2 | 0,98122 | 0 |
| V191Bpa 2 M243Bpa 0 | 1 | 0 |
| V191Bpa 2 P245Bpa 2 | 0,0692 | 0 |
| V191Bpa 2 P245Bpa 0 | 1 | 0 |
| V191Bpa 2 F250Bpa 2 | 0,25095 | 0 |
| V191Bpa 2 F250Bpa 0 | 0,89674 | 0 |
| V191Bpa 2 A254Bpa 2 | 7,00E-06 | 1 |
| V191Bpa 2 A254Bpa 0 | 0,99895 | 0 |
| V191Bpa 2 L194Bpa 2 | 0,99227 | 0 |
| V191Bpa 2 L194Bpa 0 | 0,96162 | 0 |
| V191Bpa 0 A235Bpa 2 | 0,95203 | 0 |
| V191Bpa 0 A235Bpa 0 | 0,99999 | 0 |
| V191Bpa 0 S239Bpa 2 | 0,96694 | 0 |
| V191Bpa 0 S239Bpa 0 | 0,66955 | 0 |
| V191Bpa 0 M243Bpa 2 | 1 | 0 |
| V191Bpa 0 M243Bpa 0 | 1 | 0 |
| V191Bpa 0 P245Bpa 2 | 0,38981 | 0 |
| V191Bpa 0 P245Bpa 0 | 1 | 0 |
| V191Bpa 0 F250Bpa 2 | 0,25904 | 0 |
| V191Bpa 0 F250Bpa 0 | 0,98927 | 0 |
| V191Bpa 0 A254Bpa 2 | 7,89E-06 | 1 |
| V191Bpa 0 A254Bpa 0 | 1 | 0 |
| V191Bpa 0 L194Bpa 2 | 0,99756 | 0 |
| V191Bpa 0 L194Bpa 0 | 0,98647 | 0 |
| V191Bpa 0 V191Bpa 2 | 1 | 0 |
| L188Bpa 2 A235Bpa 2 | 0,01419 | 1 |
| L188Bpa 2 A235Bpa 0 | 0,00635 | 1 |
| L188Bpa 2 S239Bpa 2 | 0,00749 | 1 |
| L188Bpa 2 S239Bpa 0 | 0,00822 | 1 |
| L188Bpa 2 M243Bpa 2 | 0,0068 | 1 |
| L188Bpa 2 M243Bpa 0 | 0,00639 | 1 |
| L188Bpa 2 P245Bpa 2 | 0,00803 | 1 |
| L188Bpa 2 P245Bpa 0 | 0,00689 | 1 |
| L188Bpa 2 F250Bpa 2 | 0,99676 | 0 |
| L188Bpa 2 F250Bpa 0 | 0,00798 | 1 |
| L188Bpa 2 A254Bpa 2 | 0,9794 | 0 |
| L188Bpa 2 A254Bpa 0 | 0,00748 | 1 |
| L188Bpa 2 L194Bpa 2 | 0,00998 | 1 |
| L188Bpa 2 L194Bpa 0 | 0,00957 | 1 |
| L188Bpa 2 V191Bpa 2 | 0,00645 | 1 |
| L188Bpa 2 V191Bpa 0 | 0,00665 | 1 |
| L188Bpa 0 A235Bpa 2 | 0,93063 | 0 |
| L188Bpa 0 A235Bpa 0 | 1 | 0 |
| L188Bpa 0 S239Bpa 2 | 0,68128 | 0 |
| L188Bpa 0 S239Bpa 0 | 0,37816 | 0 |
| L188Bpa 0 M243Bpa 2 | 0,99991 | 0 |
| L188Bpa 0 M243Bpa 0 | 1 | 0 |
| L188Bpa 0 P245Bpa 2 | 0,12798 | 0 |
| L188Bpa 0 P245Bpa 0 | 1 | 0 |
| L188Bpa 0 F250Bpa 2 | 0,25206 | 0 |
| L188Bpa 0 F250Bpa 0 | 0,93305 | 0 |
| L188Bpa 0 A254Bpa 2 | 7,11E-06 | 1 |
| L188Bpa 0 A254Bpa 0 | 0,99966 | 0 |
| L188Bpa 0 L194Bpa 2 | 0,99359 | 0 |
| L188Bpa 0 L194Bpa 0 | 0,96794 | 0 |
| L188Bpa 0 V191Bpa 2 | 1 | 0 |
| L188Bpa 0 V191Bpa 0 | 1 | 0 |
| L188Bpa 0 L188Bpa 2 | 0,00648 | 1 |
| F187Azi 2 A235Bpa 2 | 0,99565 | 0 |
| F187Azi 2 A235Bpa 0 | 0,05934 | 0 |
| F187Azi 2 S239Bpa 2 | 1 | 0 |
| F187Azi 2 S239Bpa 0 | 1 | 0 |
| F187Azi 2 M243Bpa 2 | 0,92141 | 0 |
| F187Azi 2 M243Bpa 0 | 0,1004 | 0 |
| F187Azi 2 P245Bpa 2 | 1 | 0 |
| F187Azi 2 P245Bpa 0 | 0,99998 | 0 |
| F187Azi 2 F250Bpa 2 | 0,29312 | 0 |
| F187Azi 2 F250Bpa 0 | 1 | 0 |
| F187Azi 2 A254Bpa 2 | 1,23E-05 | 1 |
| F187Azi 2 A254Bpa 0 | 1 | 0 |
| F187Azi 2 L194Bpa 2 | 1 | 0 |
| F187Azi 2 L194Bpa 0 | 1 | 0 |
| F187Azi 2 V191Bpa 2 | 0,08716 | 0 |
| F187Azi 2 V191Bpa 0 | 0,75882 | 0 |
| F187Azi 2 L188Bpa 2 | 0,00756 | 1 |
| F187Azi 2 L188Bpa 0 | 0,25047 | 0 |
| F187Azi 0 A235Bpa 2 | 0,9123 | 0 |
| F187Azi 0 A235Bpa 0 | 1 | 0 |
| F187Azi 0 S239Bpa 2 | 0,28471 | 0 |
| F187Azi 0 S239Bpa 0 | 0,19239 | 0 |
| F187Azi 0 M243Bpa 2 | 0,80779 | 0 |
| F187Azi 0 M243Bpa 0 | 1 | 0 |
| F187Azi 0 P245Bpa 2 | 0,03648 | 1 |
| F187Azi 0 P245Bpa 0 | 0,99983 | 0 |
| F187Azi 0 F250Bpa 2 | 0,24743 | 0 |
| F187Azi 0 F250Bpa 0 | 0,82551 | 0 |
| F187Azi 0 A254Bpa 2 | 6,73E-06 | 1 |
| F187Azi 0 A254Bpa 0 | 0,99506 | 0 |
| F187Azi 0 L194Bpa 2 | 0,98838 | 0 |
| F187Azi 0 L194Bpa 0 | 0,94587 | 0 |

| | | | | | |
|---|---|---|---|---|---|
| | | | F187Azi 0 V191Bpa 2 | 1 | 0 |
| | | | F187Azi 0 V191Bpa 0 | 0,99988 | 0 |
| | | | F187Azi 0 L188Bpa 2 | 0,00636 | 1 |
| | | | F187Azi 0 L188Bpa 0 | 1 | 0 |
| | | | F187Azi 0 F187Azi 2 | 0,03296 | 1 |
| | | | L185Bpa 2 A235Bpa 2 | 0,97485 | 0 |
| | | | L185Bpa 2 A235Bpa 0 | 0,99738 | 0 |
| | | | L185Bpa 2 S239Bpa 2 | 1 | 0 |
| | | | L185Bpa 2 S239Bpa 0 | 0,98007 | 0 |
| | | | L185Bpa 2 M243Bpa 2 | 1 | 0 |
| | | | L185Bpa 2 M243Bpa 0 | 0,99936 | 0 |
| | | | L185Bpa 2 P245Bpa 2 | 0,95856 | 0 |
| | | | L185Bpa 2 P245Bpa 0 | 1 | 0 |
| | | | L185Bpa 2 F250Bpa 2 | 0,26916 | 0 |
| | | | L185Bpa 2 F250Bpa 0 | 0,99996 | 0 |
| | | | L185Bpa 2 A254Bpa 2 | 9,00E-06 | 1 |
| | | | L185Bpa 2 A254Bpa 0 | 1 | 0 |
| | | | L185Bpa 2 L194Bpa 2 | 0,99961 | 0 |
| | | | L185Bpa 2 L194Bpa 0 | 0,99777 | 0 |
| | | | L185Bpa 2 V191Bpa 2 | 0,99975 | 0 |
| | | | L185Bpa 2 V191Bpa 0 | 1 | 0 |
| | | | L185Bpa 2 L188Bpa 2 | 0,00691 | 1 |
| | | | L185Bpa 2 L188Bpa 0 | 1 | 0 |
| | | | L185Bpa 2 F187Azi 2 | 0,99993 | 0 |
| | | | L185Bpa 2 F187Azi 0 | 0,99402 | 0 |
| | | | L185Bpa 0 A235Bpa 2 | 0,97882 | 0 |
| | | | L185Bpa 0 A235Bpa 0 | 0,98658 | 0 |
| | | | L185Bpa 0 S239Bpa 2 | 1 | 0 |
| | | | L185Bpa 0 S239Bpa 0 | 0,99166 | 0 |
| | | | L185Bpa 0 M243Bpa 2 | 1 | 0 |
| | | | L185Bpa 0 M243Bpa 0 | 0,99536 | 0 |
| | | | L185Bpa 0 P245Bpa 2 | 0,98324 | 0 |
| | | | L185Bpa 0 P245Bpa 0 | 1 | 0 |
| | | | L185Bpa 0 F250Bpa 2 | 0,27183 | 0 |
| | | | L185Bpa 0 F250Bpa 0 | 1 | 0 |
| | | | L185Bpa 0 A254Bpa 2 | 9,32E-06 | 1 |
| | | | L185Bpa 0 A254Bpa 0 | 1 | 0 |
| | | | L185Bpa 0 L194Bpa 2 | 0,99977 | 0 |
| | | | L185Bpa 0 L194Bpa 0 | 0,99864 | 0 |
| | | | L185Bpa 0 V191Bpa 2 | 0,99742 | 0 |
| | | | L185Bpa 0 V191Bpa 0 | 1 | 0 |
| | | | L185Bpa 0 L188Bpa 2 | 0,00698 | 1 |
| | | | L185Bpa 0 L188Bpa 0 | 0,99989 | 0 |
| | | | L185Bpa 0 F187Azi 2 | 1 | 0 |
| | | | L185Bpa 0 F187Azi 0 | 0,97401 | 0 |
| | | | L185Bpa 0 L185Bpa 2 | 1 | 0 |
| | | | V181Bpa 2 A235Bpa 2 | 0,67416 | 0 |
| | | | V181Bpa 2 A235Bpa 0 | 0,04574 | 1 |
| | | | V181Bpa 2 S239Bpa 2 | 0,08249 | 0 |
| | | | V181Bpa 2 S239Bpa 0 | 0,11429 | 0 |
| | | | V181Bpa 2 M243Bpa 2 | 0,05833 | 0 |
| | | | V181Bpa 2 M243Bpa 0 | 0,0467 | 1 |
| | | | V181Bpa 2 P245Bpa 2 | 0,10445 | 0 |
| | | | V181Bpa 2 P245Bpa 0 | 0,06197 | 0 |
| | | | V181Bpa 2 F250Bpa 2 | 0,99871 | 0 |
| | | | V181Bpa 2 F250Bpa 0 | 0,10574 | 0 |
| | | | V181Bpa 2 A254Bpa 2 | 0,4046 | 0 |
| | | | V181Bpa 2 A254Bpa 0 | 0,0848 | 0 |
| | | | V181Bpa 2 L194Bpa 2 | 0,27365 | 0 |
| | | | V181Bpa 2 L194Bpa 0 | 0,21786 | 0 |
| | | | V181Bpa 2 V191Bpa 2 | 0,04828 | 1 |
| | | | V181Bpa 2 V191Bpa 0 | 0,05423 | 0 |
| | | | V181Bpa 2 L188Bpa 2 | 0,13963 | 0 |
| | | | V181Bpa 2 L188Bpa 0 | 0,04913 | 1 |
| | | | V181Bpa 2 F187Azi 2 | 0,0845 | 0 |
| | | | V181Bpa 2 F187Azi 0 | 0,04589 | 1 |
| | | | V181Bpa 2 L185Bpa 2 | 0,0625 | 0 |
| | | | V181Bpa 2 L185Bpa 0 | 0,06475 | 0 |
| | | | V181Bpa 0 A235Bpa 2 | 0,99478 | 0 |
| | | | V181Bpa 0 A235Bpa 0 | 0,77848 | 0 |
| | | | V181Bpa 0 S239Bpa 2 | 1 | 0 |
| | | | V181Bpa 0 S239Bpa 0 | 1 | 0 |
| | | | V181Bpa 0 M243Bpa 2 | 0,99996 | 0 |
| | | | V181Bpa 0 M243Bpa 0 | 0,84235 | 0 |
| | | | V181Bpa 0 P245Bpa 2 | 1 | 0 |
| | | | V181Bpa 0 P245Bpa 0 | 1 | 0 |
| | | | V181Bpa 0 F250Bpa 2 | 0,2896 | 0 |
| | | | V181Bpa 0 F250Bpa 0 | 1 | 0 |
| | | | V181Bpa 0 A254Bpa 2 | 1,17E-05 | 1 |
| | | | V181Bpa 0 A254Bpa 0 | 1 | 0 |
| | | | V181Bpa 0 L194Bpa 2 | 1 | 0 |
| | | | V181Bpa 0 L194Bpa 0 | 1 | 0 |
| | | | V181Bpa 0 V191Bpa 2 | 0,87055 | 0 |
| | | | V181Bpa 0 V191Bpa 0 | 0,99841 | 0 |
| | | | V181Bpa 0 L188Bpa 2 | 0,00745 | 1 |
| | | | V181Bpa 0 L188Bpa 0 | 0,94478 | 0 |
| | | | V181Bpa 0 F187Azi 2 | 1 | 0 |
| | | | V181Bpa 0 F187Azi 0 | 0,73374 | 0 |
| | | | V181Bpa 0 L185Bpa 2 | 1 | 0 |
| | | | V181Bpa 0 L185Bpa 0 | 1 | 0 |
| | | | V181Bpa 0 V181Bpa 2 | 0,08158 | 0 |
| | | | S179Bpa 2 A235Bpa 2 | 0,98886 | 0 |
| | | | S179Bpa 2 A235Bpa 0 | 0,80497 | 0 |
| | | | S179Bpa 2 S239Bpa 2 | 1 | 0 |
| | | | S179Bpa 2 S239Bpa 0 | 0,99991 | 0 |
| | | | S179Bpa 2 M243Bpa 2 | 1 | 0 |
| | | | S179Bpa 2 M243Bpa 0 | 0,8736 | 0 |
| | | | S179Bpa 2 P245Bpa 2 | 0,9999 | 0 |
| | | | S179Bpa 2 P245Bpa 0 | 1 | 0 |
| | | | S179Bpa 2 F250Bpa 2 | 0,28088 | 0 |
| | | | S179Bpa 2 F250Bpa 0 | 1 | 0 |
| | | | S179Bpa 2 A254Bpa 2 | 1,05E-05 | 1 |
| | | | S179Bpa 2 A254Bpa 0 | 1 | 0 |
| | | | S179Bpa 2 L194Bpa 2 | 0,99997 | 0 |
| | | | S179Bpa 2 L194Bpa 0 | 0,99982 | 0 |
| | | | S179Bpa 2 V191Bpa 2 | 0,89091 | 0 |
| | | | S179Bpa 2 V191Bpa 0 | 0,99984 | 0 |
| | | | S179Bpa 2 L188Bpa 2 | 0,00722 | 1 |
| | | | S179Bpa 2 L188Bpa 0 | 0,96896 | 0 |
| | | | S179Bpa 2 F187Azi 2 | 1 | 0 |
| | | | S179Bpa 2 F187Azi 0 | 0,74161 | 0 |
| | | | S179Bpa 2 L185Bpa 2 | 1 | 0 |
| | | | S179Bpa 2 L185Bpa 0 | 1 | 0 |
| | | | S179Bpa 2 V181Bpa 2 | 0,07283 | 0 |
| | | | S179Bpa 2 V181Bpa 0 | 1 | 0 |
| | | | S179Bpa 0 A235Bpa 2 | 0,96484 | 0 |
| | | | S179Bpa 0 A235Bpa 0 | 1 | 0 |

| | | |
|---|---|---|
| S179Bpa 0 S239Bpa 2 | 0,99988 | 0 |
| S179Bpa 0 S239Bpa 0 | 0,92996 | 0 |
| S179Bpa 0 M243Bpa 2 | 1 | 0 |
| S179Bpa 0 M243Bpa 0 | 1 | 0 |
| S179Bpa 0 P245Bpa 2 | 0,86405 | 0 |
| S179Bpa 0 P245Bpa 0 | 1 | 0 |
| S179Bpa 0 F250Bpa 2 | 0,26347 | 0 |
| S179Bpa 0 F250Bpa 0 | 0,99942 | 0 |
| S179Bpa 0 A254Bpa 2 | 8,33E-06 | 1 |
| S179Bpa 0 A254Bpa 0 | 1 | 0 |
| S179Bpa 0 L194Bpa 2 | 0,99901 | 0 |
| S179Bpa 0 L194Bpa 0 | 0,99457 | 0 |
| S179Bpa 0 V191Bpa 2 | 1 | 0 |
| S179Bpa 0 V191Bpa 0 | 1 | 0 |
| S179Bpa 0 L188Bpa 2 | 0,00676 | 1 |
| S179Bpa 0 L188Bpa 0 | 1 | 0 |
| S179Bpa 0 F187Azi 2 | 0,99666 | 0 |
| S179Bpa 0 F187Azi 0 | 1 | 0 |
| S179Bpa 0 L185Bpa 2 | 1 | 0 |
| S179Bpa 0 L185Bpa 0 | 1 | 0 |
| S179Bpa 0 V181Bpa 2 | 0,05797 | 0 |
| S179Bpa 0 V181Bpa 0 | 1 | 0 |
| S179Bpa 0 S179Bpa 2 | 1 | 0 |
| W176Bpa 2 A235Bpa 2 | 0,00196 | 1 |
| W176Bpa 2 A235Bpa 0 | 1,39E-04 | 1 |
| W176Bpa 2 S239Bpa 2 | 2,26E-04 | 1 |
| W176Bpa 2 S239Bpa 0 | 2,97E-04 | 1 |
| W176Bpa 2 M243Bpa 2 | 1,70E-04 | 1 |
| W176Bpa 2 M243Bpa 0 | 1,41E-04 | 1 |
| W176Bpa 2 P245Bpa 2 | 2,77E-04 | 1 |
| W176Bpa 2 P245Bpa 0 | 1,77E-04 | 1 |
| W176Bpa 2 F250Bpa 2 | 1 | 0 |
| W176Bpa 2 F250Bpa 0 | 2,74E-04 | 1 |
| W176Bpa 2 A254Bpa 2 | 1 | 0 |
| W176Bpa 2 A254Bpa 0 | 2,26E-04 | 1 |
| W176Bpa 2 L194Bpa 2 | 5,91E-04 | 1 |
| W176Bpa 2 L194Bpa 0 | 4,93E-04 | 1 |
| W176Bpa 2 V191Bpa 2 | 1,45E-04 | 1 |
| W176Bpa 2 V191Bpa 0 | 1,60E-04 | 1 |
| W176Bpa 2 L188Bpa 2 | 0,97531 | 0 |
| W176Bpa 2 L188Bpa 0 | 1,47E-04 | 1 |
| W176Bpa 2 F187Azi 2 | 2,31E-04 | 1 |
| W176Bpa 2 F187Azi 0 | 1,40E-04 | 1 |
| W176Bpa 2 L185Bpa 2 | 1,79E-04 | 1 |
| W176Bpa 2 L185Bpa 0 | 1,84E-04 | 1 |
| W176Bpa 2 V181Bpa 2 | 0,64555 | 0 |
| W176Bpa 2 V181Bpa 0 | 2,22E-04 | 1 |
| W176Bpa 2 S179Bpa 2 | 2,03E-04 | 1 |
| W176Bpa 2 S179Bpa 0 | 1,68E-04 | 1 |
| W176Bpa 0 A235Bpa 2 | 0,95 | 0 |
| W176Bpa 0 A235Bpa 0 | 1 | 0 |
| W176Bpa 0 S239Bpa 2 | 0,98022 | 0 |
| W176Bpa 0 S239Bpa 0 | 0,703 | 0 |
| W176Bpa 0 M243Bpa 2 | 1 | 0 |
| W176Bpa 0 M243Bpa 0 | 1 | 0 |
| W176Bpa 0 P245Bpa 2 | 0,46434 | 0 |
| W176Bpa 0 P245Bpa 0 | 1 | 0 |
| W176Bpa 0 F250Bpa 2 | 0,25789 | 0 |
| W176Bpa 0 F250Bpa 0 | 0,99013 | 0 |
| W176Bpa 0 A254Bpa 2 | 7,76E-06 | 1 |
| W176Bpa 0 A254Bpa 0 | 1 | 0 |
| W176Bpa 0 L194Bpa 2 | 0,99733 | 0 |
| W176Bpa 0 L194Bpa 0 | 0,9857 | 0 |
| W176Bpa 0 V191Bpa 2 | 1 | 0 |
| W176Bpa 0 V191Bpa 0 | 1 | 0 |
| W176Bpa 0 L188Bpa 2 | 0,00662 | 1 |
| W176Bpa 0 L188Bpa 0 | 1 | 0 |
| W176Bpa 0 F187Azi 2 | 0,85024 | 0 |
| W176Bpa 0 F187Azi 0 | 1 | 0 |
| W176Bpa 0 L185Bpa 2 | 1 | 0 |
| W176Bpa 0 L185Bpa 0 | 1 | 0 |
| W176Bpa 0 V181Bpa 2 | 0,05349 | 0 |
| W176Bpa 0 V181Bpa 0 | 0,99892 | 0 |
| W176Bpa 0 S179Bpa 2 | 0,99994 | 0 |
| W176Bpa 0 S179Bpa 0 | 1 | 0 |
| W176Bpa 0 W176Bpa 2 | 1,57E-04 | 1 |
| L174Bpa 2 A235Bpa 2 | 0,99989 | 0 |
| L174Bpa 2 A235Bpa 0 | 1 | 0 |
| L174Bpa 2 S239Bpa 2 | 1 | 0 |
| L174Bpa 2 S239Bpa 0 | 1 | 0 |
| L174Bpa 2 M243Bpa 2 | 1 | 0 |
| L174Bpa 2 M243Bpa 0 | 1 | 0 |
| L174Bpa 2 P245Bpa 2 | 1 | 0 |
| L174Bpa 2 P245Bpa 0 | 1 | 0 |
| L174Bpa 2 F250Bpa 2 | 0,31071 | 0 |
| L174Bpa 2 F250Bpa 0 | 1 | 0 |
| L174Bpa 2 A254Bpa 2 | 1,50E-05 | 1 |
| L174Bpa 2 A254Bpa 0 | 1 | 0 |
| L174Bpa 2 L194Bpa 2 | 1 | 0 |
| L174Bpa 2 L194Bpa 0 | 1 | 0 |
| L174Bpa 2 V191Bpa 2 | 1 | 0 |
| L174Bpa 2 V191Bpa 0 | 1 | 0 |
| L174Bpa 2 L188Bpa 2 | 0,00787 | 1 |
| L174Bpa 2 L188Bpa 0 | 1 | 0 |
| L174Bpa 2 F187Azi 2 | 1 | 0 |
| L174Bpa 2 F187Azi 0 | 1 | 0 |
| L174Bpa 2 L185Bpa 2 | 1 | 0 |
| L174Bpa 2 L185Bpa 0 | 1 | 0 |
| L174Bpa 2 V181Bpa 2 | 0,11764 | 0 |
| L174Bpa 2 V181Bpa 0 | 1 | 0 |
| L174Bpa 2 S179Bpa 2 | 1 | 0 |
| L174Bpa 2 S179Bpa 0 | 1 | 0 |
| L174Bpa 2 W176Bpa 2 | 2,75E-04 | 1 |
| L174Bpa 2 W176Bpa 0 | 1 | 0 |
| L174Bpa 0 A235Bpa 2 | 0,94663 | 0 |
| L174Bpa 0 A235Bpa 0 | 1 | 0 |
| L174Bpa 0 S239Bpa 2 | 0,916 | 0 |
| L174Bpa 0 S239Bpa 0 | 0,57932 | 0 |
| L174Bpa 0 M243Bpa 2 | 1 | 0 |
| L174Bpa 0 M243Bpa 0 | 1 | 0 |
| L174Bpa 0 P245Bpa 2 | 0,28724 | 0 |
| L174Bpa 0 P245Bpa 0 | 1 | 0 |
| L174Bpa 0 F250Bpa 2 | 0,25714 | 0 |
| L174Bpa 0 F250Bpa 0 | 0,97986 | 0 |
| L174Bpa 0 A254Bpa 2 | 7,69E-06 | 1 |
| L174Bpa 0 A254Bpa 0 | 0,99999 | 0 |
| L174Bpa 0 L194Bpa 2 | 0,99675 | 0 |

| | | |
|---|---|---|
| L174Bpa 0 L194Bpa 0 | 0,9824 | 0 |
| L174Bpa 0 V191Bpa 2 | 1 | 0 |
| L174Bpa 0 V191Bpa 0 | 1 | 0 |
| L174Bpa 0 L188Bpa 2 | 0,00661 | 1 |
| L174Bpa 0 L188Bpa 0 | 1 | 0 |
| L174Bpa 0 F187Azi 2 | 0,5924 | 0 |
| L174Bpa 0 F187Azi 0 | 0,99997 | 0 |
| L174Bpa 0 L185Bpa 2 | 1 | 0 |
| L174Bpa 0 L185Bpa 0 | 1 | 0 |
| L174Bpa 0 V181Bpa 2 | 0,05279 | 0 |
| L174Bpa 0 V181Bpa 0 | 0,99393 | 0 |
| L174Bpa 0 S179Bpa 2 | 0,99871 | 0 |
| L174Bpa 0 S179Bpa 0 | 1 | 0 |
| L174Bpa 0 W176Bpa 2 | 1,56E-04 | 1 |
| L174Bpa 0 W176Bpa 0 | 1 | 0 |
| L174Bpa 0 L174Bpa 2 | 1 | 0 |
| L130Bpa 2 A235Bpa 2 | 1 | 0 |
| L130Bpa 2 A235Bpa 0 | 0,96545 | 0 |
| L130Bpa 2 S239Bpa 2 | 1 | 0 |
| L130Bpa 2 S239Bpa 0 | 1 | 0 |
| L130Bpa 2 M243Bpa 2 | 0,99936 | 0 |
| L130Bpa 2 M243Bpa 0 | 0,97313 | 0 |
| L130Bpa 2 P245Bpa 2 | 1 | 0 |
| L130Bpa 2 P245Bpa 0 | 0,99996 | 0 |
| L130Bpa 2 F250Bpa 2 | 0,33214 | 0 |
| L130Bpa 2 F250Bpa 0 | 1 | 0 |
| L130Bpa 2 A254Bpa 2 | 1,93E-05 | 1 |
| L130Bpa 2 A254Bpa 0 | 1 | 0 |
| L130Bpa 2 L194Bpa 2 | 1 | 0 |
| L130Bpa 2 L194Bpa 0 | 1 | 0 |
| L130Bpa 2 V191Bpa 2 | 0,98038 | 0 |
| L130Bpa 2 V191Bpa 0 | 0,99739 | 0 |
| L130Bpa 2 L188Bpa 2 | 0,00854 | 1 |
| L130Bpa 2 L188Bpa 0 | 0,98629 | 0 |
| L130Bpa 2 F187Azi 2 | 1 | 0 |
| L130Bpa 2 F187Azi 0 | 0,9642 | 0 |
| L130Bpa 2 L185Bpa 2 | 0,99996 | 0 |
| L130Bpa 2 L185Bpa 0 | 0,99999 | 0 |
| L130Bpa 2 V181Bpa 2 | 0,14033 | 0 |
| L130Bpa 2 V181Bpa 0 | 1 | 0 |
| L130Bpa 2 S179Bpa 2 | 1 | 0 |
| L130Bpa 2 S179Bpa 0 | 0,99963 | 0 |
| L130Bpa 2 W176Bpa 2 | 3,39E-04 | 1 |
| L130Bpa 2 W176Bpa 0 | 0,99721 | 0 |
| L130Bpa 2 L174Bpa 2 | 1 | 0 |
| L130Bpa 2 L174Bpa 0 | 0,99559 | 0 |
| L130Bpa 0 A235Bpa 2 | 0,99534 | 0 |
| L130Bpa 0 A235Bpa 0 | 0,35114 | 0 |
| L130Bpa 0 S239Bpa 2 | 1 | 0 |
| L130Bpa 0 S239Bpa 0 | 1 | 0 |
| L130Bpa 0 M243Bpa 2 | 0,99474 | 0 |
| L130Bpa 0 M243Bpa 0 | 0,44344 | 0 |
| L130Bpa 0 P245Bpa 2 | 1 | 0 |
| L130Bpa 0 P245Bpa 0 | 1 | 0 |
| L130Bpa 0 F250Bpa 2 | 0,29173 | 0 |
| L130Bpa 0 F250Bpa 0 | 1 | 0 |
| L130Bpa 0 A254Bpa 2 | 1,21E-05 | 1 |
| L130Bpa 0 A254Bpa 0 | 1 | 0 |
| L130Bpa 0 L194Bpa 2 | 1 | 0 |
| L130Bpa 0 L194Bpa 0 | 1 | 0 |
| L130Bpa 0 V191Bpa 2 | 0,46178 | 0 |
| L130Bpa 0 V191Bpa 0 | 0,96076 | 0 |
| L130Bpa 0 L188Bpa 2 | 0,00751 | 1 |
| L130Bpa 0 L188Bpa 0 | 0,66834 | 0 |
| L130Bpa 0 F187Azi 2 | 1 | 0 |
| L130Bpa 0 F187Azi 0 | 0,28514 | 0 |
| L130Bpa 0 L185Bpa 2 | 1 | 0 |
| L130Bpa 0 L185Bpa 0 | 1 | 0 |
| L130Bpa 0 V181Bpa 2 | 0,08336 | 0 |
| L130Bpa 0 V181Bpa 0 | 1 | 0 |
| L130Bpa 0 S179Bpa 2 | 1 | 0 |
| L130Bpa 0 S179Bpa 0 | 0,99981 | 0 |
| L130Bpa 0 W176Bpa 2 | 2,28E-04 | 1 |
| L130Bpa 0 W176Bpa 0 | 0,97591 | 0 |
| L130Bpa 0 L174Bpa 2 | 1 | 0 |
| L130Bpa 0 L174Bpa 0 | 0,90579 | 0 |
| L130Bpa 0 L130Bpa 2 | 1 | 0 |
| H134Bpa 2 A235Bpa 2 | 0,99723 | 0 |
| H134Bpa 2 A235Bpa 0 | 0,90988 | 0 |
| H134Bpa 2 S239Bpa 2 | 1 | 0 |
| H134Bpa 2 S239Bpa 0 | 1 | 0 |
| H134Bpa 2 M243Bpa 2 | 0,99992 | 0 |
| H134Bpa 2 M243Bpa 0 | 0,93593 | 0 |
| H134Bpa 2 P245Bpa 2 | 1 | 0 |
| H134Bpa 2 P245Bpa 0 | 1 | 0 |
| H134Bpa 2 F250Bpa 2 | 0,29515 | 0 |
| H134Bpa 2 F250Bpa 0 | 1 | 0 |
| H134Bpa 2 A254Bpa 2 | 1,25E-05 | 1 |
| H134Bpa 2 A254Bpa 0 | 1 | 0 |
| H134Bpa 2 L194Bpa 2 | 1 | 0 |
| H134Bpa 2 L194Bpa 0 | 1 | 0 |
| H134Bpa 2 V191Bpa 2 | 0,95149 | 0 |
| H134Bpa 2 V191Bpa 0 | 0,99876 | 0 |
| H134Bpa 2 L188Bpa 2 | 0,00759 | 1 |
| H134Bpa 2 L188Bpa 0 | 0,97611 | 0 |
| H134Bpa 2 F187Azi 2 | 1 | 0 |
| H134Bpa 2 F187Azi 0 | 0,89626 | 0 |
| H134Bpa 2 L185Bpa 2 | 1 | 0 |
| H134Bpa 2 L185Bpa 0 | 1 | 0 |
| H134Bpa 2 V181Bpa 2 | 0,08811 | 0 |
| H134Bpa 2 V181Bpa 0 | 1 | 0 |
| H134Bpa 2 S179Bpa 2 | 1 | 0 |
| H134Bpa 2 S179Bpa 0 | 1 | 0 |
| H134Bpa 2 W176Bpa 2 | 2,35E-04 | 1 |
| H134Bpa 2 W176Bpa 0 | 0,99899 | 0 |
| H134Bpa 2 L174Bpa 2 | 1 | 0 |
| H134Bpa 2 L174Bpa 0 | 0,99644 | 0 |
| H134Bpa 2 L130Bpa 2 | 1 | 0 |
| H134Bpa 2 L130Bpa 0 | 1 | 0 |
| H134Bpa 0 A235Bpa 2 | 0,98277 | 0 |
| H134Bpa 0 A235Bpa 0 | 0,23562 | 0 |
| H134Bpa 0 S239Bpa 2 | 1 | 0 |
| H134Bpa 0 S239Bpa 0 | 0,99038 | 0 |
| H134Bpa 0 M243Bpa 2 | 1 | 0 |
| H134Bpa 0 M243Bpa 0 | 0,38402 | 0 |
| H134Bpa 0 P245Bpa 2 | 0,96829 | 0 |
| H134Bpa 0 P245Bpa 0 | 1 | 0 |

| | | | | | |
|---|---|---|---|---|---|
| | | | H134Bpa 0 F250Bpa  2 | 0,27594 | 0 |
| | | | H134Bpa 0 F250Bpa 0 | 1 | 0 |
| | | | H134Bpa 0 A254Bpa 2 | 9,89E-06 | 1 |
| | | | H134Bpa 0 A254Bpa 0 | 1 | 0 |
| | | | H134Bpa 0 L194Bpa 2 | 0,99987 | 0 |
| | | | H134Bpa 0 L194Bpa 0 | 0,99914 | 0 |
| | | | H134Bpa 0 V191Bpa 2 | 0,29093 | 0 |
| | | | H134Bpa 0 V191Bpa 0 | 0,99872 | 0 |
| | | | H134Bpa 0 L188Bpa 2 | 0,0071 | 1 |
| | | | H134Bpa 0 L188Bpa 0 | 0,74357 | 0 |
| | | | H134Bpa 0 F187Azi 2 | 1 | 0 |
| | | | H134Bpa 0 F187Azi 0 | 0,10667 | 0 |
| | | | H134Bpa 0 L185Bpa 2 | 1 | 0 |
| | | | H134Bpa 0 L185Bpa 0 | 1 | 0 |
| | | | H134Bpa 0 V181Bpa 2 | 0,06793 | 0 |
| | | | H134Bpa 0 V181Bpa 0 | 1 | 0 |
| | | | H134Bpa 0 S179Bpa 2 | 1 | 0 |
| | | | H134Bpa 0 S179Bpa 0 | 1 | 0 |
| | | | H134Bpa 0 W176Bpa 2 | 1,93E-04 | 1 |
| | | | H134Bpa 0 W176Bpa 0 | 0,99975 | 0 |
| | | | H134Bpa 0 L174Bpa 2 | 1 | 0 |
| | | | H134Bpa 0 L174Bpa 0 | 0,98397 | 0 |
| | | | H134Bpa 0 L130Bpa 2 | 1 | 0 |
| | | | H134Bpa 0 L130Bpa 0 | 1 | 0 |
| | | | H134Bpa 0 H134Bpa 2 | 1 | 0 |
| | | | F136Bpa 2 A235Bpa 2 | 1 | 0 |
| | | | F136Bpa 2 A235Bpa 0 | 0,09524 | 0 |
| | | | F136Bpa 2 S239Bpa 2 | 0,58426 | 0 |
| | | | F136Bpa 2 S239Bpa 0 | 0,92668 | 0 |
| | | | F136Bpa 2 M243Bpa 2 | 0,21876 | 0 |
| | | | F136Bpa 2 M243Bpa 0 | 0,10307 | 0 |
| | | | F136Bpa 2 P245Bpa 2 | 0,84049 | 0 |
| | | | F136Bpa 2 P245Bpa 0 | 0,29904 | 0 |
| | | | F136Bpa 2 F250Bpa  2 | 0,47474 | 0 |
| | | | F136Bpa 2 F250Bpa 0 | 0,90611 | 0 |
| | | | F136Bpa 2 A254Bpa 2 | 8,69E-05 | 1 |
| | | | F136Bpa 2 A254Bpa 0 | 0,71372 | 0 |
| | | | F136Bpa 2 L194Bpa 2 | 1 | 0 |
| | | | F136Bpa 2 L194Bpa 0 | 1 | 0 |
| | | | F136Bpa 2 V191Bpa 2 | 0,11333 | 0 |
| | | | F136Bpa 2 V191Bpa 0 | 0,17602 | 0 |
| | | | F136Bpa 2 L188Bpa 2 | 0,01298 | 1 |
| | | | F136Bpa 2 L188Bpa 0 | 0,12416 | 0 |
| | | | F136Bpa 2 F187Azi 2 | 0,59629 | 0 |
| | | | F136Bpa 2 F187Azi 0 | 0,09448 | 0 |
| | | | F136Bpa 2 L185Bpa 2 | 0,29358 | 0 |
| | | | F136Bpa 2 L185Bpa 0 | 0,32436 | 0 |
| | | | F136Bpa 2 V181Bpa 2 | 0,45433 | 0 |
| | | | F136Bpa 2 V181Bpa 0 | 0,59535 | 0 |
| | | | F136Bpa 2 S179Bpa 2 | 0,44455 | 0 |
| | | | F136Bpa 2 S179Bpa 0 | 0,23847 | 0 |
| | | | F136Bpa 2 W176Bpa 2 | 0,00119 | 1 |
| | | | F136Bpa 2 W176Bpa 0 | 0,17394 | 0 |
| | | | F136Bpa 2 L174Bpa 2 | 0,99045 | 0 |
| | | | F136Bpa 2 L174Bpa 0 | 0,15974 | 0 |
| | | | F136Bpa 2 L130Bpa 2 | 0,99779 | 0 |
| | | | F136Bpa 2 L130Bpa 0 | 0,59787 | 0 |
| | | | F136Bpa 2 H134Bpa 2 | 0,72377 | 0 |
| | | | F136Bpa 2 H134Bpa 0 | 0,34369 | 0 |
| | | | F136Bpa 0 A235Bpa 2 | 0,95232 | 0 |
| | | | F136Bpa 0 A235Bpa 0 | 0,99317 | 0 |
| | | | F136Bpa 0 S239Bpa 2 | 0,91875 | 0 |
| | | | F136Bpa 0 S239Bpa 0 | 0,60016 | 0 |
| | | | F136Bpa 0 M243Bpa 2 | 1 | 0 |
| | | | F136Bpa 0 M243Bpa 0 | 0,99925 | 0 |
| | | | F136Bpa 0 P245Bpa 2 | 0,27882 | 0 |
| | | | F136Bpa 0 P245Bpa 0 | 1 | 0 |
| | | | F136Bpa 0 F250Bpa  2 | 0,25956 | 0 |
| | | | F136Bpa 0 F250Bpa 0 | 0,98528 | 0 |
| | | | F136Bpa 0 A254Bpa 2 | 7,96E-06 | 1 |
| | | | F136Bpa 0 A254Bpa 0 | 1 | 0 |
| | | | F136Bpa 0 L194Bpa 2 | 0,99755 | 0 |
| | | | F136Bpa 0 L194Bpa 0 | 0,98601 | 0 |
| | | | F136Bpa 0 V191Bpa 2 | 0,9997 | 0 |
| | | | F136Bpa 0 V191Bpa 0 | 1 | 0 |
| | | | F136Bpa 0 L188Bpa 2 | 0,00667 | 1 |
| | | | F136Bpa 0 L188Bpa 0 | 1 | 0 |
| | | | F136Bpa 0 F187Azi 2 | 0,53915 | 0 |
| | | | F136Bpa 0 F187Azi 0 | 0,9187 | 0 |
| | | | F136Bpa 0 L185Bpa 2 | 1 | 0 |
| | | | F136Bpa 0 L185Bpa 0 | 1 | 0 |
| | | | F136Bpa 0 V181Bpa 2 | 0,0545 | 0 |
| | | | F136Bpa 0 V181Bpa 0 | 0,99588 | 0 |
| | | | F136Bpa 0 S179Bpa 2 | 0,99911 | 0 |
| | | | F136Bpa 0 S179Bpa 0 | 1 | 0 |
| | | | F136Bpa 0 W176Bpa 2 | 1,61E-04 | 1 |
| | | | F136Bpa 0 W176Bpa 0 | 1 | 0 |
| | | | F136Bpa 0 L174Bpa 2 | 1 | 0 |
| | | | F136Bpa 0 L174Bpa 0 | 1 | 0 |
| | | | F136Bpa 0 L130Bpa 2 | 0,99707 | 0 |
| | | | F136Bpa 0 L130Bpa 0 | 0,90868 | 0 |
| | | | F136Bpa 0 H134Bpa 2 | 0,99771 | 0 |
| | | | F136Bpa 0 H134Bpa 0 | 0,97404 | 0 |
| | | | F136Bpa 0 F136Bpa 2 | 0,17258 | 0 |
| | | | A137Bpa 2 A235Bpa 2 | 1 | 0 |
| | | | A137Bpa 2 A235Bpa 0 | 0,82105 | 0 |
| | | | A137Bpa 2 S239Bpa 2 | 0,97529 | 0 |
| | | | A137Bpa 2 S239Bpa 0 | 0,99629 | 0 |
| | | | A137Bpa 2 M243Bpa 2 | 0,90605 | 0 |
| | | | A137Bpa 2 M243Bpa 0 | 0,82952 | 0 |
| | | | A137Bpa 2 P245Bpa 2 | 0,99309 | 0 |
| | | | A137Bpa 2 P245Bpa 0 | 0,92458 | 0 |
| | | | A137Bpa 2 F250Bpa  2 | 0,62595 | 0 |
| | | | A137Bpa 2 F250Bpa 0 | 0,99405 | 0 |
| | | | A137Bpa 2 A254Bpa 2 | 4,74E-04 | 1 |
| | | | A137Bpa 2 A254Bpa 0 | 0,97985 | 0 |
| | | | A137Bpa 2 L194Bpa 2 | 1 | 0 |
| | | | A137Bpa 2 L194Bpa 0 | 1 | 0 |
| | | | A137Bpa 2 V191Bpa 2 | 0,84191 | 0 |
| | | | A137Bpa 2 V191Bpa 0 | 0,88413 | 0 |
| | | | A137Bpa 2 L188Bpa 2 | 0,01832 | 1 |
| | | | A137Bpa 2 L188Bpa 0 | 0,84928 | 0 |
| | | | A137Bpa 2 F187Azi 2 | 0,97769 | 0 |
| | | | A137Bpa 2 F187Azi 0 | 0,82183 | 0 |
| | | | A137Bpa 2 L185Bpa 2 | 0,92597 | 0 |
| | | | A137Bpa 2 L185Bpa 0 | 0,93433 | 0 |
| | | | A137Bpa 2 V181Bpa 2 | 0,90615 | 0 |

| | | | | | |
|---|---|---|---|---|---|
| | | | A137Bpa 2 V181Bpa 0 | 0,97443 | 0 |
| | | | A137Bpa 2 S179Bpa 2 | 0,95773 | 0 |
| | | | A137Bpa 2 S179Bpa 0 | 0,90623 | 0 |
| | | | A137Bpa 2 W176Bpa 2 | 0,00452 | 1 |
| | | | A137Bpa 2 W176Bpa 0 | 0,88031 | 0 |
| | | | A137Bpa 2 L174Bpa 2 | 0,99746 | 0 |
| | | | A137Bpa 2 L174Bpa 0 | 0,87505 | 0 |
| | | | A137Bpa 2 L130Bpa 2 | 0,99935 | 0 |
| | | | A137Bpa 2 L130Bpa 0 | 0,9765 | 0 |
| | | | A137Bpa 2 H134Bpa 2 | 0,98288 | 0 |
| | | | A137Bpa 2 H134Bpa 0 | 0,94363 | 0 |
| | | | A137Bpa 2 F136Bpa 2 | 1 | 0 |
| | | | A137Bpa 2 F136Bpa 0 | 0,88499 | 0 |
| | | | A137Bpa 0 A235Bpa 2 | 0,91652 | 0 |
| | | | A137Bpa 0 A235Bpa 0 | 1 | 0 |
| | | | A137Bpa 0 S239Bpa 2 | 0,31571 | 0 |
| | | | A137Bpa 0 S239Bpa 0 | 0,21135 | 0 |
| | | | A137Bpa 0 M243Bpa 2 | 0,83527 | 0 |
| | | | A137Bpa 0 M243Bpa 0 | 1 | 0 |
| | | | A137Bpa 0 P245Bpa 2 | 0,04197 | 1 |
| | | | A137Bpa 0 P245Bpa 0 | 0,99993 | 0 |
| | | | A137Bpa 0 F250Bpa 2 | 0,24855 | 0 |
| | | | A137Bpa 0 F250Bpa 0 | 0,84563 | 0 |
| | | | A137Bpa 0 A254Bpa 2 | 6,84E-06 | 1 |
| | | | A137Bpa 0 A254Bpa 0 | 0,9966 | 0 |
| | | | A137Bpa 0 L194Bpa 2 | 0,98967 | 0 |
| | | | A137Bpa 0 L194Bpa 0 | 0,95084 | 0 |
| | | | A137Bpa 0 V191Bpa 2 | 1 | 0 |
| | | | A137Bpa 0 V191Bpa 0 | 0,99997 | 0 |
| | | | A137Bpa 0 L188Bpa 2 | 0,00639 | 1 |
| | | | A137Bpa 0 L188Bpa 0 | 1 | 0 |
| | | | A137Bpa 0 F187Azi 2 | 0,03857 | 1 |
| | | | A137Bpa 0 F187Azi 0 | 1 | 0 |
| | | | A137Bpa 0 L185Bpa 2 | 0,99656 | 0 |
| | | | A137Bpa 0 L185Bpa 0 | 0,98239 | 0 |
| | | | A137Bpa 0 V181Bpa 2 | 0,04662 | 1 |
| | | | A137Bpa 0 V181Bpa 0 | 0,76763 | 0 |
| | | | A137Bpa 0 S179Bpa 2 | 0,77532 | 0 |
| | | | A137Bpa 0 S179Bpa 0 | 1 | 0 |
| | | | A137Bpa 0 W176Bpa 2 | 1,41E-04 | 1 |
| | | | A137Bpa 0 W176Bpa 0 | 1 | 0 |
| | | | A137Bpa 0 L174Bpa 2 | 1 | 0 |
| | | | A137Bpa 0 L174Bpa 0 | 1 | 0 |
| | | | A137Bpa 0 L130Bpa 2 | 0,9695 | 0 |
| | | | A137Bpa 0 L130Bpa 0 | 0,31562 | 0 |
| | | | A137Bpa 0 H134Bpa 2 | 0,91217 | 0 |
| | | | A137Bpa 0 H134Bpa 0 | 0,12002 | 0 |
| | | | A137Bpa 0 F136Bpa 2 | 0,09959 | 0 |
| | | | A137Bpa 0 F136Bpa 0 | 0,93598 | 0 |
| | | | A137Bpa 0 A137Bpa 2 | 0,82813 | 0 |
| | | | L138Bpa 2 A235Bpa 2 | 0,97764 | 0 |
| | | | L138Bpa 2 A235Bpa 0 | 0,45532 | 0 |
| | | | L138Bpa 2 S239Bpa 2 | 1 | 0 |
| | | | L138Bpa 2 S239Bpa 0 | 0,96504 | 0 |
| | | | L138Bpa 2 M243Bpa 2 | 1 | 0 |
| | | | L138Bpa 2 M243Bpa 0 | 0,64809 | 0 |
| | | | L138Bpa 2 P245Bpa 2 | 0,89155 | 0 |
| | | | L138Bpa 2 P245Bpa 0 | 1 | 0 |
| | | | L138Bpa 2 F250Bpa 2 | 0,27204 | 0 |
| | | | L138Bpa 2 F250Bpa 0 | 0,99995 | 0 |
| | | | L138Bpa 2 A254Bpa 2 | 9,40E-06 | 1 |
| | | | L138Bpa 2 A254Bpa 0 | 1 | 0 |
| | | | L138Bpa 2 L194Bpa 2 | 0,99971 | 0 |
| | | | L138Bpa 2 L194Bpa 0 | 0,99814 | 0 |
| | | | L138Bpa 2 V191Bpa 2 | 0,55085 | 0 |
| | | | L138Bpa 2 V191Bpa 0 | 1 | 0 |
| | | | L138Bpa 2 L188Bpa 2 | 0,007 | 1 |
| | | | L138Bpa 2 L188Bpa 0 | 0,93821 | 0 |
| | | | L138Bpa 2 F187Azi 2 | 0,99956 | 0 |
| | | | L138Bpa 2 F187Azi 0 | 0,21251 | 0 |
| | | | L138Bpa 2 L185Bpa 2 | 1 | 0 |
| | | | L138Bpa 2 L185Bpa 0 | 1 | 0 |
| | | | L138Bpa 2 V181Bpa 2 | 0,06456 | 0 |
| | | | L138Bpa 2 V181Bpa 0 | 1 | 0 |
| | | | L138Bpa 2 S179Bpa 2 | 1 | 0 |
| | | | L138Bpa 2 S179Bpa 0 | 1 | 0 |
| | | | L138Bpa 2 W176Bpa 2 | 1,85E-04 | 1 |
| | | | L138Bpa 2 W176Bpa 0 | 1 | 0 |
| | | | L138Bpa 2 L174Bpa 2 | 1 | 0 |
| | | | L138Bpa 2 L174Bpa 0 | 0,99961 | 0 |
| | | | L138Bpa 2 L130Bpa 2 | 0,99996 | 0 |
| | | | L138Bpa 2 L130Bpa 0 | 1 | 0 |
| | | | L138Bpa 2 H134Bpa 2 | 1 | 0 |
| | | | L138Bpa 2 H134Bpa 0 | 1 | 0 |
| | | | L138Bpa 2 F136Bpa 2 | 0,29754 | 0 |
| | | | L138Bpa 2 F136Bpa 0 | 0,99973 | 0 |
| | | | L138Bpa 2 A137Bpa 2 | 0,93244 | 0 |
| | | | L138Bpa 2 A137Bpa 0 | 0,23279 | 0 |
| | | | L138Bpa 0 A235Bpa 2 | 0,96229 | 0 |
| | | | L138Bpa 0 A235Bpa 0 | 0,99999 | 0 |
| | | | L138Bpa 0 S239Bpa 2 | 0,99934 | 0 |
| | | | L138Bpa 0 S239Bpa 0 | 0,87722 | 0 |
| | | | L138Bpa 0 M243Bpa 2 | 1 | 0 |
| | | | L138Bpa 0 M243Bpa 0 | 1 | 0 |
| | | | L138Bpa 0 P245Bpa 2 | 0,74678 | 0 |
| | | | L138Bpa 0 P245Bpa 0 | 1 | 0 |
| | | | L138Bpa 0 F250Bpa 2 | 0,26275 | 0 |
| | | | L138Bpa 0 F250Bpa 0 | 0,99859 | 0 |
| | | | L138Bpa 0 A254Bpa 2 | 8,26E-06 | 1 |
| | | | L138Bpa 0 A254Bpa 0 | 1 | 0 |
| | | | L138Bpa 0 L194Bpa 2 | 0,99877 | 0 |
| | | | L138Bpa 0 L194Bpa 0 | 0,99309 | 0 |
| | | | L138Bpa 0 V191Bpa 2 | 1 | 0 |
| | | | L138Bpa 0 V191Bpa 0 | 1 | 0 |
| | | | L138Bpa 0 L188Bpa 2 | 0,00675 | 1 |
| | | | L138Bpa 0 L188Bpa 0 | 1 | 0 |
| | | | L138Bpa 0 F187Azi 2 | 0,98602 | 0 |
| | | | L138Bpa 0 F187Azi 0 | 0,99996 | 0 |
| | | | L138Bpa 0 L185Bpa 2 | 1 | 0 |
| | | | L138Bpa 0 L185Bpa 0 | 1 | 0 |
| | | | L138Bpa 0 V181Bpa 2 | 0,05725 | 0 |
| | | | L138Bpa 0 V181Bpa 0 | 0,99999 | 0 |
| | | | L138Bpa 0 S179Bpa 2 | 1 | 0 |
| | | | L138Bpa 0 S179Bpa 0 | 1 | 0 |
| | | | L138Bpa 0 W176Bpa 2 | 1,66E-04 | 1 |
| | | | L138Bpa 0 W176Bpa 0 | 1 | 0 |
| | | | L138Bpa 0 L174Bpa 2 | 1 | 0 |

| | | | | | | |
|---|---|---|---|---|---|---|
| | | | | L138Bpa 0 L174Bpa 0 | 1 | 0 |
| | | | | L138Bpa 0 L130Bpa 2 | 0,99937 | 0 |
| | | | | L138Bpa 0 L130Bpa 0 | 0,99903 | 0 |
| | | | | L138Bpa 0 H134Bpa 2 | 0,99996 | 0 |
| | | | | L138Bpa 0 H134Bpa 0 | 1 | 0 |
| | | | | L138Bpa 0 F136Bpa 2 | 0,22034 | 0 |
| | | | | L138Bpa 0 F136Bpa 0 | 1 | 0 |
| | | | | L138Bpa 0 A137Bpa 2 | 0,90187 | 0 |
| | | | | L138Bpa 0 A137Bpa 0 | 0,99999 | 0 |
| | | | | L138Bpa 0 L138Bpa 2 | 1 | 0 |
| | | | | O1 2 A235Bpa 2 | 0,99697 | 0 |
| | | | | O1 2 A235Bpa 0 | 0,99904 | 0 |
| | | | | O1 2 S239Bpa 2 | 1 | 0 |
| | | | | O1 2 S239Bpa 0 | 1 | 0 |
| | | | | O1 2 M243Bpa 2 | 1 | 0 |
| | | | | O1 2 M243Bpa 0 | 0,99953 | 0 |
| | | | | O1 2 P245Bpa 2 | 1 | 0 |
| | | | | O1 2 P245Bpa 0 | 1 | 0 |
| | | | | O1 2 F250Bpa 2 | 0,29167 | 0 |
| | | | | O1 2 F250Bpa 0 | 1 | 0 |
| | | | | O1 2 A254Bpa 2 | 1,19E-05 | 1 |
| | | | | O1 2 A254Bpa 0 | 1 | 0 |
| | | | | O1 2 L194Bpa 2 | 1 | 0 |
| | | | | O1 2 L194Bpa 0 | 1 | 0 |
| | | | | O1 2 V191Bpa 2 | 0,9998 | 0 |
| | | | | O1 2 V191Bpa 0 | 1 | 0 |
| | | | | O1 2 L188Bpa 2 | 0,00747 | 1 |
| | | | | O1 2 L188Bpa 0 | 0,99994 | 0 |
| | | | | O1 2 F187Azi 2 | 1 | 0 |
| | | | | O1 2 F187Azi 0 | 0,99888 | 0 |
| | | | | O1 2 L185Bpa 2 | 1 | 0 |
| | | | | O1 2 L185Bpa 0 | 1 | 0 |
| | | | | O1 2 V181Bpa 2 | 0,08576 | 0 |
| | | | | O1 2 V181Bpa 0 | 1 | 0 |
| | | | | O1 2 S179Bpa 2 | 1 | 0 |
| | | | | O1 2 S179Bpa 0 | 1 | 0 |
| | | | | O1 2 W176Bpa 2 | 2,26E-04 | 1 |
| | | | | O1 2 W176Bpa 0 | 1 | 0 |
| | | | | O1 2 L174Bpa 2 | 1 | 0 |
| | | | | O1 2 L174Bpa 0 | 1 | 0 |
| | | | | O1 2 L130Bpa 2 | 1 | 0 |
| | | | | O1 2 L130Bpa 0 | 1 | 0 |
| | | | | O1 2 H134Bpa 2 | 1 | 0 |
| | | | | O1 2 H134Bpa 0 | 1 | 0 |
| | | | | O1 2 F136Bpa 2 | 0,75313 | 0 |
| | | | | O1 2 F136Bpa 0 | 1 | 0 |
| | | | | O1 2 A137Bpa 2 | 0,98135 | 0 |
| | | | | O1 2 A137Bpa 0 | 0,99927 | 0 |
| | | | | O1 2 L138Bpa 2 | 1 | 0 |
| | | | | O1 2 L138Bpa 0 | 1 | 0 |
| | | | | O1 0 A235Bpa 2 | 0,89347 | 0 |
| | | | | O1 0 A235Bpa 0 | 1 | 0 |
| | | | | O1 0 S239Bpa 2 | 0,12952 | 0 |
| | | | | O1 0 S239Bpa 0 | 0,11107 | 0 |
| | | | | O1 0 M243Bpa 2 | 0,37176 | 0 |
| | | | | O1 0 M243Bpa 0 | 1 | 0 |
| | | | | O1 0 P245Bpa 2 | 0,01548 | 1 |
| | | | | O1 0 P245Bpa 0 | 0,99388 | 0 |
| | | | | O1 0 F250Bpa 2 | 0,24305 | 0 |
| | | | | O1 0 F250Bpa 0 | 0,70986 | 0 |
| | | | | O1 0 A254Bpa 2 | 6,32E-06 | 1 |
| | | | | O1 0 A254Bpa 0 | 0,97763 | 0 |
| | | | | O1 0 L194Bpa 2 | 0,98149 | 0 |
| | | | | O1 0 L194Bpa 0 | 0,92052 | 0 |
| | | | | O1 0 V191Bpa 2 | 0,99558 | 0 |
| | | | | O1 0 V191Bpa 0 | 0,96678 | 0 |
| | | | | O1 0 L188Bpa 2 | 0,00625 | 1 |
| | | | | O1 0 L188Bpa 0 | 0,99996 | 0 |
| | | | | O1 0 F187Azi 2 | 0,00832 | 1 |
| | | | | O1 0 F187Azi 0 | 1 | 0 |
| | | | | O1 0 L185Bpa 2 | 0,93212 | 0 |
| | | | | O1 0 L185Bpa 0 | 0,84774 | 0 |
| | | | | O1 0 V181Bpa 2 | 0,04303 | 1 |
| | | | | O1 0 V181Bpa 0 | 0,51822 | 0 |
| | | | | O1 0 S179Bpa 2 | 0,5008 | 0 |
| | | | | O1 0 S179Bpa 0 | 0,9981 | 0 |
| | | | | O1 0 W176Bpa 2 | 1,33E-04 | 1 |
| | | | | O1 0 W176Bpa 0 | 0,99972 | 0 |
| | | | | O1 0 L174Bpa 2 | 0,99997 | 0 |
| | | | | O1 0 L174Bpa 0 | 0,97752 | 0 |
| | | | | O1 0 L130Bpa 2 | 0,93202 | 0 |
| | | | | O1 0 L130Bpa 0 | 0,13353 | 0 |
| | | | | O1 0 H134Bpa 2 | 0,78909 | 0 |
| | | | | O1 0 H134Bpa 0 | 0,02366 | 1 |
| | | | | O1 0 F136Bpa 2 | 0,07456 | 0 |
| | | | | O1 0 F136Bpa 0 | 0,26719 | 0 |
| | | | | O1 0 A137Bpa 2 | 0,7948 | 0 |
| | | | | O1 0 A137Bpa 0 | 0,99995 | 0 |
| | | | | O1 0 L138Bpa 2 | 0,03576 | 1 |
| | | | | O1 0 L138Bpa 0 | 0,9931 | 0 |
| | | | | O1 0 O1 2 | 0,99389 | 0 |
| S1c - before and after UV | Welch-ANOVA | 0 | F(43, 372.45)=15.29 | O1 after UV O1 before UV | 1 | 0 |
| | | | | O1+STIM1 before TG O1 before UV | 1 | 0 |
| | | | | O1+STIM1 before TG O1 after UV | 1 | 0 |
| | | | | O1+STIM1 after TG O1 before UV | 0,17545 | 0 |
| | | | | O1+STIM1 after TG O1 after UV | 0,18043 | 0 |
| | | | | O1+STIM1 after TG O1+STIM1 before TG | 0,18724 | 0 |
| | | | | L130Bpa before UV O1 before UV | 1 | 0 |
| | | | | L130Bpa before UV O1 after UV | 1 | 0 |
| | | | | L130Bpa before UV O1+STIM1 before TG | 1 | 0 |
| | | | | L130Bpa before UV O1+STIM1 after TG | 0,1891 | 0 |
| | | | | L130Bpa after UV O1 before UV | 1 | 0 |
| | | | | L130Bpa after UV O1 after UV | 1 | 0 |
| | | | | L130Bpa after UV O1+STIM1 before TG | 1 | 0 |
| | | | | L130Bpa after UV O1+STIM1 after TG | 0,19241 | 0 |
| | | | | L130Bpa after UV L130Bpa before UV | 1 | 0 |
| | | | | H134Bpa before UV O1 before UV | 1 | 0 |
| | | | | H134Bpa before UV O1 after UV | 1 | 0 |
| | | | | H134Bpa before UV O1+STIM1 before TG | 0,99988 | 0 |
| | | | | H134Bpa before UV O1+STIM1 after TG | 0,1735 | 0 |
| | | | | H134Bpa before UV L130Bpa before UV | 1 | 0 |
| | | | | H134Bpa before UV L130Bpa after UV | 1 | 0 |
| | | | | H134Bpa after UV O1 before UV | 0,99604 | 0 |
| | | | | H134Bpa after UV O1 after UV | 0,99997 | 0 |

| | | | | | |
|---|---|---|---|---|---|
| | | | H134Bpa after UV O1+STIM1 before TG | 1 | 0 |
| | | | H134Bpa after UV O1+STIM1  after TG | 0,19711 | 0 |
| | | | H134Bpa after UV L130Bpa before UV | 1 | 0 |
| | | | H134Bpa after UV L130Bpa after UV | 1 | 0 |
| | | | H134Bpa after UV H134Bpa before UV | 0,85855 | 0 |
| | | | F136Bpa before UV O1 before UV | 0,77055 | 0 |
| | | | F136Bpa before UV O1 after UV | 0,89164 | 0 |
| | | | F136Bpa before UV O1+STIM1 before TG | 0,95544 | 0 |
| | | | F136Bpa before UV O1+STIM1  after TG | 0,23734 | 0 |
| | | | F136Bpa before UV L130Bpa before UV | 0,99833 | 0 |
| | | | F136Bpa before UV L130Bpa after UV | 0,9989 | 0 |
| | | | F136Bpa before UV H134Bpa before UV | 0,60141 | 0 |
| | | | F136Bpa before UV H134Bpa after UV | 0,99902 | 0 |
| | | | F136Bpa after UV O1 before UV | 0,07113 | 0 |
| | | | F136Bpa after UV O1 after UV | 0,11326 | 0 |
| | | | F136Bpa after UV O1+STIM1 before TG | 0,16385 | 0 |
| | | | F136Bpa after UV O1+STIM1  after TG | 0,29918 | 0 |
| | | | F136Bpa after UV L130Bpa before UV | 0,38246 | 0 |
| | | | F136Bpa after UV L130Bpa after UV | 0,38243 | 0 |
| | | | F136Bpa after UV H134Bpa before UV | 0,04306 | 1 |
| | | | F136Bpa after UV H134Bpa after UV | 0,36142 | 0 |
| | | | F136Bpa after UV F136Bpa before UV | 0,99998 | 0 |
| | | | A137Bpa before UV O1 before UV | 0,45353 | 0 |
| | | | A137Bpa before UV O1 after UV | 0,50111 | 0 |
| | | | A137Bpa before UV O1+STIM1 before TG | 0,56079 | 0 |
| | | | A137Bpa before UV O1+STIM1  after TG | 0,64831 | 0 |
| | | | A137Bpa before UV L130Bpa before UV | 0,59924 | 0 |
| | | | A137Bpa before UV L130Bpa after UV | 0,6226 | 0 |
| | | | A137Bpa before UV H134Bpa before UV | 0,42873 | 0 |
| | | | A137Bpa before UV H134Bpa after UV | 0,6536 | 0 |
| | | | A137Bpa before UV F136Bpa before UV | 0,9306 | 0 |
| | | | A137Bpa before UV F136Bpa after UV | 0,99904 | 0 |
| | | | A137Bpa after UV O1 before UV | 0 | 1 |
| | | | A137Bpa after UV O1 after UV | 0 | 1 |
| | | | A137Bpa after UV O1+STIM1 before TG | 0 | 1 |
| | | | A137Bpa after UV O1+STIM1  after TG | 0,99976 | 0 |
| | | | A137Bpa after UV L130Bpa before UV | 0 | 1 |
| | | | A137Bpa after UV L130Bpa after UV | 0 | 1 |
| | | | A137Bpa after UV H134Bpa before UV | 0 | 1 |
| | | | A137Bpa after UV H134Bpa after UV | 0 | 1 |
| | | | A137Bpa after UV F136Bpa before UV | 0 | 1 |
| | | | A137Bpa after UV F136Bpa after UV | 0 | 1 |
| | | | A137Bpa after UV A137Bpa before UV | 8,20E-06 | 1 |
| | | | L138Bpa before UV O1 before UV | 1 | 0 |
| | | | L138Bpa before UV O1 after UV | 1 | 0 |
| | | | L138Bpa before UV O1+STIM1 before TG | 0,61672 | 0 |
| | | | L138Bpa before UV O1+STIM1  after TG | 0,16887 | 0 |
| | | | L138Bpa before UV L130Bpa before UV | 0,99997 | 0 |
| | | | L138Bpa before UV L130Bpa after UV | 0,98797 | 0 |
| | | | L138Bpa before UV H134Bpa before UV | 1 | 0 |
| | | | L138Bpa before UV H134Bpa after UV | 0,06896 | 0 |
| | | | L138Bpa before UV F136Bpa before UV | 0,31184 | 0 |
| | | | L138Bpa before UV F136Bpa after UV | 0,01782 | 1 |
| | | | L138Bpa before UV A137Bpa before UV | 0,37852 | 0 |
| | | | L138Bpa before UV A137Bpa after UV | 0 | 1 |
| | | | L138Bpa after UV O1 before UV | 1 | 0 |
| | | | L138Bpa after UV O1 after UV | 1 | 0 |
| | | | L138Bpa after UV O1+STIM1 before TG | 1 | 0 |
| | | | L138Bpa after UV O1+STIM1  after TG | 0,18226 | 0 |
| | | | L138Bpa after UV L130Bpa before UV | 1 | 0 |
| | | | L138Bpa after UV L130Bpa after UV | 1 | 0 |
| | | | L138Bpa after UV H134Bpa before UV | 1 | 0 |
| | | | L138Bpa after UV H134Bpa after UV | 0,97807 | 0 |
| | | | L138Bpa after UV F136Bpa before UV | 0,81635 | 0 |
| | | | L138Bpa after UV F136Bpa after UV | 0,08646 | 0 |
| | | | L138Bpa after UV A137Bpa before UV | 0,5088 | 0 |
| | | | L138Bpa after UV A137Bpa after UV | 0 | 1 |
| | | | L138Bpa after UV L138Bpa before UV | 0,22521 | 0 |
| | | | L194Bpa before UV O1 before UV | 1 | 0 |
| | | | L194Bpa before UV O1 after UV | 1 | 0 |
| | | | L194Bpa before UV O1+STIM1 before TG | 1 | 0 |
| | | | L194Bpa before UV O1+STIM1  after TG | 0,20701 | 0 |
| | | | L194Bpa before UV L130Bpa before UV | 1 | 0 |
| | | | L194Bpa before UV L130Bpa after UV | 1 | 0 |
| | | | L194Bpa before UV H134Bpa before UV | 1 | 0 |
| | | | L194Bpa before UV H134Bpa after UV | 1 | 0 |
| | | | L194Bpa before UV F136Bpa before UV | 1 | 0 |
| | | | L194Bpa before UV F136Bpa after UV | 0,97496 | 0 |
| | | | L194Bpa before UV A137Bpa before UV | 0,79314 | 0 |
| | | | L194Bpa before UV A137Bpa after UV | 0 | 1 |
| | | | L194Bpa before UV L138Bpa before UV | 0,99978 | 0 |
| | | | L194Bpa before UV L138Bpa after UV | 1 | 0 |
| | | | L194Bpa after UV O1 before UV | 0,96577 | 0 |
| | | | L194Bpa after UV O1 after UV | 0,97928 | 0 |
| | | | L194Bpa after UV O1+STIM1 before TG | 0,98956 | 0 |
| | | | L194Bpa after UV O1+STIM1  after TG | 0,33082 | 0 |
| | | | L194Bpa after UV L130Bpa before UV | 0,99463 | 0 |
| | | | L194Bpa after UV L130Bpa after UV | 0,99607 | 0 |
| | | | L194Bpa after UV H134Bpa before UV | 0,95508 | 0 |
| | | | L194Bpa after UV H134Bpa after UV | 0,99745 | 0 |
| | | | L194Bpa after UV F136Bpa before UV | 1 | 0 |
| | | | L194Bpa after UV F136Bpa after UV | 1 | 0 |
| | | | L194Bpa after UV A137Bpa before UV | 0,99999 | 0 |
| | | | L194Bpa after UV A137Bpa after UV | 7,70E-08 | 1 |
| | | | L194Bpa after UV L138Bpa before UV | 0,92731 | 0 |
| | | | L194Bpa after UV L138Bpa after UV | 0,97988 | 0 |
| | | | L194Bpa after UV L194Bpa before UV | 0,99994 | 0 |
| | | | V191Bpa before UV O1 before UV | 0,99986 | 0 |
| | | | V191Bpa before UV O1 after UV | 0,96538 | 0 |
| | | | V191Bpa before UV O1+STIM1 before TG | 0,08603 | 0 |
| | | | V191Bpa before UV O1+STIM1  after TG | 0,16134 | 0 |
| | | | V191Bpa before UV L130Bpa before UV | 0,98337 | 0 |
| | | | V191Bpa before UV L130Bpa after UV | 0,71404 | 0 |
| | | | V191Bpa before UV H134Bpa before UV | 0,99417 | 0 |
| | | | V191Bpa before UV H134Bpa after UV | 0,00407 | 1 |
| | | | V191Bpa before UV F136Bpa before UV | 0,12024 | 0 |
| | | | V191Bpa before UV F136Bpa after UV | 0,00632 | 1 |
| | | | V191Bpa before UV A137Bpa before UV | 0,30925 | 0 |
| | | | V191Bpa before UV A137Bpa after UV | 0 | 1 |
| | | | V191Bpa before UV L138Bpa before UV | 0,5701 | 0 |
| | | | V191Bpa before UV L138Bpa after UV | 3,45E-05 | 1 |
| | | | V191Bpa before UV L194Bpa before UV | 0,99348 | 0 |
| | | | V191Bpa before UV L194Bpa after UV | 0,87279 | 0 |
| | | | V191Bpa after UV O1 before UV | 1 | 0 |
| | | | V191Bpa after UV O1 after UV | 1 | 0 |
| | | | V191Bpa after UV O1+STIM1 before TG | 0,97554 | 0 |
| | | | V191Bpa after UV O1+STIM1  after TG | 0,17441 | 0 |

| | | | | | |
|---|---|---|---|---|---|
| | | | V191Bpa after UV L130Bpa before UV | 1 | 0 |
| | | | V191Bpa after UV L130Bpa after UV | 0,99992 | 0 |
| | | | V191Bpa after UV H134Bpa before UV | 1 | 0 |
| | | | V191Bpa after UV H134Bpa after UV | 0,32982 | 0 |
| | | | V191Bpa after UV F136Bpa before UV | 0,51333 | 0 |
| | | | V191Bpa after UV F136Bpa after UV | 0,03509 | 1 |
| | | | V191Bpa after UV A137Bpa before UV | 0,43148 | 0 |
| | | | V191Bpa after UV A137Bpa after UV | 0 | 1 |
| | | | V191Bpa after UV L138Bpa before UV | 0,99923 | 0 |
| | | | V191Bpa after UV L138Bpa after UV | 0,98705 | 0 |
| | | | V191Bpa after UV L194Bpa before UV | 1 | 0 |
| | | | V191Bpa after UV L194Bpa after UV | 0,9549 | 0 |
| | | | V191Bpa after UV V191Bpa before UV | 0,00185 | 1 |
| | | | L188Bpa before UV O1 before UV | 0,00797 | 1 |
| | | | L188Bpa before UV O1 after UV | 0,00838 | 1 |
| | | | L188Bpa before UV O1+STIM1 before TG | 0,00898 | 1 |
| | | | L188Bpa before UV O1+STIM1 after TG | 1 | 0 |
| | | | L188Bpa before UV L130Bpa before UV | 0,0091 | 1 |
| | | | L188Bpa before UV L130Bpa after UV | 0,00941 | 1 |
| | | | L188Bpa before UV H134Bpa before UV | 0,00782 | 1 |
| | | | L188Bpa before UV H134Bpa after UV | 0,00986 | 1 |
| | | | L188Bpa before UV F136Bpa before UV | 0,01382 | 1 |
| | | | L188Bpa before UV F136Bpa after UV | 0,02154 | 1 |
| | | | L188Bpa before UV A137Bpa before UV | 0,12754 | 0 |
| | | | L188Bpa before UV A137Bpa after UV | 0,9999 | 0 |
| | | | L188Bpa before UV L138Bpa before UV | 0,00746 | 1 |
| | | | L188Bpa before UV L138Bpa after UV | 0,00856 | 1 |
| | | | L188Bpa before UV L194Bpa before UV | 0,01063 | 1 |
| | | | L188Bpa before UV L194Bpa after UV | 0,02591 | 1 |
| | | | L188Bpa before UV V191Bpa before UV | 0,00688 | 1 |
| | | | L188Bpa before UV V191Bpa after UV | 0,00791 | 1 |
| | | | L188Bpa after UV O1 before UV | 0,02496 | 1 |
| | | | L188Bpa after UV O1 after UV | 0,02667 | 1 |
| | | | L188Bpa after UV O1+STIM1 before TG | 0,02911 | 1 |
| | | | L188Bpa after UV O1+STIM1 after TG | 1 | 0 |
| | | | L188Bpa after UV L130Bpa before UV | 0,02984 | 1 |
| | | | L188Bpa after UV L130Bpa after UV | 0,03108 | 1 |
| | | | L188Bpa after UV H134Bpa before UV | 0,0243 | 1 |
| | | | L188Bpa after UV H134Bpa after UV | 0,03289 | 1 |
| | | | L188Bpa after UV F136Bpa before UV | 0,05166 | 0 |
| | | | L188Bpa after UV F136Bpa after UV | 0,09143 | 0 |
| | | | L188Bpa after UV A137Bpa before UV | 0,66955 | 0 |
| | | | L188Bpa after UV A137Bpa after UV | 0,33147 | 0 |
| | | | L188Bpa after UV L138Bpa before UV | 0,0228 | 1 |
| | | | L188Bpa after UV L138Bpa after UV | 0,0273 | 1 |
| | | | L188Bpa after UV L194Bpa before UV | 0,03731 | 1 |
| | | | L188Bpa after UV L194Bpa after UV | 0,12826 | 0 |
| | | | L188Bpa after UV V191Bpa before UV | 0,02048 | 1 |
| | | | L188Bpa after UV V191Bpa after UV | 0,0246 | 1 |
| | | | L188Bpa after UV L188Bpa before UV | 0,99995 | 0 |
| | | | F187Bpa before UV O1 before UV | 1 | 0 |
| | | | F187Bpa before UV O1 after UV | 1 | 0 |
| | | | F187Bpa before UV O1+STIM1 before TG | 0,99974 | 0 |
| | | | F187Bpa before UV O1+STIM1 after TG | 0,17626 | 0 |
| | | | F187Bpa before UV L130Bpa before UV | 1 | 0 |
| | | | F187Bpa before UV L130Bpa after UV | 1 | 0 |
| | | | F187Bpa before UV H134Bpa before UV | 1 | 0 |
| | | | F187Bpa before UV H134Bpa after UV | 0,68174 | 0 |
| | | | F187Bpa before UV F136Bpa before UV | 0,61502 | 0 |
| | | | F187Bpa before UV F136Bpa after UV | 0,04644 | 1 |
| | | | F187Bpa before UV A137Bpa before UV | 0,45077 | 0 |
| | | | F187Bpa before UV A137Bpa after UV | 0 | 1 |
| | | | F187Bpa before UV L138Bpa before UV | 0,99969 | 0 |
| | | | F187Bpa before UV L138Bpa after UV | 1 | 0 |
| | | | F187Bpa before UV L194Bpa before UV | 1 | 0 |
| | | | F187Bpa before UV L194Bpa after UV | 0,96287 | 0 |
| | | | F187Bpa before UV V191Bpa before UV | 0,09289 | 0 |
| | | | F187Bpa before UV V191Bpa after UV | 1 | 0 |
| | | | F187Bpa before UV L188Bpa before UV | 0,00805 | 1 |
| | | | F187Bpa before UV L188Bpa after UV | 0,02522 | 1 |
| | | | F187Bpa after UV O1 before UV | 0,00528 | 1 |
| | | | F187Bpa after UV O1 after UV | 0,00599 | 1 |
| | | | F187Bpa after UV O1+STIM1 before TG | 0,00703 | 1 |
| | | | F187Bpa after UV O1+STIM1 after TG | 0,99978 | 0 |
| | | | F187Bpa after UV L130Bpa before UV | 0,00755 | 1 |
| | | | F187Bpa after UV L130Bpa after UV | 0,00809 | 1 |
| | | | F187Bpa after UV H134Bpa before UV | 0,00498 | 1 |
| | | | F187Bpa after UV H134Bpa after UV | 0,00888 | 1 |
| | | | F187Bpa after UV F136Bpa before UV | 0,02155 | 1 |
| | | | F187Bpa after UV F136Bpa after UV | 0,06131 | 0 |
| | | | F187Bpa after UV A137Bpa before UV | 0,9235 | 0 |
| | | | F187Bpa after UV A137Bpa after UV | 0,03621 | 1 |
| | | | F187Bpa after UV L138Bpa before UV | 0,00437 | 1 |
| | | | F187Bpa after UV L138Bpa after UV | 0,00619 | 1 |
| | | | F187Bpa after UV L194Bpa before UV | 0,01227 | 1 |
| | | | F187Bpa after UV L194Bpa after UV | 0,13138 | 0 |
| | | | F187Bpa after UV V191Bpa before UV | 0,00355 | 1 |
| | | | F187Bpa after UV V191Bpa after UV | 0,00506 | 1 |
| | | | F187Bpa after UV L188Bpa before UV | 0,97422 | 0 |
| | | | F187Bpa after UV L188Bpa after UV | 1 | 0 |
| | | | F187Bpa after UV F187Bpa before UV | 0,00532 | 1 |
| | | | L185Bpa before UV O1 before UV | 1 | 0 |
| | | | L185Bpa before UV O1 after UV | 1 | 0 |
| | | | L185Bpa before UV O1+STIM1 before TG | 0,74829 | 0 |
| | | | L185Bpa before UV O1+STIM1 after TG | -- | 1 |
| | | | L185Bpa before UV L130Bpa before UV | 0,99996 | 0 |
| | | | L185Bpa before UV L130Bpa after UV | 0,98885 | 0 |
| | | | L185Bpa before UV H134Bpa before UV | 1 | 0 |
| | | | L185Bpa before UV H134Bpa after UV | 0,11488 | 0 |
| | | | L185Bpa before UV F136Bpa before UV | 0,30794 | 0 |
| | | | L185Bpa before UV F136Bpa after UV | 0,01701 | 1 |
| | | | L185Bpa before UV A137Bpa before UV | 0,37019 | 0 |
| | | | L185Bpa before UV A137Bpa after UV | 0 | 1 |
| | | | L185Bpa before UV L138Bpa before UV | 1 | 0 |
| | | | L185Bpa before UV L138Bpa after UV | 0,68894 | 0 |
| | | | L185Bpa before UV L194Bpa before UV | 0,99971 | 0 |
| | | | L185Bpa before UV L194Bpa after UV | 0,92259 | 0 |
| | | | L185Bpa before UV V191Bpa before UV | 0,99982 | 0 |
| | | | L185Bpa before UV V191Bpa after UV | 0,99998 | 0 |
| | | | L185Bpa before UV L188Bpa before UV | 0,00737 | 1 |
| | | | L185Bpa before UV L188Bpa after UV | 0,02246 | 1 |
| | | | L185Bpa before UV F187Bpa before UV | 0,99995 | 0 |
| | | | L185Bpa before UV F187Bpa after UV | 0,00426 | 1 |
| | | | L185Bpa after UV O1 before UV | 1 | 0 |
| | | | L185Bpa after UV O1 after UV | 1 | 0 |
| | | | L185Bpa after UV O1+STIM1 before TG | 1 | 0 |
| | | | L185Bpa after UV O1+STIM1 after TG | 0,18915 | 0 |

| | | | | | |
|---|---|---|---|---|---|
| | | | L185Bpa after UV L130Bpa before UV | 1 | 0 |
| | | | L185Bpa after UV L130Bpa after UV | 1 | 0 |
| | | | L185Bpa after UV H134Bpa before UV | 0,99983 | 0 |
| | | | L185Bpa after UV H134Bpa after UV | 1 | 0 |
| | | | L185Bpa after UV F136Bpa before UV | 0,9804 | 0 |
| | | | L185Bpa after UV F136Bpa after UV | 0,20906 | 0 |
| | | | L185Bpa after UV A137Bpa before UV | 0,58111 | 0 |
| | | | L185Bpa after UV A137Bpa after UV | 0 | 1 |
| | | | L185Bpa after UV L138Bpa before UV | 0,72044 | 0 |
| | | | L185Bpa after UV L138Bpa after UV | 1 | 0 |
| | | | L185Bpa after UV L194Bpa before UV | 1 | 0 |
| | | | L185Bpa after UV L194Bpa after UV | 0,99217 | 0 |
| | | | L185Bpa after UV V191Bpa before UV | 0,10806 | 0 |
| | | | L185Bpa after UV V191Bpa after UV | 0,98864 | 0 |
| | | | L185Bpa after UV L188Bpa before UV | 0,00914 | 1 |
| | | | L185Bpa after UV L188Bpa after UV | 0,02982 | 1 |
| | | | L185Bpa after UV F187Bpa before UV | 0,99981 | 0 |
| | | | L185Bpa after UV F187Bpa after UV | 0,00738 | 1 |
| | | | L185Bpa after UV L185Bpa before UV | 0,80598 | 0 |
| | | | V181Bpa before UV O1 before UV | 0,09113 | 0 |
| | | | V181Bpa before UV O1 after UV | 0,10717 | 0 |
| | | | V181Bpa before UV O1+STIM1 before TG | 0,12962 | 0 |
| | | | V181Bpa before UV O1+STIM1  after TG | 0,68418 | 0 |
| | | | V181Bpa before UV L130Bpa before UV | 0,14849 | 0 |
| | | | V181Bpa before UV L130Bpa after UV | 0,15879 | 0 |
| | | | V181Bpa before UV H134Bpa before UV | 0,08319 | 0 |
| | | | V181Bpa before UV H134Bpa after UV | 0,17318 | 0 |
| | | | V181Bpa before UV F136Bpa before UV | 0,47202 | 0 |
| | | | V181Bpa before UV F136Bpa after UV | 0,89219 | 0 |
| | | | V181Bpa before UV A137Bpa before UV | 1 | 0 |
| | | | V181Bpa before UV A137Bpa after UV | 1,04E-05 | 1 |
| | | | V181Bpa before UV L138Bpa before UV | 0,06873 | 0 |
| | | | V181Bpa before UV L138Bpa after UV | 0,10931 | 0 |
| | | | V181Bpa before UV L194Bpa before UV | 0,28733 | 0 |
| | | | V181Bpa before UV L194Bpa after UV | 0,99424 | 0 |
| | | | V181Bpa before UV V191Bpa before UV | 0,0515 | 0 |
| | | | V181Bpa before UV V191Bpa after UV | 0,08378 | 0 |
| | | | V181Bpa before UV L188Bpa before UV | 0,14695 | 0 |
| | | | V181Bpa before UV L188Bpa after UV | 0,7137 | 0 |
| | | | V181Bpa before UV F187Bpa before UV | 0,08977 | 0 |
| | | | V181Bpa before UV F187Bpa after UV | 0,94685 | 0 |
| | | | V181Bpa before UV L185Bpa before UV | 0,06656 | 0 |
| | | | V181Bpa before UV L185Bpa after UV | 0,1384 | 0 |
| | | | V181Bpa after UV O1 before UV | 2,82E-06 | 1 |
| | | | V181Bpa after UV O1 after UV | 3,17E-06 | 1 |
| | | | V181Bpa after UV O1+STIM1 before TG | 3,74E-06 | 1 |
| | | | V181Bpa after UV O1+STIM1  after TG | 1 | 0 |
| | | | V181Bpa after UV L130Bpa before UV | 3,79E-06 | 1 |
| | | | V181Bpa after UV L130Bpa after UV | 4,12E-06 | 1 |
| | | | V181Bpa after UV H134Bpa before UV | 2,71E-06 | 1 |
| | | | V181Bpa after UV H134Bpa after UV | 4,62E-06 | 1 |
| | | | V181Bpa after UV F136Bpa before UV | 9,70E-06 | 1 |
| | | | V181Bpa after UV F136Bpa after UV | 2,64E-05 | 1 |
| | | | V181Bpa after UV A137Bpa before UV | 0,00315 | 1 |
| | | | V181Bpa after UV A137Bpa after UV | 0,98475 | 0 |
| | | | V181Bpa after UV L138Bpa before UV | 2,45E-06 | 1 |
| | | | V181Bpa after UV L138Bpa after UV | 3,36E-06 | 1 |
| | | | V181Bpa after UV L194Bpa before UV | 5,32E-06 | 1 |
| | | | V181Bpa after UV L194Bpa after UV | 5,01E-05 | 1 |
| | | | V181Bpa after UV V191Bpa before UV | 2,03E-06 | 1 |
| | | | V181Bpa after UV V191Bpa after UV | 2,79E-06 | 1 |
| | | | V181Bpa after UV L188Bpa before UV | 1 | 0 |
| | | | V181Bpa after UV L188Bpa after UV | 0,99997 | 0 |
| | | | V181Bpa after UV F187Bpa before UV | 2,91E-06 | 1 |
| | | | V181Bpa after UV F187Bpa after UV | 0,93292 | 0 |
| | | | V181Bpa after UV L185Bpa before UV | 2,38E-06 | 1 |
| | | | V181Bpa after UV L185Bpa after UV | 3,89E-06 | 1 |
| | | | V181Bpa after UV V181Bpa before UV | 0,00392 | 1 |
| | | | S179Bpa before UV O1 before UV | 1 | 0 |
| | | | S179Bpa before UV O1 after UV | 1 | 0 |
| | | | S179Bpa before UV O1+STIM1 before TG | 0,9796 | 0 |
| | | | S179Bpa before UV O1+STIM1  after TG | 0,17193 | 0 |
| | | | S179Bpa before UV L130Bpa before UV | 1 | 0 |
| | | | S179Bpa before UV L130Bpa after UV | 0,99968 | 0 |
| | | | S179Bpa before UV H134Bpa before UV | 1 | 0 |
| | | | S179Bpa before UV H134Bpa after UV | 0,39436 | 0 |
| | | | S179Bpa before UV F136Bpa before UV | 0,46241 | 0 |
| | | | S179Bpa before UV F136Bpa after UV | 0,02913 | 1 |
| | | | S179Bpa before UV A137Bpa before UV | 0,40979 | 0 |
| | | | S179Bpa before UV A137Bpa after UV | 0 | 1 |
| | | | S179Bpa before UV L138Bpa before UV | 1 | 0 |
| | | | S179Bpa before UV L138Bpa after UV | 0,99462 | 0 |
| | | | S179Bpa before UV L194Bpa before UV | 0,99999 | 0 |
| | | | S179Bpa before UV L194Bpa after UV | 0,9454 | 0 |
| | | | S179Bpa before UV V191Bpa before UV | 0,90044 | 0 |
| | | | S179Bpa before UV V191Bpa after UV | 1 | 0 |
| | | | S179Bpa before UV L188Bpa before UV | 0,0077 | 1 |
| | | | S179Bpa before UV L188Bpa after UV | 0,02379 | 1 |
| | | | S179Bpa before UV F187Bpa before UV | 1 | 0 |
| | | | S179Bpa before UV F187Bpa after UV | 0,00476 | 1 |
| | | | S179Bpa before UV L185Bpa before UV | 1 | 0 |
| | | | S179Bpa before UV L185Bpa after UV | 0,98558 | 0 |
| | | | S179Bpa before UV V181Bpa before UV | 0,07748 | 0 |
| | | | S179Bpa before UV V181Bpa after UV | 2,62E-06 | 1 |
| | | | S179Bpa after UV O1 before UV | 1 | 0 |
| | | | S179Bpa after UV O1 after UV | 1 | 0 |
| | | | S179Bpa after UV O1+STIM1 before TG | 1 | 0 |
| | | | S179Bpa after UV O1+STIM1  after TG | 0,18229 | 0 |
| | | | S179Bpa after UV L130Bpa before UV | 1 | 0 |
| | | | S179Bpa after UV L130Bpa after UV | 1 | 0 |
| | | | S179Bpa after UV H134Bpa before UV | 1 | 0 |
| | | | S179Bpa after UV H134Bpa after UV | 0,99475 | 0 |
| | | | S179Bpa after UV F136Bpa before UV | 0,83677 | 0 |
| | | | S179Bpa after UV F136Bpa after UV | 0,09211 | 0 |
| | | | S179Bpa after UV A137Bpa before UV | 0,51041 | 0 |
| | | | S179Bpa after UV A137Bpa after UV | 0 | 1 |
| | | | S179Bpa after UV L138Bpa before UV | 0,71872 | 0 |
| | | | S179Bpa after UV L138Bpa after UV | 1 | 0 |
| | | | S179Bpa after UV L194Bpa before UV | 1 | 0 |
| | | | S179Bpa after UV L194Bpa after UV | 0,9804 | 0 |
| | | | S179Bpa after UV V191Bpa before UV | 0,02167 | 1 |
| | | | S179Bpa after UV V191Bpa after UV | 0,99961 | 0 |
| | | | S179Bpa after UV L188Bpa before UV | 0,00856 | 1 |
| | | | S179Bpa after UV L188Bpa after UV | 0,02731 | 1 |
| | | | S179Bpa after UV F187Bpa before UV | 1 | 0 |
| | | | S179Bpa after UV F187Bpa after UV | 0,00621 | 1 |
| | | | S179Bpa after UV L185Bpa before UV | 0,89969 | 0 |

| | | |
|---|---|---|
| S179Bpa after UV L185Bpa after UV | 1 | 0 |
| S179Bpa after UV V181Bpa before UV | 0,10997 | 0 |
| S179Bpa after UV V181Bpa after UV | 3,36E-06 | 1 |
| S179Bpa after UV S179Bpa before UV | 0,99952 | 0 |
| W176Bpa before UV O1 before UV | 2,46E-04 | 1 |
| W176Bpa before UV O1 after UV | 2,85E-04 | 1 |
| W176Bpa before UV O1+STIM1 before TG | 3,45E-04 | 1 |
| W176Bpa before UV O1+STIM1 after TG | 0,99989 | 0 |
| W176Bpa before UV L130Bpa before UV | 3,68E-04 | 1 |
| W176Bpa before UV L130Bpa after UV | 4,02E-04 | 1 |
| W176Bpa before UV H134Bpa before UV | 2,31E-04 | 1 |
| W176Bpa before UV H134Bpa after UV | 4,53E-04 | 1 |
| W176Bpa before UV F136Bpa before UV | 0,00128 | 1 |
| W176Bpa before UV F136Bpa after UV | 0,00483 | 1 |
| W176Bpa before UV A137Bpa before UV | 0,62375 | 0 |
| W176Bpa before UV A137Bpa after UV | 0,03314 | 1 |
| W176Bpa before UV L138Bpa before UV | 2,02E-04 | 1 |
| W176Bpa before UV L138Bpa after UV | 2,99E-04 | 1 |
| W176Bpa before UV L194Bpa before UV | 6,41E-04 | 1 |
| W176Bpa before UV L194Bpa after UV | 0,01624 | 1 |
| W176Bpa before UV V191Bpa before UV | 1,58E-04 | 1 |
| W176Bpa before UV V191Bpa after UV | 2,37E-04 | 1 |
| W176Bpa before UV L188Bpa before UV | 0,97879 | 0 |
| W176Bpa before UV L188Bpa after UV | 1 | 0 |
| W176Bpa before UV F187Bpa before UV | 2,51E-04 | 1 |
| W176Bpa before UV F187Bpa after UV | 1 | 0 |
| W176Bpa before UV L185Bpa before UV | 1,94E-04 | 1 |
| W176Bpa before UV L185Bpa after UV | 3,65E-04 | 1 |
| W176Bpa before UV V181Bpa before UV | 0,66511 | 0 |
| W176Bpa before UV V181Bpa after UV | 0,93546 | 0 |
| W176Bpa before UV S179Bpa before UV | 2,20E-04 | 1 |
| W176Bpa before UV S179Bpa after UV | 2,99E-04 | 1 |
| W176Bpa after UV O1 before UV | 0,0068 | 1 |
| W176Bpa after UV O1 after UV | 0,00802 | 1 |
| W176Bpa after UV O1+STIM1 before TG | 0,00984 | 1 |
| W176Bpa after UV O1+STIM1 after TG | 0,93838 | 0 |
| W176Bpa after UV L130Bpa before UV | 0,011 | 1 |
| W176Bpa after UV L130Bpa after UV | 0,01194 | 1 |
| W176Bpa after UV H134Bpa before UV | 0,00627 | 1 |
| W176Bpa after UV H134Bpa after UV | 0,01334 | 1 |
| W176Bpa after UV F136Bpa before UV | 0,04358 | 1 |
| W176Bpa after UV F136Bpa after UV | 0,16122 | 0 |
| W176Bpa after UV A137Bpa before UV | 0,99999 | 0 |
| W176Bpa after UV A137Bpa after UV | 4,48E-04 | 1 |
| W176Bpa after UV L138Bpa before UV | 0,00526 | 1 |
| W176Bpa after UV L138Bpa after UV | -- | 1 |
| W176Bpa after UV L194Bpa before UV | 0,02199 | 1 |
| W176Bpa after UV L194Bpa after UV | 0,42455 | 0 |
| W176Bpa after UV V191Bpa before UV | 0,004 | 1 |
| W176Bpa after UV V191Bpa after UV | 0,00638 | 1 |
| W176Bpa after UV L188Bpa before UV | 0,49551 | 0 |
| W176Bpa after UV L188Bpa after UV | 0,99773 | 0 |
| W176Bpa after UV F187Bpa before UV | 0,00682 | 1 |
| W176Bpa after UV F187Bpa after UV | 1 | 0 |
| W176Bpa after UV L185Bpa before UV | 0,00509 | 1 |
| W176Bpa after UV L185Bpa after UV | 0,0105 | 1 |
| W176Bpa after UV V181Bpa before UV | 1 | 0 |
| W176Bpa after UV V181Bpa after UV | 0,11185 | 0 |
| W176Bpa after UV S179Bpa before UV | 0,00589 | 1 |
| W176Bpa after UV S179Bpa after UV | 0,00834 | 1 |
| W176Bpa after UV W176Bpa before UV | 0,99996 | 0 |
| L174Bpa before UV O1 before UV | 1 | 0 |
| L174Bpa before UV O1 after UV | 1 | 0 |
| L174Bpa before UV O1+STIM1 before TG | 1 | 0 |
| L174Bpa before UV O1+STIM1 after TG | 0,18122 | 0 |
| L174Bpa before UV L130Bpa before UV | 1 | 0 |
| L174Bpa before UV L130Bpa after UV | 1 | 0 |
| L174Bpa before UV H134Bpa before UV | 1 | 0 |
| L174Bpa before UV H134Bpa after UV | 1 | 0 |
| L174Bpa before UV F136Bpa before UV | 0,99224 | 0 |
| L174Bpa before UV F136Bpa after UV | 0,36068 | 0 |
| L174Bpa before UV A137Bpa before UV | 0,54014 | 0 |
| L174Bpa before UV A137Bpa after UV | 0 | 1 |
| L174Bpa before UV L138Bpa before UV | 1 | 0 |
| L174Bpa before UV L138Bpa after UV | 1 | 0 |
| L174Bpa before UV L194Bpa before UV | 1 | 0 |
| L174Bpa before UV L194Bpa after UV | 0,98887 | 0 |
| L174Bpa before UV V191Bpa before UV | 1 | 0 |
| L174Bpa before UV V191Bpa after UV | 1 | 0 |
| L174Bpa before UV L188Bpa before UV | 0,00839 | 1 |
| L174Bpa before UV L188Bpa after UV | 0,02699 | 1 |
| L174Bpa before UV F187Bpa before UV | 1 | 0 |
| L174Bpa before UV F187Bpa after UV | 0,00638 | 1 |
| L174Bpa before UV L185Bpa before UV | 1 | 0 |
| L174Bpa before UV L185Bpa after UV | 1 | 0 |
| L174Bpa before UV V181Bpa before UV | 0,12476 | 0 |
| L174Bpa before UV V181Bpa after UV | 3,11E-06 | 1 |
| L174Bpa before UV S179Bpa before UV | 1 | 0 |
| L174Bpa before UV S179Bpa after UV | 1 | 0 |
| L174Bpa before UV W176Bpa before UV | 2,98E-04 | 1 |
| L174Bpa before UV W176Bpa after UV | 0,00896 | 1 |
| L174Bpa after UV O1 before UV | 0,18134 | 0 |
| L174Bpa after UV O1 after UV | 0,18789 | 0 |
| L174Bpa after UV O1+STIM1 before TG | 0,19684 | 0 |
| L174Bpa after UV O1+STIM1 after TG | 1 | 0 |
| L174Bpa after UV L130Bpa before UV | 0,19957 | 0 |
| L174Bpa after UV L130Bpa after UV | 0,20389 | 0 |
| L174Bpa after UV H134Bpa before UV | -- | 1 |
| L174Bpa after UV H134Bpa after UV | 0,21003 | 0 |
| L174Bpa after UV F136Bpa before UV | 0,26537 | 0 |
| L174Bpa after UV F136Bpa after UV | 0,35186 | 0 |
| L174Bpa after UV A137Bpa before UV | 0,80519 | 0 |
| L174Bpa after UV A137Bpa after UV | 0,97295 | 0 |
| L174Bpa after UV L138Bpa before UV | 0,17258 | 0 |
| L174Bpa after UV L138Bpa after UV | 0,1902 | 0 |
| L174Bpa after UV L194Bpa before UV | 0,22435 | 0 |
| L174Bpa after UV L194Bpa after UV | 0,40117 | 0 |
| L174Bpa after UV V191Bpa before UV | 0,1628 | 0 |
| L174Bpa after UV V191Bpa after UV | 0,17984 | 0 |
| L174Bpa after UV L188Bpa before UV | 1 | 0 |
| L174Bpa after UV L188Bpa after UV | 1 | 0 |
| L174Bpa after UV F187Bpa before UV | 0,18228 | 0 |
| L174Bpa after UV F187Bpa after UV | 1 | 0 |
| L174Bpa after UV L185Bpa before UV | 0,1712 | 0 |
| L174Bpa after UV L185Bpa after UV | 0,1994 | 0 |
| L174Bpa after UV V181Bpa before UV | 0,83817 | 0 |
| L174Bpa after UV V181Bpa after UV | 1 | 0 |

| | | |
|---|---|---|
| L174Bpa after UV S179Bpa before UV | 0,17661 | 0 |
| L174Bpa after UV S179Bpa after UV | 0,19025 | 0 |
| L174Bpa after UV W176Bpa before UV | 1 | 0 |
| L174Bpa after UV W176Bpa after UV | 0,99329 | 0 |
| L174Bpa after UV L174Bpa before UV | 0,18931 | 0 |
| A235Bpa before UV O1 before UV | 0,99758 | 0 |
| A235Bpa before UV O1 after UV | 0,99939 | 0 |
| A235Bpa before UV O1+STIM1 before TG | 0,99991 | 0 |
| A235Bpa before UV O1+STIM1  after TG | 0,25095 | 0 |
| A235Bpa before UV L130Bpa before UV | 1 | 0 |
| A235Bpa before UV L130Bpa after UV | 1 | 0 |
| A235Bpa before UV H134Bpa before UV | 0,9946 | 0 |
| A235Bpa before UV H134Bpa after UV | 1 | 0 |
| A235Bpa before UV F136Bpa before UV | 1 | 0 |
| A235Bpa before UV F136Bpa after UV | 1 | 0 |
| A235Bpa before UV A137Bpa before UV | 0,98 | 0 |
| A235Bpa before UV A137Bpa after UV | 1,43E-08 | 1 |
| A235Bpa before UV L138Bpa before UV | 0,9807 | 0 |
| A235Bpa before UV L138Bpa after UV | 0,99934 | 0 |
| A235Bpa before UV L194Bpa before UV | 1 | 0 |
| A235Bpa before UV L194Bpa after UV | 1 | 0 |
| A235Bpa before UV V191Bpa before UV | 0,93302 | 0 |
| A235Bpa before UV V191Bpa after UV | 0,99399 | 0 |
| A235Bpa before UV L188Bpa before UV | 0,01511 | 1 |
| A235Bpa before UV L188Bpa after UV | 0,06061 | 0 |
| A235Bpa before UV F187Bpa before UV | 0,99646 | 0 |
| A235Bpa before UV F187Bpa after UV | 0,03218 | 1 |
| A235Bpa before UV L185Bpa before UV | 0,97822 | 0 |
| A235Bpa before UV L185Bpa after UV | 0,99997 | 0 |
| A235Bpa before UV V181Bpa before UV | 0,69274 | 0 |
| A235Bpa before UV V181Bpa after UV | 1,18E-05 | 1 |
| A235Bpa before UV S179Bpa before UV | 0,99063 | 0 |
| A235Bpa before UV S179Bpa after UV | 0,9994 | 0 |
| A235Bpa before UV W176Bpa before UV | 0,00212 | 1 |
| A235Bpa before UV W176Bpa after UV | 0,08116 | 0 |
| A235Bpa before UV L174Bpa before UV | 0,99993 | 0 |
| A235Bpa before UV L174Bpa after UV | 0,28591 | 0 |
| A235Bpa after UV O1 before UV | 0,25429 | 0 |
| A235Bpa after UV O1 after UV | 0,3162 | 0 |
| A235Bpa after UV O1+STIM1 before TG | 0,3909 | 0 |
| A235Bpa after UV O1+STIM1  after TG | 0,34881 | 0 |
| A235Bpa after UV L130Bpa before UV | 0,51217 | 0 |
| A235Bpa after UV L130Bpa after UV | 0,53236 | 0 |
| A235Bpa after UV H134Bpa before UV | 0,21479 | 0 |
| A235Bpa after UV H134Bpa after UV | 0,55279 | 0 |
| A235Bpa after UV F136Bpa before UV | 0,99642 | 0 |
| A235Bpa after UV F136Bpa after UV | 1 | 0 |
| A235Bpa after UV A137Bpa before UV | 1 | 0 |
| A235Bpa after UV A137Bpa after UV | 1,01E-07 | 1 |
| A235Bpa after UV L138Bpa before UV | 0,15535 | 0 |
| A235Bpa after UV L138Bpa after UV | 0,30524 | 0 |
| A235Bpa after UV L194Bpa before UV | 0,92181 | 0 |
| A235Bpa after UV L194Bpa after UV | 1 | 0 |
| A235Bpa after UV V191Bpa before UV | 0,09929 | 0 |
| A235Bpa after UV V191Bpa after UV | 0,20842 | 0 |
| A235Bpa after UV L188Bpa before UV | 0,02914 | 1 |
| A235Bpa after UV L188Bpa after UV | 0,13691 | 0 |
| A235Bpa after UV F187Bpa before UV | 0,23246 | 0 |
| A235Bpa after UV F187Bpa after UV | 0,12884 | 0 |
| A235Bpa after UV L185Bpa before UV | 0,14974 | 0 |
| A235Bpa after UV L185Bpa after UV | 0,42922 | 0 |
| A235Bpa after UV V181Bpa before UV | 0,99476 | 0 |
| A235Bpa after UV V181Bpa after UV | 5,40E-05 | 1 |
| A235Bpa after UV S179Bpa before UV | 0,18849 | 0 |
| A235Bpa after UV S179Bpa after UV | 0,31032 | 0 |
| A235Bpa after UV W176Bpa before UV | 0,01367 | 1 |
| A235Bpa after UV W176Bpa after UV | 0,38382 | 0 |
| A235Bpa after UV L174Bpa before UV | 0,45366 | 0 |
| A235Bpa after UV L174Bpa after UV | 0,42285 | 0 |
| A235Bpa after UV A235Bpa before UV | 1 | 0 |
| S239Bpa before UV O1 before UV | 1 | 0 |
| S239Bpa before UV O1 after UV | 1 | 0 |
| S239Bpa before UV O1+STIM1 before TG | 0,99978 | 0 |
| S239Bpa before UV O1+STIM1  after TG | 0,17545 | 0 |
| S239Bpa before UV L130Bpa before UV | 1 | 0 |
| S239Bpa before UV L130Bpa after UV | 1 | 0 |
| S239Bpa before UV H134Bpa before UV | 1 | 0 |
| S239Bpa before UV H134Bpa after UV | 0,72322 | 0 |
| S239Bpa before UV F136Bpa before UV | 0,60314 | 0 |
| S239Bpa before UV F136Bpa after UV | 0,04439 | 1 |
| S239Bpa before UV A137Bpa before UV | 0,4439 | 0 |
| S239Bpa before UV A137Bpa after UV | 0 | 1 |
| S239Bpa before UV L138Bpa before UV | 1 | 0 |
| S239Bpa before UV L138Bpa after UV | 1 | 0 |
| S239Bpa before UV L194Bpa before UV | 1 | 0 |
| S239Bpa before UV L194Bpa after UV | 0,96047 | 0 |
| S239Bpa before UV V191Bpa before UV | 0,48526 | 0 |
| S239Bpa before UV V191Bpa after UV | 1 | 0 |
| S239Bpa before UV L188Bpa before UV | 0,00799 | 1 |
| S239Bpa before UV L188Bpa after UV | 0,02495 | 1 |
| S239Bpa before UV F187Bpa before UV | 1 | 0 |
| S239Bpa before UV F187Bpa after UV | 0,00522 | 1 |
| S239Bpa before UV L185Bpa before UV | 1 | 0 |
| S239Bpa before UV L185Bpa after UV | 0,9998 | 0 |
| S239Bpa before UV V181Bpa before UV | 0,08767 | 0 |
| S239Bpa before UV V181Bpa after UV | 2,85E-06 | 1 |
| S239Bpa before UV S179Bpa before UV | 1 | 0 |
| S239Bpa before UV S179Bpa after UV | 1 | 0 |
| S239Bpa before UV W176Bpa before UV | 2,45E-04 | 1 |
| S239Bpa before UV W176Bpa after UV | 0,00665 | 1 |
| S239Bpa before UV L174Bpa before UV | 1 | 0 |
| S239Bpa before UV L174Bpa after UV | 0,18123 | 0 |
| S239Bpa before UV A235Bpa before UV | 0,99589 | 0 |
| S239Bpa before UV A235Bpa after UV | 0,22605 | 0 |
| S239Bpa after UV O1 before UV | 0,63781 | 0 |
| S239Bpa after UV O1 after UV | 0,84285 | 0 |
| S239Bpa after UV O1+STIM1 before TG | 0,91844 | 0 |
| S239Bpa after UV O1+STIM1  after TG | 0,21865 | 0 |
| S239Bpa after UV L130Bpa before UV | 0,9999 | 0 |
| S239Bpa after UV L130Bpa after UV | 0,99995 | 0 |
| S239Bpa after UV H134Bpa before UV | 0,29057 | 0 |
| S239Bpa after UV H134Bpa after UV | 0,99989 | 0 |
| S239Bpa after UV F136Bpa before UV | 1 | 0 |
| S239Bpa after UV F136Bpa after UV | 0,9364 | 0 |
| S239Bpa after UV A137Bpa before UV | 0,82785 | 0 |
| S239Bpa after UV A137Bpa after UV | 0 | 1 |
| S239Bpa after UV L138Bpa before UV | 0,02896 | 1 |

| | | | | | |
|---|---|---|---|---|---|
| | | | S239Bpa after UV L138Bpa after UV | 0,4979 | 0 |
| | | | S239Bpa after UV L194Bpa before UV | 1 | 0 |
| | | | S239Bpa after UV L194Bpa after UV | 0,99999 | 0 |
| | | | S239Bpa after UV V191Bpa before UV | 0,00295 | 1 |
| | | | S239Bpa after UV V191Bpa after UV | 0,10981 | 0 |
| | | | S239Bpa after UV L188Bpa before UV | 0,01191 | 1 |
| | | | S239Bpa after UV L188Bpa after UV | 0,0422 | 1 |
| | | | S239Bpa after UV F187Bpa before UV | 0,21104 | 0 |
| | | | S239Bpa after UV F187Bpa after UV | 0,01435 | 1 |
| | | | S239Bpa after UV L185Bpa before UV | 0,03453 | 1 |
| | | | S239Bpa after UV L185Bpa after UV | 0,98051 | 0 |
| | | | S239Bpa after UV V181Bpa before UV | 0,30457 | 0 |
| | | | S239Bpa after UV V181Bpa after UV | 7,04E-06 | 1 |
| | | | S239Bpa after UV S179Bpa before UV | 0,10342 | 0 |
| | | | S239Bpa after UV S179Bpa after UV | 0,57969 | 0 |
| | | | S239Bpa after UV W176Bpa before UV | 7,96E-04 | 1 |
| | | | S239Bpa after UV W176Bpa after UV | 0,0251 | 1 |
| | | | S239Bpa after UV L174Bpa before UV | 0,99828 | 0 |
| | | | S239Bpa after UV L174Bpa after UV | 0,2393 | 0 |
| | | | S239Bpa after UV A235Bpa before UV | 1 | 0 |
| | | | S239Bpa after UV A235Bpa after UV | 0,89402 | 0 |
| | | | S239Bpa after UV S239Bpa before UV | 0,21638 | 0 |
| | | | M243Bpa before UV O1 before UV | 1 | 0 |
| | | | M243Bpa before UV O1 after UV | 0,99984 | 0 |
| | | | M243Bpa before UV O1+STIM1 before TG | 0,35929 | 0 |
| | | | M243Bpa before UV O1+STIM1  after TG | 0,16617 | 0 |
| | | | M243Bpa before UV L130Bpa before UV | 0,99952 | 0 |
| | | | M243Bpa before UV L130Bpa after UV | 0,9468 | 0 |
| | | | M243Bpa before UV H134Bpa before UV | 1 | 0 |
| | | | M243Bpa before UV H134Bpa after UV | 0,02687 | 1 |
| | | | M243Bpa before UV F136Bpa before UV | 0,23036 | 0 |
| | | | M243Bpa before UV F136Bpa after UV | 0,0125 | 1 |
| | | | M243Bpa before UV A137Bpa before UV | 0,35325 | 0 |
| | | | M243Bpa before UV A137Bpa after UV | 0 | 1 |
| | | | M243Bpa before UV L138Bpa before UV | 1 | 0 |
| | | | M243Bpa before UV L138Bpa after UV | 0,03339 | 1 |
| | | | M243Bpa before UV L194Bpa before UV | 0,99912 | 0 |
| | | | M243Bpa before UV L194Bpa after UV | 0,91018 | 0 |
| | | | M243Bpa before UV V191Bpa before UV | 0,98391 | 0 |
| | | | M243Bpa before UV V191Bpa after UV | 0,75722 | 0 |
| | | | M243Bpa before UV L188Bpa before UV | 0,00725 | 1 |
| | | | M243Bpa before UV L188Bpa after UV | 0,02195 | 1 |
| | | | M243Bpa before UV F187Bpa before UV | 0,93052 | 0 |
| | | | M243Bpa before UV F187Bpa after UV | 0,00406 | 1 |
| | | | M243Bpa before UV L185Bpa before UV | 1 | 0 |
| | | | M243Bpa before UV L185Bpa after UV | 0,45596 | 0 |
| | | | M243Bpa before UV V181Bpa before UV | 0,06215 | 0 |
| | | | M243Bpa before UV V181Bpa after UV | 2,30E-06 | 1 |
| | | | M243Bpa before UV S179Bpa before UV | 1 | 0 |
| | | | M243Bpa before UV S179Bpa after UV | 0,32976 | 0 |
| | | | M243Bpa before UV W176Bpa before UV | 1,84E-04 | 1 |
| | | | M243Bpa before UV W176Bpa after UV | 0,00478 | 1 |
| | | | M243Bpa before UV L174Bpa before UV | 1 | 0 |
| | | | M243Bpa before UV L174Bpa after UV | 0,16906 | 0 |
| | | | M243Bpa before UV A235Bpa before UV | 0,9686 | 0 |
| | | | M243Bpa before UV A235Bpa after UV | 0,13321 | 0 |
| | | | M243Bpa before UV S239Bpa before UV | 0,99697 | 0 |
| | | | M243Bpa before UV S239Bpa after UV | 0,01365 | 1 |
| | | | M243Bpa after UV O1 before UV | 1 | 0 |
| | | | M243Bpa after UV O1 after UV | 1 | 0 |
| | | | M243Bpa after UV O1+STIM1 before TG | 1 | 0 |
| | | | M243Bpa after UV O1+STIM1  after TG | 0,18822 | 0 |
| | | | M243Bpa after UV L130Bpa before UV | 1 | 0 |
| | | | M243Bpa after UV L130Bpa after UV | 1 | 0 |
| | | | M243Bpa after UV H134Bpa before UV | 0,99669 | 0 |
| | | | M243Bpa after UV H134Bpa after UV | 1 | 0 |
| | | | M243Bpa after UV F136Bpa before UV | 0,955 | 0 |
| | | | M243Bpa after UV F136Bpa after UV | 0,16322 | 0 |
| | | | M243Bpa after UV A137Bpa before UV | 0,56806 | 0 |
| | | | M243Bpa after UV A137Bpa after UV | 0 | 1 |
| | | | M243Bpa after UV L138Bpa before UV | 0,13423 | 0 |
| | | | M243Bpa after UV L138Bpa after UV | 1 | 0 |
| | | | M243Bpa after UV L194Bpa before UV | 1 | 0 |
| | | | M243Bpa after UV L194Bpa after UV | 0,99038 | 0 |
| | | | M243Bpa after UV V191Bpa before UV | 0,00378 | 1 |
| | | | M243Bpa after UV V191Bpa after UV | 0,68024 | 0 |
| | | | M243Bpa after UV L188Bpa before UV | 0,00907 | 1 |
| | | | M243Bpa after UV L188Bpa after UV | 0,02947 | 1 |
| | | | M243Bpa after UV F187Bpa before UV | 0,98466 | 0 |
| | | | M243Bpa after UV F187Bpa after UV | 0,00718 | 1 |
| | | | M243Bpa after UV L185Bpa before UV | 0,31302 | 0 |
| | | | M243Bpa after UV L185Bpa after UV | 1 | 0 |
| | | | M243Bpa after UV V181Bpa before UV | 0,13245 | 0 |
| | | | M243Bpa after UV V181Bpa after UV | 3,83E-06 | 1 |
| | | | M243Bpa after UV S179Bpa before UV | 0,81513 | 0 |
| | | | M243Bpa after UV S179Bpa after UV | 1 | 0 |
| | | | M243Bpa after UV W176Bpa before UV | 3,55E-04 | 1 |
| | | | M243Bpa after UV W176Bpa after UV | 0,01009 | 1 |
| | | | M243Bpa after UV L174Bpa before UV | 1 | 0 |
| | | | M243Bpa after UV L174Bpa after UV | 0,19812 | 0 |
| | | | M243Bpa after UV A235Bpa before UV | 0,99993 | 0 |
| | | | M243Bpa after UV A235Bpa after UV | 0,39674 | 0 |
| | | | M243Bpa after UV S239Bpa before UV | 0,99022 | 0 |
| | | | M243Bpa after UV S239Bpa after UV | 0,89588 | 0 |
| | | | M243Bpa after UV M243Bpa before UV | 0,04215 | 1 |
| | | | P245Bpa before UV O1 before UV | 1 | 0 |
| | | | P245Bpa before UV O1 after UV | 1 | 0 |
| | | | P245Bpa before UV O1+STIM1 before TG | 1 | 0 |
| | | | P245Bpa before UV O1+STIM1  after TG | 0,18238 | 0 |
| | | | P245Bpa before UV L130Bpa before UV | 1 | 0 |
| | | | P245Bpa before UV L130Bpa after UV | 1 | 0 |
| | | | P245Bpa before UV H134Bpa before UV | 1 | 0 |
| | | | P245Bpa before UV H134Bpa after UV | 0,99864 | 0 |
| | | | P245Bpa before UV F136Bpa before UV | 0,85409 | 0 |
| | | | P245Bpa before UV F136Bpa after UV | 0,09765 | 0 |
| | | | P245Bpa before UV A137Bpa before UV | 0,51244 | 0 |
| | | | P245Bpa before UV A137Bpa after UV | 0 | 1 |
| | | | P245Bpa before UV L138Bpa before UV | 0,90222 | 0 |
| | | | P245Bpa before UV L138Bpa after UV | 1 | 0 |
| | | | P245Bpa before UV L194Bpa before UV | 1 | 0 |
| | | | P245Bpa before UV L194Bpa after UV | 0,98098 | 0 |
| | | | P245Bpa before UV V191Bpa before UV | 0,07357 | 0 |
| | | | P245Bpa before UV V191Bpa after UV | 0,99999 | 0 |
| | | | P245Bpa before UV L188Bpa before UV | 0,00856 | 1 |
| | | | P245Bpa before UV L188Bpa after UV | 0,02735 | 1 |
| | | | P245Bpa before UV F187Bpa before UV | 1 | 0 |
| | | | P245Bpa before UV F187Bpa after UV | 0,00623 | 1 |

| | | | | | |
|---|---|---|---|---|---|
| | | | P245Bpa before UV L185Bpa before UV | 0,96431 | 0 |
| | | | P245Bpa before UV L185Bpa after UV | 1 | 0 |
| | | | P245Bpa before UV V181Bpa before UV | 0,11078 | 0 |
| | | | P245Bpa before UV V181Bpa after UV | 3,36E-06 | 1 |
| | | | P245Bpa before UV S179Bpa before UV | 0,99994 | 0 |
| | | | P245Bpa before UV S179Bpa after UV | 1 | 0 |
| | | | P245Bpa before UV W176Bpa before UV | 3,00E-04 | 1 |
| | | | P245Bpa before UV W176Bpa after UV | 0,00839 | 1 |
| | | | P245Bpa before UV L174Bpa before UV | 1 | 0 |
| | | | P245Bpa before UV L174Bpa after UV | 0,19038 | 0 |
| | | | P245Bpa before UV A235Bpa before UV | 0,99946 | 0 |
| | | | P245Bpa before UV A235Bpa after UV | 0,31552 | 0 |
| | | | P245Bpa before UV S239Bpa before UV | 1 | 0 |
| | | | P245Bpa before UV S239Bpa after UV | 0,64675 | 0 |
| | | | P245Bpa before UV M243Bpa before UV | 0,58916 | 0 |
| | | | P245Bpa before UV M243Bpa after UV | 1 | 0 |
| | | | P245Bpa after UV O1 before UV | 0,20317 | 0 |
| | | | P245Bpa after UV O1 after UV | 0,38879 | 0 |
| | | | P245Bpa after UV O1+STIM1 before TG | 0,37819 | 0 |
| | | | P245Bpa after UV O1+STIM1 after TG | 0,22061 | 0 |
| | | | P245Bpa after UV L130Bpa before UV | 0,99624 | 0 |
| | | | P245Bpa after UV L130Bpa after UV | 0,99572 | 0 |
| | | | P245Bpa after UV H134Bpa before UV | 0,02719 | 1 |
| | | | P245Bpa after UV H134Bpa after UV | 0,96374 | 0 |
| | | | P245Bpa after UV F136Bpa before UV | 1 | 0 |
| | | | P245Bpa after UV F136Bpa after UV | 0,92349 | 0 |
| | | | P245Bpa after UV A137Bpa before UV | 0,83498 | 0 |
| | | | P245Bpa after UV A137Bpa after UV | 0 | 1 |
| | | | P245Bpa after UV L138Bpa before UV | 1,10E-04 | 1 |
| | | | P245Bpa after UV L138Bpa after UV | 0,02419 | 1 |
| | | | P245Bpa after UV L194Bpa before UV | 1 | 0 |
| | | | P245Bpa after UV L194Bpa after UV | 1 | 0 |
| | | | P245Bpa after UV V191Bpa before UV | 5,34E-06 | 1 |
| | | | P245Bpa after UV V191Bpa after UV | 9,88E-04 | 1 |
| | | | P245Bpa after UV L188Bpa before UV | 0,01213 | 1 |
| | | | P245Bpa after UV L188Bpa after UV | 0,04307 | 1 |
| | | | P245Bpa after UV F187Bpa before UV | 0,00382 | 1 |
| | | | P245Bpa after UV F187Bpa after UV | 0,01479 | 1 |
| | | | P245Bpa after UV L185Bpa before UV | 1,88E-04 | 1 |
| | | | P245Bpa after UV L185Bpa after UV | 0,66522 | 0 |
| | | | P245Bpa after UV V181Bpa before UV | 0,31027 | 0 |
| | | | P245Bpa after UV V181Bpa after UV | 7,37E-06 | 1 |
| | | | P245Bpa after UV S179Bpa before UV | 0,00141 | 1 |
| | | | P245Bpa after UV S179Bpa after UV | 0,04882 | 1 |
| | | | P245Bpa after UV W176Bpa before UV | 8,29E-04 | 1 |
| | | | P245Bpa after UV W176Bpa after UV | 0,02591 | 1 |
| | | | P245Bpa after UV L174Bpa before UV | 0,98556 | 0 |
| | | | P245Bpa after UV L174Bpa after UV | 0,24187 | 0 |
| | | | P245Bpa after UV A235Bpa before UV | 1 | 0 |
| | | | P245Bpa after UV A235Bpa after UV | 0,89161 | 0 |
| | | | P245Bpa after UV S239Bpa before UV | 0,00501 | 1 |
| | | | P245Bpa after UV S239Bpa after UV | 1 | 0 |
| | | | P245Bpa after UV M243Bpa before UV | 3,80E-05 | 1 |
| | | | P245Bpa after UV M243Bpa after UV | 0,24984 | 0 |
| | | | P245Bpa after UV P245Bpa before UV | 0,07919 | 0 |
| | | | F250Bpa before UV O1 before UV | 0,30465 | 0 |
| | | | F250Bpa before UV O1 after UV | 0,31922 | 0 |
| | | | F250Bpa before UV O1+STIM1 before TG | 0,33882 | 0 |
| | | | F250Bpa before UV O1+STIM1 after TG | 1 | 0 |
| | | | F250Bpa before UV L130Bpa before UV | 0,34619 | 0 |
| | | | F250Bpa before UV L130Bpa after UV | 0,35536 | 0 |
| | | | F250Bpa before UV H134Bpa before UV | 0,29843 | 0 |
| | | | F250Bpa before UV H134Bpa after UV | 0,36825 | 0 |
| | | | F250Bpa before UV F136Bpa before UV | 0,49132 | 0 |
| | | | F250Bpa before UV F136Bpa after UV | 0,66132 | 0 |
| | | | F250Bpa before UV A137Bpa before UV | 0,99776 | 0 |
| | | | F250Bpa before UV A137Bpa after UV | 0,19625 | 0 |
| | | | F250Bpa before UV L138Bpa before UV | 0,28443 | 0 |
| | | | F250Bpa before UV L138Bpa after UV | 0,32377 | 0 |
| | | | F250Bpa before UV L194Bpa before UV | 0,40471 | 0 |
| | | | F250Bpa before UV L194Bpa after UV | 0,75505 | 0 |
| | | | F250Bpa before UV V191Bpa before UV | 0,26269 | 0 |
| | | | F250Bpa before UV V191Bpa after UV | 0,3006 | 0 |
| | | | F250Bpa before UV L188Bpa before UV | 0,99746 | 0 |
| | | | F250Bpa before UV L188Bpa after UV | 1 | 0 |
| | | | F250Bpa before UV F187Bpa before UV | 0,30613 | 0 |
| | | | F250Bpa before UV F187Bpa after UV | 1 | 0 |
| | | | F250Bpa before UV L185Bpa before UV | 0,28147 | 0 |
| | | | F250Bpa before UV L185Bpa after UV | 0,34467 | 0 |
| | | | F250Bpa before UV V181Bpa before UV | 0,99902 | 0 |
| | | | F250Bpa before UV V181Bpa after UV | 0,99625 | 0 |
| | | | F250Bpa before UV S179Bpa before UV | 0,29354 | 0 |
| | | | F250Bpa before UV S179Bpa after UV | 0,32396 | 0 |
| | | | F250Bpa before UV W176Bpa before UV | 1 | 0 |
| | | | F250Bpa before UV W176Bpa after UV | 1 | 0 |
| | | | F250Bpa before UV L174Bpa before UV | 0,32426 | 0 |
| | | | F250Bpa before UV L174Bpa after UV | 1 | 0 |
| | | | F250Bpa before UV A235Bpa before UV | 0,54114 | 0 |
| | | | F250Bpa before UV A235Bpa after UV | 0,77772 | 0 |
| | | | F250Bpa before UV S239Bpa before UV | 0,30384 | 0 |
| | | | F250Bpa before UV S239Bpa after UV | 0,43338 | 0 |
| | | | F250Bpa before UV M243Bpa before UV | 0,2766 | 0 |
| | | | F250Bpa before UV M243Bpa after UV | 0,34155 | 0 |
| | | | F250Bpa before UV P245Bpa before UV | 0,32434 | 0 |
| | | | F250Bpa before UV P245Bpa after UV | 0,43846 | 0 |
| | | | F250Bpa after UV O1 before UV | 0,94308 | 0 |
| | | | F250Bpa after UV O1 after UV | 0,9527 | 0 |
| | | | F250Bpa after UV O1+STIM1 before TG | 0,96331 | 0 |
| | | | F250Bpa after UV O1+STIM1 after TG | 0,81956 | 0 |
| | | | F250Bpa after UV L130Bpa before UV | 0,96724 | 0 |
| | | | F250Bpa after UV L130Bpa after UV | 0,97097 | 0 |
| | | | F250Bpa after UV H134Bpa before UV | 0,93826 | 0 |
| | | | F250Bpa after UV H134Bpa after UV | 0,97558 | 0 |
| | | | F250Bpa after UV F136Bpa before UV | 0,9968 | 0 |
| | | | F250Bpa after UV F136Bpa after UV | 0,99995 | 0 |
| | | | F250Bpa after UV A137Bpa before UV | 1 | 0 |
| | | | F250Bpa after UV A137Bpa after UV | 3,06E-04 | 1 |
| | | | F250Bpa after UV L138Bpa before UV | 0,92643 | 0 |
| | | | F250Bpa after UV L138Bpa after UV | 0,95513 | 0 |
| | | | F250Bpa after UV L194Bpa before UV | 0,98637 | 0 |
| | | | F250Bpa after UV L194Bpa after UV | 1 | 0 |
| | | | F250Bpa after UV V191Bpa before UV | 0,90473 | 0 |
| | | | F250Bpa after UV V191Bpa after UV | 0,93968 | 0 |
| | | | F250Bpa after UV L188Bpa before UV | 0,30552 | 0 |
| | | | F250Bpa after UV L188Bpa after UV | 0,95516 | 0 |
| | | | F250Bpa after UV F187Bpa before UV | 0,94377 | 0 |
| | | | F250Bpa after UV F187Bpa after UV | 0,99916 | 0 |

| | | | | | | |
|---|---|---|---|---|---|---|
| | | | | F250Bpa after UV L185Bpa before UV | 0,92383 | 0 |
| | | | | F250Bpa after UV L185Bpa after UV | 0,96614 | 0 |
| | | | | F250Bpa after UV V181Bpa before UV | 1 | 0 |
| | | | | F250Bpa after UV V181Bpa after UV | 0,07088 | 0 |
| | | | | F250Bpa after UV S179Bpa before UV | 0,93425 | 0 |
| | | | | F250Bpa after UV S179Bpa after UV | 0,95528 | 0 |
| | | | | F250Bpa after UV W176Bpa before UV | 0,98574 | 0 |
| | | | | F250Bpa after UV W176Bpa after UV | 1 | 0 |
| | | | | F250Bpa after UV L174Bpa before UV | 0,95654 | 0 |
| | | | | F250Bpa after UV L174Bpa after UV | 0,9451 | 0 |
| | | | | F250Bpa after UV A235Bpa before UV | 0,99886 | 0 |
| | | | | F250Bpa after UV A235Bpa after UV | 1 | 0 |
| | | | | F250Bpa after UV S239Bpa before UV | 0,94216 | 0 |
| | | | | F250Bpa after UV S239Bpa after UV | 0,99102 | 0 |
| | | | | F250Bpa after UV M243Bpa before UV | 0,91917 | 0 |
| | | | | F250Bpa after UV M243Bpa after UV | 0,96459 | 0 |
| | | | | F250Bpa after UV P245Bpa before UV | 0,95553 | 0 |
| | | | | F250Bpa after UV P245Bpa after UV | 0,9917 | 0 |
| | | | | F250Bpa after UV F250Bpa before UV | 0,99999 | 0 |
| | | | | A254Bpa before UV O1 before UV | 1,30E-05 | 1 |
| | | | | A254Bpa before UV O1 after UV | 1,55E-05 | 1 |
| | | | | A254Bpa before UV O1+STIM1 before TG | 1,96E-05 | 1 |
| | | | | A254Bpa before UV O1+STIM1 after TG | 0,99993 | 0 |
| | | | | A254Bpa before UV L130Bpa before UV | 2,11E-05 | 1 |
| | | | | A254Bpa before UV L130Bpa after UV | 2,34E-05 | 1 |
| | | | | A254Bpa before UV H134Bpa before UV | 1,21E-05 | 1 |
| | | | | A254Bpa before UV H134Bpa after UV | 2,72E-05 | 1 |
| | | | | A254Bpa before UV F136Bpa before UV | 9,47E-05 | 1 |
| | | | | A254Bpa before UV F136Bpa after UV | 4,79E-04 | 1 |
| | | | | A254Bpa before UV A137Bpa before UV | 0,39347 | 0 |
| | | | | A254Bpa before UV A137Bpa after UV | 0,03307 | 1 |
| | | | | A254Bpa before UV L138Bpa before UV | 1,03E-05 | 1 |
| | | | | A254Bpa before UV L138Bpa after UV | 1,66E-05 | 1 |
| | | | | A254Bpa before UV L194Bpa before UV | 4,15E-05 | 1 |
| | | | | A254Bpa before UV L194Bpa after UV | 0,00284 | 1 |
| | | | | A254Bpa before UV V191Bpa before UV | 7,72E-06 | 1 |
| | | | | A254Bpa before UV V191Bpa after UV | 1,25E-05 | 1 |
| | | | | A254Bpa before UV L188Bpa before UV | 0,98238 | 0 |
| | | | | A254Bpa before UV L188Bpa after UV | 1 | 0 |
| | | | | A254Bpa before UV F187Bpa before UV | 1,34E-05 | 1 |
| | | | | A254Bpa before UV F187Bpa after UV | 1 | 0 |
| | | | | A254Bpa before UV L185Bpa before UV | 9,82E-06 | 1 |
| | | | | A254Bpa before UV L185Bpa after UV | 2,09E-05 | 1 |
| | | | | A254Bpa before UV V181Bpa before UV | 0,42303 | 0 |
| | | | | A254Bpa before UV V181Bpa after UV | 0,94258 | 0 |
| | | | | A254Bpa before UV S179Bpa before UV | 1,15E-05 | 1 |
| | | | | A254Bpa before UV S179Bpa after UV | 1,66E-05 | 1 |
| | | | | A254Bpa before UV W176Bpa before UV | 1 | 0 |
| | | | | A254Bpa before UV W176Bpa after UV | 0,99936 | 0 |
| | | | | A254Bpa before UV L174Bpa before UV | 1,63E-05 | 1 |
| | | | | A254Bpa before UV L174Bpa after UV | 1 | 0 |
| | | | | A254Bpa before UV A235Bpa before UV | 1,85E-04 | 1 |
| | | | | A254Bpa before UV A235Bpa after UV | 0,00185 | 1 |
| | | | | A254Bpa before UV S239Bpa before UV | 1,30E-05 | 1 |
| | | | | A254Bpa before UV S239Bpa after UV | 5,34E-05 | 1 |
| | | | | A254Bpa before UV M243Bpa before UV | 9,27E-06 | 1 |
| | | | | A254Bpa before UV M243Bpa after UV | 2,03E-05 | 1 |
| | | | | A254Bpa before UV P245Bpa before UV | 1,66E-05 | 1 |
| | | | | A254Bpa before UV P245Bpa after UV | 5,62E-05 | 1 |
| | | | | A254Bpa before UV F250Bpa before UV | 1 | 0 |
| | | | | A254Bpa before UV F250Bpa after UV | 0,95782 | 0 |
| | | | | A254Bpa after UV O1 before UV | 0,05814 | 0 |
| | | | | A254Bpa after UV O1 after UV | 0,0754 | 0 |
| | | | | A254Bpa after UV O1+STIM1 before TG | 0,09984 | 0 |
| | | | | A254Bpa after UV O1+STIM1 after TG | 0,48124 | 0 |
| | | | | A254Bpa after UV L130Bpa before UV | 0,13497 | 0 |
| | | | | A254Bpa after UV L130Bpa after UV | 0,14532 | 0 |
| | | | | A254Bpa after UV H134Bpa before UV | 0,04875 | 1 |
| | | | | A254Bpa after UV H134Bpa after UV | 0,15873 | 0 |
| | | | | A254Bpa after UV F136Bpa before UV | 0,69008 | 0 |
| | | | | A254Bpa after UV F136Bpa after UV | 0,99811 | 0 |
| | | | | A254Bpa after UV A137Bpa before UV | 1 | 0 |
| | | | | A254Bpa after UV A137Bpa after UV | 7,07E-07 | 1 |
| | | | | A254Bpa after UV L138Bpa before UV | 0,03456 | 1 |
| | | | | A254Bpa after UV L138Bpa after UV | 0,07455 | 0 |
| | | | | A254Bpa after UV L194Bpa before UV | 0,4103 | 0 |
| | | | | A254Bpa after UV L194Bpa after UV | 1 | 0 |
| | | | | A254Bpa after UV V191Bpa before UV | 0,02129 | 1 |
| | | | | A254Bpa after UV V191Bpa after UV | 0,04798 | 1 |
| | | | | A254Bpa after UV L188Bpa before UV | 0,05858 | 0 |
| | | | | A254Bpa after UV L188Bpa after UV | 0,31157 | 0 |
| | | | | A254Bpa after UV F187Bpa before UV | 0,05411 | 0 |
| | | | | A254Bpa after UV F187Bpa after UV | 0,44699 | 0 |
| | | | | A254Bpa after UV L185Bpa before UV | 0,03303 | 1 |
| | | | | A254Bpa after UV L185Bpa after UV | 0,11186 | 0 |
| | | | | A254Bpa after UV V181Bpa before UV | 1 | 0 |
| | | | | A254Bpa after UV V181Bpa after UV | 2,98E-04 | 1 |
| | | | | A254Bpa after UV S179Bpa before UV | 0,04257 | 1 |
| | | | | A254Bpa after UV S179Bpa after UV | 0,07572 | 0 |
| | | | | A254Bpa after UV W176Bpa before UV | 0,10083 | 0 |
| | | | | A254Bpa after UV W176Bpa after UV | 0,92581 | 0 |
| | | | | A254Bpa after UV L174Bpa before UV | 0,11083 | 0 |
| | | | | A254Bpa after UV L174Bpa after UV | 0,60403 | 0 |
| | | | | A254Bpa after UV A235Bpa before UV | 0,94661 | 0 |
| | | | | A254Bpa after UV A235Bpa after UV | 1 | 0 |
| | | | | A254Bpa after UV S239Bpa before UV | 0,05225 | 0 |
| | | | | A254Bpa after UV S239Bpa after UV | 0,38096 | 0 |
| | | | | A254Bpa after UV M243Bpa before UV | 0,02921 | 1 |
| | | | | A254Bpa after UV M243Bpa after UV | 0,10244 | 0 |
| | | | | A254Bpa after UV P245Bpa before UV | 0,07698 | 0 |
| | | | | A254Bpa after UV P245Bpa after UV | 0,38302 | 0 |
| | | | | A254Bpa after UV F250Bpa before UV | 0,94922 | 0 |
| | | | | A254Bpa after UV F250Bpa after UV | 1 | 0 |
| | | | | A254Bpa after UV A254Bpa before UV | 0,02541 | 1 |
| S3j | Welch-ANOVA | 3,85E-09 | F(6, 16.91)=40.4 | 0,7 0,5 | 8,07E-05 | 1 |
| | | | | 1 0,5 | 3,89E-05 | 1 |
| | | | | 1 0,7 | 0,53902 | 0 |
| | | | | 1,4 0,5 | 1,74E-04 | 1 |
| | | | | 1,4 0,7 | 0,00266 | 1 |
| | | | | 1,4 1 | 0,37823 | 0 |
| | | | | 1,8 0,5 | 4,78E-05 | 1 |
| | | | | 1,8 0,7 | 3,36E-04 | 1 |
| | | | | 1,8 1 | 0,12948 | 0 |
| | | | | 1,8 1,4 | 0,38986 | 0 |
| | | | | 2,2 0,5 | 2,92E-05 | 1 |

| | | | | | | |
|---|---|---|---|---|---|---|
| | | | | 2,2 0,7 | 2,18E-05 | 1 |
| | | | | 2,2 1 | 0,02276 | 1 |
| | | | | 2,2 1,4 | 0,00273 | 1 |
| | | | | 2,2 1,8 | 0,22542 | 0 |
| | | | | 4 0,5 | 1,69E-05 | 1 |
| | | | | 4 0,7 | 0,00561 | 1 |
| | | | | 4 1 | 0,05989 | 0 |
| | | | | 4 1,4 | 0,27365 | 0 |
| | | | | 4 1,8 | 0,61222 | 0 |
| | | | | 4 2,2 | 0,99925 | 0 |
| S3k | Welch-ANOVA | 0,91757 | F(6, 18.35)=0,32 | 0,7 0,5 | 0,99852 | 0 |
| | | | | 1 0,5 | 0,99895 | 0 |
| | | | | 1 0,7 | 0,98009 | 0 |
| | | | | 1,4 0,5 | 1 | 0 |
| | | | | 1,4 0,7 | 0,99929 | 0 |
| | | | | 1,4 1 | 0,99859 | 0 |
| | | | | 1,8 0,5 | 0,94059 | 0 |
| | | | | 1,8 0,7 | 0,99928 | 0 |
| | | | | 1,8 1 | 0,8498 | 0 |
| | | | | 1,8 1,4 | 0,95816 | 0 |
| | | | | 2,2 0,5 | 0,99938 | 0 |
| | | | | 2,2 0,7 | 1 | 0 |
| | | | | 2,2 1 | 0,99123 | 0 |
| | | | | 2,2 1,4 | 0,99966 | 0 |
| | | | | 2,2 1,8 | 0,99984 | 0 |
| | | | | 4 0,5 | 0,99575 | 0 |
| | | | | 4 0,7 | 0,99954 | 0 |
| | | | | 4 1 | 0,98804 | 0 |
| | | | | 4 1,4 | 0,99642 | 0 |
| | | | | 4 1,8 | 0,99999 | 0 |
| | | | | 4 2,2 | 0,99973 | 0 |
| S4e | Welch-ANOVA | 2,65E-07 | F(7, 26.77)=13,32 | 0,5 0 | 1 | 0 |
| | | | | 1 0 | 0,2782 | 0 |
| | | | | 1 0,5 | 0,40043 | 0 |
| | | | | 1,5 0 | 0,21566 | 0 |
| | | | | 1,5 0,5 | 0,22591 | 0 |
| | | | | 1,5 1 | 0,68009 | 0 |
| | | | | 2 0 | 0,17989 | 0 |
| | | | | 2 0,5 | 0,18698 | 0 |
| | | | | 2 1 | 0,51059 | 0 |
| | | | | 2 1,5 | 0,99828 | 0 |
| | | | | 10 0 | 1,06E-05 | 1 |
| | | | | 10 0,5 | 1,16E-05 | 1 |
| | | | | 10 1 | 1,67E-04 | 1 |
| | | | | 10 1,5 | 0,10588 | 0 |
| | | | | 10 2 | 0,54355 | 0 |
| | | | | 20 0 | 0,0082 | 1 |
| | | | | 20 0,5 | 0,00801 | 1 |
| | | | | 20 1 | 0,01381 | 1 |
| | | | | 20 1,5 | 0,04068 | 1 |
| | | | | 20 2 | 0,09753 | 0 |
| | | | | 20 10 | 0,55898 | 0 |
| | | | | 110 0 | 0,05955 | 0 |
| | | | | 110 0,5 | 0,05973 | 0 |
| | | | | 110 1 | 0,08528 | 0 |
| | | | | 110 1,5 | 0,16213 | 0 |
| | | | | 110 2 | 0,24006 | 0 |
| | | | | 110 10 | 0,6016 | 0 |
| | | | | 110 20 | 0,99744 | 0 |
| S4g | one-way ANOVA | <0.0001 | F(6, 37)=16,11 | 0,7 0,5 | 0,10828 | 0 |
| | | | | 1 0,5 | 0,00678 | 1 |
| | | | | 1 0,7 | 0,2414 | 0 |
| | | | | 1,4 0,5 | 4,24E-04 | 1 |
| | | | | 1,4 0,7 | 0,04313 | 1 |
| | | | | 1,4 1 | 0,39559 | 0 |
| | | | | 1,8 0,5 | <0.0001 | 1 |
| | | | | 1,8 0,7 | 0,00128 | 1 |
| | | | | 1,8 1 | 0,02183 | 1 |
| | | | | 1,8 1,4 | 0,09885 | 0 |
| | | | | 2,2 0,5 | <0.0001 | 1 |
| | | | | 2,2 0,7 | <0.0001 | 1 |
| | | | | 2,2 1 | 0,00105 | 1 |
| | | | | 2,2 1,4 | 0,00739 | 1 |
| | | | | 2,2 1,8 | 0,43422 | 0 |
| | | | | 5 0,5 | <0.0001 | 1 |
| | | | | 5 0,7 | <0.0001 | 1 |
| | | | | 5 1 | <0.0001 | 1 |
| | | | | 5 1,4 | 1,47E-04 | 1 |
| | | | | 5 1,8 | 0,03047 | 1 |
| | | | | 5 2,2 | 0,09004 | 0 |
| S6e | Welch-ANOVA | 0,000446631 | F(4, 29,942)=6,932 | S1/O1 DKO HEK293 | 0,96629 | 0 |
| | | | | S1/S2 DKO HEK293 | 0,28365 | 0 |
| | | | | S1/S2 DKO S1/O1 DKO | 0,47008 | 0 |
| | | | | O1/O2/O3 TKO HEK293 | 0,03706 | 1 |
| | | | | O1/O2/O3 TKO S1/O1 DKO | 0,04206 | 1 |
| | | | | O1/O2/O3 TKO S1/S2 DKO | 0,32121 | 0 |
| | | | | RBL 2H3 HEK293 | 0,02155 | 1 |
| | | | | RBL 2H3 S1/O1 DKO | 0,01708 | 1 |
| | | | | RBL 2H3 S1/S2 DKO | 0,06652 | 0 |
| | | | | RBL 2H3 O1/O2/O3 TKO | 0,99936 | 0 |
| S6h | Welch-ANOVA | 0 | F(15, 31.96)=111.47 | A137Bpa -UV WT | 0,02677 | 1 |
| | | | | A137Bpa +UV WT | 0,99214 | 0 |
| | | | | A137Bpa +UV A137Bpa -UV | 0,04638 | 1 |
| | | | | A137Bpa +S1 t=0 WT | 0,01685 | 1 |
| | | | | A137Bpa +S1 t=0 A137Bpa -UV | 0,05062 | 0 |
| | | | | A137Bpa +S1 t=0 A137Bpa +UV | 0,03793 | 1 |
| | | | | A137Bpa+S1 -UV WT | 0,76272 | 0 |
| | | | | A137Bpa+S1 -UV A137Bpa -UV | 0,56615 | 0 |
| | | | | A137Bpa+S1 -UV A137Bpa +UV | 0,27516 | 0 |
| | | | | A137Bpa+S1 -UV A137Bpa +S1 t=0 | 0,42674 | 0 |
| | | | | A137Bpa+S1+UV WT | 1 | 0 |
| | | | | A137Bpa+S1+UV A137Bpa -UV | 0,06086 | 0 |
| | | | | A137Bpa+S1+UV A137Bpa +UV | 0,97748 | 0 |
| | | | | A137Bpa+S1+UV A137Bpa +S1 t=0 | 0,04838 | 1 |
| | | | | A137Bpa+S1+UV A137Bpa+S1 -UV | 0,66298 | 0 |
| | | | | L174Bpa -UV WT | 0,01951 | 1 |
| | | | | L174Bpa -UV A137Bpa -UV | 0,72486 | 0 |
| | | | | L174Bpa -UV A137Bpa +UV | 0,04015 | 1 |
| | | | | L174Bpa -UV A137Bpa +S1 t=0 | 0,98126 | 0 |
| | | | | L174Bpa -UV A137Bpa+S1 -UV | 0,47147 | 0 |
| | | | | L174Bpa -UV A137Bpa+S1+UV | 0,05109 | 0 |

| | | | | | | |
|---|---|---|---|---|---|---|
| | | | | L174Bpa +UV WT | 0,12614 | 0 |
| | | | | L174Bpa +UV A137Bpa -UV | 0,36278 | 0 |
| | | | | L174Bpa +UV A137Bpa +UV | 0,08591 | 0 |
| | | | | L174Bpa +UV A137Bpa +S1 t=0 | 0,16924 | 0 |
| | | | | L174Bpa +UV A137Bpa+S1 -UV | 0,9862 | 0 |
| | | | | L174Bpa +UV A137Bpa+S1+UV | 0,12933 | 0 |
| | | | | L174Bpa +UV L174Bpa -UV | 0,21591 | 0 |
| | | | | L174Bpa +S1 t=0 WT | 0,01729 | 1 |
| | | | | L174Bpa +S1 t=0 A137Bpa -UV | 0,06871 | 0 |
| | | | | L174Bpa +S1 t=0 A137Bpa +UV | 0,03833 | 1 |
| | | | | L174Bpa +S1 t=0 A137Bpa +S1 t=0 | 1 | 0 |
| | | | | L174Bpa +S1 t=0 A137Bpa+S1 -UV | 0,43404 | 0 |
| | | | | L174Bpa +S1 t=0 A137Bpa+S1+UV | 0,04891 | 1 |
| | | | | L174Bpa +S1 t=0 L174Bpa -UV | 0,99755 | 0 |
| | | | | L174Bpa +S1 t=0 L174Bpa +UV | 0,17602 | 0 |
| | | | | L174Bpa+S1 -UV WT | 0,9536 | 0 |
| | | | | L174Bpa+S1 -UV A137Bpa -UV | 0,00458 | 1 |
| | | | | L174Bpa+S1 -UV A137Bpa +UV | 1 | 0 |
| | | | | L174Bpa+S1 -UV A137Bpa +S1 t=0 | 0,0036 | 1 |
| | | | | L174Bpa+S1 -UV A137Bpa+S1 -UV | 0,09094 | 0 |
| | | | | L174Bpa+S1 -UV A137Bpa+S1+UV | 0,87498 | 0 |
| | | | | L174Bpa+S1 -UV L174Bpa -UV | 0,00372 | 1 |
| | | | | L174Bpa+S1 -UV L174Bpa +UV | 0,00775 | 1 |
| | | | | L174Bpa+S1 -UV L174Bpa +S1 t=0 | 0,00363 | 1 |
| | | | | L174Bpa+S1+UV WT | 0,5799 | 0 |
| | | | | L174Bpa+S1+UV A137Bpa -UV | 0,01625 | 1 |
| | | | | L174Bpa+S1+UV A137Bpa +UV | 0,99412 | 0 |
| | | | | L174Bpa+S1+UV A137Bpa +S1 t=0 | 0,01346 | 1 |
| | | | | L174Bpa+S1+UV A137Bpa+S1 -UV | 0,07033 | 0 |
| | | | | L174Bpa+S1+UV A137Bpa+S1+UV | 0,49547 | 0 |
| | | | | L174Bpa+S1+UV L174Bpa -UV | 0,01417 | 1 |
| | | | | L174Bpa+S1+UV L174Bpa +UV | 0,02747 | 1 |
| | | | | L174Bpa+S1+UV L174Bpa +S1 t=0 | 0,01359 | 1 |
| | | | | L174Bpa+S1+UV L174Bpa+S1 -UV | 0,98878 | 0 |
| | | | | A254Azi-UV WT | 0,0188 | 1 |
| | | | | A254Azi-UV A137Bpa -UV | 0,15767 | 0 |
| | | | | A254Azi-UV A137Bpa +UV | 0,0398 | 1 |
| | | | | A254Azi-UV A137Bpa +S1 t=0 | 0,61585 | 0 |
| | | | | A254Azi-UV A137Bpa+S1 -UV | 0,45654 | 0 |
| | | | | A254Azi-UV A137Bpa+S1+UV | 0,05118 | 0 |
| | | | | A254Azi-UV L174Bpa -UV | 1 | 0 |
| | | | | A254Azi-UV L174Bpa +UV | 0,20129 | 0 |
| | | | | A254Azi-UV L174Bpa +S1 t=0 | 0,9728 | 0 |
| | | | | A254Azi-UV L174Bpa+S1 -UV | 0,00383 | 1 |
| | | | | A254Azi-UV L174Bpa+S1+UV | 0,01409 | 1 |
| | | | | A254Azi +UV WT | 1,78E-04 | 1 |
| | | | | A254Azi +UV A137Bpa -UV | 0 | 1 |
| | | | | A254Azi +UV A137Bpa +UV | 0,04271 | 1 |
| | | | | A254Azi +UV A137Bpa +S1 t=0 | 0 | 1 |
| | | | | A254Azi +UV A137Bpa+S1 -UV | 0,0011 | 1 |
| | | | | A254Azi +UV A137Bpa+S1+UV | 0,00321 | 1 |
| | | | | A254Azi +UV L174Bpa -UV | 0 | 1 |
| | | | | A254Azi +UV L174Bpa +UV | 0 | 1 |
| | | | | A254Azi +UV L174Bpa +S1 t=0 | 0 | 1 |
| | | | | A254Azi +UV L174Bpa+S1 -UV | 0,00346 | 1 |
| | | | | A254Azi +UV L174Bpa+S1+UV | 0,27882 | 0 |
| | | | | A254Azi +UV A254Azi-UV | 0 | 1 |
| | | | | A254Azi +S1 t=0 WT | 0,01983 | 1 |
| | | | | A254Azi +S1 t=0 A137Bpa -UV | 0,34487 | 0 |
| | | | | A254Azi +S1 t=0 A137Bpa +UV | 0,04071 | 1 |
| | | | | A254Azi +S1 t=0 A137Bpa +S1 t=0 | 0,49539 | 0 |
| | | | | A254Azi +S1 t=0 A137Bpa+S1 -UV | 0,47254 | 0 |
| | | | | A254Azi +S1 t=0 A137Bpa+S1+UV | 0,05244 | 0 |
| | | | | A254Azi +S1 t=0 L174Bpa -UV | 1 | 0 |
| | | | | A254Azi +S1 t=0 L174Bpa +UV | 0,21954 | 0 |
| | | | | A254Azi +S1 t=0 L174Bpa +S1 t=0 | 0,8594 | 0 |
| | | | | A254Azi +S1 t=0 L174Bpa+S1 -UV | 0,00392 | 1 |
| | | | | A254Azi +S1 t=0 L174Bpa+S1+UV | 0,01439 | 1 |
| | | | | A254Azi +S1 t=0 A254Azi-UV | 0,99978 | 0 |
| | | | | A254Azi +S1 t=0 A254Azi +UV | 0 | 1 |
| | | | | A254Azi+S1 -UV WT | 0,98165 | 0 |
| | | | | A254Azi+S1 -UV A137Bpa -UV | 0,01985 | 1 |
| | | | | A254Azi+S1 -UV A137Bpa +UV | 1 | 0 |
| | | | | A254Azi+S1 -UV A137Bpa +S1 t=0 | 0,01526 | 1 |
| | | | | A254Azi+S1 -UV A137Bpa+S1 -UV | 0,20528 | 0 |
| | | | | A254Azi+S1 -UV A137Bpa+S1+UV | 0,95545 | 0 |
| | | | | A254Azi+S1 -UV L174Bpa -UV | 0,01646 | 1 |
| | | | | A254Azi+S1 -UV L174Bpa +UV | 0,0449 | 1 |
| | | | | A254Azi+S1 -UV L174Bpa +S1 t=0 | 0,01547 | 1 |
| | | | | A254Azi+S1 -UV L174Bpa+S1 -UV | 1 | 0 |
| | | | | A254Azi+S1 -UV L174Bpa+S1+UV | 0,99859 | 0 |
| | | | | A254Azi+S1 -UV A254Azi-UV | 0,01625 | 1 |
| | | | | A254Azi+S1 -UV A254Azi +UV | 0,02887 | 1 |
| | | | | A254Azi+S1 -UV A254Azi +S1 t=0 | 0,01674 | 1 |
| | | | | A254Azi+S1+UV WT | 0,78591 | 0 |
| | | | | A254Azi+S1+UV A137Bpa -UV | 0,01342 | 1 |
| | | | | A254Azi+S1+UV A137Bpa +UV | 0,99991 | 0 |
| | | | | A254Azi+S1+UV A137Bpa +S1 t=0 | 0,01064 | 1 |
| | | | | A254Azi+S1+UV A137Bpa+S1 -UV | 0,09626 | 0 |
| | | | | A254Azi+S1+UV A137Bpa+S1+UV | 0,69568 | 0 |
| | | | | A254Azi+S1+UV L174Bpa -UV | 0,01136 | 1 |
| | | | | A254Azi+S1+UV L174Bpa +UV | 0,02661 | 1 |
| | | | | A254Azi+S1+UV L174Bpa +S1 t=0 | 0,01077 | 1 |
| | | | | A254Azi+S1+UV L174Bpa+S1 -UV | 0,99986 | 0 |
| | | | | A254Azi+S1+UV L174Bpa+S1+UV | 1 | 0 |
| | | | | A254Azi+S1+UV A254Azi-UV | 0,01125 | 1 |
| | | | | A254Azi+S1+UV A254Azi +UV | 0,12068 | 0 |
| | | | | A254Azi+S1+UV A254Azi +S1 t=0 | 0,01155 | 1 |
| | | | | A254Azi+S1+UV A254Azi+S1 -UV | 0,99999 | 0 |
| S8a | Man-Whitney test | 0,02374 | | A137Bpa ANSGA + STIM1 after UV-light - A137Bpa ANSGA + STIM before UV-light | | |
| | | 0,04533 | | A254Azi + STIM1 after UV-light - A254Azi + STIM1 before UV-light | | |
| | | 0,00217 | | A254Azi ANSGA + STIM1 is after UV-light - A254Azi ANSGA + STIM1 before UV-light | | |
| S8a STIM1+ A137Bpa XXX | Welch-ANOVA | 6,64E-07 | F(11, 20.08)=13.30 | + - | 0,82924 | 0 |
| | | | | A137Bpa V102A - - | 0,96128 | 0 |
| | | | | A137Bpa V102A - + | 0,53713 | 0 |
| | | | | A137Bpa V102A + - | 0,93468 | 0 |
| | | | | A137Bpa V102A + + | 0,48873 | 0 |
| | | | | A137Bpa V102A + A137Bpa V102A - | 1 | 0 |
| | | | | A137Bpa H134A - - | 0,00846 | 1 |
| | | | | A137Bpa H134A - + | 0,0837 | 0 |
| | | | | A137Bpa H134A - A137Bpa V102A - | 0,7153 | 0 |
| | | | | A137Bpa H134A - A137Bpa V102A + | 0,57176 | 0 |
| | | | | A137Bpa H134A + - | 0,0134 | 1 |
| | | | | A137Bpa H134A + + | 0,09928 | 0 |

| | | | | | | |
|---|---|---|---|---|---|---|
| | | | | A137Bpa H134A + A137Bpa V102A - | 0,82306 | 0 |
| | | | | A137Bpa H134A + A137Bpa V102A + | 0,70177 | 0 |
| | | | | A137Bpa H134A + A137Bpa H134A - | 0,84054 | 0 |
| | | | | A137Bpa V181K - - | 0,00674 | 1 |
| | | | | A137Bpa V181K - + | 0,06651 | 0 |
| | | | | A137Bpa V181K - A137Bpa V102A - | 0,54139 | 0 |
| | | | | A137Bpa V181K - A137Bpa V102A + | 0,39319 | 0 |
| | | | | A137Bpa V181K - A137Bpa H134A - | 0,24811 | 0 |
| | | | | A137Bpa V181K - A137Bpa H134A + | 0,00405 | 1 |
| | | | | A137Bpa V181K + - | 0,00681 | 1 |
| | | | | A137Bpa V181K + + | 0,06733 | 0 |
| | | | | A137Bpa V181K + A137Bpa V102A - | 0,55031 | 0 |
| | | | | A137Bpa V181K + A137Bpa V102A + | 0,40156 | 0 |
| | | | | A137Bpa V181K + A137Bpa H134A - | 0,30926 | 0 |
| | | | | A137Bpa V181K + A137Bpa H134A + | 0,00457 | 1 |
| | | | | A137Bpa V181K + A137Bpa V181K - | 0,99999 | 0 |
| | | | | A137Bpa P245L - - | 0,00602 | 1 |
| | | | | A137Bpa P245L - + | 0,06359 | 0 |
| | | | | A137Bpa P245L - A137Bpa V102A - | 0,51185 | 0 |
| | | | | A137Bpa P245L - A137Bpa V102A + | 0,36595 | 0 |
| | | | | A137Bpa P245L - A137Bpa H134A - | 0,14044 | 0 |
| | | | | A137Bpa P245L - A137Bpa H134A + | 0,00222 | 1 |
| | | | | A137Bpa P245L - A137Bpa V181K - | 0,9304 | 0 |
| | | | | A137Bpa P245L - A137Bpa V181K + | 0,87039 | 0 |
| | | | | A137Bpa P245L + - | 0,00702 | 1 |
| | | | | A137Bpa P245L + + | 0,07175 | 0 |
| | | | | A137Bpa P245L + A137Bpa V102A - | 0,59962 | 0 |
| | | | | A137Bpa P245L + A137Bpa V102A + | 0,44931 | 0 |
| | | | | A137Bpa P245L + A137Bpa H134A - | 0,82508 | 0 |
| | | | | A137Bpa P245L + A137Bpa H134A + | 0,07668 | 0 |
| | | | | A137Bpa P245L + A137Bpa V181K - | 0,87227 | 0 |
| | | | | A137Bpa P245L + A137Bpa V181K + | 0,94974 | 0 |
| | | | | A137Bpa P245L + A137Bpa P245L - | 0,64366 | 0 |
| | | | | A137Bpa ANSGA - - | 0,01215 | 1 |
| | | | | A137Bpa ANSGA - + | 0,11265 | 0 |
| | | | | A137Bpa ANSGA - A137Bpa V102A - | 0,91488 | 0 |
| | | | | A137Bpa ANSGA - A137Bpa V102A + | 0,83779 | 0 |
| | | | | A137Bpa ANSGA - A137Bpa H134A - | 0,87482 | 0 |
| | | | | A137Bpa ANSGA - A137Bpa H134A + | 0,99925 | 0 |
| | | | | A137Bpa ANSGA - A137Bpa V181K - | 0,25692 | 0 |
| | | | | A137Bpa ANSGA - A137Bpa V181K + | 0,27794 | 0 |
| | | | | A137Bpa ANSGA - A137Bpa P245L - | 0,19963 | 0 |
| | | | | A137Bpa ANSGA - A137Bpa P245L + | 0,43197 | 0 |
| | | | | A137Bpa ANSGA + - | 0,23929 | 0 |
| | | | | A137Bpa ANSGA + + | 0,73775 | 0 |
| | | | | A137Bpa ANSGA + A137Bpa V102A - | 0,13461 | 0 |
| | | | | A137Bpa ANSGA + A137Bpa V102A + | 0,13173 | 0 |
| | | | | A137Bpa ANSGA + A137Bpa H134A - | 0,05774 | 0 |
| | | | | A137Bpa ANSGA + A137Bpa H134A + | 0,06374 | 0 |
| | | | | A137Bpa ANSGA + A137Bpa V181K - | 0,05048 | 0 |
| | | | | A137Bpa ANSGA + A137Bpa V181K + | 0,05084 | 0 |
| | | | | A137Bpa ANSGA + A137Bpa P245L - | 0,04916 | 1 |
| | | | | A137Bpa ANSGA + A137Bpa P245L + | 0,05278 | 0 |
| | | | | A137Bpa ANSGA + A137Bpa ANSGA - | 0,06801 | 0 |
| S8a STIM1+ L174Bpa XXX | Welch-ANOVA | 1,88E-07 | F(11, 32.27)=9.82 | + - | 0,94136 | 0 |
| | | | | L174Bpa V102A - - | 0,28475 | 0 |
| | | | | L174Bpa V102A - + | 0,01032 | 1 |
| | | | | L174Bpa V102A + - | 0,42072 | 0 |
| | | | | L174Bpa V102A + + | 0,01684 | 1 |
| | | | | L174Bpa V102A + L174Bpa V102A - | 0,99999 | 0 |
| | | | | L174Bpa H134A - - | 0,23064 | 0 |
| | | | | L174Bpa H134A - + | 0,01409 | 1 |
| | | | | L174Bpa H134A - L174Bpa V102A - | 1 | 0 |
| | | | | L174Bpa H134A - L174Bpa V102A + | 0,99671 | 0 |
| | | | | L174Bpa H134A + - | 0,24107 | 0 |
| | | | | L174Bpa H134A + + | 0,01521 | 1 |
| | | | | L174Bpa H134A + L174Bpa V102A - | 1 | 0 |
| | | | | L174Bpa H134A + L174Bpa V102A + | 0,99856 | 0 |
| | | | | L174Bpa H134A + L174Bpa H134A - | 1 | 0 |
| | | | | L174Bpa V181K - - | 0,99999 | 0 |
| | | | | L174Bpa V181K - + | 0,5646 | 0 |
| | | | | L174Bpa V181K - L174Bpa V102A - | 0,19384 | 0 |
| | | | | L174Bpa V181K - L174Bpa V102A + | 0,40693 | 0 |
| | | | | L174Bpa V181K - L174Bpa H134A - | 0,11367 | 0 |
| | | | | L174Bpa V181K - L174Bpa H134A + | 0,12318 | 0 |
| | | | | L174Bpa V181K + - | 1 | 0 |
| | | | | L174Bpa V181K + + | 0,6687 | 0 |
| | | | | L174Bpa V181K + L174Bpa V102A - | 0,1119 | 0 |
| | | | | L174Bpa V181K + L174Bpa V102A + | 0,26775 | 0 |
| | | | | L174Bpa V181K + L174Bpa H134A - | 0,06458 | 0 |
| | | | | L174Bpa V181K + L174Bpa H134A + | 0,07048 | 0 |
| | | | | L174Bpa V181K + L174Bpa V181K - | 1 | 0 |
| | | | | L174Bpa P245L - - | 0,99988 | 0 |
| | | | | L174Bpa P245L - + | 0,40881 | 0 |
| | | | | L174Bpa P245L - L174Bpa V102A - | 0,12263 | 0 |
| | | | | L174Bpa P245L - L174Bpa V102A + | 0,35219 | 0 |
| | | | | L174Bpa P245L - L174Bpa H134A - | 0,04735 | 1 |
| | | | | L174Bpa P245L - L174Bpa H134A + | 0,05307 | 0 |
| | | | | L174Bpa P245L - L174Bpa V181K - | 1 | 0 |
| | | | | L174Bpa P245L - L174Bpa V181K + | 1 | 0 |
| | | | | L174Bpa P245L + - | 1 | 0 |
| | | | | L174Bpa P245L + + | 0,6438 | 0 |
| | | | | L174Bpa P245L + L174Bpa V102A - | 0,08572 | 0 |
| | | | | L174Bpa P245L + L174Bpa V102A + | 0,24478 | 0 |
| | | | | L174Bpa P245L + L174Bpa H134A - | 0,03839 | 1 |
| | | | | L174Bpa P245L + L174Bpa H134A + | 0,04271 | 1 |
| | | | | L174Bpa P245L + L174Bpa V181K - | 1 | 0 |
| | | | | L174Bpa P245L + L174Bpa V181K + | 1 | 0 |
| | | | | L174Bpa P245L + L174Bpa P245L - | 1 | 0 |
| | | | | L174Bpa ANSGA - - | 0,96076 | 0 |
| | | | | L174Bpa ANSGA - + | 0,13825 | 0 |
| | | | | L174Bpa ANSGA - L174Bpa V102A - | 0,55897 | 0 |
| | | | | L174Bpa ANSGA - L174Bpa V102A + | 0,86685 | 0 |
| | | | | L174Bpa ANSGA - L174Bpa H134A - | 0,30756 | 0 |
| | | | | L174Bpa ANSGA - L174Bpa H134A + | 0,33352 | 0 |
| | | | | L174Bpa ANSGA - L174Bpa V181K - | 0,99709 | 0 |
| | | | | L174Bpa ANSGA - L174Bpa V181K + | 0,98084 | 0 |
| | | | | L174Bpa ANSGA - L174Bpa P245L - | 0,99862 | 0 |
| | | | | L174Bpa ANSGA - L174Bpa P245L + | 0,98278 | 0 |
| | | | | L174Bpa ANSGA + - | 1 | 0 |
| | | | | L174Bpa ANSGA + + | 0,91186 | 0 |
| | | | | L174Bpa ANSGA + L174Bpa V102A - | 0,19005 | 0 |
| | | | | L174Bpa ANSGA + L174Bpa V102A + | 0,34731 | 0 |
| | | | | L174Bpa ANSGA + L174Bpa H134A - | 0,12771 | 0 |
| | | | | L174Bpa ANSGA + L174Bpa H134A + | 0,13667 | 0 |

| | | | | | | |
|---|---|---|---|---|---|---|
| | | | | L174Bpa ANSGA + L174Bpa V181K - | 1 | 0 |
| | | | | L174Bpa ANSGA + L174Bpa V181K + | 1 | 0 |
| | | | | L174Bpa ANSGA + L174Bpa P245L - | 0,99997 | 0 |
| | | | | L174Bpa ANSGA + L174Bpa P245L + | 1 | 0 |
| | | | | L174Bpa ANSGA + L174Bpa ANSGA - | 0,96925 | 0 |
| S8a STIM1+A254Azi XXX | Welch-ANOVA | 1,18E-08 | F(11, 25.27)=15.9 | + - | 0,41097 | 0 |
| | | | | A254Azi V102A - - | 0,24865 | 0 |
| | | | | A254Azi V102A - + | 0,02299 | 1 |
| | | | | A254Azi V102A + - | 0,25859 | 0 |
| | | | | A254Azi V102A + + | 0,02302 | 1 |
| | | | | A254Azi V102A + A254Azi V102A - | 1 | 0 |
| | | | | A254Azi H134A - - | 0,21823 | 0 |
| | | | | A254Azi H134A - + | 0,02977 | 1 |
| | | | | A254Azi H134A - A254Azi V102A - | 1 | 0 |
| | | | | A254Azi H134A - A254Azi V102A + | 1 | 0 |
| | | | | A254Azi H134A + - | 0,20839 | 0 |
| | | | | A254Azi H134A + + | 0,02943 | 1 |
| | | | | A254Azi H134A + A254Azi V102A - | 1 | 0 |
| | | | | A254Azi H134A + A254Azi V102A + | 1 | 0 |
| | | | | A254Azi H134A + A254Azi H134A - | 1 | 0 |
| | | | | A254Azi V181K - - | 0,50297 | 0 |
| | | | | A254Azi V181K - + | 0,03788 | 1 |
| | | | | A254Azi V181K - A254Azi V102A - | 0,9993 | 0 |
| | | | | A254Azi V181K - A254Azi V102A + | 0,99937 | 0 |
| | | | | A254Azi V181K - A254Azi H134A - | 0,99975 | 0 |
| | | | | A254Azi V181K - A254Azi H134A + | 0,99947 | 0 |
| | | | | A254Azi V181K + - | 0,505 | 0 |
| | | | | A254Azi V181K + + | 0,03815 | 1 |
| | | | | A254Azi V181K + A254Azi V102A - | 0,9992 | 0 |
| | | | | A254Azi V181K + A254Azi V102A + | 0,99928 | 0 |
| | | | | A254Azi V181K + A254Azi H134A - | 0,99969 | 0 |
| | | | | A254Azi V181K + A254Azi H134A + | 0,99936 | 0 |
| | | | | A254Azi V181K + A254Azi V181K - | 1 | 0 |
| | | | | A254Azi P245L - - | 1 | 0 |
| | | | | A254Azi P245L - + | 0,27266 | 0 |
| | | | | A254Azi P245L - A254Azi V102A - | 0,06726 | 0 |
| | | | | A254Azi P245L - A254Azi V102A + | 0,0792 | 0 |
| | | | | A254Azi P245L - A254Azi H134A - | 0,00239 | 1 |
| | | | | A254Azi P245L - A254Azi H134A + | 0,00193 | 1 |
| | | | | A254Azi P245L - A254Azi V181K - | 0,15435 | 0 |
| | | | | A254Azi P245L - A254Azi V181K + | 0,15372 | 0 |
| | | | | A254Azi P245L + - | 1 | 0 |
| | | | | A254Azi P245L + + | 0,34584 | 0 |
| | | | | A254Azi P245L + A254Azi V102A - | 0,04237 | 1 |
| | | | | A254Azi P245L + A254Azi V102A + | 0,0508 | 0 |
| | | | | A254Azi P245L + A254Azi H134A - | 3,55E-04 | 1 |
| | | | | A254Azi P245L + A254Azi H134A + | 2,61E-04 | 1 |
| | | | | A254Azi P245L + A254Azi V181K - | 0,07694 | 0 |
| | | | | A254Azi P245L + A254Azi V181K + | 0,07622 | 0 |
| | | | | A254Azi P245L + A254Azi P245L - | 0,99985 | 0 |
| | | | | A254Azi ANSGA - - | 0,08704 | 0 |
| | | | | A254Azi ANSGA - + | 0,01967 | 1 |
| | | | | A254Azi ANSGA - A254Azi V102A - | 0,98838 | 0 |
| | | | | A254Azi ANSGA - A254Azi V102A + | 0,99125 | 0 |
| | | | | A254Azi ANSGA - A254Azi H134A - | 0,55689 | 0 |
| | | | | A254Azi ANSGA - A254Azi H134A + | 0,56176 | 0 |
| | | | | A254Azi ANSGA - A254Azi V181K - | 0,68152 | 0 |
| | | | | A254Azi ANSGA - A254Azi V181K + | 0,66994 | 0 |
| | | | | A254Azi ANSGA - A254Azi P245L - | 3,29E-04 | 1 |
| | | | | A254Azi ANSGA - A254Azi P245L + | 3,90E-05 | 1 |
| | | | | A254Azi ANSGA + - | 0,9112 | 0 |
| | | | | A254Azi ANSGA + + | 1 | 0 |
| | | | | A254Azi ANSGA + A254Azi V102A - | 0,21218 | 0 |
| | | | | A254Azi ANSGA + A254Azi V102A + | 0,21331 | 0 |
| | | | | A254Azi ANSGA + A254Azi H134A - | 0,22342 | 0 |
| | | | | A254Azi ANSGA + A254Azi H134A + | 0,21885 | 0 |
| | | | | A254Azi ANSGA + A254Azi V181K - | 0,31179 | 0 |
| | | | | A254Azi ANSGA + A254Azi V181K + | 0,31296 | 0 |
| | | | | A254Azi ANSGA + A254Azi P245L - | 0,84678 | 0 |
| | | | | A254Azi ANSGA + A254Azi P245L + | 0,90587 | 0 |
| | | | | A254Azi ANSGA + A254Azi ANSGA - | 0,13927 | 0 |
| S8h Vrev Orai1 V102A XXX | one-way ANOVA | 0,00619 | F(13, 42) = 2,77341 | V102A STIM1 V102A | <0.0001 | 1 |
| | | | | A137Bpa V102A -UV V102A | 5,29E-04 | 1 |
| | | | | A137Bpa V102A -UV V102A STIM1 | 0,25971 | 0 |
| | | | | A137Bpa V102A +UV V102A | 0,00118 | 1 |
| | | | | A137Bpa V102A +UV V102A STIM1 | 0,16457 | 0 |
| | | | | A137Bpa V102A +UV A137Bpa V102A -UV | 0,79761 | 0 |
| | | | | A137Bpa V102A+STIM1 -UV V102A | 0,00535 | 1 |
| | | | | A137Bpa V102A+STIM1 -UV V102A STIM1 | 0,05682 | 0 |
| | | | | A137Bpa V102A+STIM1 -UV A137Bpa V102A -UV | 0,44314 | 0 |
| | | | | A137Bpa V102A+STIM1 -UV A137Bpa V102A +UV | 0,60846 | 0 |
| | | | | A137Bpa V102A +STIM1+UV V102A | 0,01085 | 1 |
| | | | | A137Bpa V102A +STIM1+UV V102A STIM1 | 0,0311 | 1 |
| | | | | A137Bpa V102A +STIM1+UV A137Bpa V102A -UV | 0,30785 | 0 |
| | | | | A137Bpa V102A +STIM1+UV A137Bpa V102A +UV | 0,44314 | 0 |
| | | | | A137Bpa V102A +STIM1+UV A137Bpa V102A+STIM1 -UV | 0,79761 | 0 |
| | | | | L174Bpa V102A-UV V102A | 3,79E-04 | 1 |
| | | | | L174Bpa V102A-UV V102A STIM1 | 0,53016 | 0 |
| | | | | L174Bpa V102A-UV A137Bpa V102A -UV | 0,69249 | 0 |
| | | | | L174Bpa V102A-UV A137Bpa V102A +UV | 0,52749 | 0 |
| | | | | L174Bpa V102A-UV A137Bpa V102A+STIM1 -UV | 0,2712 | 0 |
| | | | | L174Bpa V102A-UV A137Bpa V102A +STIM1+UV | 0,18301 | 0 |
| | | | | L174Bpa V102A+UV V102A | 4,87E-04 | 1 |
| | | | | L174Bpa V102A+UV V102A STIM1 | 0,47777 | 0 |
| | | | | L174Bpa V102A+UV A137Bpa V102A -UV | 0,75163 | 0 |
| | | | | L174Bpa V102A+UV A137Bpa V102A +UV | 0,58015 | 0 |
| | | | | L174Bpa V102A+UV A137Bpa V102A+STIM1 -UV | 0,30643 | 0 |
| | | | | L174Bpa V102A+UV A137Bpa V102A +STIM1+UV | 0,20957 | 0 |
| | | | | L174Bpa V102A+UV L174Bpa V102A-UV | 0,94097 | 0 |
| | | | | L174Bpa V102A+STIM1-UV V102A | 0,0033 | 1 |
| | | | | L174Bpa V102A+STIM1-UV V102A STIM1 | 0,04391 | 1 |
| | | | | L174Bpa V102A+STIM1-UV A137Bpa V102A -UV | 0,41906 | 0 |
| | | | | L174Bpa V102A+STIM1-UV A137Bpa V102A +UV | 0,58928 | 0 |
| | | | | L174Bpa V102A+STIM1-UV A137Bpa V102A+STIM1 -UV | 1 | 0 |
| | | | | L174Bpa V102A+STIM1-UV A137Bpa V102A +STIM1+UV | 0,78693 | 0 |
| | | | | L174Bpa V102A+STIM1-UV L174Bpa V102A-UV | 0,25016 | 0 |
| | | | | L174Bpa V102A+STIM1-UV L174Bpa V102A+UV | 0,28508 | 0 |
| | | | | L174Bpa V102A+STIM1+UV V102A | 0,0033 | 1 |
| | | | | L174Bpa V102A+STIM1+UV V102A STIM1 | 0,04391 | 1 |
| | | | | L174Bpa V102A+STIM1+UV A137Bpa V102A -UV | 0,41906 | 0 |
| | | | | L174Bpa V102A+STIM1+UV A137Bpa V102A +UV | 0,58928 | 0 |
| | | | | L174Bpa V102A+STIM1+UV A137Bpa V102A+STIM1 -UV | 1 | 0 |
| | | | | L174Bpa V102A+STIM1+UV A137Bpa V102A +STIM1+UV | 0,78693 | 0 |

| | | | | | | |
|---|---|---|---|---|---|---|
| | | | | L174Bpa V102A+STIM1+UV  L174Bpa V102A-UV | 0,25016 | 0 |
| | | | | L174Bpa V102A+STIM1+UV  L174Bpa V102A+UV | 0,28508 | 0 |
| | | | | L174Bpa V102A+STIM1+UV  L174Bpa V102A+STIM1-UV | 1 | 0 |
| | | | | A254Azi V102A -UV  V102A | 3,79E-04 | 1 |
| | | | | A254Azi V102A -UV  V102A STIM1 | 0,53016 | 0 |
| | | | | A254Azi V102A -UV  A137Bpa V102A -UV | 0,69249 | 0 |
| | | | | A254Azi V102A -UV  A137Bpa V102A +UV | 0,52749 | 0 |
| | | | | A254Azi V102A -UV  A137Bpa V102A+STIM1 -UV | 0,2712 | 0 |
| | | | | A254Azi V102A -UV  A137Bpa V102A +STIM1+UV | 0,18301 | 0 |
| | | | | A254Azi V102A -UV  L174Bpa V102A-UV | 1 | 0 |
| | | | | A254Azi V102A -UV  L174Bpa V102A+UV | 0,94097 | 0 |
| | | | | A254Azi V102A -UV  L174Bpa V102A+STIM1-UV | 0,25016 | 0 |
| | | | | A254Azi V102A -UV  L174Bpa V102A+STIM1+UV | 0,25016 | 0 |
| | | | | A254Azi V102A +UV  V102A | 0,00164 | 1 |
| | | | | A254Azi V102A +UV  V102A STIM1 | 0,26374 | 0 |
| | | | | A254Azi V102A +UV  A137Bpa V102A -UV | 0,9369 | 0 |
| | | | | A254Azi V102A +UV  A137Bpa V102A +UV | 0,87421 | 0 |
| | | | | A254Azi V102A +UV  A137Bpa V102A+STIM1 -UV | 0,52749 | 0 |
| | | | | A254Azi V102A +UV  A137Bpa V102A +STIM1+UV | 0,38598 | 0 |
| | | | | A254Azi V102A +UV  L174Bpa V102A-UV | 0,65718 | 0 |
| | | | | A254Azi V102A +UV  L174Bpa V102A+UV | 0,7114 | 0 |
| | | | | A254Azi V102A +UV  L174Bpa V102A+STIM1-UV | 0,50884 | 0 |
| | | | | A254Azi V102A +UV  L174Bpa V102A+STIM1+UV | 0,50884 | 0 |
| | | | | A254Azi V102A +UV  A254Azi V102A -UV | 0,65718 | 0 |
| | | | | A254Azi V102A+STIM1 -UV  V102A | 7,91E-04 | 1 |
| | | | | A254Azi V102A+STIM1 -UV  V102A STIM1 | 0,20808 | 0 |
| | | | | A254Azi V102A+STIM1 -UV  A137Bpa V102A -UV | 0,89795 | 0 |
| | | | | A254Azi V102A+STIM1 -UV  A137Bpa V102A +UV | 0,89795 | 0 |
| | | | | A254Azi V102A+STIM1 -UV  A137Bpa V102A+STIM1 -UV | 0,52232 | 0 |
| | | | | A254Azi V102A+STIM1 -UV  A137Bpa V102A +STIM1+UV | 0,37155 | 0 |
| | | | | A254Azi V102A+STIM1 -UV  L174Bpa V102A-UV | 0,6074 | 0 |
| | | | | A254Azi V102A+STIM1 -UV  L174Bpa V102A+UV | 0,66361 | 0 |
| | | | | A254Azi V102A+STIM1 -UV  L174Bpa V102A+STIM1-UV | 0,50019 | 0 |
| | | | | A254Azi V102A+STIM1 -UV  L174Bpa V102A+STIM1+UV | 0,50019 | 0 |
| | | | | A254Azi V102A+STIM1 -UV  A254Azi V102A -UV | 0,6074 | 0 |
| | | | | A254Azi V102A+STIM1 -UV  A254Azi V102A +UV | 0,96842 | 0 |
| | | | | A254Azi V102A +STIM1+UV  V102A | 5,29E-04 | 1 |
| | | | | A254Azi V102A +STIM1+UV  V102A STIM1 | 0,25971 | 0 |
| | | | | A254Azi V102A +STIM1+UV  A137Bpa V102A -UV | 1 | 0 |
| | | | | A254Azi V102A +STIM1+UV  A137Bpa V102A +UV | 0,79761 | 0 |
| | | | | A254Azi V102A +STIM1+UV  A137Bpa V102A+STIM1 -UV | 0,44314 | 0 |
| | | | | A254Azi V102A +STIM1+UV  A137Bpa V102A +STIM1+UV | 0,30785 | 0 |
| | | | | A254Azi V102A +STIM1+UV  L174Bpa V102A-UV | 0,69249 | 0 |
| | | | | A254Azi V102A +STIM1+UV  L174Bpa V102A+UV | 0,75163 | 0 |
| | | | | A254Azi V102A +STIM1+UV  L174Bpa V102A+STIM1-UV | 0,41906 | 0 |
| | | | | A254Azi V102A +STIM1+UV  L174Bpa V102A+STIM1+UV | 0,41906 | 0 |
| | | | | A254Azi V102A +STIM1+UV  A254Azi V102A -UV | 0,69249 | 0 |
| | | | | A254Azi V102A +STIM1+UV  A254Azi V102A +UV | 0,9369 | 0 |
| | | | | A254Azi V102A +STIM1+UV  A254Azi V102A+STIM1 -UV | 0,89795 | 0 |
| S9c Before UV | Welch-ANOVA | 3,49E-05 | F(8, 25.15)=7.73 | 47 - | 0,99995 | 0 |
| | | | | 60 - | 0,99968 | 0 |
| | | | | 60 47 | 1 | 0 |
| | | | | 64 - | 0,70976 | 0 |
| | | | | 64 47 | 0,80289 | 0 |
| | | | | 64 60 | 0,853 | 0 |
| | | | | 68 - | 0,99051 | 0 |
| | | | | 68 47 | 0,9991 | 0 |
| | | | | 68 60 | 0,99981 | 0 |
| | | | | 68 64 | 0,99097 | 0 |
| | | | | 70 - | 0,99976 | 0 |
| | | | | 70 47 | 1 | 0 |
| | | | | 70 60 | 1 | 0 |
| | | | | 70 64 | 0,80493 | 0 |
| | | | | 70 68 | 0,99941 | 0 |
| | | | | 71 - | 1 | 0 |
| | | | | 71 47 | 0,99944 | 0 |
| | | | | 71 60 | 0,99696 | 0 |
| | | | | 71 64 | 0,58132 | 0 |
| | | | | 71 68 | 0,97091 | 0 |
| | | | | 71 70 | 0,9968 | 0 |
| | | | | 72 - | 0,5517 | 0 |
| | | | | 72 47 | 0,09426 | 0 |
| | | | | 72 60 | 0,10063 | 0 |
| | | | | 72 64 | 0,08821 | 0 |
| | | | | 72 68 | 0,30294 | 0 |
| | | | | 72 70 | 0,02458 | 1 |
| | | | | 72 71 | 0,23168 | 0 |
| | | | | 78 - | 0,55766 | 0 |
| | | | | 78 47 | 0,09603 | 0 |
| | | | | 78 60 | 0,10186 | 0 |
| | | | | 78 64 | 0,08875 | 0 |
| | | | | 78 68 | 0,30477 | 0 |
| | | | | 78 70 | 0,02509 | 1 |
| | | | | 78 71 | 0,23488 | 0 |
| | | | | 78 72 | 1 | 0 |
| S9c After UV | Welch-ANOVA | 3,92E-09 | F(8, 26.08)=19.43 | 47 - | 0,99685 | 0 |
| | | | | 60 - | 0,77091 | 0 |
| | | | | 60 47 | 0,97761 | 0 |
| | | | | 64 - | 0,85599 | 0 |
| | | | | 64 47 | 0,98592 | 0 |
| | | | | 64 60 | 1 | 0 |
| | | | | 68 - | 0,99571 | 0 |
| | | | | 68 47 | 0,56697 | 0 |
| | | | | 68 60 | 0,13304 | 0 |
| | | | | 68 64 | 0,376 | 0 |
| | | | | 70 - | 0,75142 | 0 |
| | | | | 70 47 | 0,10994 | 0 |
| | | | | 70 60 | 0,02556 | 1 |
| | | | | 70 64 | 0,1453 | 0 |
| | | | | 70 68 | 0,84518 | 0 |
| | | | | 71 - | 0,13653 | 0 |
| | | | | 71 47 | 0,00872 | 1 |
| | | | | 71 60 | 0,00443 | 1 |
| | | | | 71 64 | 0,03624 | 1 |
| | | | | 71 68 | 0,06143 | 0 |
| | | | | 71 70 | 0,08806 | 0 |
| | | | | 72 - | 0,0419 | 1 |
| | | | | 72 47 | 0,00319 | 1 |
| | | | | 72 60 | 0,00254 | 1 |
| | | | | 72 64 | 0,01914 | 1 |
| | | | | 72 68 | 0,02101 | 1 |
| | | | | 72 70 | 0,00243 | 1 |

| | | | | | | |
|---|---|---|---|---|---|---|
| | | | | 72 71 | 0,28146 | 0 |
| | | | | 78 - | 0,04646 | 1 |
| | | | | 78 47 | 0,00348 | 1 |
| | | | | 78 60 | 0,00268 | 1 |
| | | | | 78 64 | 0,02023 | 1 |
| | | | | 78 68 | 0,02281 | 1 |
| | | | | 78 70 | 0,00314 | 1 |
| | | | | 78 71 | 0,37274 | 0 |
| | | | | 78 72 | 0,99994 | 0 |
| S9d After UV | Welch-ANOVA | 1,03E-05 | F(8, 24.85)=8.94 | 47 - | 1 | 0 |
| | | | | 60 - | 1 | 0 |
| | | | | 60 47 | 1 | 0 |
| | | | | 64 - | 0,99998 | 0 |
| | | | | 64 47 | 1 | 0 |
| | | | | 64 60 | 1 | 0 |
| | | | | 68 - | 0,32212 | 0 |
| | | | | 68 47 | 0,54565 | 0 |
| | | | | 68 60 | 0,95646 | 0 |
| | | | | 68 64 | 0,76226 | 0 |
| | | | | 70 - | 0,04561 | 1 |
| | | | | 70 47 | 0,06274 | 0 |
| | | | | 70 60 | 0,65203 | 0 |
| | | | | 70 64 | 0,1402 | 0 |
| | | | | 70 68 | 0,58464 | 0 |
| | | | | 71 - | 0,03448 | 1 |
| | | | | 71 47 | 0,0426 | 1 |
| | | | | 71 60 | 0,58552 | 0 |
| | | | | 71 64 | 0,0999 | 0 |
| | | | | 71 68 | 0,41248 | 0 |
| | | | | 71 70 | 0,75934 | 0 |
| | | | | 72 - | 0,02624 | 1 |
| | | | | 72 47 | 0,027 | 1 |
| | | | | 72 60 | 0,51074 | 0 |
| | | | | 72 64 | 0,06665 | 0 |
| | | | | 72 68 | 0,25673 | 0 |
| | | | | 72 70 | 0,01838 | 1 |
| | | | | 72 71 | 0,62601 | 0 |
| | | | | 78 - | 0,0271 | 1 |
| | | | | 78 47 | 0,02901 | 1 |
| | | | | 78 60 | 0,52232 | 0 |
| | | | | 78 64 | 0,07109 | 0 |
| | | | | 78 68 | 0,27801 | 0 |
| | | | | 78 70 | 0,07493 | 0 |
| | | | | 78 71 | 0,83303 | 0 |
| | | | | 78 72 | 0,99999 | 0 |
| S9e After UV | Welch-ANOVA | 1,09E-05 | F(7, 16.83)=12.89 | 47 - | 1 | 0 |
| | | | | 60 - | 0,14534 | 0 |
| | | | | 60 47 | 0,18222 | 0 |
| | | | | 64 - | 1 | 0 |
| | | | | 64 47 | 1 | 0 |
| | | | | 64 60 | 0,17943 | 0 |
| | | | | 68 - | 0,65248 | 0 |
| | | | | 68 47 | 0,8124 | 0 |
| | | | | 68 60 | 0,03237 | 1 |
| | | | | 68 64 | 0,80979 | 0 |
| | | | | 70 - | 0,01266 | 1 |
| | | | | 70 47 | 0,20018 | 0 |
| | | | | 70 60 | 0,00464 | 1 |
| | | | | 70 64 | 0,1605 | 0 |
| | | | | 70 68 | 0,96694 | 0 |
| | | | | 72 - | 0,00108 | 1 |
| | | | | 72 47 | 0,07791 | 0 |
| | | | | 72 60 | 0,00241 | 1 |
| | | | | 72 64 | 0,04632 | 1 |
| | | | | 72 68 | 0,67322 | 0 |
| | | | | 72 70 | 0,29288 | 0 |
| | | | | 78 - | 0,0014 | 1 |
| | | | | 78 47 | 0,08729 | 0 |
| | | | | 78 60 | 0,00258 | 1 |
| | | | | 78 64 | 0,05437 | 0 |
| | | | | 78 68 | 0,72329 | 0 |
| | | | | 78 70 | 0,41813 | 0 |
| | | | | 78 72 | 0,98607 | 0 |
| S11c -70mV | one-way ANOVA | 0,00418 | F(3, 26)=5.61 | A137Bpa Ca WT Ca | 0,55556 | 0 |
| | | | | WT Na WT Ca | 0,0056 | 1 |
| | | | | WT Na A137Bpa Ca | 0,03458 | 1 |
| | | | | A137Bpa Na WT Ca | 0,00201 | 1 |
| | | | | A137Bpa Na A137Bpa Ca | 0,01689 | 1 |
| | | | | A137Bpa Na WT Na | 0,83671 | 0 |
| S11c -90mV | Welch-ANOVA | 2,14E-04 | F(3, 11.38)=16.6 | A137Bpa Ca WT Ca | 0,14437 | 0 |
| | | | | WT Na WT Ca | 0,00455 | 1 |
| | | | | WT Na A137Bpa Ca | 0,00574 | 1 |
| | | | | A137Bpa Na WT Ca | 0,00477 | 1 |
| | | | | A137Bpa Na A137Bpa Ca | 0,0063 | 1 |
| | | | | A137Bpa Na WT Na | 0,993 | 0 |
| S11c -110mV | one-way ANOVA | <0.0001 | F(3, 29)=50.6 | A137Bpa Ca WT Ca | <0.0001 | 1 |
| | | | | WT Na WT Ca | <0.0001 | 1 |
| | | | | WT Na A137Bpa Ca | <0.0001 | 1 |
| | | | | A137Bpa Na WT Ca | <0.0001 | 1 |
| | | | | A137Bpa Na A137Bpa Ca | <0.0001 | 1 |
| | | | | A137Bpa Na WT Na | 0,68826 | 0 |
| S11d -70mV | one-way ANOVA | 0,00323 | F(3, 21)=6.3 | A137Bpa Ca WT Ca | 0,004 | 1 |
| | | | | WT Na WT Ca | 0,99344 | 0 |
| | | | | WT Na A137Bpa Ca | 0,00391 | 1 |
| | | | | A137Bpa Na WT Ca | 0,9748 | 0 |
| | | | | A137Bpa Na A137Bpa Ca | 0,00236 | 1 |
| | | | | A137Bpa Na WT Na | 0,98164 | 0 |
| S11d -90mV | one-way ANOVA | <0.0001 | F(3, 21)=13.95 | A137Bpa Ca WT Ca | 0,00102 | 1 |
| | | | | WT Na WT Ca | 0,32708 | 0 |
| | | | | WT Na A137Bpa Ca | <0.0001 | 1 |
| | | | | A137Bpa Na WT Ca | 0,18936 | 0 |
| | | | | A137Bpa Na A137Bpa Ca | <0.0001 | 1 |
| | | | | A137Bpa Na WT Na | 0,76081 | 0 |

| | | | | | | |
|---|---|---|---|---|---|---|
| S11d -110mV | one-way ANOVA | <0.0001 | F(3, 21)=23.71 | A137Bpa Ca  WT Ca | <0.0001 | 1 |
| | | | | WT Na  WT Ca | 0,304 | 0 |
| | | | | WT Na  A137Bpa Ca | <0.0001 | 1 |
| | | | | A137Bpa Na  WT Ca | 0,10072 | 0 |
| | | | | A137Bpa Na  A137Bpa Ca | <0.0001 | 1 |
| | | | | A137Bpa Na  WT Na | 0,5443 | 0 |
