# Supplementary material for: Photocrosslinking-induced CRAC channel-like Orai1 activation independent of STIM1
Source: Nat Commun. 2023 Mar 8;14:1286. doi: 10.1038/s41467-023-36458-4 (PMC9995687; doi:10.1038/s41467-023-36458-4)
Supplement: Supplementary file 3 — Reporting Summary [file 41467_2023_36458_MOESM3_ESM.pdf]

nature portfolio

Corresponding author(s): Isabella Derler

Last updated by author(s): Jan 18, 2023

Reporting Summary

Nature Portfolio wishes to improve the reproducibility of the work that we publish. This form provides structure for consistency and transparency in reporting. For further information on Nature Portfolio policies, see our Editorial Policies and the Editorial Policy Checklist.

Statistics

For all statistical analyses, confirm that the following items are present in the figure legend, table legend, main text, or Methods section.

n/a	Confirmed	
☐	☒	The exact sample size (n) for each experimental group/condition, given as a discrete number and unit of measurement
☐	☒	A statement on whether measurements were taken from distinct samples or whether the same sample was measured repeatedly
☐	☒	The statistical test(s) used AND whether they are one- or two-sided<br>*Only common tests should be described solely by name; describe more complex techniques in the Methods section.*
☒	☐	A description of all covariates tested
☐	☒	A description of any assumptions or corrections, such as tests of normality and adjustment for multiple comparisons
☐	☒	A full description of the statistical parameters including central tendency (e.g. means) or other basic estimates (e.g. regression coefficient) AND variation (e.g. standard deviation) or associated estimates of uncertainty (e.g. confidence intervals)
☐	☒	For null hypothesis testing, the test statistic (e.g. F, t, r) with confidence intervals, effect sizes, degrees of freedom and P value noted<br>*Give P values as exact values whenever suitable.*
☒	☐	For Bayesian analysis, information on the choice of priors and Markov chain Monte Carlo settings
☒	☐	For hierarchical and complex designs, identification of the appropriate level for tests and full reporting of outcomes
☒	☐	Estimates of effect sizes (e.g. Cohen's d, Pearson's r), indicating how they were calculated

Our web collection on statistics for biologists contains articles on many of the points above.

Software and code

Policy information about availability of computer code

Data collection	pClamp11 (Clampfit), VisiView (5.0.0.0 and v2.1.4, Visitron Systems)
Data analysis	Origin 2022 (OriginLab Corporation, USA); Microsoft Excel, MATLAB (v7.11.0, The MathWorks, Inc., MA, USA), PyMOL (v. 2.3, Schrödinger, LLC)

For manuscripts utilizing custom algorithms or software that are central to the research but not yet described in published literature, software must be made available to editors and reviewers. We strongly encourage code deposition in a community repository (e.g. GitHub). See the Nature Portfolio guidelines for submitting code & software for further information.

Data

Policy information about availability of data

All manuscripts must include a data availability statement. This statement should provide the following information, where applicable:

- Accession codes, unique identifiers, or web links for publicly available datasets
- A description of any restrictions on data availability
- For clinical datasets or third party data, please ensure that the statement adheres to our policy

All relevant data are available in this article and its supplementary information files, and source data are provided with this paper as source data file and are deposited in an open public repository [https://doi.org/ 10.5281/zenodo.7551827]

Human research participants

Policy information about studies involving human research participants and Sex and Gender in Research.

Reporting on sex and gender	The findings do not apply to one sex or gender.
Population characteristics	No human research participants contributed to the study.
Recruitment	not applicable
Ethics oversight	not applicable

Note that full information on the approval of the study protocol must also be provided in the manuscript.

Field-specific reporting

Please select the one below that is the best fit for your research. If you are not sure, read the appropriate sections before making your selection.

☒ Life sciences ☐ Behavioural & social sciences ☐ Ecological, evolutionary & environmental sciences

For a reference copy of the document with all sections, see nature.com/documents/nr-reporting-summary-flat.pdf

Life sciences study design

All studies must disclose on these points even when the disclosure is negative.

Sample size	Conventional, power-based sample size estimation for two-tailed Student´s t-test (OriginPro 2022, Origin Lab) confirmed our experience from previous studies, suggesting a sample size of 5 – 10 (measurements in individual cells) as sufficient to detect relevant differences in our electrophysiological settings at α of 0.05 and a power of of 0.8., while relevant sample size in Ca2+ imaging and electrophysiology is the range of 30 or higher.
Data exclusions	Current recordings displaying initial leak currents were excluded in patch clamp experiments based on the observation of a time and intervention-independent linear noisy current-voltage relationship. This exclusion criteria was pre-established.
Replication	Live cell experiments were replicated on at least twice on two different days using independent transfections with the indicated number of cells (n).
Randomization	The detailed procedure for our experiments is as follows: The experiments are performed on living cells from cell culture. The cells were seeded in Petri dishes. For each Petri dish, we transfected a different mutant, while one Petri dish contained the control (e.g., wild-type transfection). All experiments of transfected cells in a Petri dish represent one group, or in other words, one group was formed from all experiments within a given transfection (transfected cells in a Petri dish). Each experiment in this group is analyzed and all experiments are used to calculate the mean, which are shown in the individual graphs. Experiments of different mutants (groups) are performed on at least two different days in paired comparison. Each mean curve includes the specified number n of measurements of the same mutant. The incorporation of the correct mutation into the DNA sequence was checked by sequencing before transfection.<br>Regarding randomization: Since transfected mutants are self-produced, we know which mutant is transfected in which Petri dish. However, we have no expectation of the outcome of the experimental design for the individual mutants. All experiments in one Petri dish (one transfection) are considered for one group. After analysis of single cells, all data of one group are used to calculate the mean and distinct groups are compared. Cells expressing either wild-type or mutant constructs were selected based on the fluorescence of the heterologously expressed CFP/YFP-labeled constructs. Constructs were alternately expressed in the available passages of host cells (HEK293).
Blinding	Investigators were typically not blinded when performing the live cell recording experiments. Blinding does not apply to such electrophysiological and FRET microscopy measurements, as we do not have any expectation of the outcome of the experimental setup of individual mutants.

Reporting for specific materials, systems and methods

We require information from authors about some types of materials, experimental systems and methods used in many studies. Here, indicate whether each material, system or method listed is relevant to your study. If you are not sure if a list item applies to your research, read the appropriate section before selecting a response.

Materials & experimental systems

n/a	Involved in the study	
☒	☐	Antibodies
☐	☒	Eukaryotic cell lines
☒	☐	Palaeontology and archaeology
☒	☐	Animals and other organisms
☒	☐	Clinical data
☒	☐	Dual use research of concern

Methods

n/a	Involved in the study	
☒	☐	ChIP-seq
☒	☐	Flow cytometry
☒	☐	MRI-based neuroimaging

Eukaryotic cell lines

Policy information about cell lines and Sex and Gender in Research

Cell line source(s)	HEK293 cells (ACC305) from DSMZ (Germany)
Authentication	Authentication is provided by DSMZ using multiplex PCR of minisatellite markers revealing a unique DNA profile
Mycoplasma contamination	HEK293 cells were regularly (every 3 months) tested for mycoplasm contamination and were negative.
Commonly misidentified lines (See ICLAC register)	No misidentified cell line used.
